# Pursuing Advances in DNA Sequencing Technology to Solve a Complex Genomic Jigsaw Puzzle: The Agglutinin-Like Sequence (*ALS*) Genes of *Candida tropicalis*

**DOI:** 10.3389/fmicb.2020.594531

**Published:** 2021-01-20

**Authors:** Soon-Hwan Oh, Allyson Isenhower, Rubi Rodriguez-Bobadilla, Brooke Smith, Jillian Jones, Vit Hubka, Christopher Fields, Alvaro Hernandez, Lois L. Hoyer

**Affiliations:** ^1^Department of Pathobiology, College of Veterinary Medicine, University of Illinois at Urbana-Champaign, Urbana, IL, United States; ^2^Department of Biology, Millikin University, Decatur, IL, United States; ^3^Department of Botany, Faculty of Science, Charles University, Prague, Czechia; ^4^Laboratory of Fungal Genetics and Metabolism, Institute of Microbiology, Czech Academy of Sciences, Prague, Czechia; ^5^Roy J. Carver Biotechnology Center, University of Illinois at Urbana-Champaign, Urbana, IL, United States

**Keywords:** genome, *Candida tropicalis*, *ALS* genes, gene expression, fungal adhesion

## Abstract

The agglutinin-like sequence (*ALS*) gene family encodes cell-surface adhesins that interact with host and abiotic surfaces, promoting colonization by opportunistic fungal pathogens such as *Candida tropicalis*. Studies of Als protein contribution to *C. tropicalis* adhesion would benefit from an accurate catalog of *ALS* gene sequences as well as insight into relative gene expression levels. Even in the genomics era, this information has been elusive: genome assemblies are often broken within *ALS* genes because of their extensive regions of highly conserved, repeated DNA sequences and because there are many similar *ALS* genes at different chromosomal locations. Here, we describe the benefit of long-read DNA sequencing technology to facilitate characterization of *C. tropicalis ALS* loci. Thirteen *ALS* loci in *C. tropicalis* strain MYA-3404 were deduced from a genome assembly constructed from Illumina MiSeq and Oxford Nanopore MinION data. Although the MinION data were valuable, PCR amplification and Sanger sequencing of *ALS* loci were still required to complete and verify the gene sequences. Each predicted Als protein featured an N-terminal binding domain, a central domain of tandemly repeated sequences, and a C-terminal domain rich in Ser and Thr. The presence of a secretory signal peptide and consensus sequence for addition of a glycosylphosphatidylinositol (GPI) anchor was consistent with predicted protein localization to the cell surface. TaqMan assays were designed to recognize each *ALS* gene, as well as both alleles at the divergent *CtrALS3882* locus. *C. tropicalis* cells grown in five different *in vitro* conditions showed differential expression of various *ALS* genes. To place the *C. tropicalis* data into a larger context, TaqMan assays were also designed and validated for analysis of *ALS* gene expression in *Candida albicans* and *Candida dubliniensis*. These comparisons identified the subset of highly expressed *C. tropicalis ALS* genes that were predicted to encode proteins with the most abundant cell-surface presence, prioritizing them for subsequent functional analysis. Data presented here provide a solid foundation for future experimentation to deduce *ALS* family contributions to *C. tropicalis* adhesion and pathogenesis.

## Introduction

Adhesion and subsequent colonization provide the opportunity for microbial pathogens to cause disease. Identification of adhesin-encoding genes is facilitated by the availability of genome sequences. An accurate catalog of potential adhesins focuses efforts to characterize protein function and develop therapeutic approaches that could be effective against multiple microbial species. Anti-adhesion therapies have been hailed as superior to traditional antimicrobials because their mechanism of action does not promote antimicrobial resistance (Krachler and Orth, 2013). This report describes characterization of genes encoding the agglutinin-like sequence (Als) family of adhesins in the fungal pathogen *Candida tropicalis*. The work highlights how emerging advances in DNA sequencing technology were used to fill key knowledge gaps that have persisted for many years.

Agglutinin-like sequence (*ALS*) genes encode cell-surface adhesins that recognize a broad variety of peptide ligands (Lin et al., 2014). The gene family was characterized initially in *Candida albicans* with investigations into protein structure and activity also focused on this species (reviewed in Hoyer and Cota, 2016). Starting in the pre-genome era and extending over a period of approximately 20 years, projects moved from identification of the first *ALS* gene, to the recognition that the gene was part of a larger family, to characterization of the family, and the relative abundance of specific Als proteins on the cell surface under a variety of growth conditions. More recently, the structure of the N-terminal Als protein adhesive domain was solved (NT-Als; reviewed in Hoyer and Cota, 2016). Experimental progress to define the *C. albicans ALS* family was slow because detection of additional *ALS* genes relied on techniques such as the Southern blotting of genomic DNA (Hoyer et al., 1995, 1998a,b). Locating *ALS* genes in draft genome sequences would accelerate progress substantially.

As emerging genome sequencing technologies were applied to pathogenic fungal species, the *C. albicans ALS* sequences were used to recognize potential *ALS* genes in draft genomes (Butler et al., 2009). *ALS* gene hallmarks such as the 5′ end sequences that encode the adhesive domain, central regions of a conserved 108-bp tandemly repeated sequence, and the 3′ end sequences that include a glycosylphosphatidylinositol (GPI) anchor addition site were clues for identifying possible *ALS* loci. While these techniques could quickly identify potential *ALS* genes, the large *ALS* gene size and considerable sequence identity across multiple physical loci in the same species complicated accurate assembly. Computational assemblies broke within the conserved tandemly repeated sequences in the middle of the coding region, or mistakenly placed the 5′ end of one gene onto the 3′ end of another. The research community was left without accurate knowledge of the *ALS* gene number or sequence in common fungal pathogens.

Like *C. albicans*, *C. tropicalis* causes superficial mucosal infections, as well as disseminated and deep-seated infections; the rising incidence of azole-resistant *C. tropicalis* is causing clinical concern (reviewed in de Oliveira et al., 2020). Previous reports about *C. tropicalis ALS* genes suggested that the family is larger in this species than in other common fungal pathogens (Butler et al., 2009; Jackson et al., 2009), representing perhaps the most challenging *ALS* family puzzle to solve. Short-read sequence technologies were not helpful in this regard because the read lengths often did not include unique information that could place *ALS* sequences in the correct physical location.

One key technological development that appeared at the start of this project was the ability to derive long-read DNA sequences. Here, we describe combining short-read (Illumina MiSeq) and long-read (Oxford Nanopore MinION) datasets to develop a *C. tropicalis* genome assembly that served as the foundation for characterization of the 13 *ALS* loci in strain MYA-3404. Accurate gene sequences facilitated design of a specific TaqMan assay for each *ALS* gene. The assays were used to assess relative gene expression under a variety of growth conditions to reveal which *ALS* genes were likely to produce the most abundant Als cell-surface proteins, thereby prioritizing them for further functional analysis toward the development of anti-adhesion therapies.

## Materials and Methods

### Fungal Strains

Table 1 shows the fungal strains used in this study. *C. tropicalis* strains were authenticated using several methods (data not shown). First, each isolate was streaked on CHROMagar *Candida* medium (Becton Dickinson) to ensure that it produced the expected blue colony color. Molecular verification of strain identification used PCR primers ITS4 (5′ TCCTCCGCTTATTGATATGC 3′) and ITS5 (5′ GGAAGTAAAAGTCGTAACAAGG 3′; White et al., 1990). PCR products were Sanger sequenced at the Roy J. Carver Biotechnology Center, University of Illinois at Urbana-Champaign. DNA sequences were confirmed as *C. tropicalis* by comparison to the non-redundant nucleotide database at the National Center for Biotechnology Information using the blastn algorithm^1^. Karyotypes for the *C. tropicalis* strains were also investigated using contour-clamped homogeneous electrical field (CHEF) gels and found to be similar across the isolates; methods for the karyotype analysis and examples of the results were published previously (Hoyer et al., 2001).

**TABLE 1:** Fungal strains and sources.

Species	Strain	Source
*Candida tropicalis*	951 (CAPG-3)	Pat Kammeyer, Loyola University Medical Center
	952 (T60700)	Pat Kammeyer, Loyola University Medical Center
	1019 (ATCC 13803)	American Type Culture Collection
	1020 (ATCC 201380)	American Type Culture Collection
	1021 (ATCC 201381)	American Type Culture Collection
	3242 (NRRL Y-5716)	Agricultural Research Service Culture Collection
	3528 (MYA-3404)	David Soll, University of Iowa
*Candida albicans*	SC5314	American Type Culture Collection
*Candida dubliniensis*	CD36	David Coleman, Trinity College Dublin

### *C. tropicalis* Genome Sequences

A previously published *C. tropicalis* genome sequence was used as the basis for initial identification of putative *ALS* genes (strain MYA-3404; ASM633v3; GCA_000006335.3; Butler et al., 2009). A new genome sequence was generated for *C. tropicalis* strain MYA-3404. Methods for this effort were similar to the methods for generating a genomic sequence for *Candida metapsilosis* (Oh et al., 2019) and were reproduced here for the reader’s convenience.

Cells were grown in YPD (per liter: 10 g yeast extract, 20 g Bacto peptone, 20 g dextrose) to saturation (approximately 16 h at 37°C and 200 r/min shaking). Genomic DNA was isolated according to Sherman et al. (1986). The method involved zymolyase spheroplasting of cells, sodium dodecyl sulfate lysis, phenol extraction, DNA precipitation with isoproposal, and Proteinase K treatment of the final preparation. Gentle mixing and pipetting with wide-bore tips were used to minimize DNA shearing. High-molecular-weight DNA was visualized by agarose gel electrophoresis and ethidium-bromide staining prior to further processing.

Strain MYA-3404 libraries were constructed and sequenced at the Roy J. Carver Biotechnology Center, University of Illinois at Urbana-Champaign. Data were derived using Illumina (short-read) and Oxford Nanopore (long-read) methods. MiSeq shotgun libraries were prepared with the Hyper Library construction kit (Kapa Biosystems). The library was quantitated by qPCR and sequenced on one MiSeq flowcell for 151 cycles from each end of the fragment using a MiSeq 300-cycle sequencing kit (version 2). FASTQ files were generated and demultiplexed with the bclfastq Conversion Software (Illumina, version 2.17.1.14). MiSeq reads were quality trimmed using Trimmomatic (Bolger et al., 2014) with the parameters “LEADING:30 TRAILING:30” prior to assembly.

For Oxford Nanopore long-read sequencing, 1 μg of genomic DNA was sheared in a gTube (Covaris, Woburn, MA, United States) for 1 min at 6,000 r/min in a MiniSpin plus microfuge (Eppendorf, Hauppauge, NY, United States). The sheared DNA was converted to a shotgun library with the LSK-108 kit from Oxford Nanopore, following the manufacturer’s instructions. The library was sequenced on a SpotON R9.4 flowcell for 48 h using a MinION MK 1B sequencer.

Basecalling and demultiplexing were performed in real time with the Metrichor Agent V2.45.3 using the 1D BaseCalling plus Barcoding for FLO-MIN_106_450bp workflow. Removed from both ends of each Oxford Nanopore read were 60 nt, followed by additional trimming using a Github checkout (commit 92c0b65f) of Porechop (Wick et al., 2017) to remove reads with potential internal barcodes which were likely chimeric. Only reads longer than 800 nt were used in the final assembly. Canu v1.4 (Koren et al., 2017) was used for assembly with the following parameters: “canu -p asm -d C_trop_default genomeSize = 14m useGrid = false -nanopore-raw c_tropicalis.qualtrim.clean.fastq.gz.”

Oxford Nanopore reads were then aligned against the assembly using bwa mem (Li, 2018) with parameters “bwa mem -x ont2d C_tropicalisCanuAsm.fasta reads.fa”, and the alignment was then used to polish the assembly using nanopolish v 0.6.0 (Senol Cali et al., 2018). Quality-trimmed MiSeq data were used to polish the assembly using Pilon v1.21 for error correction (Walker et al., 2014). Supplementary File S1 includes details regarding the computational analyses and characteristics of the resulting *C. tropicalis* MYA-3404 genome sequence. The sequence was deposited in the NCBI database (ASM694213v1; GCA_006942135.1).

Ambiguities in the genome sequence data were resolved by PCR amplification of the region and Sanger sequencing of the product. Primer design was aided by the PrimerQuest Tool (Integrated DNA Technologies). Supplementary Table S1 lists PCR primer sequences that were used for amplification and/or Sanger sequencing for the various *ALS* loci.

During finalization of this manuscript, a new *C. tropicalis* MYA-3404 genome sequence was noted in the NCBI database (ASM1317755v1; GCA_013177555.1; Guin et al., 2020). The sequence was generated with a combination Illumina HiSeq and PacBio Sequel technology and had the same number of contigs as *C. tropicalis* has chromosomes. This sequence was used for comparative analysis of *ALS* sequences derived from ASM694213v1.

### Identification of *ALS* Genes and Predicted Als Protein Features

Methods for identifying *C. tropicalis ALS* genes and deducing predicted protein features were identical to those reported for analysis of *C. metapsilosis* (Oh et al., 2019). Details were reproduced here for the reader’s convenience. BLAST^2^ was used to identify potential *ALS* genes and Als proteins in the genome sequences. Query sequences included all *C. albicans ALS* genes as reported by Oh et al. (2019). As new *ALS*/Als sequences were identified, they were also used as BLAST queries until search reports failed to reveal new sequences. SignalP Server^3^ (Nielsen, 2017) was used to locate putative secretory signal peptides. The big-PI Predictor^4^ (Eisenhaber et al., 1999) identified potential GPI anchor addition sites. European Bioinformatics Institute (EMBL-EBI) tools were used for translating nucleotide sequences, sequence alignment, and other general processes^5^ (Cook et al., 2017).

### Phylogenetics Analysis

Phylogeny of the *ALS* family was estimated using sequences from the 5′ domain that is present in each gene (see Supplementary File S2). Because of the large sequence divergences within the 5′ domain, nucleotide sequences were translated using the alternative yeast nuclear code and the resulting amino acid sequences aligned with PROMALS3D (Pei et al., 2008). Poorly aligned regions were eliminated using Gblocks v 0.91b with default settings (Castresana, 2000). There were 227 positions in the final alignment. Model selection was performed using ModelFinder (Kalyaanamoorthy et al., 2017) implemented in IQ-TREE (Nguyen et al., 2015); LG+I+G4 was chosen as a best-fit model according to the Bayesian information criterion. The maximum likelihood tree was constructed with IQ-TREE v. 1.6.12 with nodal support determined by non-parametric bootstrapping with 500 replicates. Bayesian posterior probabilities were calculated using MrBayes 3.2.6 (Ronquist et al., 2012). The analysis ran for 3 × 10^6^ generations. Two parallel runs were used with four chains each, sample frequency of 100 generations, and 25% burn-in.

### Fungal Growth Conditions for Gene Expression Analysis

*C. tropicalis*, *C. albicans*, and *Candida dubliniensis* isolates were grown using the same methods except where noted below. All fungal isolates were stored as 30% glycerol stocks at −80°C and streaked to a YPD plate prior to use in an experiment. YPD stock plates were incubated for approximately 24 h at 37°C and then kept at 4°C for no more than 1 week. A starter culture was prepared by inoculating one representative colony from the YPD plate into 20 ml of liquid YPD in a 50-ml flask. The flask was incubated at 30°C and 200 r/min shaking for 16 h. All cells were collected by centrifugation and washed twice in sterile MilliQ water. Cell number was calculated using a hemacytometer. A small portion of the 16 h culture was flash frozen in dry ice/ethanol and duplicate samples stored at −80°C for RNA extraction to measure relative gene expression in a saturated culture.

Growth conditions were selected from previous analyses of *ALS* gene expression and Als protein production in cultured *C. albicans* cells. Growth in RPMI 1640 medium (Gibco; 11875-135) induces *C. albicans* germ tube formation and its associated differential Als protein production (Coleman et al., 2009). For growth in RPMI 1640, washed cells from the starter culture were used to inoculate 20 ml of the medium at a density of 5 × 10^7^ cells/ml in a 50-ml flask. Flasks were incubated at 37°C and 200 r/min shaking for 1 h. The culture was divided into two equal portions, and cells were collected by filtration over a sterile 0.45-μm pore-size membrane (GVS Life Sciences; 1213776). Filters were flash frozen in dry ice/ethanol and stored at −80°C.

Analysis of *ALS* gene expression in cells from an early-growth-stage culture used YPD (rich) medium. Two identical 250-ml flasks were filled with 100 ml of YPD and inoculated at a cell density of 1 × 10^6^ per ml. Flasks were incubated for 1 h at either 37°C (*C. tropicalis*) or 30°C (*C. albicans* and *C. dubliniensis*) and 200 r/min shaking. Cells were collected by filtration as detailed above and filters flash frozen and stored at −80°C for RNA extraction.

Additional growth conditions for *C. tropicalis* were intended to examine *ALS* gene expression during morphological change. Lackey et al. (2013) described growth in synthetic defined medium [SD; 6.7 g/l yeast nitrogen base without amino acids (BD Biosciences)] supplemented with 50% fetal bovine serum (FBS) for this purpose. Early (2 h) and late (24 h) time points were evaluated to assess differential gene expression over the course of a growth curve. Washed cells from the 16-h YPD starter culture were resuspended in 15 ml of SD medium at an OD_600_ of approximately 1.7. Ten milliliters of the SD culture were added to 10 ml of 100% FBS and incubated at 30°C and 200 r/min shaking for 2 h. The culture was divided in half, cells collected by centrifugation, flash frozen in dry ice/ethanol, and stored at −80°C. The remaining 5 ml of the 15 ml SD starter culture was combined with 5 ml of 100% FBS and incubated for 24 h at 30°C and 200 r/min shaking. Cells were collected and stored as described above.

Cultures were prepared on three different days. Each experimental day had daily replication by creating two unique cultures from two different colonies on the original agar plate.

### TaqMan Assays for Analysis of *ALS* Gene Expression

TaqMan assays were designed to specifically detect each of the *C. tropicalis ALS* genes, as well as to differentiate between alleles of *CtrALS3882*. To place *C. tropicalis* gene expression data into a larger context, TaqMan assays were also designed and validated for each *ALS* gene in *C. albicans*. TaqMan assays were also designed for *C. dubliniensis ALS* genes because the literature lacked information about *ALS* gene expression in this species. Methods for designing and validating TaqMan assays were detailed by Oh et al. (2019). Although an amplicon size of 140 bp was targeted, some assays required a shorter or longer product to ensure specificity of detection. Amplicon sizes ranged from 106 to 213 bp; PCR efficiencies ranged from 95 to 102% (Supplementary Table S2).

TaqMan assay specificity was validated carefully using cloned control gene fragments (DNA templates; Supplementary Table S3). Detailed methods and examples of acceptable results were described previously (Oh et al., 2019). TaqMan control reactions targeted *ACT1* and *TEF1* using primer/probe sets capable of recognizing all species in the study (Supplementary Table S2).

Methods for RNA extraction, genomic DNA removal, cDNA synthesis, and TaqMan assays were described previously (Oh et al., 2019). Experiments in this report were completed entirely on a QuantStudio 3 Real-Time PCR System. Statistical significance was assessed using a mixed-model analysis of variance (PROC MIXED in SAS 9.4; SAS Institute Inc., Cary, NC, United States). LSMEANS was used for separation of means.

## Results

### Generation of a Novel Genome Sequence for *C. tropicalis* MYA-3404

The major goal of this work was to define the *ALS* gene family in *C. tropicalis*. At the time the project began, the best genome assembly available was accession ASM633v3, initially deposited in 2005. The dataset had 128 contigs, assembled into 24 scaffolds that, in places, were spliced together by strings of “NNN” to indicate sequence ambiguity. Putative *ALS* loci were located using BLAST. The fragmented loci were documented in Supplementary Figure S1 and the predicted partial proteins in Supplementary Figure S2.

Long-read sequencing using the Oxford Nanopore MinION technology was just emerging at the start of this project in 2017. The potential for long-read sequencing to span sometimes-lengthy repeated regions within *ALS* open reading frames led to the development of a new genome assembly that used Illumina MiSeq and Oxford Nanopore MinION data (ASM694213v1; GCA_006942135.1). The accurate Illumina data were included to correct the MinION data, which were already recognized as error prone (de Lannoy et al., 2017). The assembly included 29 contigs and no Ns (Supplementary File S1). Although the long-read data provided an improved assembly compared to ASM633v3, completion of the *ALS* loci still required additional considerable effort.

**FIGURE 1:**
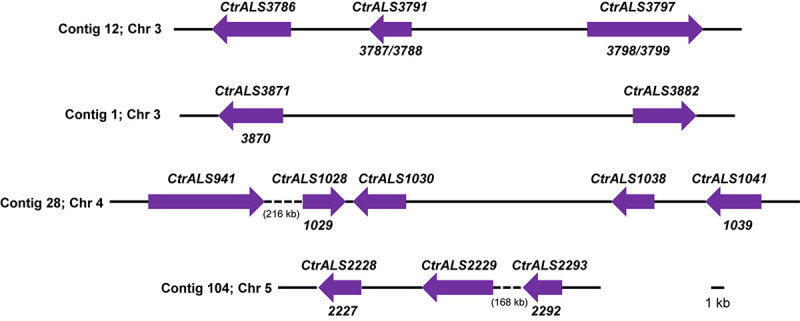
Schematic of *ALS* loci in *Candida tropicalis* MYA-3404 as deduced using assembly ASM694213v1, PCR amplification, and Sanger sequencing. Contig numbers from assembly ASM694213v1 were noted at the left of each diagram; chromosome assignments included in assembly ASM1317755v1 (Guin et al., 2020) were also noted. Arrows were drawn to scale and represent the size of one allele at each *ALS* locus. Because *ALS* sequences were finalized using PCR amplification for Sanger sequencing, the alleles shown here were likely biased toward the smaller allele when one existed. Final gene names were indicated above each locus; ASM633v3 open reading frames (ORFs) that were combined into the final gene were designated below. Intergenic distances were drawn to scale except where quite large and then indicated by a dashed line and approximate length (in kb). Sequences were deposited in GenBank and accession numbers listed in Table 2. In some cases where the number of copies of the tandem repeat sequence was exceptionally large, unique primers for PCR amplification and Sanger sequencing could not be designed. Instead, the reported sequence was the consensus from the 105× coverage of the Illumina MiSeq/Oxford Nanopore MinION data.

**FIGURE 2:**
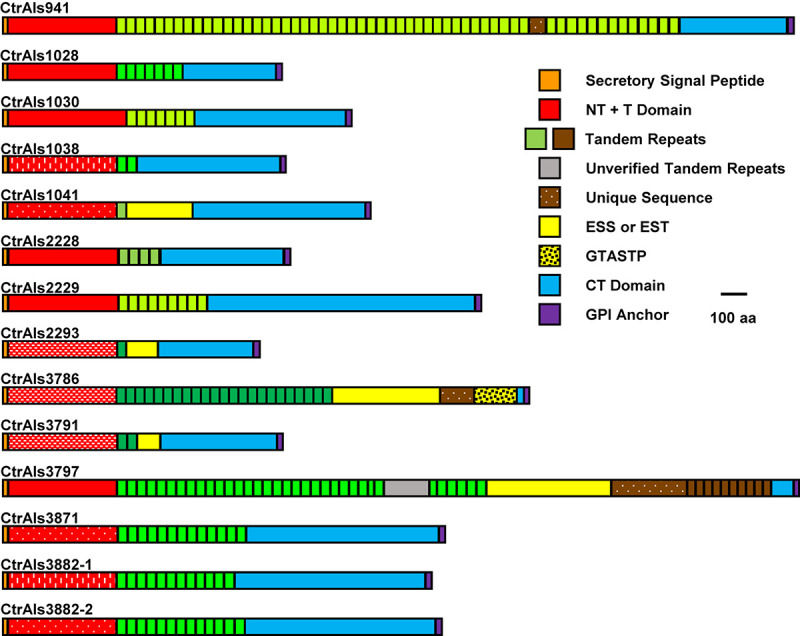
Schematic of predicted *C. tropicalis* MYA-3404 Als proteins drawn to scale. Protein sequences showed common Als features (reviewed in Hoyer and Cota, 2016) including a secretory signal sequence, NT + T domain, copies of tandemly repeated sequences, and a C-terminal (CT) domain that was rich in Ser/Thr. *C. tropicalis* Als proteins had some novel repeated sequences such as those rich in ESS or EST, or the GTASTP motif. Protein features were color coded to recognize similarity across the family. Red blocks that represent NT + T domains were further modified to denote similarity between sequences (e.g., speckles or dashed lines as reflected by percent identity values in Figure 3). Blocks denoting tandemly repeated sequences were in different shades of green to indicate sequences with greater similarity to each other. CtrAls2293 was drawn to include a predicted GPI anchor domain, although the putative signal is much weaker in this sequence than others in the family. Extremely long tandem-repeat domains (e.g., *CtrALS941* and *CtrALS3797*) could not be verified by PCR amplification and Sanger sequencing because highly conserved repeated units did not permit the design of unique primers. The reported sequence relied on the 105× coverage from assembly ASM694213v1, which for *CtrALS3797* required insertion of “XXX” (gray color) to make the size of the gene match the fragment sizes generated by PCR.

The overall strategy for accurate assembly of the *C. tropicalis ALS* family involved identifying unique genomic landmarks to anchor specific *ALS* loci in their proper physical location. PCR primers were designed to amplify regions that required improvement. Sanger sequencing was used to generate the final data. Figures 1, 2 display the assembled *ALS* loci and predicted proteins, respectively. Table 2 lists the assembled *ALS* genes and places them into the context of the original MYA-3404 genome assembly (ASM633v3) as well as previous publications that mentioned the *C. tropicalis ALS* family.

**TABLE 2:** *C. tropicalis* MYA-3404 *ALS* genes from ASM694213v1 and their corresponding open reading frames (ORFs) in previously published work.

Gene Name	Size (bp)	GenBank Accession Number	NCBI ORFs Included in Final ORF*	*ALS* ORFs from Butler et al. (2009)	*ALS* ORFs from Jackson et al. (2009)
*CtrALS941*	8,865	MH753531	CTRG_00941	00941	00941.3
*CtrALS1028*	3,192	MH753521	CTRG_01028^b,c^, CTRG_01029^a^	01028	1028.3
*CtrALS1030*^†^	3,963	MH753522	CTRG_01030	01030	1030.3
*CtrALS1038*	3,204	MK128125	CTRG_01038^b,c^	01038	1038.3
*CtrALS1041*	4,200	MK128127	CTRG_01041^b,c^, CTRG_01039^c^	01041	1041.3
*CtrALS2228*^‡^	3,255	MT863732	CTRG_02228^b,c^, CTRG_02227^a,c^	02228	02228.3
*CtrALS2229*	5,406	MH753523	CTRG_02229^a^	02229	02229.3
*CtrALS2293*^†^	2,925	MK182724	CTRG_02293^b^, CTRG_02292^c^	02293	02293.3
*CtrALS3786*	6,030	MK332912	CTRG_03786^b,c^	03786	03786.3
	N/A	N/A	Incorporated into *CtrALS3791*	03787	
*CtrALS3791*	3,171	MK170233	CTRG_03791^c^, CTRG_03788^a,c^, CTRG_03787^a,c^	03791	03791.3
*CtrALS3797*^†^	8,850	MN224675	CTRG_03797^b^, CTRG_03798^a^, CTRG_03799^c^	03797	03797.3
*CtrALS3871*	4,968	MH753524	CTRG_03871^b,c^	03871	03871.3
*CtrALS3882-1*	4,833	MH753525	CTRG_03882	03882	03882.3
*CtrALS3882-2*	4,440	MN893367	N/A	N/A	N/A

*Superscripts in the NCBI ORFs column denote defects in the ASM633v3 assembly of *ALS* genes. a = lack of a secretory signal peptide. b = lack of a GPI anchor addition signal. c = lack of sequence features expected for the *ALS* family or not recognized as a potential *ALS* gene fragment in the original annotation. ^†^Partial *ALS* sequences were deduced from PCR amplification of *C. tropicalis* genomic DNA using consensus primers developed from alignment of *C. albicans ALS* sequences (Hoyer et al., 2001). These gene fragments can now be assigned to a complete ORF. In the original manuscript, the gene fragments were named *ALST1* (GenBank accession AF201686.1; now recognized as *CtrALS3797*), *ALST2* (AF211865.1; *CtrALS1030*), and *ALST3* (AF211866.1; *CtrALS2293*). ^‡^The larger allele of *CtrALS2228* was also deposited into GenBank (accession number MK128126; 4,872 bp). The larger allele encoded more copies of the tandemly repeated sequence than the smaller allele and, in assembly ASM694213v1, included frameshifts within the tandem repeat domain.

In May 2020, a new *C. tropicalis* MYA-3404 genome assembly became available in the NCBI database (ASM1317755v1; GCA_01317755.1; Guin et al., 2020). This assembly was generated using PacBio Sequel and Illumina HiSeq data. It was notable because the number of contigs equaled the number of *C. tropicalis* chromosomes, signaling the highest-quality assembly available to date. A comparison between our carefully curated *ALS* genes and the new assembly is presented in Table 3. Overall, the new Sequel assembly had the correct number of *ALS* genes, placed in the proper physical locations. Some of the Sequel *ALS* ORFs were identical in length to our hand-curated data, although many were longer. Nucleotide polymorphisms existed between our gene sequences and those in the Sequel assembly. For three genes, these polymorphisms resulted in frameshifts and/or premature stop codons, thus an incomplete *ALS* ORF in the Sequel data.

**TABLE 3:** Comparison between *ALS* sequences in *C. tropicalis* MYA-3404 genome assemblies ASM694213v1 (MiSeq/MinION) and ASM1317755v1 (HiSeq/Sequel).

	MinION	Sequel	Identity
Gene	(aa)	(aa)*	(%)^†^
*CtrALS941*	2,954	2,954	99.1
*CtrALS1028*	1,063	1,063	97.0
*CtrALS1030*	1,320	**1,356**	99.7
*CtrALS1038*	1,069	**1,356**	98.5
*CtrALS1041*	1,399	**2,803**	99.6
*CtrALS2228*	1,084	**1,696**	99.7
*CtrALS2229*	1,801	1,801	100
*CtrALS2293*	974	**1,639**	96.9
*CtrALS3786*	2,009	**2,469**^‡^	—^§^
*CtrALS3791*	1,056	**2,264**^‡^	—^§^
*CtrALS3797*	2,949	**3,109**^‡^	—^§^
*CtrALS3871*	1,655	**2,015**	99.6
*CtrALS3882-1*	1,610	1,610	99.5
*CtrALS3882-2*	1,479	1,610	82.8

*Bold type indicates predicted protein lengths that were larger than the sequences from MinION/MiSeq/Sanger sequencing. Smaller size for the MinION alleles was likely due to PCR amplification of target regions which biases for smaller fragments. ^†^Percent identity was calculated using Clustal Omega alignment of the sequences (Cook et al., 2017). ^‡^Predicted size was estimated by reading past the broken gene sequence and locating a putative GPI anchor signal and stop codon. ^§^Percent identity could not be calculated because genes in the Sequel assembly were incomplete. In the Sequel assembly, *CtrALS3786* had two frameshifts, then two premature stop codons. *CtrALS3791* had a frameshift. *CtrALS3797* had two premature stop codons.

### The *C. tropicalis ALS*/Als Family

The 13 distinct physical loci in the *C. tropicalis ALS* family (Figure 1) predicted proteins (Figure 2) with Als features including a secretory signal peptide and a GPI anchor addition site that target the mature protein toward linkage to β-1,6-glucan in the fungal cell wall (Lu et al., 1994). Each predicted protein had a domain structure characteristic of Als proteins (Hoyer and Cota, 2016) including an NT-Als adhesive domain (Lin et al., 2014), followed by a Thr-rich (T) sequence and at least one copy of a sequence that resembled an Als tandem repeat. Like previously characterized Als sequences, those in *C. tropicalis* were increasingly rich in Ser and Thr residues toward the C-terminal end (reviewed in Hoyer et al., 2008; Lombardi et al., 2019; Oh et al., 2019).

**FIGURE 3:**
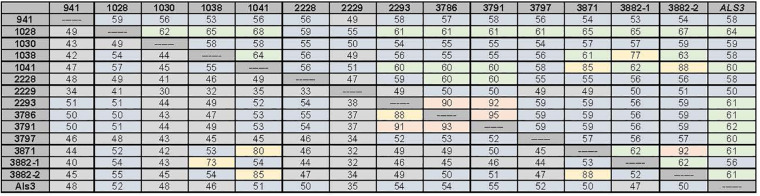
Percent identity values between the nucleotide sequences for the 5′ domain of the *C. tropicalis ALS* genes (upper diagonal) and their predicted amino acid sequences (lower diagonal). The region used for comparisons corresponds to the NT-Als domain of each protein. *C. albicans* Als3 (GenBank AY223552.1) was included for comparison. Boxes were shaded to indicate overall percent identity with hotter colors (red, yellow) used for higher percent identity than cooler colors (green, blue, and gray). Both alleles of *CtrALS3882* were included in the diagram to highlight lack of identity to each other, but high sequence conservation with other loci.

**FIGURE 4:**
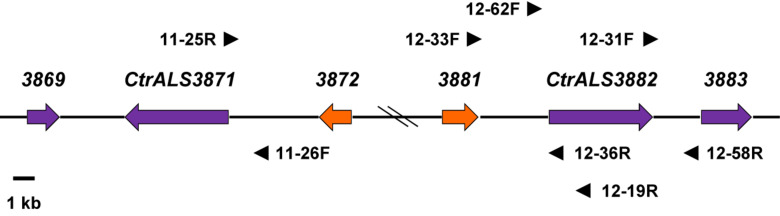
Schematic of the *CtrALS3882* locus to support the conclusion that *CtrALS3882-1* and *CtrALS3882-2* occupied the same relative physical location on the diploid chromosomes of *C. tropicalis* MYA-3404. Hash marks in the diagram indicated an approximate distance; an accurate distance was shown in Figure 1. *CtrALS3882-1* and *CtrALS3882-2* were only 52% identical in the 5′ domain of the ORF (Figure 3). ORFs were represented by arrows that indicate the orientation of each gene. Orange arrows indicate *CTRG_03872* and *CTRG_03881*, which were 97% identical. Primers used to demonstrate the physical location of each ORF are shown; primer sequences are recorded in Supplementary Table S1 to indicate whether they recognized both alleles or were specific for *CtrALS3882-1* or *CtrALS3882-2*.

Closer examination of these features suggested that the predicted NT-Als adhesive domain of each *C. tropicalis* Als protein was only approximately 50% identical to *C. albicans* NT-Als3 for which the molecular structure is known (Lin et al., 2014; Figure 3). Percent sequence identity among the predicted NT-Als domains in *C. tropicalis* was also in the 30–50% range for most comparisons, even between proteins encoded by the *CtrALS3882* alleles, which were only 52% identical (Figure 4). A high degree of sequence conservation was noted among some NT-Als domains including CtrAls2293/CtrAls3786/CtrAls3791, which were 88–93% identical (Figure 3). CtrAls3882-2 NT-Als was 88% identical to CtrAls3871, while CtrAls3882-1 NT-Als more closely resembled CtrAls1038 (73%). CtrAls1041 NT-Als was 80% identical to the same region in CtrAls3871. Nearly all NT-Als domains predicted from DNA sequence data encoded eight conserved Cys residues that direct folding of the adhesive domain (Lin et al., 2014); CtrAls1030 had only six Cys residues and CtrAls3871 had 10.

The divergent sequences in the 5′ domain of *CtrALS3882-1* and *CtrALS3882-2* complicated the assembly and definition of the *C. tropicalis ALS* family. The presence of divergent sequences was first noted during attempts to amplify the 5′ domain of *CtrALS3882* for Sanger sequencing. Resulting chromatograms yielded overlapping peaks that suggested a diploid sequence (data not shown). Subsequent efforts focused on designing PCR primers that anchored *CtrALS3882* to an exact physical location, then demonstrating that both alleles occupied the same location, presumably on sister chromosomes (Figure 4). The effort was made more difficult by additional sequence conservation in the region, including the closely related genes *CTRG_03872* and *CTRG_03881*, which were 97% identical. DNA fragments were amplified, cloned, and Sanger sequenced to reveal unique areas to which additional primers could be designed. For example, primers 12-62F and 12-58R were used with Q5 polymerase (New England Biolabs) to specifically amplify *CtrALS3882-2*. The physical location of each allele was validated by amplifying upstream and downstream fragments for Sanger sequence analysis. Examples include the use of primers 12-33F/12-36R and 12-31F/12-58R that amplified the regions upstream and downstream of *CtrALS3882-2*, respectively, amplification of the region upstream of *CtrALS3882-1* with primers 12-33F/12-19R, and amplification of the region upstream of *CtrALS3871* with primers 11-26F/11-25R. Sequence polymorphisms unique to each physical location were deduced by comparison among the PCR products and available genome assemblies. Each *CtrALS3882* allele was detected in all six *C. tropicalis* isolates studied suggesting that heterozygosity at this locus is reasonably common within the species. DNA sequences for each allele in the various isolates are included in Supplementary File S3.

**FIGURE 5:**
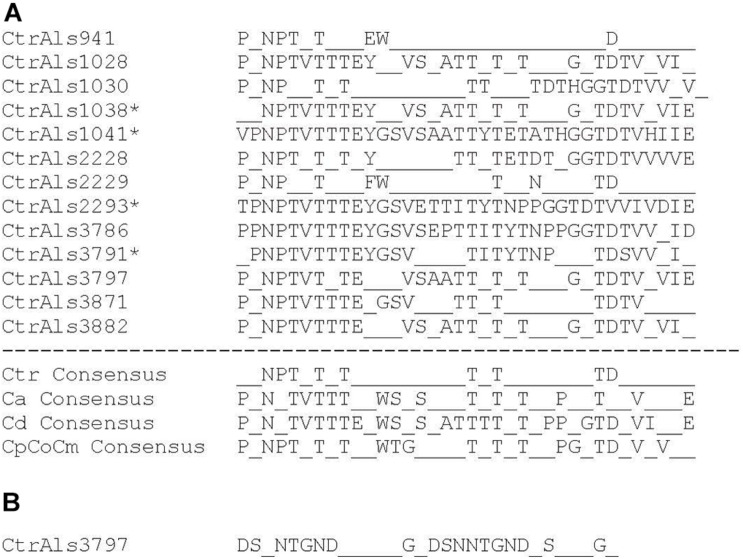
Analysis of tandem repeat consensus sequences to assess similarity among repeat units in Als proteins from various fungal species. Most repeat units were 36 amino acids, although exceptions were noted. Consensus sequences required that 80% or more of the amino acids were identical at each position. **(A)** Consensus sequences derived from protein translation of *C. tropicalis ALS* sequences identified in Table 2. The repeat unit in CtrAls1030 was 37 amino acids. Asterisks marked sequences for which only one to two tandem repeat copies were present. Lack of tandem repeat copies in these proteins provided the potentially false impression of a high level of sequence conservation. *ALS* tandem repeat consensus sequences for *C. albicans* (Ca; Oh et al., 2019), *C. dubliniensis* (Cd), and the *C. parapsilosis* species complex (CpCoCm; Oh et al., 2019) were included for comparison. **(B)** Consensus sequence for the novel tandem repeat from the C-terminal domain of CtrAls3797 (see Figure 2).

Tandem repeat copy number in some *C. tropicalis* MYA-3404 *ALS* alleles was large (e.g., *CtrALS941* and *CtrALS3797*) and in others was limited to a single unit (*CtrALS1041* and *CtrALS2293*). The consensus sequence derived from the main, central domain of tandem repeats was similar to those found in previously characterized Als proteins (Figure 5A). Repeat unit length generally was 36 amino acids, although CtrAls1030 contained multiple copies of a 37-amino-acid repeated sequence, and other alleles contained irregular repeat lengths (i.e., individual units of 35 or 40 amino acids). Novel repeat sequences were present such as short motifs (ESS, EST, GTASTP) or a 30-amino acid tandem motif in CtrAls3797 (Figure 5B).

**FIGURE 6:**
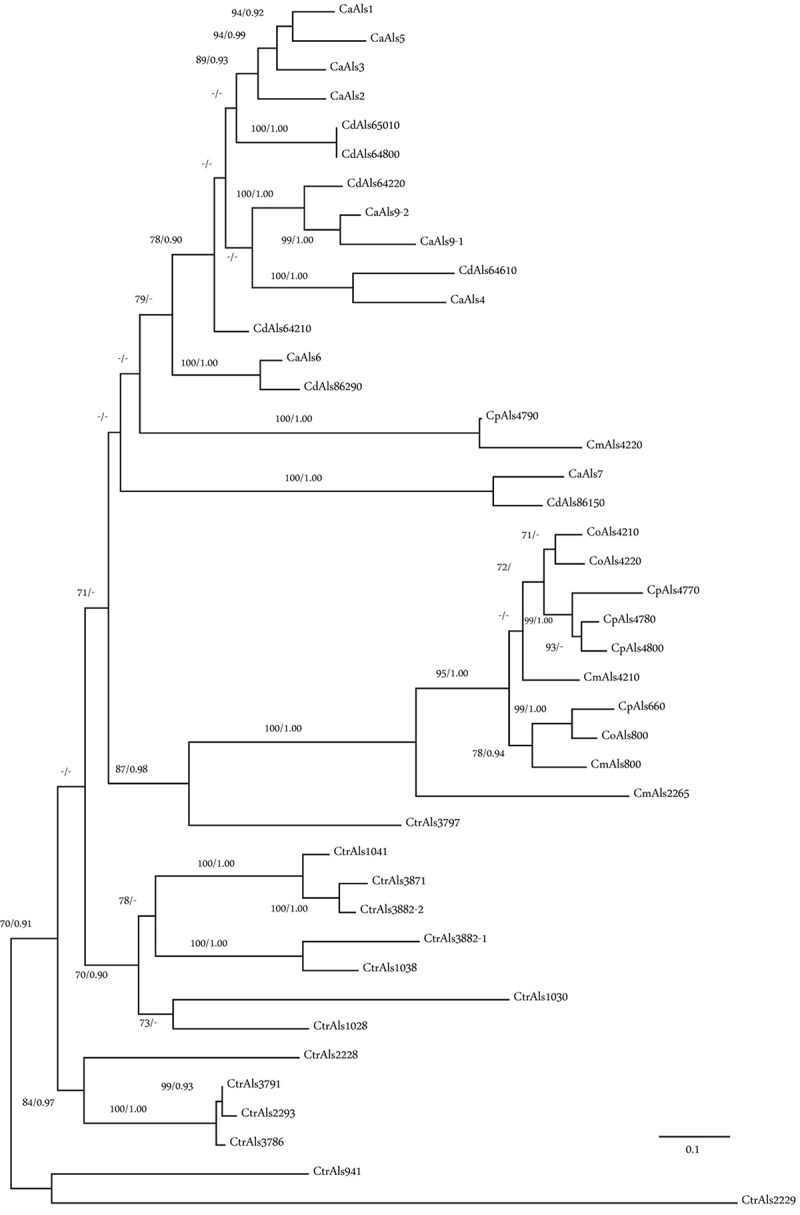
Phylogeny of Als N-terminal domain sequences from *C. albicans* (Ca), *C. dubliniensis* (Cd), *C. tropicalis* (Ctr), *C. parapsilosis* (Cp), *C. orthopsilosis* (Co), and *C. metapsilosis* (Cm). The best-scoring Maximum likelihood tree is shown with maximum likelihood bootstrap values and Bayesian posterior probabilities at each node; only support values greater than 70% and 0.90, respectively, were shown. Branch lengths were proportional to evolutionary change and measured in substitutions per site.

Phylogenetic analysis was conducted using the amino acid sequences from the NT-Als adhesive (functional) domain from each Als protein in *C. albicans* (Ca), *C. dubliniensis* (Cd), *C. tropicalis* (Ctr), *Candida parapsilosis* (Cp), *Candida orthopsilosis* (Co), and *C. metapsilosis* (Cm; Figure 6). Sequences beyond this region could not be included because of their high level of divergence. Nucleotide sequences encoding NT-Als were also too divergent, so amino acid sequences were aligned and variable regions extracted as described in Section “Materials and Methods.” Results showed three phylogenetically distant groups: Ca/Cd, Cp/Co/Cm, and Ctr. Orthologs were apparent in the Ca/Cd group (e.g., CaAls9/CdAls64220, CaAls4/CdAls64610, CaAls6/CdAls86290, and CaAls7/CdAls86150). A previous publication (Jackson et al., 2009) offered evidence to support the orthology of CaAls1/CdAls64210. Within the Cp/Co/Cm group, orthologs CpAls660/CoAls800/CmAls800 were evident. Paralogous sequences, most likely resulting from duplication events and generating contiguous genes within a species included CoAls4210/CoAls4220 and CpAls4770/CpAls4780/CpAls4800. The NT-Als sequences from *C. tropicalis* were relatively distant phylogenetically. The larger number of *ALS* genes in *C. tropicalis* compared to the other species likely arose from gene duplication. Potential paralogs included CtrAls1041/CtrAls3871/CtrAls3882-2, CtrAls1038/CtrAls3882-1, and CtrAls3791/CtrAls2293/CtrALS3786. CtrAls3797 grouped more closely to the Cp/Co/Cm sequences. This trend was also apparent for CpAls4790/CmAls4220 that grouped more closely with the Ca/Cd sequences suggesting a potential ortholog within that group. Functional information for each protein would aid interpretation of these distinctions.

### Relative Expression Levels of *Candida tropicalis ALS* Genes

Real-time RT-PCR analysis of *ALS* gene expression was pursued to determine if the family was differentially expressed by growth condition and cellular morphology. High gene expression levels might also predict which Als proteins were present in the greatest abundance on the *C. tropicalis* cell surface, thereby positioned to contribute most to the adhesive function. A unique TaqMan assay was designed for each *ALS* gene and to differentiate between the alleles of *CtrALS3882*. Assay primers and probe sequences were placed at a similar location within the 5′ domain for each gene. *C. tropicalis* MYA-3404 cells were harvested from five different culture conditions, RNA extracted, and cDNA synthesized for TaqMan analysis.

**FIGURE 7:**
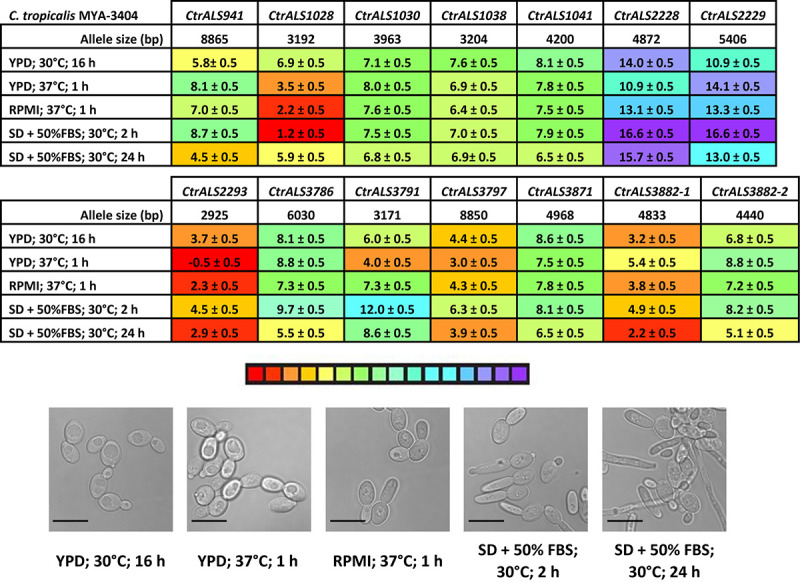
Relative expression of *C. tropicalis ALS* genes as measured by TaqMan assays. Expression of the 13 *C. tropicalis ALS* genes was measured for strain MYA-3404 grown in five different culture conditions. Micrographs of representative cells from each culture condition were captured to show cellular morphology that corresponded to the gene expression data (scale bar = 10 μm). ΔC_*t*_ values and standard errors of the mean were reported. The color bar below the data tables was shaded to represent high (red) to low (purple) expression levels.

Color coding in Figure 7 denotes high (red) and low (purple) expression levels based on the threshold (ΔC_*t*_) values relative to the *ACT1* and *TEF1* control genes. With C_*t*_ values that lagged only one to two cycles behind the control gene expression, *CtrALS1028* showed high expression levels in RPMI medium and from a 2-h culture of SD + FBS. These expression levels were not significantly different from each other (*p* = 0.1276). Expression levels were lower in cells from a 16-h YPD culture and the 24-h SD + FBS condition; these values were not significantly different from each other either (*p* = 0.1245). All other comparisons were significantly different suggesting differential expression of *CtrALS1028* by stage of culture growth (1 vs. 16 h in YPD; *p* < 0.0001; 2 h vs. 24 h in SD + FBS; *p* < 0.0001) with higher expression levels in cells that were more recently transferred to fresh growth medium. In contrast, *CtrALS941* expression was lower at early stages of culture growth and significantly higher as the culture reached saturation (16 h vs. 1 h in YPD; *p* = 0.0007; 24 vs. 2 h in SD + FBS; *p* < 0.0001). *CtrALS3791* showed yet another type of differential expression pattern with its highest expression in the 1 h YPD culture and significantly lower values in the early-stage growth in other media (*p* < 0.0001). Many genes had an expression pattern like *CtrALS1030*, expressed at only moderate levels in all five growth conditions. For *CtrALS1030*, none of the statistical comparisons were significant (*p* > 0.05) suggesting lack of differential expression for the growth conditions tested.

**FIGURE 8:**
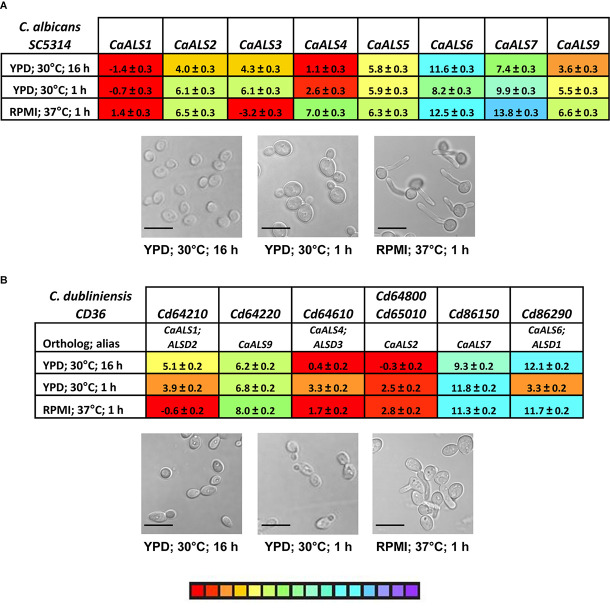
TaqMan measurement of *ALS* gene expression in *C. albicans* **(A)** and *C. dubliniensis* **(B)**. *C. albicans* SC5314 and *C. dubliniensis* CD36 were grown in various culture conditions known to display differential expression of *C. albicans ALS* genes. Images were captured for representative cells from each culture (scale bar = 10 μm). ΔC_*t*_ values and standard errors of the mean were reported. The color-coded scale bar ranged from high expression (red) to low (purple). Two *C. dubliniensis ALS* loci had identical sequences and were detected by the same TaqMan assay (*CdALS64800* and *CdALS65010*). *C. dubliniensis ALS* genes were orthologous to *C. albicans ALS* loci as designated in **(B)**. Prior to availability of a *C. dubliniensis* genome sequence, *ALS* genes were identified using consensus PCR primers derived from *C. albicans ALS* sequences (Hoyer et al., 2001). These were designated *ALSD1* (now recognized as *CdALS86290*), *ALSD2* (*CdALS64210*), and *ALSD3* (*CdALS64610*).

The examples above provided a context for statistical significance for differences between the gene expression values. Coupled with the color coding in Figure 7, many other comparisons can be made visually and quickly evaluated for their potential statistical significance. However, the data did not provide a biological context for the relationship between TaqMan ΔC_*t*_ values and detectable Als protein on the *C. tropicalis* cell surface. A potential context was provided by designing TaqMan assays for *C. albicans ALS* genes (Supplementary Table S2) and using them to assess gene expression in growth conditions for which the association between differential gene expression and protein abundance on the fungal cell surface was known. The rationale for this approach was that use of monoclonal antibodies specific for individual Als proteins demonstrated that CaAls1, CaAls3, and CaAls4 can be visualized readily by immunofluorescent labeling on the *C. albicans* surface, while CaAls5 and CaAls6 cannot (Coleman et al., 2009, 2010, 2012). CaAls5 could be detected in Western blots of *C. albicans* cell wall extracts, while the CaAls6 signal remained negative (Zhao et al., 2011). These previous observations corresponded well with the TaqMan ΔC_*t*_ values (Figure 8A) and suggested that a ΔC_*t*_ value of approximately 7 might serve as a potential cutoff between detectable and non-detectable cell-surface protein.

Revisiting Figure 7 with this insight suggested that although most comparisons between TaqMan ΔC_*t*_ values for *CtrALS2228* and *CtrALS2229* were statistically significant, neither gene was transcribed at a level that was likely to produce detectable protein on the *C. tropicalis* cell surface. In each growth condition assayed, several other genes were transcribed at a level that was also unlikely to produce detectable protein (i.e., *CtrALS1030*, *CtrALS1038*, *CtrALS1041*, and *CtrALS3871*). Some genes were differentially expressed at a sufficient level to produce detectable protein in specific growth conditions (e.g., *CtrALS3791* in 1 h YPD), while others appeared to produce the proteins that might have the greatest cell-surface presence. For example, *CtrALS2293* was transcribed highly in all growth conditions tested. In a 24-h SD + FBS culture, it was likely that CtrAls2293 and CtrAls3882-1 would dominate the cell surface. In each growth condition tested, *CtrALS3882-1* was more highly expressed than its allele *CtrALS3882-2* (*p* < 0.0001).

One larger goal of our work was to understand the cell-surface Als presence on various fungal species that cause candidiasis. In this regard, little attention has been given to the expression of *C. dubliniensis ALS* genes. *C. dubliniensis* and *C. albicans* are closely related species in which *ALS* genes occupy similar physical loci (Jackson et al., 2009). However, *C. dubliniensis* does not encode an *ALS3* ortholog, and sequences for two of the *C. dubliniensis ALS* loci (*Cd64800* and *Cd65010*) are 100% identical within the 5′ domain of the gene. TaqMan assays were designed and used to analyze *ALS* gene expression in cells from strain CD36 grown using the same conditions applied to *C. tropicalis* and *C. albicans*; a single assay detected transcription from *Cd64800* and *Cd65010* (Figure 8B). Some *C. dubliniensis ALS* genes were capable of high expression levels (e.g., *Cd64610*, *Cd64800/Cd65010*), while others were barely transcribed (*Cd86150*). Differential expression was observed among the growth conditions tested (*Cd64210*, *Cd86290*).

**FIGURE 9:**
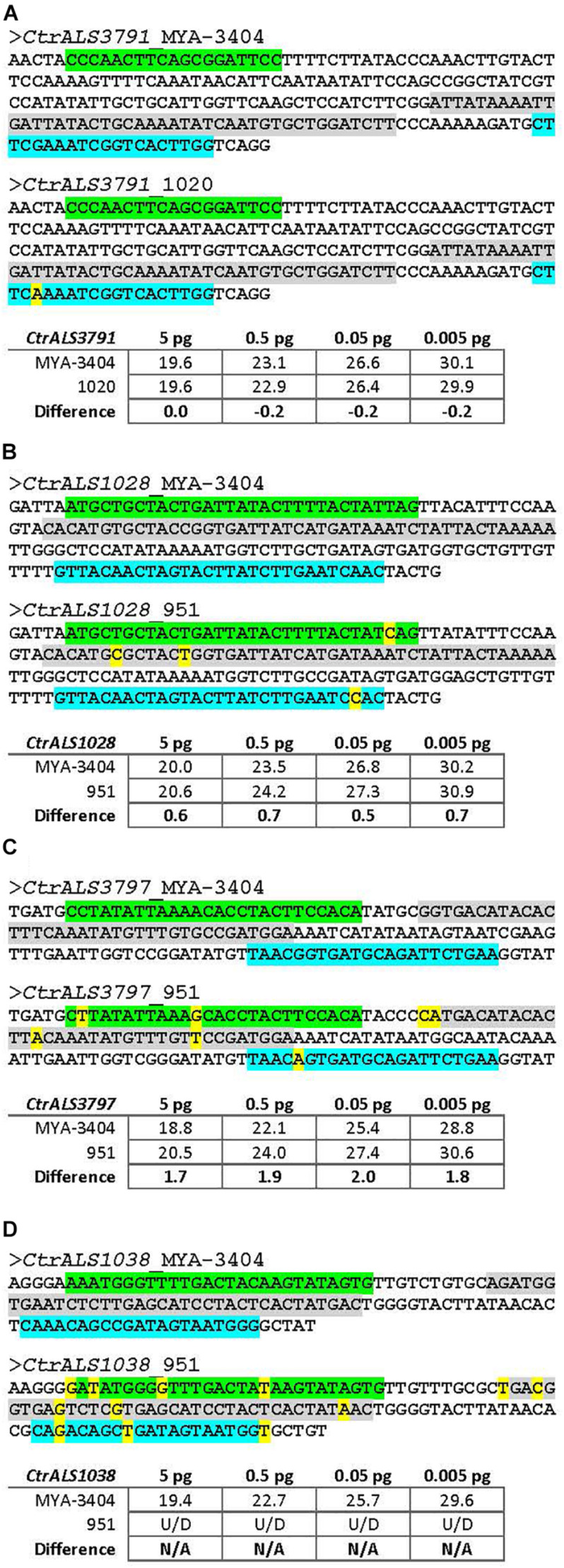
Effect of nucleotide sequence mismatch on TaqMan assay measurement of nucleic acid abundance. TaqMan assays were designed using gene sequences from *C. tropicalis* strain MYA-3404. Selection of unique target regions that distinguished among genes in the *ALS* family was the primary consideration in assay design. Sequencing of the 5′ domain of each *ALS* gene in six other *C. tropicalis* isolates revealed some mismatches in the TaqMan primer and/or probe sequences. Examples were selected to titrate the effect of an increasing number of mismatches, some in key primer/probe positions. Sequences in each panel were taken from Supplementary File S3. The forward primer was highlighted in green, reverse primer in blue, and probe in gray. Mismatches were noted with yellow color. Target sequences in each strain examined were PCR amplified, cloned, and Sanger sequence verified to ensure the mismatches were present. Cloned DNA was purified, diluted, and used in the assays reported here. **(A)** The sequence of the 5′ domain of *CtrALS3791* in strain 1020 showed a single nucleotide mismatch in the middle of the reverse primer. C_*t*_ values from TaqMan assays with 10-fold dilutions of cloned construct showed almost an equal ability for the assay to detect each strain (e.g., no difference for 5 pg DNA and negligible differences for subsequent dilutions). **(B)** *C. tropicalis* strain 951 showed one to two mismatches each in the primer and probe sequences for gene *CtrALS1028*. C_*t*_ values suggested that detection of strain 951 lagged approximately 0.6 cycle behind detection of strain MYA-3404, providing an underestimate of DNA abundance. **(C)** Increasing numbers of mismatches between primer and probe sequences resulted in greater underestimates of DNA abundance for strain 951, this time in *CtrALS3797* where nearly a two-cycle difference in C_*t*_ was observed. **(D)** Mismatches for *CtrALS1038* in strain 951 were so marked that the TaqMan assay was unable to detect genomic DNA, even at the highest concentration. These data provide the foundation necessary to adapt use of the TaqMan assays to *C. tropicalis* isolates in which primer and probe mismatches may exist.

The newly validated TaqMan assays were a tool that others may use to explore various hypotheses regarding the *C. tropicalis ALS* family. One potential experimental question was whether *ALS* expression patterns varied across *C. tropicalis* clinical isolates. DNA sequences were derived for the 5′ domain of each *ALS* gene in the six *C. tropicalis* isolates in our laboratory collection (Supplementary File S3). While many sequences showed a perfect match to TaqMan assay primers and probes, sequence variation was noted in some instances. These target sequences were cloned and verified; dilutions of purified DNA were used as the TaqMan assay template. Figure 9 shows an array of these examples, selected to titrate the degree of sequence mismatch that might result in underestimated or false-negative assay results. For example, one nucleotide mismatch in the middle of one primer was not sufficient to interfere with assay function (Figure 9A); however, accumulation of more mismatches, especially in key primer or probe positions led to falsely low estimates of transcript abundance (Figures 9B,C). Some mismatches between the TaqMan primers/probe and target sequences were so extensive that the assay was unable to detect the sequence (Figure 9D). Examination of the sequence data for the collection of six *C. tropicalis* isolates (Supplementary File S3) suggested that the TaqMan assays matched target sequences in nearly all instances. Strain 951 was the most divergent with three genes at risk of falsely low estimates of expression level (*CtrALS1038*, *CtrALS3797*, and *CtrALS3882-2*); sequence mismatches for *CtrALS3797* in three additional strains (1019, 1020, 3242) suggested the potential for redesign of that TaqMan assay depending on the strains and biological questions addressed.

## Discussion

Advances in DNA sequencing technology are providing long-sought information about the composition of the *ALS* family in fungal species. The availability of Oxford Nanopore MinION technology provided the initial opportunity to incorporate long-read DNA sequence data to resolve the *C. tropicalis ALS* genes. Despite the insights added by these long-read data, assembling accurate *ALS* sequences required considerable additional effort. The more recently released PacBio Sequel-based assembly (Guin et al., 2020) leverages technological improvements in DNA sequence accuracy that will continue to reduce the tedium associated with solving genomic puzzles that involve complex genes like those in the *ALS* family. The accurate list of *C. tropicalis ALS* loci provides an essential foundation for subsequent investigations into adhesion and pathogenesis of the species.

The *C. tropicalis* gene list (Table 2) also provides a standard set of names that can be used to derive and communicate unambiguous experimental results. Two published studies attempted to characterize *C. tropicalis ALS* gene expression without the benefit of a complete gene list (Yu et al., 2016; Galán-Ladero et al., 2019). Supplementary Table S4 shows that some of the PCR primers used in these studies are likely to amplify more than one *C. tropicalis ALS* gene, potentially complicating interpretation of results.

*ALS* gene names across publications can also be confusing. This confusion seems to originate from misinterpretation of Supplementary Table 23 from Butler et al. (2009). The Als family portion of the table is reproduced in Supplementary Table S5. Protein names in the left column match the entries in the *C. albicans* column but do not necessarily correspond to protein names in the other species. For example, the Candida Gene Order Browser^6^ (Fitzpatrick et al., 2010; Maguire et al., 2013) indicates that there is no positional ortholog for *C. albicans ALS1* (orf19.5714) in *C. tropicalis*, so *C. tropicalis* CTRG_02293 is not “*C. tropicalis ALS1*” and is definitely not *ALST1* as described by Hoyer et al. (2001; Table 2). This disambiguation of gene names is offered to assist readers with interpreting published information about the *C. tropicalis ALS* family.

The current literature is limited to two papers that examine *C. tropicalis ALS* gene expression (Yu et al., 2016; Galán-Ladero et al., 2019). The analyses in both papers compared *C. tropicalis ALS* gene expression in planktonic and sessile cells. Yu et al. (2016) grew sessile *C. tropicalis* on either a polystyrene surface or a human urinary bladder epithelial cell line. Nine clinical isolates and ATCC 750 were studied. Although results varied by strain, *ALST1* (*CtrALS3797*) expression increased in sessile cells. *ALST3* (which likely combines readings from *CtrALS2293*, *CtrALS3786*, and *CtrALS3791*) had the highest expression across the three experimental conditions, perhaps consistent with the high level of *CtrALS2293* expression demonstrated here (Figure 7). Galán-Ladero et al. (2019) studied similar *C. tropicalis* properties, also using multiple clinical isolates. Although there was considerable strain variation, *ALS* gene expression generally increased in biofilm-grown cells. Interest in the role of *ALS* genes in *C. tropicalis* biofilm formation is stimulated by the key role that Als proteins play in *C. albicans* biofilms (reviewed in Lohse et al., 2018).

Gene expression patterns investigated here focus on the potential for differential *ALS* expression with growth stage or cellular morphology. Growth conditions (e.g., use of YPD and RPMI media) were selected deliberately to place *C. tropicalis* results into a well-characterized context that was developed for studies of *ALS* gene expression in *C. albicans* (reviewed in Hoyer et al., 2008). The ability of *C. tropicalis* to grow in a filamentous form (Lackey et al., 2013; Figure 7) was also leveraged to assess *ALS* gene expression changes associated with morphology. Comparative analyses were furthered by developing TaqMan assays for *C. albicans* and *C. dubliniensis ALS* genes. TaqMan assays correctly identified *C. albicans ALS* genes that were capable of high expression levels (*CaALS1*, *CaALS3*, and *CaALS4*) and those that were not (*CaALS6*, *CaALS7*; reviewed in Hoyer et al., 2008). Differential expression patterns such as upregulation of *CaALS3* in growth conditions that promote germ tube and hyphal formation were also observed (Hoyer et al., 1998a). High levels of *CaALS4* expression in saturated cultures were also noted using the TaqMan assay, as were the residual high level of mRNA in cells that were early in the growth stage in fresh medium that had been previously documented using Northern blots (Hoyer et al., 1998b). TaqMan-assessed expression of *CaALS1* showed high levels in cells transferred to fresh medium and in cells transitioning to hyphal growth (Zhao et al., 2004). However, the *CaALS1* expression level in saturated culture conditions was higher than anticipated.

Some of these *C. albicans ALS* gene expression themes were noted in *C. tropicalis* such as *CtrALS1028* increased expression in cells placed into fresh growth medium and *CtrALS941* increased expression in saturated cultures (Figure 7). None of the *CtrALS* genes showed increased expression in filamentous growth morphologies. Other genes, particularly *CtrALS2293* and *CtrAls3882-1*, were highly expressed regardless of growth condition. The highly abundant CtrAls proteins are the strongest candidates for contributing to adhesive function *in vitro*. However, the *C. albicans ALS* gene expression is known to differ *in vitro* and *in vivo* (Coleman et al., 2012), so assaying *in vivo*-grown *C. tropicalis* would better predict the display of Als proteins on cells in the host environment. Understanding which Als proteins are prominent on the fungal cell surface is the first step toward developing adhesion inhibitors that will function across pathogenic fungal species.

Although strain differences in *CtrALS* gene expression were not systematically examined here, the works of Yu et al. (2016) and Galán-Ladero et al. (2019) suggest that they exist. Strain differences in *ALS* gene expression were also noted for *C. parapsilosis* and *C. metapsilosis* (Oh et al., 2019). Although there was some strain variation in *C. orthopsilosis ALS* gene expression, *ALS* expression increases in cells that are transferred to fresh culture media compared to saturated cultures (Lombardi et al., 2019). *C. orthopsilosis ALS* gene transcription also showed a reliable hierarchy of expression level with *CoALS4220* > *CoALS4210* > *CoALS800* (Lombardi et al., 2019).

*ALS* genes in other species are known to have considerable allelic variation, even within the same strain (Zhang et al., 2003; reviewed in Hoyer et al., 2008). *C. tropicalis* genes likely have this large degree of allelic variation, as well. For example, examination of *CtrALS1030* (called “*ALS2*” in the manuscript) in 68 *C. tropicalis* isolates revealed considerable sequence variation in the coding region, localized primarily to the central tandem repeat domain (Zhang et al., 2019). The present work with strain MYA-3404 resulted in two distinct *CtrALS2228* sequences deposited in GenBank (Table 2) that primarily varied in the number of copies of the repeated sequence in the tandem repeat domain. DNA sequences in Supplementary File S3 document diploid nucleotide sequences and variation between strains within the 5′ domain of each *C. tropicalis ALS* gene reported here. Comparison between the MinION *C. tropicalis ALS* sequences and those derived from the Sequel assembly (Guin et al., 2020) reveals length variations that are probably the diploid alleles in strain MYA-3404. MinION data were validated by PCR amplification and Sanger sequencing, techniques that would preferentially amplify the shorter allele where one exists.

All six *C. tropicalis* strains examined encode the 13 *ALS* loci described here (Supplementary File S3). Genome sequencing would be helpful to determine if the strains encode additional *ALS* loci. The number of *ALS* loci varies among strains in some species. For example, examination of draft *C. parapsilosis* genome sequences available in GenBank revealed variable *ALS* gene numbers with some strains encoding only two and others having five (Oh et al., 2019). Intergenic recombination between the contiguous *C. albicans ALS5* and *ALS1* loci resulted in a reduction in the *ALS* gene number in approximately 5% of isolates tested (Zhao et al., 2011); heterozygosity at the locus was observed with some strains revealing recombination on one chromosome but not the other. In *C. tropicalis*, the recombination events that created the divergent 5′ domains of *CtrALS3882-1* and *CtrALS3882-2* are found in the six isolates examined. Divergent 5′ domain sequences were also noted for *CaALS9*, although the sequences were 89% identical compared to the 52% identity at the *CtrALS3882* locus (Zhao et al., 2007). Analysis of the adhesive phenotype of strains in which *C. albicans ALS9* was deleted and individual alleles reintegrated revealed the ability of *CaALS9-2*, but not *CaALS9-1*, to rescue the adhesion defect. It is possible that alleles of *CtrALS3882* may make different contributions to *C. tropicalis* adhesion because of their sequence differences but also because of the greater representation of *CtrALS3882-1* on the cell surface as predicted from the higher transcriptional activity from this allele (Figure 7).

Compared to discovery of the *ALS* family in *C. albicans* where genes were introduced into the literature over the period of a decade or more as they were detected using Southern blots (reviewed in Hoyer et al., 2008), advances in genome sequencing technology make it possible to detect all genes in the family at one time. Because of the complexity of the *ALS* genes in *C. tropicalis*, however, accurate assembly of the sequences was not attempted until the availability of long-read sequence technology. The Illumina MiSeq/Oxford Nanopore genome assembly (ASM694213v1) was completed during 2017 yet the large number of *C. tropicalis ALS* genes, lengthy tandem repeat regions, and unexpected allelic variation at the *CtrALS3882* locus required considerably more effort to resolve. The recent PacBio Sequel/Illumina HiSeq assembly (ASM1317755v1; Guin et al., 2020) would have provided a great time savings and suggests that other fungal genomes should be re-sequenced as newer, more accurate, long-read technologies emerge. The relatively low cost of generating accurate long-read sequence data would more than pay for itself in savings of human effort to assemble the complex jigsaw puzzle for *ALS* sequences and other gene families that contain conserved, repeated sequences.

## Data Availability Statement

The genome assembly for *Candida tropicalis* strain MYA-3404, generated using Illumina MiSeq and Oxford Nanopore MinION data, is available in GenBank under BioProject accession number PRJNA432250, BioSample accession number SAMN08439037, and Genome accession number PQTP00000000. Version 01 of the project has the accession number PQTP01000000 and consists of sequences PQTP01000001–PQTP01000029. All individual *ALS* gene sequences were deposited in GenBank. Accession numbers are noted throughout the manuscript.

## Author Contributions

LLH conceptualized the study, acquired funding, and was in charge of project administration. VH, CF, and AH conducted formal analysis. S-HO, AI, RR-B, BS, JJ, CF, AH, and LLH performed the investigation. VH, CF, AH, and LLH developed the study methodology. S-HO, AI, RR-B, JJ, VH, CF, AH, and LLH wrote the original draft. All authors contributed to the article and approved the submitted version.

## Conflict of Interest

The authors declare that the research was conducted in the absence of any commercial or financial relationships that could be construed as a potential conflict of interest.

## Footnotes

1 https://blast.ncbi.nlm.nih.gov/Blast.cgi

2 https://blast.ncbi.nlm.nih.gov/Blast.cgi

3 http://www.cbs.dtu.dk/services/SignalP

4 https://mendel.imp.ac.at/gpi/gpi_server.html

5 https://www.ebi.ac.uk/services

6 http://cgob.ucd.ie/
