# Supplementary material for: Pursuing Advances in DNA Sequencing Technology to Solve a Complex Genomic Jigsaw Puzzle: The Agglutinin-Like Sequence (ALS) Genes of Candida tropicalis
Source: Front Microbiol. 2021 Jan 20;11:594531. doi: 10.3389/fmicb.2020.594531 (PMC7856822; doi:10.3389/fmicb.2020.594531)
Supplement: Supplementary file 1 [file Data_Sheet_1.zip › SupplementaryFigureS1.pptx]

## Slide 1
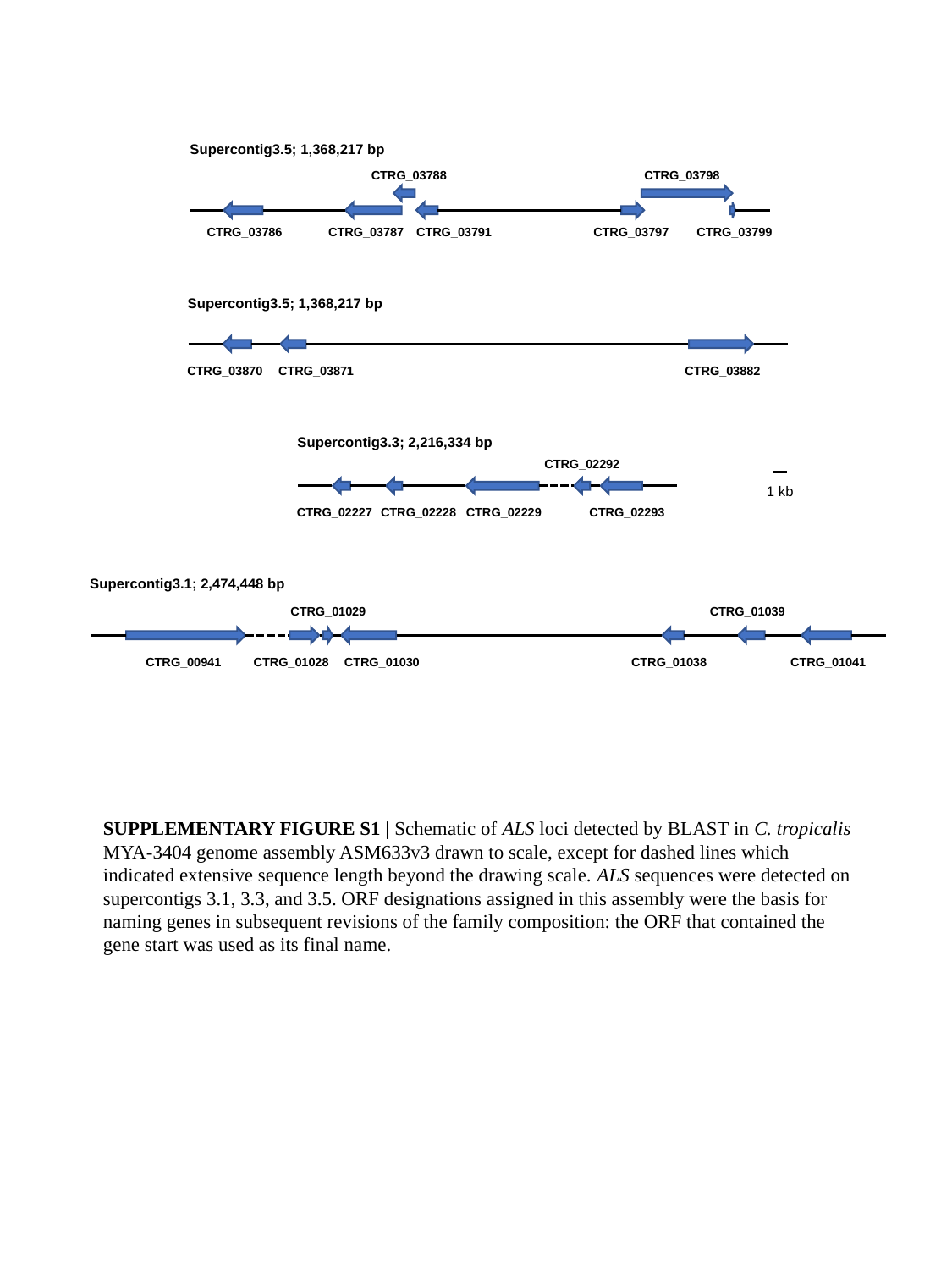

Supercontig3.5; 1,368,217 bp
CTRG_03788
CTRG_03798
CTRG_03786
CTRG_03787
CTRG_03791
CTRG_03797
CTRG_03799
Supercontig3.5; 1,368,217 bp
CTRG_03870
CTRG_03871
CTRG_03882
Supercontig3.3; 2,216,334 bp
CTRG_02292
CTRG_02227
CTRG_02228
CTRG_02229
CTRG_02293
1 kb
Supercontig3.1; 2,474,448 bp
CTRG_01029
CTRG_01039
CTRG_00941
CTRG_01028
CTRG_01030
CTRG_01038
CTRG_01041
SUPPLEMENTARY FIGURE S1 | Schematic of ALS loci detected by BLAST in C. tropicalis MYA-3404 genome assembly ASM633v3 drawn to scale, except for dashed lines which indicated extensive sequence length beyond the drawing scale. ALS sequences were detected on supercontigs 3.1, 3.3, and 3.5. ORF designations assigned in this assembly were the basis for naming genes in subsequent revisions of the family composition: the ORF that contained the gene start was used as its final name.
