# Supplementary material for: Pursuing Advances in DNA Sequencing Technology to Solve a Complex Genomic Jigsaw Puzzle: The Agglutinin-Like Sequence (ALS) Genes of Candida tropicalis
Source: Front Microbiol. 2021 Jan 20;11:594531. doi: 10.3389/fmicb.2020.594531 (PMC7856822; doi:10.3389/fmicb.2020.594531)
Supplement: Supplementary file 1 [file Data_Sheet_1.zip › SupplementaryFigureS2.pptx]

## Slide 1
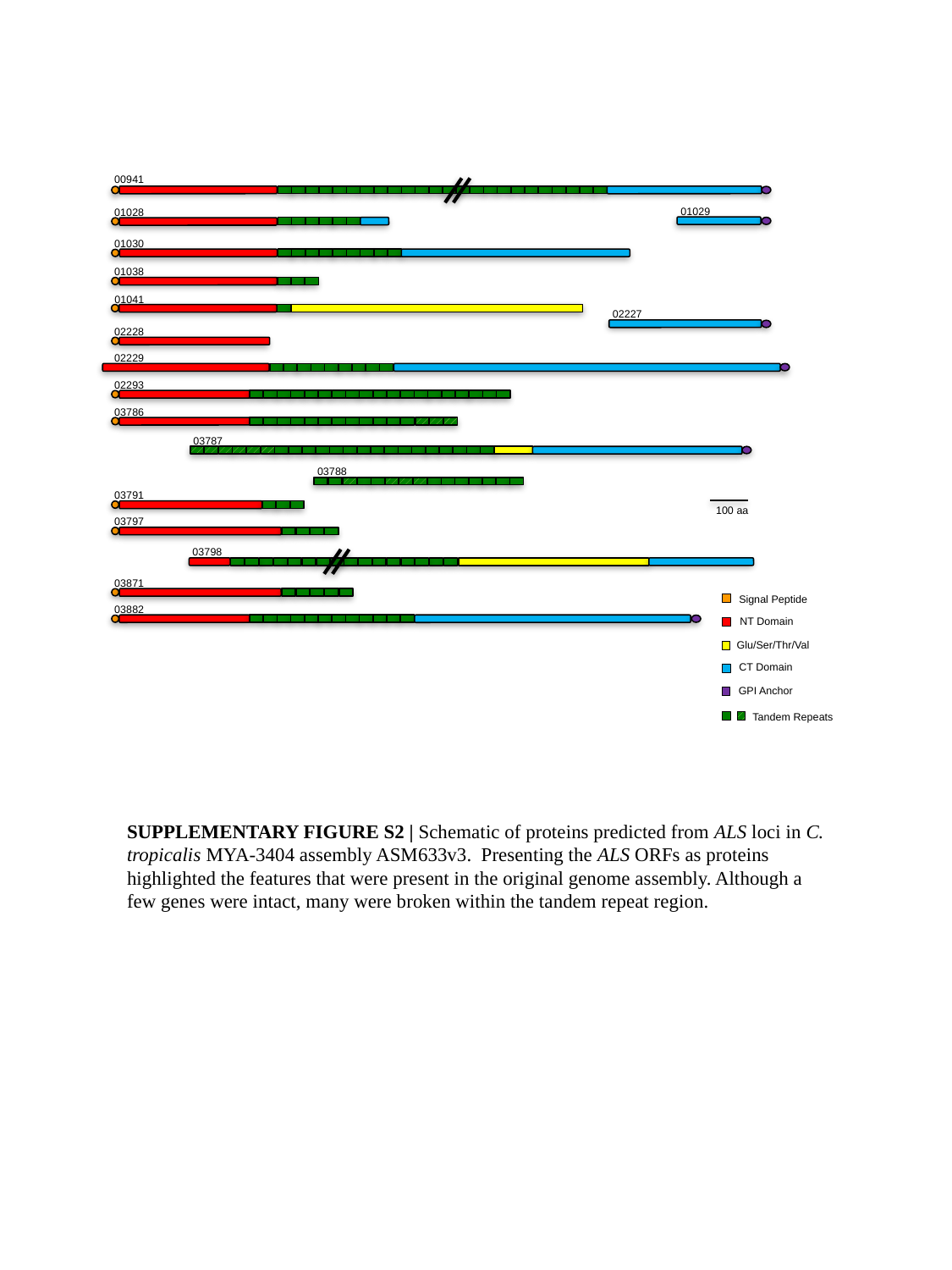

00941
01029
01028
01030
01038
01041
02227
02228
02229
02293
03786
03787
03788
03791
03797
03871
03882
Signal Peptide
NT Domain
Glu/Ser/Thr/Val
CT Domain
GPI Anchor
Tandem Repeats
100 aa
03798
SUPPLEMENTARY FIGURE S2 | Schematic of proteins predicted from ALS loci in C. tropicalis MYA-3404 assembly ASM633v3. Presenting the ALS ORFs as proteins highlighted the features that were present in the original genome assembly. Although a few genes were intact, many were broken within the tandem repeat region.
