# Supplementary material for: Pursuing Advances in DNA Sequencing Technology to Solve a Complex Genomic Jigsaw Puzzle: The Agglutinin-Like Sequence (ALS) Genes of Candida tropicalis
Source: Front Microbiol. 2021 Jan 20;11:594531. doi: 10.3389/fmicb.2020.594531 (PMC7856822; doi:10.3389/fmicb.2020.594531)
Supplement: Supplementary file 1 [file Data_Sheet_1.zip › SupplementaryFileS1.docx]

**SUPPLEMENTARY FILE S1 |** Details for assembly of *C. tropicalis* MYA-3404 genome sequence data.

Poretools V0.6.0

Data Analysis Workflow

Nanopore Sequencing Data

FastQC V0.11.4

Adaptor Trimming

*Removing Ecoli*. Reads

Canu Assembling V1.4

Nanopolish V0.6.0

PILON V1.21

MUMmer

Illumina Paired-Ends Sequencing Data

NCBI Blast

1. **Poretools**

Poretools v-0.6.0 software was used to extract 1D sequences from Oxford Nanopore MinION output file folder, convert them to fastq format. The following table shows the statistics of the output data set:

**Statistics of Ctropicalis_Raw.fastq**

| total reads | 57,554 |
| --- | --- |
| total base pairs | 563,211,492 |
| Mean | 9,785.79 |
| Median | 9,451 |
| Min | 151 |
| Max | 105,535 |
| N25 | 15,143 |
| N50 | 11,679 |
| N75 | 8,957 |

1. **FastQC**

FastQC v-0.11.4 software was also used to further access quality scores and other attributes of the data set. The following two figures were selected from the FastQC reports.


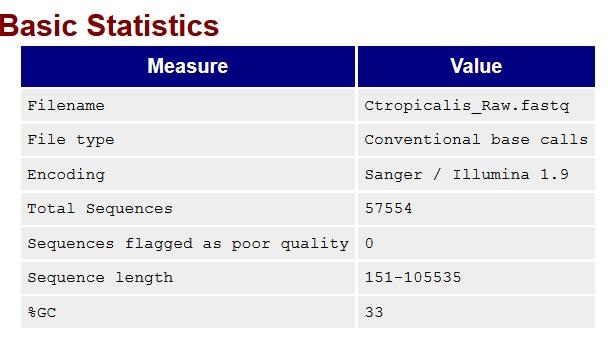


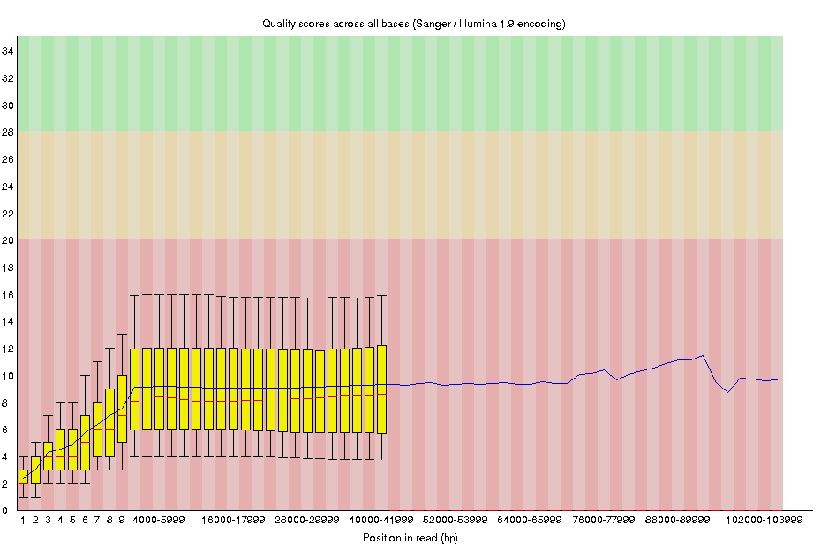


1. **Barcode trimming**

An in-house Perl script was used to trim 60 bases from each end of each raw sequence.

1. **Canu-v1.4 Assembly**

Canu assembling produced 29 contigs.

| Canu-v1.4 Assembly | | |
| --- | --- | --- |
| Contig Name | Length | Reads |
| tig00000001 | 2,384,935 | 6,823 |
| tig00000028 | 1,928,885 | 5,392 |
| tig00000023 | 1,718,445 | 5,016 |
| tig00000102 | 1,263,456 | 3,678 |
| tig00000047 | 1,036,213 | 2,872 |
| tig00000040 | 988,932 | 2,810 |
| tig00000049 | 751,765 | 1,981 |
| tig00000054 | 696,146 | 2,019 |
| tig00000100 | 602,466 | 1,559 |
| tig00000104 | 519,956 | 1,272 |
| tig00000069 | 480,569 | 1,396 |
| tig00000065 | 464,863 | 1,356 |
| tig00000077 | 410,321 | 1,192 |
| tig00000033 | 340,200 | 962 |
| tig00000012 | 201,150 | 512 |
| tig00000061 | 201,072 | 510 |
| tig00000083 | 192,773 | 547 |
| tig00000073 | 130,815 | 801 |
| tig00000093 | 78,998 | 1,429 |
| tig00000020 | 58,874 | 77 |
| tig00000103 | 55,327 | 65 |
| tig00000101 | 36,224 | 55 |
| tig00000042 | 32,126 | 33 |
| tig00000082 | 30,203 | 23 |
| tig00000005 | 30,202 | 23 |
| tig00000086 | 28,359 | 17 |
| tig00000006 | 26,682 | 18 |
| tig00000066 | 25,360 | 14 |
| tig00000052 | 22,011 | 16 |

**Canu-v1.4 Commands:**

module load canu/1.4

module load java/1.8.0_65

module load gnuplot/4.6.3

canu -p asm -d Ctropicalis-Oxford genomeSize=14m useGrid=false \

-nanopore-raw Ctropicalis_Trimmed.fastq

1. **Nanopolish V-0.6.0**

All 29 contigs from the Canu assembly were added to Nanopolish v-dev-0.6.0 for error correction. Nanopolish uses the signal-level data from the nanopore with a novel hidden Markov model to achieve a more accurate assembly.

**Nanopolish commands:**

module load biopython/github

module load parallel/20140122

module load nanopolish

nanopolish extract --type template tropicolis > reads.fa

bwa index C_tropicalisCanuAsm.fasta

bwa mem -x ont2d -t 8 C_tropicalisCanuAsm.fasta reads.fa | \

samtools view -Sb - | samtools sort -f - reads.sorted.bam

samtools index reads.sorted.bam

python nanopolish_makerange.py C_tropicalsCanuAsm.fasta | parallel --results \

nanopolish.results -P 8 \

nanopolish consensus -o nanopolish.{1}.fa -w {1} --r reads.fa -b \

reads.sorted.bam -g C_tropicalsCanuAsm.fasta -t 4 \

--min-candidate-frequency 0.1

python nanopolish_merge.py nanopolish.*.fa > C_tropicalsCanuAsmPolished.fasta

1. **PILON-v1.21**

Total 6,990,806 Illumina reads from the Illumina paired end MiSeq run were used to align to the error corrected genome from Nanopolish with BWA v-0.7.15. Then, the bam file from BWA and the corrected assembly were used as input data for PILON software.

PILON-v1.21 Commands:

module load samtools/1.3.1

module load pilon/1.21

module load bwa/0.7.15

#Build index

bwa index C_tropicolisCanuAsmPolished.fasta

#Create alignments

bwa mem –x ont2d C_tropicolisCanuAsmPolished.fasta \

C_tropicalis_ACAGTGAT_L001_R1_001.fastq \

C_tropicalis_ACAGTGAT_L001_R2_001.fastq > \

PE_Aln_C_tropicolisCanuAsmPolished.fasta.sam

samtools view -Sb PE_Aln_C_tropicolisCanuAsmPolished.sam \

-o PE_Aln_C_tropicolisCanuAsmPolished.bam

samtools sort PE_Aln_C_tropicolisCanuAsmPolished.bam \

-o PE_Aln_C_tropicolisCanuAsmPolished_sorted .bam

samtools index PE_Aln_C_tropicolisCanuAsmPolished_sorted .bam

java –Xmx20G -jar /home/apps/pilon/pilon-1.21/pilon-1.21.jar \

--genome C_tropicolisCanuAsmPolished.fasta \

--bam PE_Aln_C_tropicolisCanuAsmPolished_sorted .bam \

--outdir out

1. **Mummer Plots**

Nucleotide level comparisons between GCF_000006335.2_ASM633v2_genomic.fna from NCBI and the newly constructed assembly were done with the dnadiff program from MUMmer v-3.23.

Dnadiff Results:

|  | GCF_000006335.2_ASM633v2_genomic | C_tropicalisCanuAsmPolishedPilon.fasta |
| --- | --- | --- |
| TotalSeqs | 24 | 29 |
| AlignedSeqs | 24(100.00%) | 29(100.00%) |
| UnalignedSeqs | 0(0.00%) | 0(0.00%) |
| TotalBases | 14,630,139.00 | 15,100,058.00 |
| AlignedBases | 14,442,072(98.71%) | 14,962,687(99.09%) |
| UnalignedBases | 188,067(1.29%) | 137,371(0.91%) |
| 1-to-1 | 417.00 | 417.00 |
| TotalLength | 14,682,667.00 | 14,643,224.00 |
| AvgLength | 35,210.23 | 35,115.65 |
| AvgIdentity | 99.43 | 99.43 |
| M-to-M | 837.00 | 837.00 |
| TotalLength | 15,495,591.00 | 15,441,804.00 |
| AvgLength | 18,513.25 | 18,448.99 |
| AvgIdentity | 99.22 | 99.22 |
| TotalSNPs | 23,819.00 | 23,819.00 |
| TotalGSNPs | 11,044.00 | 11,044.00 |
| TotalIndels | 48,100.00 | 48,100.00 |
| TotalGIndels | 4,963.00 | 4,963.00 |

Mummerplot from MUMmer was used to create a graphical representation of the alignments for the newly constructed assembly and GCF_000006335.2_ASM633v2_genomic.fna.

**MUMmer-3.23 commands:**

module load MUMmer

# create report

dnadiff GCF_000006335.2_ASM633v2_genomic.fna \

C_tropicalisCanuAsmPolishedPilon.fasta

# create plot

nucmer -maxmatch GCF_000006335.2_ASM633v2_genomic.fna \

C_tropicalisCanuAsmPolishedPilon.fasta

delta-filter -m out.delta > out.delta.m

mummerplot –large –layout out.delta.m

Mummer Plot: GCF_000006335.2_ASM633v2_genomic.fna vs C_tropicalisCanuAsmPolishedPilon.fasta


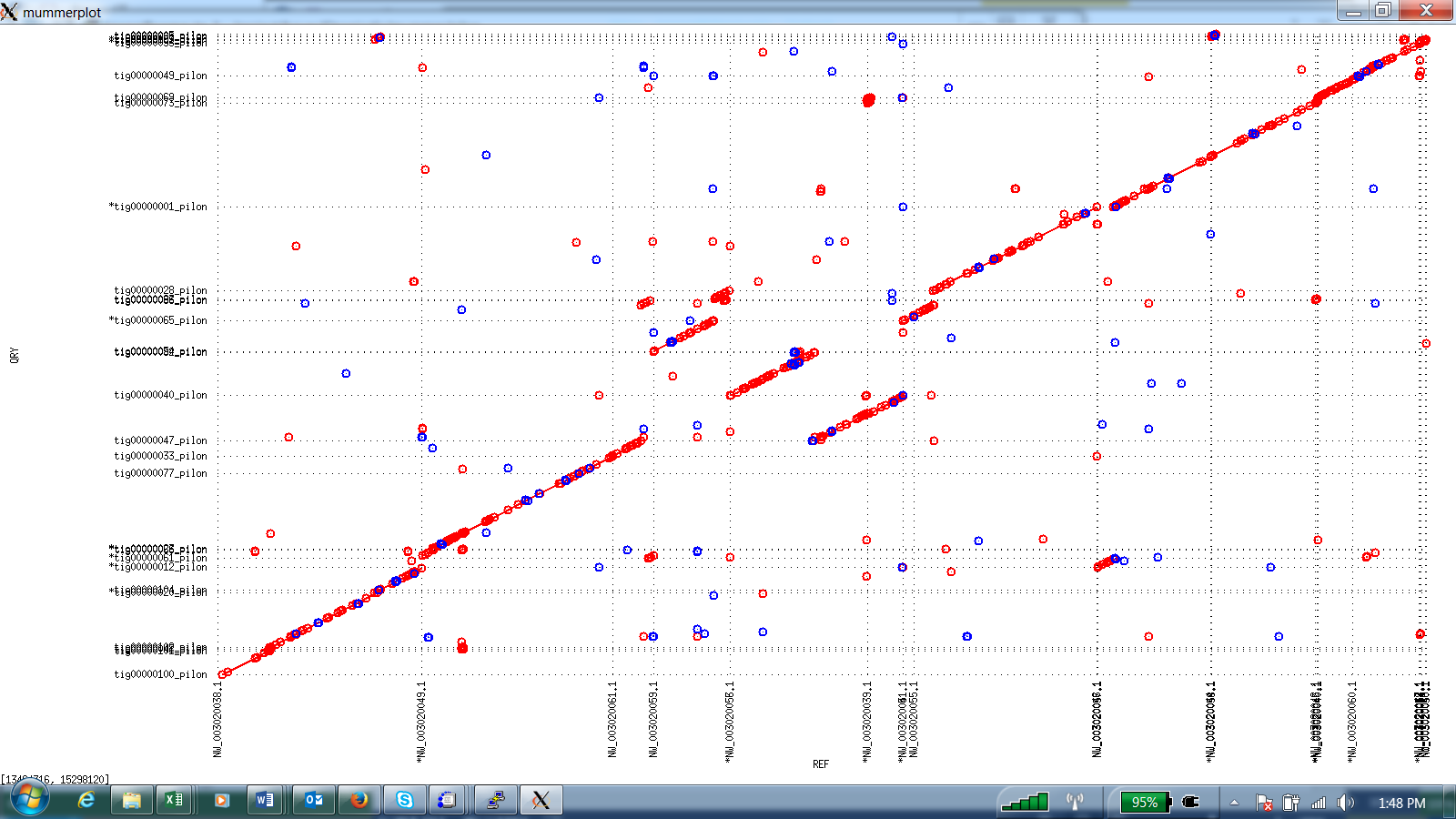


1. **NCBI Blast Resutls:**

NCBI blastn search was conducted using the new assembly against Candida (taxid:1535326).

| Contig Name | Length | Top Hits |
| --- | --- | --- |
| tig00000001_pilon | 2,451,777 | [Candida tropicalis MYA-3404 conserved hypothetical protein, mRNA](https://blast.ncbi.nlm.nih.gov/Blast.cgi#alnHdr_255729627) |
| tig00000005_pilon | 30,667 | [Candida tropicalis MYA-3404 conserved hypothetical protein, mRNA](https://blast.ncbi.nlm.nih.gov/Blast.cgi#alnHdr_255731198) |
| tig00000006_pilon | 217,174 | [Candida tropicalis MYA-3404 hypothetical protein, mRNA](https://blast.ncbi.nlm.nih.gov/Blast.cgi#alnHdr_255731232) |
| tig00000012_pilon | 206,686 | [Candida tropicalis MYA-3404 predicted protein, mRNA](https://blast.ncbi.nlm.nih.gov/Blast.cgi#alnHdr_255729047) |
| tig00000020_pilon | 60,170 | [Candida tropicalis MYA-3404 leucyl-tRNA synthetase, mRNA](https://blast.ncbi.nlm.nih.gov/Blast.cgi#alnHdr_255722260) |
| tig00000023_pilon | 1,769,004 | [Candida tropicalis MYA-3404 hypothetical protein, mRNA](https://blast.ncbi.nlm.nih.gov/Blast.cgi#alnHdr_255724143) |
| tig00000028_pilon | 1,983,186 | [Candida tropicalis MYA-3404 conserved hypothetical protein, mRNA](https://blast.ncbi.nlm.nih.gov/Blast.cgi#alnHdr_255727207) |
| tig00000033_pilon | 350,091 | [Candida tropicalis MYA-3404 conserved hypothetical protein, mRNA](https://blast.ncbi.nlm.nih.gov/Blast.cgi#alnHdr_255732805) |
| tig00000040_pilon | 1,016,891 | [Candida tropicalis MYA-3404 conserved hypothetical protein, mRNA](https://blast.ncbi.nlm.nih.gov/Blast.cgi#alnHdr_255728616) |
| tig00000042_pilon | 32,331 | [Candida tropicalis MYA-3404 opaque-specific ABC transporter CDR3, mRNA](https://blast.ncbi.nlm.nih.gov/Blast.cgi#alnHdr_255721140) |
| tig00000047_pilon | 1,066,980 | [Candida tropicalis MYA-3404 pre-mRNA splicing factor PRP8, mRNA](https://blast.ncbi.nlm.nih.gov/Blast.cgi#alnHdr_255727638) |
| tig00000049_pilon | 775,012 | [Candida tropicalis MYA-3404 conserved hypothetical protein, mRNA](https://blast.ncbi.nlm.nih.gov/Blast.cgi#alnHdr_255732066) |
| tig00000052_pilon | 22,495 | [Candida tropicalis MYA-3404 phosphatidylinositol 4-kinase PIK1a, mRNA](https://blast.ncbi.nlm.nih.gov/Blast.cgi#alnHdr_255723655) |
| tig00000054_pilon | 715,263 | [Candida tropicalis MYA-3404 conserved hypothetical protein, mRNA](https://blast.ncbi.nlm.nih.gov/Blast.cgi#alnHdr_255731485) |
| tig00000061_pilon | 206,422 | [Candida tropicalis MYA-3404 conserved hypothetical protein, mRNA](https://blast.ncbi.nlm.nih.gov/Blast.cgi#alnHdr_255725763) |
| tig00000065_pilon | 477,298 | [Candida tropicalis MYA-3404 hypothetical protein, mRNA](https://blast.ncbi.nlm.nih.gov/Blast.cgi#alnHdr_255725875) |
| tig00000066_pilon | 25,931 | [Candida tropicalis MYA-3404 hypothetical protein, mRNA](https://blast.ncbi.nlm.nih.gov/Blast.cgi#alnHdr_255725377) |
| tig00000069_pilon | 493,508 | [Candida tropicalis hsr1 gene for heat-shock related protein Hsr1](https://blast.ncbi.nlm.nih.gov/Blast.cgi#alnHdr_10697034) |
| tig00000073_pilon | 131,972 | [Candida albicans SC5314 chromosome R sequence](https://blast.ncbi.nlm.nih.gov/Blast.cgi#alnHdr_1078736431) |
| tig00000077_pilon | 422,053 | [Candida tropicalis MYA-3404 hypothetical protein, mRNA](https://blast.ncbi.nlm.nih.gov/Blast.cgi#alnHdr_255723891) |
| tig00000082_pilon | 30,863 | [Candida tropicalis MYA-3404 protein PDC2, mRNA](https://blast.ncbi.nlm.nih.gov/Blast.cgi#alnHdr_255728276) |
| tig00000083_pilon | 198,444 | [Candida tropicalis MYA-3404 hypothetical protein, mRNA](https://blast.ncbi.nlm.nih.gov/Blast.cgi#alnHdr_255731991) |
| tig00000086_pilon | 28,830 | [Candida tropicalis MYA-3404 predicted protein, mRNA](https://blast.ncbi.nlm.nih.gov/Blast.cgi#alnHdr_255731955) |
| tig00000093_pilon | 80,440 | [Candida tropicalis strain CBS 94 mitochondrion, complete genome](https://blast.ncbi.nlm.nih.gov/Blast.cgi#alnHdr_528750140) |
| tig00000100_pilon | 568,922 | [Candida tropicalis MYA-3404 conserved hypothetical protein, mRNA](https://blast.ncbi.nlm.nih.gov/Blast.cgi#alnHdr_255720674) |
| tig00000101_pilon | 36,911 | [Candida tropicalis MYA-3404 opaque-specific ABC transporter CDR3, mRNA](https://blast.ncbi.nlm.nih.gov/Blast.cgi#alnHdr_255721140) |
| tig00000102_pilon | 1,300,794 | [Candida tropicalis MYA-3404 DNA polymerase epsilon, mRNA](https://blast.ncbi.nlm.nih.gov/Blast.cgi#alnHdr_255721984) |
| tig00000103_pilon | 56,161 | [Candida tropicalis MYA-3404 predicted protein, mRNA](https://blast.ncbi.nlm.nih.gov/Blast.cgi#alnHdr_255722318) |
| tig00000104_pilon | 533,782 | [Candida tropicalis MYA-3404 conserved hypothetical protein, mRNA](https://blast.ncbi.nlm.nih.gov/Blast.cgi#alnHdr_255722448) |

**Files Created:**

Row ONT Data:

Ctropicalis_Raw.fasta

Ctropicalis_Raw.fastq

Clean ONT Data:

Ctropicalis_Trimmed.fasta

Ctropicalis_Trimmed.fastq

Clean Illumina Data:

C_tropicalis_ACAGTGAT_L001_R1_001.fastq

C_tropicalis_ACAGTGAT_L001_R2_001.fastq

Final Assembly:

C_tropicalisCanuAsmPolishedPilon.fasta

Reference Genome from NCBI:

GCF_000006335.2_ASM633v2_genomic.fna
