# Supplementary material for: Pursuing Advances in DNA Sequencing Technology to Solve a Complex Genomic Jigsaw Puzzle: The Agglutinin-Like Sequence (ALS) Genes of Candida tropicalis
Source: Front Microbiol. 2021 Jan 20;11:594531. doi: 10.3389/fmicb.2020.594531 (PMC7856822; doi:10.3389/fmicb.2020.594531)
Supplement: Supplementary file 1 [file Data_Sheet_1.zip › SupplementaryFileS2.docx]

**SUPPLEMENTARY FILE S2 |** Nucleotide sequences used to draw the *ALS* phylogenetic tree in **Figure 6**.

**>*CaALS1* GenBank Accession L25902**

ATGCTTCAACAATTTACATTGTTATTCCTATATTTGTCAATTGCAAGTGCAAAGACAATCACTGGTGTTTTTGATAGTTTTAATTCATTAACTTGGTCCAATGCTGCTAATTATGCTTTCAAAGGGCCAGGATACCCAACTTGGAATGCTGTTTTGGGTTGGTCCTTAGATGGTACCAGTGCCAATCCAGGGGATACATTCACATTGAATATGCCATGTGTGTTTAAATATACTACTTCACAAACATCTGTTGATTTAACTGCCGATGGTGTTAAATATGCTACTTGTCAATTTTATTCTGGTGAAGAATTCACAACTTTTTCTACATTAACATGTACTGTGAACGACGCTTTGAAATCATCCATTAAGGCATTTGGTACAGTTACTTTACCAATTGCATTCAATGTTGGTGGAACAGGTTCATCAACTGATTTGGAAGATTCTAAATGTTTTACTGCTGGTACCAATACAGTCACATTTAATGATGGTGATAAAGATATCTCAATTGATGTTGAGTTTGAAAAGTCAACCGTTGATCCAAGTGCATATTTGTATGCTTCCAGAGTTATGCCAAGTCTCAATAAGGTCACAACTCTTTTTGTGGCACCACAATGTGAAAATGGTTACACATCTGGTACAATGGGGTTCTCCAGTAGTAACGGTGACGTTGCTATTGATTGCTCAAATATTCATATTGGTATCACAAAAGGATTAAATGATTGGAATTATCCGGTTTCATCTGAATCATTTAGTTACACTAAAACTTGTACATCTAATGGAATTCAGATTAAATATCAAAATGTACCTGCTGGTTATCGTCCATTTATTGATGCTTATATTTCTGCTACAGATGTTAACCAATATACTTTAGCATATACCAATGATTATACTTGTGCTGGCAGTCGTCTGCAAAGTAAACCTTTCACTTTAAGATGGACTGGATACAAGAATAGTGATGCCGGATCTAACGGTATTGTCATTGTTGCT

**>*CaALS2* GenBank Accession AH006927.2**

ATGCTTTTACAATTTTTGTTGCTAAGCCTCTGTGTATCAGTTGCTACTGCAAAAGTTATTACGGGTGTTTTCAATAGTTTTGATTCGTTGACATGGACAAGAGCTGGTAATTATGCTTATAAGGGCCCAAATAGACCAACTTGGAATGCTGTTTTGGGCTGGTCTTTAGATGGTACTAGTGCAAATCCAGGAGACACATTCACATTGAATATGCCATGTGTTTTTAAATTTATTACCGATCAAACATCTGTTGATTTGACTGCTGAAGGTGTTAAATATGCTACATGTCAGTTTTATTCAGGTGAAGAATTTACAACATTTTCTTCATTAAAATGTACTGTGAGCAATACTTTAACATCATCTATTAAGGCTTTGGGTACGGTTACTTTACCAATTTCATTTAATGTTGGTGGAACAGGTTCATCGGTTGATTTGGAAAGTTCTCAATGTTTTAAGGCTGGCACCAACACAGTTACTTTTAATGATGGTGATAAAAAAATCTCAATTGACGTTGATTTTGAGAAAACAAACGAAGATGCAAGTGGATATTTCATAGCGTCAAGACTTATTCCAAGTATTAACAAAGTTTCAATCACTTATGTGGCACCACAATGTGCAAATGGCTACACATCTGGTGCAATGGGGTTCATAGTTCTCACTGGTGACACTACTATTGACTGTTCAAATGTTCATGTTGGTATTACAAAGGGATTAAATGATTGGAATTTTCCGGTATCGTCTGATTCATTAAGTTACAATAAAACTTGTTCATCTACAGGTATTTCTATCACATATGAAAATGTCCCCGCTGGTTATCGTCCATTTTTTGACGTATATACTCTGGTGTCAGGCCAGAACAGACAATTAAGATATACTAATGATTATGCCTGTGTTGGTAGTTCCTTACAAAGTAAGCCGTTCAATTTAAGATTGAGAGGATACAATAATAGTGAAGCTAATTCTAACGGTTTTGTCATTGTTGCT

**>*CaALS3* GenBank Accession AY223552.1**

ATGCTACAACAATATACATTGTTACTCATATATTTGTCGGTTGCGACTGCAAAGACAATCACTGGTGTTTTCAACAGTTTTAATTCATTGACTTGGTCTAATGCTGCTACGTATAATTATAAGGGACCAGGAACCCCAACTTGGAATGCTGTTTTGGGTTGGTCTTTAGATGGTACTAGTGCAAGTCCGGGAGATACATTCACATTGAATATGCCATGTGTGTTTAAATTTACTACTTCTCAAACATCTGTTGATTTGACTGCTCATGGTGTTAAATATGCTACATGTCAATTTCAGGCAGGTGAAGAATTTATGACCTTTTCTACATTAACATGTACTGTGAGCAATACTTTGACTCCATCTATTAAGGCTTTGGGTACTGTCACTTTACCACTTGCATTCAATGTAGGTGGAACTGGTTCTTCTGTTGATTTGGAAGATTCTAAATGTTTTACTGCTGGTACTAACACAGTTACATTTAATGATGGTGGCAAGAAAATCTCAATTAATGTTGATTTTGAAAGGTCAAATGTCGATCCAAAAGGGTACTTAACTGATTCCAGAGTTATACCAAGTCTCAACAAAGTGTCAACTCTTTTTGTTGCACCACAATGTGCAAATGGTTACACATCTGGTACAATGGGATTCGCTAACACTTATGGTGATGTTCAAATTGACTGTTCAAATATTCATGTTGGTATTACAAAAGGATTGAATGATTGGAATTATCCGGTTTCATCTGAATCATTTAGTTACACCAAAACTTGTTCATCTAATGGTATCTTTATCACATATAAAAACGTTCCTGCCGGTTATCGTCCATTTGTTGACGCTTATATTTCTGCTACAGATGTTAATTCGTACACCTTGTCGTATGCTAATGAATATACTTGTGCTGGTGGTTATTGGCAACGTGCACCTTTCACATTAAGATGGACTGGATACAGAAATAGTGATGCTGGATCTAACGGTATTGTTATTGTGGCT

**>*CaALS4* GenBank Accession AH006929.2**

ATGCTTTTACAATTTTTGTTGCTAAGCCTCTGTGTATCAGTTGCTACGGCAAAAGTTATTACAGGTGTTTTCAATAGTTTTAATTCGTTAACTTGGGCCAATGCTGCTTCTTATCCATATAGAGGTCCAGCTACTCCTACTTGGACCGCTGTAATAGGATGGTCTTTAGATGGAGCTACTGCTAGTGCTGGTGACACATTCACGTTAGACATGCCTTGTGTTTTCAAATTTATTACTGATCAAACGTCAATTGATTTAGTTGCTGATGGTCGTACTTATGCTACTTGTAATTTGAATTCTGCCGAAGAGTTTACTACTTTTTCTAGTGTGTCATGTACTGTGACTACTACAATGACTGCTGACACCAAAGCCATAGGAACTGTAACATTACCTTTCTCATTCAGTGTGGGGGGATCAGGTTCAGATGTTGATTTGGCAAATTCTCAATGTTTTACTGCAGGAATCAATACAGTTACTTTTAATGATGGTGACACTAGCATTTCCACAACAGTTGATTTTGAAAAATCAACCGTGGCCTCCAGCGATCGTATCTTGTTGTCAAGAATTTTACCCAGTCTTTCACAAGCAGTAAATCTTTTTCTTCCCCAAGAATGTGCAAATGGTTATACTTCTGGTACAATGGGATTTTCGACTGCTGGTACTGGTGCTACTATAGATTGTTCCACAGTTCATGTCGGGATATCAAATGGGTTGAATGATTGGAATTATCCAATTTCACTGGAATCTTTTTCTTACACAAAGACCTGTACATCAACAAGTGTTTTAGTAACTTTTCAAAATGTTCCTGCCGGATATCGTCCATTTGTTGATGCTTATATTTCTGCAACACGAGTCAGCTCATATACCATGCAATACACTAATATATATGCTTGTGTTGGCGCGGCTTCTGTTGATGACTCATTTACTCATACTTGGCGGGGATATAGTAATAGTCAAGCTGGTTCTAATGGTATTACCATTGTGGTA

**>*CaALS5* GenBank Accession AY227440.1**

ATGATTCAACAATTTACATTGTTATTCCTATATTTGTCGTTTGCGACTGCAAAAGCGATCACTGGTATTTTCAATAGTATTGACTCATTAACTTGGTCCAATGCTGGCAATTACGCTTTCAAAGGACCAGGATACCCAACTTGGAATGCTGTGTTGGGTTGGTCATTAGATGGTACCAGTGCCAATCCAGGGGATACATTCATATTAAACATGCCATGTGTGTTTAAATTCACTGCTTCCCAAAAATCTGTTGATTTGACTGCCGATGGTGTTAAATATGCTACTTGTCAATTTTATTCTGGTGAAGAGTTTACAACTTTTTCTTCATTAAAATGTACAGTGAACAATAATTTGAGATCATCTATTAAGGCTTTGGGTACGGTTACTTTACCAATTGCATTCAATGTTGGTGGAACAGGGTCATCAGTTGATTTGGAAGATTCTAAATGTTTTACTGCTGGTACCAATACGGTAACATTTAATGATGGCAGTAAAAAGCTCTCAATTGCTGTTAATTTTGAAAAGTCAACAGTTGATCAAAGTGGGTATTTGACTACTTCCAGATTTATGCCGAGTCTCAATAAAATTGCTACTCTTTATGTGGCACCACAATGTGAAAACGGTTACACATCTGGTACAATGGGATTCTCCACTAGTTATGGGGATGTTGCTATTGACTGTTCAAATGTACATATTGGTATTTCAAAAGGAGTAAATGATTGGAATCATCCAGTTACGTCTGAATCATTTAGTTACACTAAAAGCTGTTCATCTTTTGGTATCTCTATCACATATCAAAATGTTCCTGCCGGTTATCGTCCATTTATTGACGCTTATATTTCTCCCTCAGATAATAACCAGTATCAATTGTCGTATAAAAATGACTATACTTGTGTTGATGATTATTGGCAGCATGCACCTTTCACTTTAAAATGGACTGGATATAAGAATAGTGATGCCGGATCTAACGGTATTGTCATTGTTGCT

**>*CaALS6* GenBank Accession AY225310.1**

ATGAAGACAGTAATACTATTACATCTTTTTTTCTATTGCACGATAGCAATGGCAAAAACTATATCGGGAGTTTTCACGAGTTTCAACTCATTGACCTATACTAATACTGGTAACTACCCATATGGGGGTCCTGGTTATCCAACATGGACTGCTGTTTTAGGTTGGAGCTTGGACGGAACACTAGCTAGTCCAGGTGATACATTTACATTGGTCATGCCCTGCGTTTTCAAATTTATTACCACACAAACTTCAGTAGACTTAACTGCTAATGGTGTCAAGTATGCAACATGTACTTTCCATGCAGGGGAAGACTTTACTACTTTTTCAAGTATGAGTTGTGTAGTAAATAATGGGCTATCTTCAAATATCAGAGCGTTTGGTACCGTCAGGCTACCAATTTCATTCAATGTGGGCGGAACTGGTTCATCTGTCAACATTCAAGATTCAAAGTGTTTCACTGCTGGAACGAACACTGTAACATTTACAGACGGCGATCACAAAATTTCTACTACAGTCAATTTCCCTAAGACTCCACAATCATCTAGTAGCTTGGTTTATTTCGCAAGGGTTATTCCAAGTCTTGATAAATTATCTAGTCTTGTTGTTGCTTCTCAGTGTACTGCTGGATATGCATCCGGTGTGCTCGGATTTTCAGCAACAAAAGATGATGTGACAATTGATTGTTCTACTATACATGTGGGAATAACAAATGGTTTGAATAGTTGGAATATGCCAGTATCATCAGAATCATTTTCTTACACCAAAACTTGTACACCAAACAGTTTTATTATTACTTATGAAAATGTTCCTGCAGGTTATCGTCCATTTATTGATTCTTACGTGAAAAAATCAGCAACAGCAACGAATGGATTTAATTTGAATTACACGAATATATACAATTGTATGGATGGCAAAAAGGGAAATGATCCTCTTATATACTTTTGGACATCATACACAAATAGTGATGCAGGATCCAATGGAGCTGCCGTAGTTGTT

**>*CaALS7* GenBank Accession AF201684.1**

ATGAAGAAACTATATCTTTTATATTTGTTGGCCCTGTTTACAACGGTCATATCCAAAGAAGTTACTGGTGTTTTCAACCAATTCAATTCATTGATATGGTCTTACACATACAGAGCTCGATACGAAGAAATATCTACTCTTACCGCTAATGCTCAATTGGAATGGGCTTTGGATGGTACTATTGCCAGTCCCGGTGATACATTTACATTAGTCATGCCCTGTGTATATAAATTCATGACGTACGAAACCTCAGTGCAATTAACTGCCAACTCTATTGCATATGCCACATGTGACTTTGATGCTGGTGAAGACACTAAAAGTTTTTCAAGTTTGAAGTGTACGGTGACTGATGAGTTGACAGAAGATACCAGCGTTTTTGGAAGTGTTATTTTGCCTATTGCTTTCAATGTTGGAGGTTCCGGATCTAAATCTACGATAACAGACTCCAAATGTTTTTCAAGTGGGTACAACACTGTCACGTTTTTTGACGGAAACAATCAACTTTCTACAACTGCAAATTTTCTTCCCCGAAGAGAACTAGCGTTTGGTCTAGTTGTTAGTCAAAGACTTTCCATGTCGCTCGATACAATGACTAATTTTGTTATGTCTACACCTTGTTTCATGGGTTATCAGCTGGGTAAGTTAGGTTTTACATCTAATGATGATGATTTTGAAATTGATTGTTCTTCTATACATGTTGGTATAACTAATGAAATAAATGATTGGAGTATGCCAGTATCTTCTGTTCCCTTCGATCATACTATAAGATGTACATCACGTGCACTTTACATTGAGTTTAAAACAATTCCTGCAGGTTATCGACCTTTTGTGGATGCGATTGTTCAAATACCAACGACAGAACCTTTTTTTGTAAAATATACAAATGAGTTTGCCTGTGTGAATGGCATATACACGTCCATACCTTTCACAAGTTTCTTTTCTCAGCCAATTTTATATGACGAGGCTTTAGCTATTGGTGCAGACCTAGTTCGTACC

**>*CaALS9-1* GenBank Accession AY269423.1**

ATGCTACCACAATTCCTATTGTTATTGCTATATTTGACAGTTTCGACTGCAAAAACGATTACCGGTGTTTTCAATAGTTTCAACTCATTAACTTGGGCCAATGCTGCTAATTATGGCTACCAAACACCAGAGACTCCAACTTGGACTGCAGTTTTAGGCTGGTCTTTAAATAGCACTACCGCTGACGCAGGAGACACGTTTACCTTGATTATGCCTTGTGTGTTTAAATTTATAACTAGCCAAACATCTGTTGATTTGACTGCTGATGGTGTTAGCTATGCCACTTGTGACTTTAATGCTGGTGAAGAATTTACAACATTTTCTTCCTTATCATGTACTGTGAACAGTGTTTCAGTATCATATGATAAGGCTTCTGGTACGGTCAAATTGCCCTTTTCGTTTAATGTGGGTGGAACAGGTTCGTCAGTTGATTTGACAGATTCCAAATGTTTTACTGCCGGAAAAAATACCGTCACTTTTACCGATGGCGATACAGAAATTTCTACTAGTGTTGATTTTCAAGCATCTCCAATATCCTCCAGTGGCTATATTGCAAGCGCACGAGTTGTTCCTAGTCTCAATAAAGCTTCAAGTCTTTTTGTGCTGCCACAATGTGAAAATGGTTACACATCTGGTATAATGGGATTTGTAACTAGTCAGGGTGCCACTATTGATTGCTCAAATATCAATATAGGGATATCGAAAGGTTTAAATGATTGGAATTTTCCAGTAAGTTCAGAATCATTTACTTACACAAAAACTTGTTCGTCAAGCGGTATTATAGTTGAATACGAAAATGTTCCTGCTGGGTATCGTCCTTTTGTTGATGCATATATTTCTTCAGAAAATGTTGAGCAATATACCTTGACGTACGCAAATGAGTATACTTGTAAAAATGGCAATACTGTGGTTGATCCATTCACCTTGACATGGACGGGATATAAAAATTCTGAAGCAGACTCTAACGGGGATATAATCGTAGTT

**>*CaALS9-2* GenBank Accession AY269422.1**

ATGCTTCCACAATTCATATTGTTATTCATATCTTTGACAGTGTCGACTGCAAAAACTATTACTGGTGTTTTCAATAGTTTTGACTCATTGACATGGACTAGATCCGTTGAATATGCTTACAAAGGGCCAGAGACTCCAACTTGGAATGCAGTTTTAGGGTGGTCCTTAAATAGTACCACTGCTGACCCAGGAGACACATTCACCTTGATTTTGCCTTGTGTATTTAAATTTATAACTACCCAAACATCTGTTGATTTGACTGCTGATGGTGTTAGCTATGCCACTTGTGACTTTAATGCTGGTGAAGAATTTACGACATTTTCTTCCTTATCATGTACTGTGAACAGTGTTTCGGTATCATATGCTAGGGTTTCTGGTACGGTCAAATTGCCCATTACATTCAATGTAGGTGGAACAGGTTCTTCAGTTGATTTGGCAGATTCCAAATGTTTTACTGCCGGAAAAAACACTGTGACTTTCATGGATGGCGATACAAAGATTTCTACCACTGTTGATTTTGACGCGTCTCCAGTATCACCCAGTGGTTATATTACAAGCTCACGAATTATTCCTAGTCTCAATAAACTTTCAAGTCTTTTCGTGGTGCCACAATGTGAGAATGGTTACACATCTGGTATAATGGGATTTGTAGCTAGTAACGGTGCTACTATTGATTGCTCAAATGTCAATATAGGAATATCAAAAGGTTTAAATGATTGGAATTTTCCAGTAAGTTCAGAATCATTTTCTTACACGAAAACTTGTACGTCAACCAGTATTACAGTTGAATTTCAAAATGTTCCTGCTGGGTATCGCCCTTTTGTTGATGCATATATTTCTGCAGAAAATATTGATAAATATACCTTGACGTACGCAAATGAGTATACTTGTGAAAATGGCAATACTGTGGTTGATCCATTTACTTTAACATGGTGGGGGTATAAAAACTCTGAAGCAGACTCTGACGGGGATGTGATCGTAGTT

**>*CdALS64210* GenBank Accession FM992693.1**

ATGCTTCAACAATTTATATTGTTATTCGTATATTTGTCGGTTGCGACTGCAAAGACAATCACTGGTGTTTTTAACAGTTTCGACTCATTGACTTGGTCCAATGCTGCTAATTACGCTTTTAAGGGTCCCGGATACCCAACTTGGAATGCTGTCTTAGGTTGGTCTTTAGACGGCACTACTGCAGATCCAGGAGACACATTTACATTGATCATGCCTTGTGTGTTCAAATTCATAACTACCCAAACATCTGTTGATTTGACCGCCAATGGTGTTAAATATGCTACATGTCTGTTTTATTCGGGTGAAGAATTCACAACTTTTTCATCATTAACATGTACTGTTAACAGTGCTTTAACCTCATCTGTTAAGGCTTTTGGTACCGTTACTTTACCCATCTCATTCAATGTAGGTGGAACGGGATCGTCAGTTGATTTAGAAGACTCCAAATGCTTCACTGCTGGTACCAACACCGTCACTTTTACCGACGGCGACAACAAAGTCTCAACTACTGTTGATTTTGAAAAATCAACAGTTGATTCAACTGGATATTTGACTTCCTCCAGACTTATGCCAAGTCTCAATAAAGTCACATCTCTTTTTGTAGCACCACAATGTGCAAGAGGTTATACATCTGGTACAATTGGATTTTCGAGTAGTAATGGTGGTGTTTCTTTTGACTGTTCAAATGTTCATGTTGGAATTACAAATGGTGTGAATGATTGGAATTTTCCAGTGTCATCAGAATCATTTAGTTACACTAAAACTTGTTCGTCTACAGGTATTACAATCACATACAGAAATGTTCCTGCTGGTTATCGTCCATTTATCGACGCTTACATTACTGCTTCGGATGTTAACCTGTATACCTTGTCATATACCAATGATTATACTTGTGTTGGTGGTAGCGTGCAACATAAACCATTTACTTTAAGATGGTCTGGATACAAAAATGGGGAAGCCGGGTCTAACGGTATTGTCATTGTTGCT

**>*CdALS65010* GenBank Accession FM992693.1**

ATGTTTTTACGATTTTTGTTGCTATGCATCTGTTTATCAGTTGCTGGTGCAAAGGTAATTACGGGTATTTTCAATAGTTTTGATTCATTGACTTGGACCAATGCTGCTAGATACCCGTTTAAGGGACCCGGATACCCAACTTGGAATGCTGTTTTAGGTTGGTCTTTAGATGGAACTGTTGCTAGCCCAGGTGACACCTTTACTTTGATCATGCCTTGTGTATTCAAATTTATTACTAATCAAACATCTGTTGATTTGATTGCTGATGGGGCAGACTATGCTACATGCCAGTTCCATGCAGGTGAAGAATTTACAACCTTTTCATCCTTGACGTGTACTGTGAGCAGTGCTTTGAACCCATCTATTAAGGCTCTTGGTACAGTTACTTTACCCATCTCATTTAACGTAGGTGGAACAGGATCATCGGTTGACTTAGAAAATTCCAAGTGCTTTACTGCTGGTACCAACACGGTCACATTCACTGATGGGAATAACAAAATTTCAACTACTGTTGAGTTCAATAAAACCACAGTTGATCCAAGTGGATACTTGACTTCCGCCAGACGTATTCCAAGTCTCAATCAAGTTACATCTCTTTATATGGCCCCACAATGTGTGAATGGTTACACTTCTGGTATAATGGGAATTTCGAGTAATAGCGGCATTGATATTGACTGTTCAACTGTTCATGTTGGGATTTCAAAAGGAGTGAATGATTGGAATTTTCCAGTATCATACGATTCATTTAGTTACAAGAAAACTTGCTCACCTACTGGTATTTCTATCACATATGAAAATATCCCTGCAGGTTATCGTCCATTTATTGACGCTTACATTTCTGCTTCAGGAGTTAAACTGTACGCTTTGTCATATGCTAATAATTATACTTGTGTTGATAGCAGCTTTCAAAATAAACCATTTACTATAAGATGGACCGGATACAATAGTAGCGAAGCTGGTTCCGACGGTGTTGTCATTGTGGTA

**>*CdALS64800* GenBank Accession FM992693.1**

ATGTTTTTACGATTTTTGTTGCTATGCATCTGTTTATCAGTTGCTGGTGCAAAGGTAATTACGGGTATTTTCAATAGTTTTGATTCATTGACTTGGACCAATGCTGCTAGATACCCGTTTAAGGGACCCGGATACCCAACTTGGAATGCTGTTTTAGGTTGGTCTTTAGATGGAACTGTTGCTAGCCCAGGTGACACCTTTACTTTGATCATGCCTTGTGTATTCAAATTTATTACTAATCAAACATCTGTTGATTTGATTGCTGATGGGGCAGACTATGCTACATGCCAGTTCCATGCAGGTGAAGAATTTACAACCTTTTCATCCTTGACGTGTACTGTGAGCAGTGCTTTGAACCCATCTATTAAGGCTCTTGGTACAGTTACTTTACCCATCTCATTTAACGTAGGTGGAACAGGATCATCGGTTGACTTAGAAAATTCCAAGTGCTTTACTGCTGGTACCAACACGGTCACATTCACTGATGGGAATAACAAAATTTCAACTACTGTTGAGTTCAATAAAACCACAGTTGATCCAAGTGGATACTTGACTTCCGCCAGACGTATTCCAAGTCTCAATCAAGTTACATCTCTTTATATGGCCCCACAATGTGTGAATGGTTACACTTCTGGTATAATGGGAATTTCGAGTAATAGCGGCATTGATATTGACTGTTCAACTGTTCATGTTGGGATTTCAAAAGGAGTGAATGATTGGAATTTTCCAGTATCATACGATTCATTTAGTTACAAGAAAACTTGCTCACCTACTGGTATTTCTATCACATATGAAAATATCCCTGCAGGTTATCGTCCATTTATTGACGCTTACATTTCTGCTTCAGGAGTTAAACTGTACGCTTTGTCATATGCTAATAATTATACTTGTGTTGATAGCAGCTTTCAAAATAAACCATTTACTATAAGATGGACCGGATACAATAGTAGCGAAGCTGGTTCCGACGGTGTTGTCATTGTGGTA

**>*CdALS64610* GenBank Accession FM992693.1**

ATGTTTTTACAATTTTTGTTGCTATGCATCTGTTTATCAGTTGCTACTGCAAAGGTAGTTACAGGCATTTTCCATAGTTTTGATTCGTTGACTTGGACCAATGCTGCTTCTTATCCGTATAGAGGTCCATCAACTCCTACTTGGAATGCTGTTATCGGTTGGACTTTGGATGGAGCTGTTGCTAGTCCTGGTGACACATTCACATTGAACATGCCTTGTGTTTTCAAATTTATTACTGATCAAACTTCAGTTGATTTAGTTGCTGATGGTCGTACTTATGCTACATGTGATTTATATTCTGGAGAAGAGTTTACTACTTTTTCAAGTTTGAAATGTACTGTTAGTAATGCATTGAATTCTCAAACTAAGGCCCTAGGAACAGTAACTTTACCATTGTCCTTCAATATTGGTGGATCAGGTTCAGATGTTGATATTACAAGTTCCCAATGTTTTAAAGAAGGAACAAACACTGTTACATTTAACGATGGTGACACTACATTTTCTACAACTGCTAATTTCCAAAGATCAGATGTGAATGCAAATGATCGTATCTTACTTTCAAGAATTTTACCCAGTCTTGCAAAATCAGTAACTATTTTTATCCCTCCAAGATGTGCAAGTGGTTATTCTTCGGGTACAATGGGGTTTTCAACTGCTGGTACTGATGCCATTATTGATTGTTCTACTGTTCATGTGGGGATATCAAATGGTTTGAATGATTGGAATTATCCAATAGAGCTGAAGTCGTTTTCTTATACAACAAGTTGTTCATCAAAGGGTGTTCTGGTTACCTATCAGAATGTTCCTGCTGGATATCGTCCATTTGTTGATGCTTATATTTCTGCGCTCACCTCATATACTATGCAATACACAAATCAGTATACTTGCGTTGGTGCTCGCCCTGTTGATGCTTCTTTTAGTTATAATTGGCTTGGCTATGATAATGCCGAGGCTGGTTCTAGAGGTATTACCATTGTCGTA

**>*CdALS64220* GenBank Accession FM992693.1**

ATGCTCCAACAATTTGTATTGTTATTCATATATCTGACAATTGCGACCGCAAAGACAATTACCGGTGTTTTTAACAGTTTTGACTCATTGACATGGACTAACTCCGGTAATTATGGCTTCAAAGGACCAGAGACTCCCACTTGGAATGCAGTTTTAGGCTGGTCTTTAAATAGTACTATTGCAGACCCAGGTGACACATTTACTTTGATCATGCCATGTGTATTCAAATTCATAACCACCCAAACATCTGTTGATTTGACTGTTGATGGTGTTAATTATGCTACATGTCTGTTTTATTCAGGTGAAGAATTTACGACCTTTTCATCCTTAACATGTACCGTGAACAATGTTTTGACGCCATATGCTATGGCTTCGGGTACCGTTACTTTACCCATCTCATTCAACGTAGGTGGATCAGGCTCATCGGTTGATTTGGAAGATTCTAAATGTTTTACTGCTGGTACCAACACAGTTACATTCAATGATGGTGATACAAAGATTTCTACTACTGTTGATTTTGATGCATCTCCAGTAGCTTCCAGTGGCTACATTACAAGCTCACGGATTATTCCAAGTCTTAATAAAGTTTCAAGTCTTTATGTGGTGCCAGAATGTGAAAGCGGTTATGCATTTGGTGTAATGGGGTTTGTAGCTAGCAACGGTGCTACTATTGATTGCTCAAATGTTCATATTGGTATATCAAAAGGTTTGAATGATTGGAATTTTCCAGTGTCATCAGAATCGTTTTCTTACACCAAAACCTGTACGTCAGCCAGTATTACAGTTGAATTCCAGAATGTTCCAGCTGGATATCGTCCGTTTGTTGATACGTATATTTCTGCAGAAAATGTGGGTAGATACACCTTGACGTATGCTAATGACTATACCTGTAACAATGGCTACTCTGTAGTTGATCCATTTACCTTAACATGGTGGGGATATAAGAATAGTGAGGCAGATTCTGATGGTGCTATAATCGTAGTT

**>*CdALS86290* GenBank Accession FM992690.1**

ATGAAGGGAGTAGTACTATTACATCTTTTCTTCTATTGTGCAATAGCAACAGCCAAAACTATATCGGGAGTCTTTACGAGTTTTAACTCTTTGACTTATGCTAATACTGGTAACTATCCATACGGGGGTCCTGGTTATCCAACATGGACTGCAGTATTAGGTTGGAGTTTGGACGGAACTGTAGCTAGTCCAAGTGATACATTCACATTGATCATGCCATGTGTTTTCAAATTTATTACCACGCAAACTTCGGTAGACTTAACTGCTAATGGTGTCAAGTATGCAACATGTACTTTCCACGCAGGGGAAGATTTTACTGCCTTTTCAAGTATGAGTTGTGTGGTAAATAATGGGTTGACTTCAAATATTAAAGCATTTGGTACCGTAAGGATACCAATTTCATTTAATGTTGGTGGAACTGGATCTTCTGTCAATCTTCAAGATTCCAAGTGCTTTACAGCCGGAACTAACAGTGTCACCTTTACCGATGGAGATCACAAAATTTCTATTCCAGTGGATTTCCCCAAAACACCGGAATCATCTAGTGGTTTGATTAAATACTCAAGAGTTATTCCAACCCTTGATAAACTATCCAGTCTTGCAGTAGCCTCTCAGTGTACCGCTGGATATAAGTCTGGTGTGCTCGGGTTTTCTGCAACAAAGAACGATGTGACAATTGAGTGTTCAAATGTACATGTGGGGATAACAAATGGTTTGAATAGTTGGAATATGCCGGTGTCATCAGACTCATTTTCTTATACAAAAACTTGTACTTCAAGTAGTTTTATCATTACCTATGAAAACGTTCCTGCAGGTTATCGTCCTTTTATTGATACATATGTGAAAAAAACATCAACAACATCGACCGGATTTAATTTGAATTACACGAATTCATACGTTTGTACTGATGGCAAAAAGGGAAATGACCCACTTATTTACTTTTGGACTTCGTACACCAATAGTGATGCAGGATCCGATGGAGCTGTTGTAATTGTT

**>*CdALS86150* GenBank Accession FM992690.1**

ATGAACAAATTATATCTTCCATATTTGTTGGCTTTGATCACAACGGTCATATCTAAAGAAATTACTGGAGTCTTTACTAAATTTGATTCATTAATATGGTCATTTACATACAGAGCCCGTTACGAAGAAATTCCTACTCTTACTGCTAATGCTCAAATGGAATGGGTTTTGGATGGTACTATGGCCAGTCCAGATGATACTTTTACATTAGTCATGCCCTGTGTGTATAAATTCATGACGTATCAATCATCGGTACAGTTAACTTCCAATTCTATTCCATATGCCACATGTAACTTTGATGCAGGTGAAGATTCCAAAACTTTTTCCAGTTTGAAGTGTACAGTGACCAATGAGTTGACTGAGAATACCAATGTTTTTGGAGACATTATCTTGCCGATTGTATTCAATGTTGGAGGTTCGGGTTCCGAATCTACGCTAAAGGACTCGAAATGCTTTTCAGGTGGGTACAATAGTGTAACATTTTTTGATGGTGATAATCAACTTTCTACAACTGCATATTTTGCTCCTCGAAGAGATCTTGCTTATGGTCTCGTTGTGGGTCAAAGACTTTTTGCGTCACTTGATAAAATGACTAATCTTGTCATGTCCACTCCATGTCTCACGGGTTATCAGCTGGGTAAATTAGGTTTTTCATTTGCTGATGATGATTTTGAAATTGACTGTTCTTCTATACATGTGGGTATAACCAATGAAATAAATGATTGGAGTATGCCCGTATCTTCTGTTCCCTTCGATCATACTAGAAGTTGCACATCACGTGCTCTTTATATTGAGTTTCAAGAAATTCCTGCAGGTTACCGACCTTTTGTGGACGCGATCGTTCAAATACCAACAACAAAACCTTTTTACGTACAGTATACAAATGAGTTTACTTGTGTTAATGGCCTTTTCACATCCTTACCTTTCACAAGTTTCTTTTCTCAGTCTATTCTATATGACAAAGCTTTAGTTAACGGAGCGGATTTGGTT

**>*CpALS4770* GenBank Accession MH753532.1**

ATGATTAAACAATTATCATTTGCCTCCGCGTTTATTGCCTTTGTGCTTACAACCTTCGTTCAAGCAACTGATATTAGCAATGTTTTCCAAAGCTTTGACAGTTTAACTTGGCAGAATGGAGCAAACTATAGGTACAGAACACCAGCAGCTCCAAGTTGGATTGCACAATTGTCTTGGCACATATTAGGTTCCAATGTCAAACCAGGTGATACATTCACATTGAACATGCCATGTGTATTCAAGTTTACCACCTCTCAAAAGACTGTTGATTTGAAAGTAGGCGGCACCGTGTATGCCACATGCCAATTTGCTCCAGGTGATTTAGTCGTTGCATATTCACAATTGAAATGTACTGCCAGCGATAATGTTAAAGATAGCACTGATGCTACTGGTACTGTTAGATTCCCTTTCACATTCAATGTTGGTGGGTCTGCTGGTGCAGTTGATTTGCAAAACTCAAAATGTTTCACACCAGGTACTAATGAAGTAACTTTTACTGATGGTGATAAAAAGTTAACCGTAATTGCCAATTTCCAAGGTGGTTCCAATACCAATACAGGCTCAACCGAAGATATCGTATACAGCAACCGCCTTGTTCCAACCTTGAACAAGCAGCAATTATACTTATTGGGAGGCACGTGTCCAAATGGTTACAGAAGGGGAACGTTGGGAATCACAACCGTAGGGGGAACCTTTGATTGTTCTAGTATTCACTCAGCGATCACAAACAATTTGAATGAGTGGTTCTTACCAGAGGTGGTAGAGACTATTTTAGCTACATCCCGCTGTAATGGCCAATCATATACAATCAATTACGACAACATTCCCGCTGGTTATAGACCTTTTATCGATATTTTAATTTCGCGTCCAGTAGGTCAAGTATTACGCACAACTTATACAAATAGATTTCAATGTGCAGGTCTGCTCACCACTACTGACAGGTCCTTGTCAGTGACTTGGGCAGAATATAGAAATAACGAAGCTGGTGCTAATGGTAATGAGGTCGTTGTTACTACATCTACATGGCTC

**>*CpALS4780* GenBank Accession MH753533.1**

ATGGTCAAACACTTGCAATTTGTGACGATATTGGTGGCCTTTACACTTACAGCCCTCACTCAAGCAGCTGAGATTAGCAATGTTTTCCAAAGCTTTGATAGTTTAACTTGGGAAAACGGTGCAAGCTACAGGTACAGAACACCAGCAGCTCCAAGTTGGATTGCACAATTGTCTTGGAAGATTTTAGGTTCCAATGTCAAACCAGGCGACACATTCACATTGAACATGCCATGTGTATTCAAGTTTACAACAACACAACAGAGCGTTGACTTGGACGTCGGAGACACGGTATATGCTACATGTCGTTTCGAACCAGGTGATTTAGTCGTTGCATATTCACAATTGAAATGTACTGCCAGCGATAATGTTAAAGATAGCACTGATGCTACTGGTACTGTTAGATTCCCTTTCACATTCAATGTTGGTGGGTCTGCTGGTGTAGTTGATTTGCAAAACTCAAAATGTTTCACACCAGGTACTAATGAAGTAACTTTTACTGATGGCGATAAGAAATTAACAACCACTGCAAACTTCCAAGGAGGCTCCAATACCAACCCAGGTACATCAACTAATAACATTGTCTTCAACAATCGTGTTGTTCCTTCTTTGAACAAACAGCAATTGTATTTATTGGGAGGTACTTGTCCAAATGGTTATAGAAGTGGAACATTAGGAATAACAGTCAGTGGAGGTACACTTGACTGTTCAAGTATTCACTCAGCTATTACGAACAAATTGAATGGCTGGTTCTTACCAGAGGTGGCAGAAGCTATCTCGGCCTCAACCAATTGTAATGGTCAATCCTACACAATAAACTACAATAATATCCCAGGCGGTTATAGACCTTTCCTTGATGTTTTGGTCCAACGTCCAACAGGTCAAGTGTTACGCACAAATTACATAAACAGATATCAATGCGCGGGAGAAATTTTCGTACGTGACAACTCACAATCAATAAAATGGGGAGAATATAGAAATAACGAAGCTGGTGCTAATGGTAATGAGGTCGTTGTTACTACATCTACATGGCTC

**>*CpALS4790* GenBank Accession BK010629.1**

ATGACGCGAATGCACATATGGGCTCAGTTGTTGATATTGCTATATTCCCTAACACTAACAACAGCCGCGCAAGTATCGGGAATTTTCACCAGTTTCAACTCGCTTACTTGGAAATCCGCAAGCAATTACAGGAATGCCGCACCAAACTACCCTACATGGACGGCTGTTTTGGGGTGGTCTTTAGATGGAGCCAAGGTGAATCCGGGGGATACTTTTACATTGACAATGCCCTGTGTTTTCAAATTCATTACGACACAAACTTCAGTTGATTTGAGCGCCGATGGAGTTTCATACGCCACATGTCAATTGAATCCAGGTGAGATCCTCGTGACATATTCGACATTAACATGTACCGTCAATTCTGCACTCAGATCAAATATGGAAGCAACAGGAACATTGTCACTTCCTTTGTCATTCAATGTTGGTGGATCCGGAGGTAATGCTGATGTAGCTGATGCATCATGCTTCAAAGTTGGGCAAAACACAGTTACGTTTACAGATGGATCGAATTCGATCTCCACGACTGCTAATTTTAAACAAGGTGATTACCAGTTAGGAACTTATGATAAATTCATAAATTACAGACTTATACCCTCATTGAATGAGGCCCAGCATTATATGGTCAGTGGACCTTGTGCTAAAGGGTACGTTTCGGGAACCATTGGGCTTGCAACATCTGATAGTGGACTGATTGATTGTTCCAATTGGCATGTTGGTTACTCCAATGATTTCAATGAATGGGCGTTTCCAAAAAGTTTTTCTTCGGATTATACTGTTACAAGTCTGTGTCTGAGTTCGCAATTACTTGTCAGTTTTAAAAACGTTCCCGCTGGTTACAGACCATTTATTGATGCATTGTTTCGAGTTCCTAATGGTGCAGGAGTTAAAGTCACCTATATCAATACAGCGTCGTGTGTTGCGGATACTAAGCAACAAAACTGGGGTGAAGAAGGATACGGGTGGGGATCGTATCAAAATGGCGAGGCGGGCGCCAACGGGATTATTGTTGTACTCACGACTTCAACAATTCTT

**>*CpALS4800* GenBank Accession BK010630.1**

ATGGTCAAACACTTGCAATTTGTGGCGATATTGGTGGCCTTTACACTTACAGCCCTCACTCAAGCAGCTGAGATTAGCAATGTTTTCCAAAGCTTTGATAGTTTAACTTGGGAAAACGGTGCAAGCTATAGGTACAGAACCCCTTTAACTCCTAGTTGGATTGCACAATTGTCTTGGAAGATTTTAGGTTCCAATGTCAAACCAGGCGACACATTCACATTGAACATGCCATGTGTATTCAAGTTTACAACAACACAAGAGAGTATTGACTTGGACGTCGGAGACACGGTATATGCTACATGTCGTTTCGAACCAGGTGATTTAGTCGTTGCATATTCACAATTGAAATGTACTGCCAGCGATAATGTTAAAGATAGCACTGATGCTACTGGTACTGTTAGATTCCCTTTCACATTCAATGTTGGTGGGTCTGCTGGTGTAGTTGATTTGCAAAACTCAAAATGTTTCACACCAGGTACTAATGAAGTAACTTTTACTGATGGCGATAAGAAATTAACAACCACTGCAAACTTCCAAGGAGGCTCCAATACCAATACGGACAACACCCCAACTGACGACATTGTTTACTCTAACCGAGTTGTCCCATCTTTGAACAAACAGCAATTGTATTTATTGGGAGGCACGTGTTCAAATGGTTATAGAAGTGGAACATTAGGAATAACAGTCAGTGGAGGTACACTTGACTGTTCCGCTCTTCACTCAGCTATTACGAACAAATTGAACGGTTGGTTTTTCCCAGAGGTGGCAGATGCTATTTCTGCTTCTTCAAGTTGTAATGGCCAGTCTTATACAATCAATTACGACAACATTCCCGCTGGTTATAGACCTTTTATCGATATTTTAGTTTCTGTTCCCAATGGTCAAAAATTACGCACAACTTATACAAATAGTTATAAATGTGTTGGCGAACAGCGCGCTCGCGACAAGTCAAAGGTTGTGACATGGGGTGCATACAATAACAATGTGGCTGGTGCCAATGGTAATGAAGTCATTCTTACTACATCTACATGGCTT

**>*CpALS660* GenBank Accession MH753534.1**

ATGAAAAACTGGAATTCATTTGTGGCCATTTTCACGGCTTTCACATTCACAACGCTTACACTCGCTTCTGAAGTTACCGATGTTTTTCAAAGCTTTGACAGTTTGGTATGGCAAAATGCCGCCAACTACCAGTACAGTACTCCTGCAGCCCCAAGTTGGATTGCAACCTTATCCTGGAAGATTTTGGGCTCAAACGTGCATGCTGGTGATACTTTTACTTTAAATATGCCCTGTGTTTTCAAGTTTACCACCACTCAAGACAGCGTCGACTTAACGGTTGGCGATACTGTTTATGCTACTTGTCAATTTGCTCCTGGTGATTTAGTTGTTGCTTACTCCCAATTAAAGTGTACTGCTAGTAACAATGTTAAAGCAAGCACAAATGCTGCTGGTTCTGTTCATTTTCCTATTGCTTTCAACGTTGGGGGATCAGCAAATTCTGTCGATTTACAAAATTCCCAATGTTTTACCGCTGGTTCCAACCAAGTTACATTCACTGACGGTGACAAGGAGTTGACTACCACAGCAAACTTTCAAGGAGGAACAAACACGAATGGAAATTCGCCAACTGATACTATTGTTTACAACAATCGTGTAGTCCCATCGTTGAACAAACAACAGTTGTACTTGCTTGGTGGAACTTGTCCTAATGGATATAGGAGTGGAACTTTAGGCGTTACTATTGTTGGTGGAACACTTGACTGCTCAACCTTTCACGCTTCCATCACAAACCAATTAAATGATTGGTTCTTGCCAGAAGAGGCGGAGGCTATATCAGGCTCAACAAGCTGCAGTGGTAACTCTTTTACAATCAACTACAACAACATCCCCGCCGGTTATAGACCATTTTTGGACATTTTGGTTTCAGTTCCAACAGGTCAAGGATTACAAACTGCTTACACCAACACATACTTGTGTGCAGGAAGTTCATCGGTTAAGGATGGTTCAAAATCTACAACTTGGGGACCATACCAGAATAACGATGCTGGTGCTAATGGTAATGAAGTTGTTGTCACTACATCTACATGGCTT

**>*CoALS4210* GenBank Accession MG799558.1**

ATGGTTAAACATTTATCATTTGCGGCGATATTTGTTGCCTTTGCGCTCACAACGCTTACTCAAGCAGCTGAGATCAGCAATGTTTTTCAAAGCTTTGACAGTTTGACTTGGGAAAATGGGGCCAATTATCAATACAGAATACCAGCAGCTCCTAGTTGGATTGCGACTTTGTCATGGAAGATTTTGGGCTCAAATGTTCACCCGGGAGATACGTTTACATTAAATATGCCATGTGTATTCAAGTTCACAACCACACAAGAAAGCGTTGATTTAGATGTTGGGGGCACAGTTTATGCTACATGTCAGTTCAAACCAGGTGATTTAGTTGTTCCATATTCTCAATTAAAATGTACAGCAAGCAATAACGTTAAGGGCAGTACCGATGCTACTGGTACTGTGAGGTTCCCACTCACCTTCAATGTTGGTGGATCTGCTAATTCGGTTGATTTGCAAGACTCGAAATGTTTCACGCCAGGTACTAATGACGTCACATTCACTGATGGTGATAAGGAATTGACCACCACTGCAAACTTTCAAGGTGGTACCAACACTAACAGAGGTATTCCATATGATGATATCGTTTTCAACAATCGAGTAGTTCCATCTTTGAACAAACAACAATTATACTTATTGGGAGGAACATGTCCAAATGGATATAGAAGTGGAACCTTAGGAATTACAGTCAGTGGAGGTACACTTGACTGTTCGTCTCTTCACGCTGCTATAACAAATCAATTAAATGGATGGTACAACCCCGAAGTTGCAGAAGCTATTTCAGCTTCTTCAAGCTGTAATGGCCGGTCATACACAATCAACTATAACAACATTCCTGCTGGTTATAGACCTTTCATGGACATTTTAGTTGCGCGTCCGGCTGGTCAACAATTACGCACAAGTTATACTAATACGTATCAATGTGCAGGGCAGTTATTTCCGCGCGACAATTCGAGACTGATAAGATGGGGAGCTTACAACAACAATGAGGCAGGTGCTAATGGTAATGAAGTCGTCGTCACCACATCCACATGGCTT

**>*CoALS4220* GenBank Accession MG799559.1**

ATGGTTAAACATTTATCATTTGCGGCGATATTTGTTGCCTTTGCGCTCACAACACTTACTCAAGCAGCTGAGATCAGCAATGTTTTCCAAAGCTTTGACAGTTTGACTTGGGAAAATGGGGCCAATTATCAATACAGAACACCAGCAGCTCCTAGTTGGATTGCGACTTTGTCATGGAAGATTTTGGGCTCAAATGTTCACCCAGGAGATACGTTTACATTAAATATGCCATGTGTATTCAAGTTCACAACCACACAAGAAAGCGTTGATTTAGATGTTGGGGGCACAGTTTATGCTACATGTCAGTTCGAACCAGGTGATGTAGTTGTTGCATATTCTCAATTAAAATGTACAGCAAGCAACAACGTTAAAGACAGCACTGATGCTACAGGTTCCGTTAAGTTTCCATTCACCTTCAATGTTGGTGGATCAGCAGGAGATGTTGATTTGCAAGACTCGAAATGTTTTACTGCTGGTACTAATCAAGTTACATTCACTGATGGTGATAAGGAATTGACCACCACTGCAAACTTTCAAGGTGGTTCCAACACTAACAGCGGTTCAACTGATGACATCGTTTTCAACAATCGAGTAGTTCCCTCTTTGAACAAGCAGCAAATGTATTTGTTGGGAGGAACATGTCCAAATGGATATAGAAGTGGAACCTTAGGAATTACAGTCAGTGGAGGTACACTTGACTGTTCGTCTCTTCACGCTGCTATAACAAATCAATTAAATGCTTGGTACTTCCCTGAAGTAGCGGACTCTATTTCAGCTTCTTCAAGCTGTAATGGCCAATCATACACAATCAACTATAACAACATTCCTGCTGGCTACAGACCTTTCATGGACATTTTGGTTGCAGTTCCAAATGGTCAGAGATTGAAAGCAAGTTACACAAACAGATACCAATGCGCTGATGAAGTTGGTAGTCACGACAACTCCAAATCAATTACATGGTCACCTTACAGCAATAACGTGGCAGGAGCCAATGGTAATGAAGTCGTTGTTACAACATCTACATACCTA

**>*CoALS800* GenBank Accession MG799557.1**

ATGAAAAATTGGAGCTCACTTGTGGCCATTTTTACGGCTTTCACATTAACAACACTTACATTCGCTGCTGAGGTCACTGATGTTTTTCAAAGTTTTGATAGCTTGACCTGGGAAAATGGAGCTACATACCAATACAGTACACCAGCCGCTCCAAGTTGGATTGCAACTTTGTCGTGGAAGGTTTTGGGTTCAAATGTTCATGCAGGTGATACATTTACATTAAACATGCCTTGCGTTTTCAAGTTTACAACAACTCAAGACAGTGTCGACTTGAATGTTGGTGATACTGTTTATGCTACATGCCAGTTTGCTCCTGGTGATTTGGTTGTTGCTTACTCTCAATTAAAGTGTACTGCTAGCAACAATGTCAAAGATAGCACAAATGCCGCTGGTTCAGTACATTTTCCGATCGCTTTTAATGTTGGTGGATCTGCAGGAGAAGTTGATTTGCAAGACTCGAAATGTTTTACTGCTGGTACTAATCAAGTTACTTTTACTGATGGTGATAAGAAATTGACCACCACTGCAAACTTTCAAGGCGGTTCCAACACCAATGGAGGCATTTCGACTGATACAATTGTCTACAATAATCGTGTTGTCCCATCTTTGAACAAGCAGCAGTTATACTTGCTTGGAGGAACCTGTGACAATGGATACAGAAGTGGAACTTTGGGTATTACAGTCAGTGGAGGAACACTTGATTGCTCAACGCTCCATGCTTCCATCACAGATCAATTGAATGCTTGGTTTTTACCTGAAGAGGCAGAAGCAATTTCTGCTTCCACCAGTTGTAATGAAGGGTCTTATACAATTAATTACAGCAATATTCCTGCAGGTTATAGACCATTCTTGGATATTTTGGTTTCAGTTCCTACTGGTCAAAGTTTACAAACATCTTATACTAACACATACTTGTGTGCTGGAGATTCGTTTCCTAACGATGGATCAAGATCTGTTACTTGGTCACCATACAACAATAACGATGCTGGTGCCAACGGTAATGAAGTCGTTGTCACTACTTCTACATACTAT

**>*CmALS4210* GenBank Accession MH753528.1**

ATGGCGAAACATTTGTCAATCGTGGCTATTTATTTTGTCCTTGCACTCACAGCCATCACTCAAGCTGCTGAAATTACCAATGTTTTCCAAAGCTTCGATAGTTTAATCTGGGAAAATGGAGCCAATTATCGTTACAGAACACCTTCAGCACCAAGTTGGATTGCAACTTTGTCATGGAAAATCTTGGGTTCAAACGTCCATGCTGGTGACACATTCACCTTAAACATGCCTTGTGTATTCAAGTTCACCACTACTCAGCAAAGTGTTGATTTAGATGTTGGTAATACCGTTTATGCTACTTGTCAATTCTTGCCTGGTGATTTAGTTGTTCCATATTCTCAATTGAAATGTACTGTTAGTAACAACGTTAAAGCTAATACTGATGCTACTGGTACTGTGAGATTCCCAATTACTTTCAACGTTGGAGGTTCTGGTGGGTCAGTTGATTTGGAAAACTCCCAGTGTTTTACTCCAGGTACCAATGATGTGACATTTACTGATGGAGATAAGAATTTAACTACAACAGCAAACTTTCAAGGTGGTACCAACACAAACTCGGGGATTTCATATGATGACATTGTTTTCAACGATCGTGTGGTTCCTTCATTGAACAAAGAGCAATTGTACTTGTTAGGAGGTACTTGTCCAAATGGATATAGAAGTGGTACTTTGGGAATCACAGTCAACAGTGGAGGTACACTTGATTGTTCAACCATCCATTCAGCTATAACCAATCAATTGAATGCCTGGTACTTTCCAGAGGCTGCAGAAGCCATCAGTGCTTCATCAAGCTGTAATGGACGCTCTTACACAATCAACTACGGTGCCATACCTGCTGGATTCAGACCTTTCATCGATATTTTAGTTTCCCGTCCAACTGGACAACAATTGAGGACAACTTACACAAACAGATACCAATGCGCAGGCCAATTATTTCCTCGCGATAATTCAAGACTGGTGAGTTGGGGAGCATACAACAATAACGAAGCTGGTGCAAATGGTAATGAAGTCATCGTCACTACATCAACATGGCTT

**>*CmALS4220* GenBank Accession MH753512.1**

ATGATGCGAATGCATATTTGGACTCAATTGTCTATATTTCTACATATTTTTACGCTAACTACTGCTGCACAAGTTTCCGGGATTTTCAACAGTTTCAATTCACTTACTTGGAAAGCAGCTTCTACAGGGTACATCAATGCTTCGCCGAACAACCCTACTTGGACTGCTGTTTTAGGTTGGTCTTTAGACGGTTCGAAACAAGCTCCAGGTGACACTTTTACATTAATGATGCCATGTGTCTTCAAATTCATCACAAGCCAAACATCAGTCGACTTAACTGCCGATGGAGTTTCATACGCAACTTGTCAAATGAATCCGGGTGAGATTCTTGTGACATATTCAACATTGACCTGTACCGTGAAATCTGCATTACAATCGAATGTACAAGCAACAGGATCTCTTTCCATTCCACTATCATTCAATATCGGTGGATCAGGAGGTAATGCTGACGTCATAGATGCACTGTGCTATAAAGTTGGTCAAAATACAGTTACATTCACTGATGGACCAAATGCACTTTCGATAAATGTTAATTTTGGAAAACCGGATTATAAATTCACTTCTGATAAGTACACTGCCTTCAGAAGTATACCTTCATTAAATGAAGCTCAACATTACTTAGTGAGTGGTAATTGTGCCAAGGGTTATACTTCAGGTACAATTGGGCTTGCGGCAGCTGGTGGTGCCCAAATTGATTGTTCAAATTGGCACGTTGGTTACTCGAATGACTTTAATGATTGGGCATTCCCAAAAAGTTACTCTTCAGACTACACCGTAACCGGCCTGTGTCTGAGCTCTCAAGTACTTGTCAACTTTAAAAACGTTCCAGCTGGTTATAGACCATTTATTGATGCATTGTTTAGAGTACCTGATGGCCAACAAGTTAAGGTTACGTACATCAATGAAGTTCAGTGTGTTGGAAGCTCCAGCACAGATGATTATGGCGAACAAGCATATGGGTGGGGATCTTATCAAAATAGTGAAGCTGGTGCCAATGGTGTCATTGTTGTTGTTACAACTTCAACTATTCTT

**>*CmALS800* GenBank Accession MH753530.1**

ATGAAGAATTGGCACTCACTTGCAGCCATTTTAACGGCTATTTCATTCTCATCATTTTCACTCGCTGCTCAAGTTTCAAATGTCTTTCAAAGTTTCGATAGTTTAACCTGGGACAATGCTGCTGGTTACCAATATGCTACACCTGCATCTCCAAGTTGGATTGCAACCTTGTCATGGACAATTTTGGGTTCAAATGTACACGCTGGTGATACTTTCACCTTGAATATGCCATGTGTTTTCAAATTCACCACAACTCAACAAAGTGTCGATTTAACTGTTGGTGATACTGTATATGCTACCTGTCAATTTGCTCCAGGTGATTTGGTTGTTGCTTACTCACAATTGAAGTGCACTGCAAGTGATAATGTTAAAGATTCTACAGATGCTACTGGTTCAGTCCATTTCCCCATTGCGTTTAACGTTGGTGGTTCAGGAGGTGATGTTGACTTGCAGGACTCAAAGTGTTTCACTCCAGGTACCAATGATGTAACTTTTACTGACGGGGACAAGAAGTTGACAACCACAGCGGACTTTGAAGGTGGTACCAACACCAACAGAGGAACATCCTATGATGATATTGTCTTCAACGATCGTGTCGTCCCATCTTTAAACAAGCAACAAATATACTTACTTGGAGGTGTCTGTCCAAATGGCTACAGCAGTGGAACTTTGGGAGTGTCAGTCAGTGAAGGTACACTTGATTGTTCCGTCCTCCACTCTGCCATTACAAATCAATTGAACGCTTGGTATTTGCCGGAAACCGCTGAAGCTATTTCTGCTTCTACTAGTTGCACTGAACAAGCATTTATGGTCAACTACAACAACATCCCAGCTGGTTACAGACCATTCTTGGACATATTGGTCTCACTTTTACCAGGTGAAAGCTTACAAACATATTTCACAAATAGATACACTTGTGCTGGTGATTTATTTGCCCAAGATAACTCAAAATATACATACTGGGCACCATACGACAACGATGAAGCTGGTGCTAATGGTAATGAAGTCATTGTCACTACATCTACCTATTAT

**>*CmALS2265* GenBank Accession MH765692.1**

ATGGCTGGGTTATCTACCAATGTTGCATGGTTTGCAACGTTCATGTTTGTTGCACTAACCAATGCTGCACAAGTGTCAAACATTTTTCAAAGCTTTGATAATTTAGTTTTTGCTCCTGCTGCTGCTTACAATCTCGAAGTTCCTTATGCTCCTTCTTGGAATGCTACATTATCATGGGAGATTGCAGGTTCCAAGGTTAAGGCTGGTGATACATTTACGTTGAACATGCCTTGCGTCTTTAAATTTACAACATCCAATCCAAGTGTTGACTTGAAAGTTGGAAATACCGTGTTTGCTACCTGTAAGTATGCTCCTGGTGATTTGGTTGTTCCATATTCACAATTGCAATGTACCGCAGCTGATGCTGTTACTGACGGTTCAACCGCAAGTGGAAAAGTCACGTTCCCCATCACATTCAATGTTGGTGGATCAGGAAATGCAGTTGATTTACAGGACTCGAAATGTTTTACTGGTGGAGAGAACACAGTGACATTTAACGATGGTGATAAGAAGTTAAGTACTCAAGTCGATTTTGACAGTGGCTCCTATTGGAATGATGCTACTAAACAAGACCCTGCCGTTCAAGTTGCTGCTACTCGAACTGTTCCTTCTTTGAACAAACAACAGTTTTATCTACTTGGTCCCCAGTGCCAGTACGGTATAGTTAGTGGAAAATTAGGTATCAAGTCAAACGCAGGTTGGCACGATTGTTCAACAATGCATGCTGCTTTAACAAATCAATTGAATGCTTGGTATTTCCCAGAAAGTGCAGATAAATTTTCCTTCACGACAACATGTAACTCTAGGGGATTAACCGTAACTTACTCGAATGTTCCACAAGATTACCGACCATTCATTGACATTTTGGTTGAGCTCCAACAAGGAGCTAGCAATGACGTCTATTATACGAACCAGTACCAATGTAGGTACGAGAGCGAGAAAAGGGCTGACCTTGGTTACACATGGGGTACCTACAATAATTCAGAAGCTGGGTCGAATGGAAATGAAATCAAGGTAATCACATCAACATATTAT

**>*CtrALS941* GenBank Accession MH753531.1**

ATGTTGTTGCTACAACTAGTAATTACATTATTGACCTCTGTTAAAGCTGTGCTTGCAGACGAAATATCGGGTGTTTTTACCAGTTTTGATTCATTAACGTTTTCCCATCCTAGACTTACTTATACTCCACCAAATTTCCCAACATGGACAGCTGTTTTTGGTTGGGATCTTGAAGGTAGCACTGCTCGTCCAGGAGATGTGTTCACCTTGGTTATGCCATGTGTTTTCAAATTCCTTACCAGTACTCCATATGTTGAGTTGACGGCAGATGGTGTCACTTATGCAACTTGTAGATTAAATTCTGGAGAAGAATTTGTTCTGTTTTCAAGTATGGAATGTACTGTTTCTGAAAACTTGACTCCTTCATCAATTGTCTACGGACAAGTTTCTGTTCCACTTACTTTTAATGCTGGTGGATCTGGTTCAGAAACAGATATTGAAGCTTCTACATGTTTTGTTGTTGGAGAAAACACTGTGACCTTTACCGATGGTGATAATAGTCTTTCTATCCAAGTTAATTTTGAAGCAAATCCTGCAGATCCATCTGGATTACTTAGTTCTCAAAGAGTTATTCAATCACTTGCTAAATCATTAGCTTTGGTAATAATTCCAGACTGTCCCAACGGATATGCTTCCGGTACTCTTGGTATTTCTTCTACTGCGGATGGATACCAATTAGATTGTAATTCTATTGAAGCTGGCTTAACTAGTGGATTAAATGCTTGGAACAATCCTATTGATAACATTGACTTCCCACATACTTCACAATGTACTACGAAAGGTTTTAGTATATCATTTCTGAACATACCAGCAGGGTATAGACCATTTATTAATGCGCTTGCAACAGTTCCAAGTACTGAACAATATAGGGTTGCTTATGAGGTTAAATATACTTGTGTTGGTGGATCATATCGTGACGATTCGATGACAAGATTATGGAATCCATACCAAAGAAGTGAAGCTGATCTGTATGGGCAACCAATTGAAATCATC

**>*CtrALS1028* GenBank Accession MH753521.1**

ATGAAGTTTTTGGGATTAGTTTTATTATTCTTGTCCTTGATTAATCAAGTGACTCCTAAGGAAGTATCGGGAATATTCACCAGTTTTAATTCATTAACTTGGTCAGATGCCGGTAATTATGGTTATCGTGGTCCTGCTAATCCAGCTTGGCAAGCTAAATTAAGTTGGTCTTTAGAAGGTAAAAAGGTTAACCCAGGTGATACTTTTACTTTAACAATGCCTTGTGTTTTTAAATTTGTTACTACTCAACCTTCTATTGATTTGGCTGCTAATGGTATTACTTATGCTACTTGTACATTTCATTCGGGTGAAGAATTTACCACTTTTTCAACTGTTAGTTGTATTGTTAGCGATGCATTGACTTCCTCAACTCAAGCTTTTGGTACGGTCAGTATTCCATTTTCTTTCAATATTGGTGGTTCTGGTTCGGATGTTGATTTAACTGATTCAACTTGTTTCACTACTGGCTCCAATACTGTTACATTTAAAGATGGTGATAATGAACTTTCAATTCAAACAAATTTTGAACAAACTAAAGATTCACAATCTGGTTTAATCACTAATGCCAGAGTTATTCCATCACTTGGTCAATTGTCCCATCTTGTTGTTGCTCCAGATTGTCCAAATGGTTATGCCAGTGGTGAATTAGGAATCTATGCAAGAGACAATTCAGTTACTATTAATTGTGAAAATATTCACATTGGTATTACTGATAAATTAAATGCATGGAATAATCCAACAAACTCTAATGGTTTCACCTACACTAAGAAATGTGATTCCAATGGATTTTCAATTTCTTTTAAAAACGTTCCAACTGGTTATAGACCATTTTTGGATTCATTGATTAATGCTGCTACTGATTATACTTTTACTATTAGTTACATTTCCAAGTACACATGTGCTACCGGTGATTATCATGATAAATCTATTACTAAAAATTGGGCTCCATATAAAAATGGTCTTGCTGATAGTGATGGTGCTGTTGTTTTTGTT

**>*CtrALS1030* GenBank Accession MH753522.1**

ATGTTCGTTTTTAGATTATATTTACTACTATTAGTATTTCTTTCTGAAGTAACTCCAAAAGAACTATCTGATGTCTTCACCAGTTTTAATTCATTGACGTTTACAGATGCTGGATATGGATATAGAGGTCCATCGAATCCAACATGGCATGCTAAATTAAGTTGGAATTTAAATGGTGCGTATGCTCGACCTGGTGATACTTTTGGTTTAGTTTTACCACATGTATTTAAGTTTGTCACTGCTCAATCGTATTTTACTTTATCTGCAGGGGGTGTGACATATGCAATCTGTGATTTCCAACCAGGTGAGTTATTCACAACATTTTCCTCGATCAAATGTACTGTTAGTGAGAAATTGAACCCAAACATTGAAGCTTTTGGTACAATTACTTTCCCGTTTGCTTTTGGTGTTGGTGGTTCAGGATCAGATACAGACTTAGTAAATTCTAATAGTTTCACCACTGGTGAGAATAGAGTCACTTTCAAGCATGGTTCGAAAGATCTTTGTATTGATGTTGATTTTCAAGGAAGTCCTGCCAAGACGACGGATTTACTTAGTTATGGTAGAATTATTCCTTCACTAAGAAAAATATCACATCTATTAACTAGTGCTGATAGTCCAAATGGTTATAAAAGTGGCAAATTAGGACTCGCGTCATCTGACGCAGGCCTCGGCATTGATTGTGATTCTGTTCATGTTGGTATTACCAATATGTTGAATCCTTGGAATCAACCAATGAATGCAGAATCGTTTTCTTATACTACACAATGTTCTGAAGAAGAAATTATGATAACTTTCAATGAAGTTCCAGAAGGGTATCGCCCATTTTTTGATGTTTTGTTTTCTCATACTGCCAGTGATATTTTTACCATGTTGTACACAAATGAATATGTTGGGGCTGATGGGGTTACATATGATGCTTCGATGAAAAAAGCTTGGAAATCCTATCAAGATAGTCTTCCACTGGGTGATGGCGCCATTATTATTGTT

**>*CtrALS1038* GenBank Accession MK128125.1**

ATGAAGCTCATTGGATTTGGTTTGCTATTACTGTCTTTGGCTACTTTGGTGGCACCGAAGGAAATTTCTGGTGTTTTCACTTCCTTTGATTCATTAAAATGGAACGAAGATCTGAATGATTTTAGAGGTCCAGCTAGTCCTACTTGGAAAGCAACATTGGGATGGTCTTTGGATGGTACTAAGGTCAATCCAGGTGATACATTCACCTTGATTATGCCTTGTGTTTTTAAATTTATCACTGAACAGACTACTATTGATTTAACAGCAAACGGTGTGAACTATGCTACATGTACCTTTCATGCTGGTGAAGAATTTACTGCATTTTCAAGTGTTGGATGTGTTGTTAAGGACGCTTTGAAATCTAACATCCAGGCCTTTGGTACTGTTACAATTCCATTTACTTTCAATGTTGGTGGCACTGGTACTTCTGTCAGTTTGCAAGATTCTACTTGTTTTACTACTGGTGATAATATTGTCACTTTCCAAGATGGTGATAACAAACTTTCGATTACTGCCAATTTCGAACCTACAGGTGCTTCTAGAAGCGATTTAATTGCCAATGCCAGATCAATTCCATCACTTGAGAAAATGACCCACCTTGTCATTGCTCCTGATTGCCCAAGTGGTTTTAAAAGTGGTACTATATCTTTGAATACAAATAATGGTGCTGATATTGACTGTGCCCAGGTACATGTTGGTATGACTAATTTTATTAATCCGTGGAACTACCCAACTAATTCCGAACAGAATTTCTCAAAACAACCAACCTGTAATGAAGGAAGTTTCACTCTTTCATTTGAAAGTGTTCCTGCCGGTTTCAGACCATTCTTTGATGTTATGGTTACTCCTAAAGGGAAAATGGGTTTTGACTACAAGTATAGTGTTGTCTGTGCAGATGGTGAATCTCTTGAGCATCCTACTCACTATGACTGGGGTACTTATAACACTCAAACAGCCGATAGTAATGGGGCTATTTTAGTTATC

**>*CtrALS1041* GenBank Accession MK128127.1**

ATGAGTTATTTTGGATTTGCTATACTATTGTTGGCCTTGTTCACAAGAGTAACCCCTAAAGAAATCACGGGGATATTCACCAGTTTTGACTCGTTAACTTATTCTGATGCAGGTAATTATGGATACCAAGGTCCAGGGAATCCAACATGGACTGCAACTTTAGGTTGGTCATTAGATGGTTCTGTTGCCTCTCCTGGTGATACATTCACCTTAATTATGCCTTGTGTTTTCAAATTTACTAGTTCATCAACATCAGTGGACTTAACTGCTGATGGTGTAACTTATGCTACTTGTAAATTGAATAATGGAGAAGAATTTACCACATTTTCTAGTATGTCTTGTGTTGTTAATAGTGCTTTAACTTCTGATACTCAAGCATTTGGAACTGTCACTGTACCATTCTCGTTTAATGTTGGTGGTACTGGTTCTTCTGTTGATTTAGAGGACTCCACTTGTTTTACTGCTGGTACCAATACTGTTACTTTTAAAGATGGTGATAATGAACTCTCGATCAATGCCGTTTTTGAGAAAACCACTGCTTCAGTATCAGATGAAATTATTTTTGTCAGATCAGTTCCTTCCATTGGTAAATTGCAACAGATTTCTATTGCAAAAGATTGTCCAAGTGGGTATGAAAGTGGTTATATGAGTATAATTATTAGAGATAATTCTGCTGTTATGGATTGTTCTTCAACTCATATTGGTATTACTAATGATCTTAATGATTGGAATCAACCAACAAATTCTGAAACATTTTCTTATACTGAGAGCTGTTCTGCTACAAACTTTACTATTTCATTCACTGATATCGAAGCTGGTTATAGACCATTTATGGATTCATTCCTCACTGCAACCGCCAATGCAAGATTTAATGTTGACTATATTTACAAGTATACTTGTAAAAATGGTGACACGGTTGATAAAACTAATAGTAGAGTTTATGCTCCTTATATCAACAGTAATACTGATAGTAATGGGGCTATTTTAGTTATC

**>*CtrALS2228* GenBank Accession MK128126.1**

ATGGTTCTCATACAAGCCATTGTGCTATTAATATGGATTCAATTGGTATCTTCAAAAGAGATATCTGGGATTTTTATTGGTTTCGATTCATTAACATGGAATGCTGCTTCGGATTTACCCAGTGCATACCAAGGTCCACAAATTCCTACCTGGACTGCAGAACTAACATGGTTTTTGAATGGAGAATCTGCTGAACCAGGTGATACATTCACATTAATAATGCCATGTGTATTCAAGTTCATTACAAATCAAAACACTGTTGATTTGATTGCTGATGGCACTACTTATGCGACCTGTAATCTCAATTCTGGAGAAGAATTTACTACTTTCTCAAGTTTAAGCTGTACTGTTTCATCTACCTTGACAACATATACCCAAGCTCGTGGTACTTTACATGTTCCTTTGACATTTAATGTGGGTAGTTCGGGAACTTCTGTTTCGTTGACTGATTCTACTTGCTTCAGACCAGGTGTAAACACAGTAACTTTCAGAGATGGAGACAATGAAATTTCAACACAAGCAACTTTTCAAGGTAGTCCAGATGACCCATCACCAGACCGTTATTATCAAAGAGTTATTCCATCCCTTAACAAAGTGTCAAATCTAGTCTATCCTCCTAATTGTCCAAATGGGTATTCTTCCGGTGTTATCAGCTTCTCCTCGAGTGATTCTAATTTCCAACTTGACTGTTCCTCACTTGATGTTGGTATGACAAATCAATTAAATGCATGGAATTTCCCTACTAGTAGAGATTCTTTGTCTTATACAAGGAGTTGTAGTGACAAAAGTATTACTGTTAACTTTCAGAATGTTCCTGCGGGTTCGCGTCCCTACATTAGTGTCCTATCTGCTTTTCTTGGTACCAAAACCTATTCAATTAATTACAATTTGAGATACACTTGCAGTGGTAGTAGATCAAGTTCTGTGACAAAAACTATAAGCTGGGCTCCATATAGAAATAGTTTGGCCGATTCCAATGGGGCAGTGGTCGTG

**>*CtrALS2229* GenBank Accession MH753523.1**

ATGTTGCGTCTATCAATTACTTATTCCATTTTCACATTAATTAACGTTATAAATGCCAAGGTCCTCTCCGGCATATTCACCAGATTTGTTTCATTAACACAATCATCTTATAATTCGTACAGTTTCGACGGTCCAATGCTGACAACTTGGATTGCAGCTCTAGGGTGGGAAATCAATGGAACAAAAGCAAAACCAGGTGATACATTTACTTTAGAGATGCCCTGTGTTTATAAAATTTTTATAAATGAAGAATCTATTGACTTAATTGGAAATGATATTAGTTTAGCAACTTGTGAAGTCCACTCTGGTGAACATATATCCACAAATTCGTATTTGAATTGTGTTATGAATGATTCTTTAGACGAAAGAACCAATATTGATGGAATTTTGAGACTCCCTATTATGTTCAATGTTGGAGGTTCCGGTCAAGATACTGACTTAGATGCGGCATCATCGTGTTTCAATACTGATTCCAACATAATTGCTTTCAACAATGGCGACACAAGTATTTCCATTCAACGAAGCTTTTCAGTAAGAGCAATTTTTGATACTGTACCAATAAACTTTGTTCGGGTAGGAAAAACTATTAGTGAATTAGAGGTACTCATTGTTGCTCCAAATTGTCCACAAGGTTACACAAATGGTAGACTAGCAATTGCTGCTTCAGACCGTGACGTTATCATAAAATGCTCTACTATAACATCGGGATTTGCCAGTAAACTTAACAAGTGGAACCTTCCAGAAAATTTAGTACAACTTTACCACGGCAGTCTTTGTACTTCACGTCAATTTTCTATTAGTTATACTAATATTCCAGAAGGGTATCGTCCATTTCTACTGGTAACTTTAAGCAATGCTGTTAGTTCTTCATTTAACCTTCGTTACACGATTCAGAATACATGTGAAAAAGACACGTTTAATGATCAATCAAGATCAGTTTCCTGGAGAAAGTTAAACTATGGATCTATCGATTCATATGGTGGTATCATGATTCCCGTC

**>*CtrALS2293* GenBank Accession MK182724.1**

ATGTTTCTATTACAAGCAGTAATTTTATACTGTTCATTCATTGTTACTGCGGTTGCTAAAGAAATTTCTGGTGTATTTACTGGTTTTGAATCCTTAACGTGGGATAAAGCTGCTAATTACGGCTTTCAGGGTCCACAATATCCAACATGGAATGCCGTTCTTGATTGGTCGTTAGATGGTACAACCACGTCCCCAGGTGACACGTTCACTTTGATCATGCCTTGTGTCTTCAAATTCACTACATCTGCCACTTCTATCGATTTAAGAGCTAATGGTATTACATATGCCACATGTGATCTTCATGCCGGTGAAGAGTTTACTACTTACTCCAGTTTGACTTGTACTGTTACCGATTCGTTAAGTTCTGTCCATGAAGCATCGGGTACAGTCACAATTCCTTTGGCATTTAACGTTGGTGGTTCTGGTTCTTCTGTTGATATTGCTGATTCTACTTGTTTCACTGCTGGCACAAACACTGTTACTTTCCAAGATGGTGATACATCAATCAGTACCCAAGCCTATTTTGCTGCAGCTACTGAATCTTCTTCTGGTCTCCTTTACTTCCAAAGAAGTGTTCCTTCATTGAACAAGCTTAATGCACTTGCAATTCTCCCAGATTGTCCCAATGGTTACACTTCTGGTACTCTTGGTTTCTCATCTTCTAATTCCAGATTTCTGATTGATTGTTCCAGTGCTGAAGCTTACATTACAAATCTTTTAAATTCCTGGAACTACCCAACTTCAGCGGATTCCTTTTCTTATACCCAAACTTGTACTTCCAAAAGTTTTCAAATAACATTCAATAATATTCCAGCTGGTTATCGTCCATATATTGCTGCATTGGTTCAAGCTCCATCTTCGGATTATGCTATACAATATACTGCAAAATACAGGTGTGAAGGTTCTGTCCAAAGAGATGATTCACAAAAGATATCTTGGGCCGGTTACACAAATAGTGACCCAGATTCAAATGGTGCTGTAGTTGTT

**>*CtrALS3786* GenBank Accession MK332912.1**

ATGATTTTTTCTGAGTTTTTAATATTGTCGCTAACATTCATTGCTACTTCAGTTGCTAAAGAAATTTCCGGTGTGTTCATAGGATTTGAATCCTTAACTTGGGATAAAGCTGGTGATTATGCTTATCAAGGTCCTCAATATCCAACATGGAATGCCGTGCTTGATTGGTCGTTAGATGGCACAACAACTTCCCCAGGTGATACGTTCACTTTGATCATGCCCTGTGTCTTCAAGTTCACTACGTCTGCCACTTCTGTTGATTTGACTGCCAATGGTATTACATATGCCACATGTGATCTTCATGCCGGTGAAGAGTTTACTACTTACTCTAGTTTGACTTGTACTGTTACTGATTCGTTAAGTTCTGTTCATGAAGCTATGGGTACAGTCACAATTCCTTTGGCATTTAACGTTGGTGGTTCTGGTTCTTCTGTTGATATTGCTGATTCTACTTGTTTCACTGCTGGCACAAACACTGTTACTTTCCAAGATGGTGATACATCAATCAGTACCCAAGCCTATTTTGCTGCAGCTACTGGATCTTCTTCTGATCTCCTTTACTTCCAAAGAGTTGTTCCTTCATTGAACAAGCTTAATGCACTTGTAATTCTCCCAGATTGTCCAAATGGTTACACTTCTGGTACTCTTGGTTTCTCATCTTCCAATTCCAGATTTCTGATTGATTGTTCCAGTGCTGAAGCTTACATTACAAATCTTTTAAATTCCTGGAACAAGCCAACTACGGCTGATTCTTTTACATATACCCAAACTTGTACTTCCAAAAGTTTTCAAATAACATTCAATAATATTCCAGCTGGTTATCGTCCATATATTGCTGCATTGGTTCAAGCTCCATCTTCGGATTATGCTATACAATATACTGCAAAATATCAATGTACTGGATCTACCCAAAAAGATATTACGAAATCGGTCACTTGGTCAGGCTATACAAATAGTGACACAGATTCAAATGGTGCTGTAGTTGTT

**>*CtrALS3791* GenBank Accession MK170233.1**

ATGTTTCTACTACAAACAACTCTCTTATGCTGTGCATTGATTGCTACTTCAGTTGCTAAAGAAATTTCCGGTGTGTTCATAGGATTTGAATCCTTAACTTGGGATAAAGCTGGTAATTATGCTTATCAAGGTCCTCAATATCCAACATGGAATGCCGTGCTTGATTGGTCGTTAGATGGCACAACAACTTCCCCAGGTGATACGTTCACTTTGATCATGCCCTGTGTCTTCAAGTTCACTACGTCTGCCACTTCTGTTGATTTGACTGCCAATGGTATTACATATGCCACATGTGATCTTCATGCCGGTGAAGAGTTTACTACTTACTCTAGTTTGACTTGTACTGTTACTGATTCGTTAAGTTCTGTTCATGAAGCTATGGGTACAGTCACAATTCCTTTGGCATTTAACGTTGGTGGTTCTGGTTCTTCTGTTGATATTGCTGATTCTACTTGTTTCACTGCTGGCACAAACACTGTTACTTTCCAAGATGGTGATACATCAATCAGTACCCAAGCCTATTTTGCTGCAGCTACTGGATCTTCTTCTGGTCTCCTTTACTTTCAAAGAAGTGTTCCTTCATTGAACAAGCTTAATGCACTTGCAATTCTCCCAGATTGTCCAAATGGTTACACTTCTGGTACTCTTGGTTTCTCATCTTCTAATTCCAGATTTCTGATTGATTGTTCCAGTGCTGAAGCTTACATTACAAATCTTTTAAATTCCTGGAACTACCCAACTTCAGCGGATTCCTTTTCTTATACCCAAACTTGTACTTCCAAAAGTTTTCAAATAACATTCAATAATATTCCAGCCGGCTATCGTCCATATATTGCTGCATTGGTTCAAGCTCCATCTTCGGATTATAAAATTGATTATACTGCAAAATATCAATGTGCTGGATCTTCCCAAAAAGATGCTTCGAAATCGGTCACTTGGTCAGGCTATACAAATAGTGACACAGATTCAAATGGTGCTGTAGTTGTT

**>*CtrALS3797* GenBank Accession MN224675.1**

ATGAATTTCGTACTGTTATTGTTTACATTGCTCCTTTTAGTCACTCGAGCAACGTCAAAAACACTTACTGGAGTTTTCCAAAGTTTTAATTCGTTGACTTGGGAGAAAGCAGCTCTGTATAAATACAGAGGACCGCAATTTCCGACATGGAACGCTGCTGTTAATTGGGCATTGGATTCCAATGCTAATGCCGGTGATACATTTACCTTAATTATGCCATGTGTTTTTAAATTTACTACTAGTGAAACTTCTATTGATTTAACTGTGGGTAGTAAATCCTATGCTACTTGTAATTTCAATGCTGGGGAACATTTTACCACTTTTTCTAGTTTGAGTTGTACTGTGACACAGAGTGTTCCTGATAATACCAATGCATATGGTACAATCACTGTTCCACTTGCCTTTAATGTTGGGGGTTCTGGTCGTGATGTCGATACTACTGATGCAAAGTGTTTTACTACAGGTGACAATACTGTTACATTTAGTGATGGTGATAAATCATTCTCAACTACAGCAAATTTTGAAGGTGCTGGTACTTTGAATGATGATTATGAATCTTCAAGACTCATTCCTTCACTTGGTAAAACTGATGCTTTGTTGGTTGCACCATTGTGTTCCAATGGGTATAAATCAGGTACTATTGGGTTTTCTTCGAAAGCAAGCGGTTATTCAATTGATTGTAACAATATTCAAGCTGGTATTACTAGTCAATTGAATGCATGGGGTTTTCCAACGGACCTGCAAAGCTTTTCATACACCACTCAATGTACTACCACTAGTTATTCCATAACTTTTAGTACTATTCCAAAAGGTTTACGTCCATTCATTGATGCCTATATTAAAACACCTACTTCCACATATGCGGTGACATACACTTTCAAATATGTTTGTGCCGATGGAAAATCATATAATAGTAATCGAAGTTTGAATTGGTCCGGATATGTTAACGGTGATGCAGATTCTGAAGGTATGGAAATTGTTGTTGCT

**>*CtrALS3871* GenBank Accession MH753524**

ATGAACCTAATTACATTTATTTTGTTATTGTCGTCCCTCATCACAATAGTAACACCCAAAGAAATCACTGGAGTATTTACAAGTTTTAATTCCTTAACTTATAATGATGCAGCTAATTATGGTGCTCAATGCCCGGGATATCCAACATGGATTGCAACTTTAGGTTGGTCTTTAGATGGTTCTGTTGCCTCTCCTGGTGACTCATTCACCTTGATTATGCCTTGTGTTTTCAAGTTTACTAGTTCAGAAACATCAGTGGACTTAACTGTTGATGGAGTAAGTTATGCTACTTGTAATTTGAATAACGGGGAAGAATTTACTACATTTTCTAGTATGTCTTGTGTTGTTAGTAGTGCTTTAACTTCTACTACTCAAGCTTTAGGTGCTGTTAGTATTCCATTCTCATTTAATGTTGGTGGTTCTGGTTCGTCCGTTGATTTAGAAGATGCCACTTGTTTTACTGCTGGTACCAATACTGTTACTTTCAAAGATGGTGATAACGAACTCTCGATCAATGCCGTTTTTGACAAAACCACTGCTTCAGTATCAGATGAAATTATTTCAGTTAGATCTGTTCCGTCGATTGGAAAATTGCAACAAATATCTATTGCAAAAGATTGTCCAAGTGGCTATGGTAGTGGATATATGAGTATAATTATTAAAGATAATACTGCTGTTATGGATTGCTCTTCAGTTCATATTGGTATTACTAATGAACTCAATGATTGGAATCAACCAATGAATTCTGAATCATTTTCTTACACTAAAAGCTGTTCAGCAACTGAGTTTATTGTTTCATTCACTGATATTGCAGCTGGTTATAGACCATTCATGGACTCATTCCTTACAACAACTGGCAATGCAAAATTAACTGTTGATTATCATTACGAGTATACTTGTAAAAATGGTGATACGGTTGCTGAAACTGACAGAAGAATTTTCAGTCCTTATACGAACAGTAATACTGGCTGTAGCGGTGTTGTTTTGGTTATA

**>*CtrALS3882-1* GenBank Accession MH753525**

ATGAAGTTTATTACATTTGGTTTGTTATTGCTGTCTTTGCTTACCTTGGTGACACCGAAAGAAGTTACTGGTATTTTCACTTCTTTTGATTCATTAACATGGAACGAACAAACTACCCCTTTTAGTAGTCCTGCTAGTCCAACCTGGAGAGCAACTTTAGGTTGGTCATTGGATGGTACTAAACTCAACCAAGGTGATACTTTCACTTTAACAATGCCTTGTGTCTTCAAGTTCATCACTGATCAAACTACCATTGATTTAATGGCAAATGGTGTAAGCTATGCTACCTGTACTTTTCATGCTGGTGAAGAATTTACCACTTTCTCAACTGTTGCCTGTGTCGTCAATGACGCTTTGAAATCAAATATGCAAGTTACTGGTTCTGTTACAATTCCATTTACTTTTAATGTTGGTGGTACTGGTACTTCAGTTAGTTTAGAGGATTCCACTTGCTATACCGCTGGTAAGAACACTGTTATTTTCAAGGATGGTGATAACGAGCTTTCAACCATAGCAAACTTTGAACCTACAGATGCATCCAGAACTGAATTAATTACTAATGCTAGATCGATTCCTTCAATTAAAAGAACCAGTCATGTTATTATTGCTCCAGATTGTCCTAGCGGTTACAAGAGTGGTACTATAACTTTTGACACAAATAATGGTGCTGACATTGACTGTGGTCAGACTCACGTTGGTATGACGAATTTTATCAATCCATGGAACTACCCAACTAATTCAGAACAAAATTTTTCAAAGCAACCAACATGTACTAAAGGAAAATATACTCTTTCATTTCAAGATGTTCCTGCTGGATATAGACCATTCTTCGATGTTTTGGTGAAGCCTACTGGTAAAATGACGTTTTATTACAACTCGGATCTTGTCTGTGCCGACGGTACAACCTATAAAAAGGGTCTTGCATGGGATTGGGGTTCGTATCAGAATGATGTTGCTGATAGCAGTGGTGATGTTATAGTAATA

**>*CtrALS3882-2* GenBank Accession MN893367**

ATGAAGTTTATTACAATTGGTTTGTTAGTGTCGGCCCTATTCACAAAAGTAACCCCTAAAGAAATCACAGGGGTATTCACCAGTTTTAATTCCTTAACTTATTTTGATGCAGGTAATTATGGATACCAAGGTCCAGGGAATCCAACATGGACTTCAACTTTAGGTTGGTCATTAGATGGTTCTGTTGCCTCTCCTGGTGATACATTCACCTTGATTATGCCTTGTGTTTTCAAATTTACTAGTTCATCAACATCAGTGGACTTAACTGTTGATGGAGTAAGTTATGCTACTTGTAATTTGAATAACGGTGAAGAATTTACCACATTTTCAAGTATGTCATGTGTTGTTAGTAGTGCTTTAACTTCTACTACTCAAGCTCTTGGTACTGTTACTGTTCCATTCGCATTTAATATTGGTGGTTCTGGTTCGTCCGTTGATTTAGAAGATGCCACTTGTTTCACTTCTGGTACCAATACTGTTACTTTCAAAGATGGTGATAACGAACTCTCGATCAATGCCGTTTTTGACAAAACCACTGCTTCAGTATCAGATGAAATTATTTCAGTTAGATCTGTTCCGTCGATTGGAAAATTGCAACAACTCTCTATTGCAAAAGATTGTCCAAGTGGCTATGGTAGTGGATATATGAGTATAATTATTAAAGATAATACTGCTGTTATGGATTGCTCTTCAGTTCATATTGGTATTACTAATGAATTAAATGATTGGAATCAACCAATGAATTCTGAATCATTTTCTTACACTAAAAGCTGTTCAGCAACTGAGTTTATTGTTTCATTCACTGATATTGCAGCTGGTTATAGACCATTCATGGACTCATTCCTTACTACTACTGCCAATGCTGGATTTACTGTTGATTATCATTACGAGTATACTTGTAAAAATGGTGACACGATTACTAAAACTAACAGTAGAGTTTACAGTCCTTATATTAATGGTAATACTGATAGTAACGGTGCTATTTTGGTTATA
