# Supplementary material for: Pursuing Advances in DNA Sequencing Technology to Solve a Complex Genomic Jigsaw Puzzle: The Agglutinin-Like Sequence (ALS) Genes of Candida tropicalis
Source: Front Microbiol. 2021 Jan 20;11:594531. doi: 10.3389/fmicb.2020.594531 (PMC7856822; doi:10.3389/fmicb.2020.594531)
Supplement: Supplementary file 1 [file Data_Sheet_1.zip › SupplementaryFileS3.docx]

**SUPPLEMENTARY FILE S3 |** Nucleotide sequences of the 5’ domain of *CtrALS* genes amplified from genomic DNA from seven different *C. tropicalis* isolates.

Primers for amplification and DNA sequencing of each fragment were included at the top of the entry for each *ALS* gene below (see **Supplementary Table S1**). Real-time assay primers and probes were highlighted in each sequence (forward primer = green; reverse primer = pink; probe = brown). Heterozygous nucleotides were highlighted in blue (R=A/G; Y=C/T; W=A/T; S=G/C; M=A/C; K=G/T). Mismatched nucleotides within primers and probes were highlighted in yellow. Sequences could not be deposited in GenBank because they had heterozygous nucleotides due to the diploid nature of *C. tropicalis*.

***CtrALS941* (Amplified with primers Ctr941NT-F and Ctr941NT-R)**

**>*CtrALS941*_MYA-3404**

ATGTTGTTGCTACAACTAGTAATTACATTATTGACCTCTGTTAAAGCTGTGCTTGCAGACGAAATATCGGGTGTTTTTACCAGTTTTGATTCATTAACGTTTTCCCATCCTAGACTTACTTATACTCCACCAAATTTCCCAACATGGACAGCTGTTTTTGGTTGGGATCTTGAAGGTAGCACTGCTCGTCCAGGAGATGTGTTCACCTTGGTTATGCCATGTGTTTTCAAATTCCTTACCAGTACTCCATATGTTGAGTTGACGGCAGATGGTGTCACTTATGCAACTTGTAGATTAAATTCTGGAGAAGAATTTGTTCTGTTTTCAAGTATGGAATGTACTGTTTCTGAAAACTTGACTCCTTCATCAATTGTCTACGGACAAGTTTCTGTTCCACTTACTTTTAATGCTGGTGGATCTGGTTCAGAAACAGATATTGAAGCTTCTACATGTTTTGTTGTTGGAGAAAACACTGTGACCTTTACCGATGGTGATAATAGTCTTTCTATCCAAGTTAATTTTGAAGCAAATCCTGCAGATCCATCTGGATTACTTAGTTCTCAAAGAGTTATTCAATCACTTGCTAAATCATTAGCTTTGGTAATAATTCCAGACTGTCCCAACGGATATGCTTCCGGTACTCTTGGTATTTCTTCTACTGCRGATGGATACCAATTAGATTGTAATTCTATTGAAGCTGGCTTAACTAGTGGATTAAATGCTTGGAACAATCCTATTGATAACATTGACTTCCCACATACTTCACAATGTACTACGAAAGGTTTTAGTATATCATTTCTGAACATACCAGCAGGGTATAGACCATTTATTAATGCGCTTGCAACAGTTCCAAGTACTGAACAATATAGGGTTGCTTATGAGGTTAAATATACTTGTGTTGGTGGATCATATCGTGACGATTCGATGACAAGATTATGGAATCCATACCAAAGAAGTGAAGCTGATCTGTATGGGCAACCAATTGAAATCATCACTAGAACAGTTACAGAAGCAACTACCTACGTTACAACTTTACCATTTGATTCAGCTACCCAAAGAACAAGAACCATTGAAATTGTTAAACAAATGCCTTTAACAACAATAACAGGCTCATATGTGGGTGTGACAACAAGAAGTACAACACTTCCTTTTGTACTTGGAGARACTGCTACTGTATTGGTGGAATCGCCATACCATATTACTACAACAGTCACAACTTTTTGGGATGAAGAATACACTTCTACTTTGACAGTTATTGATCCATCAGAATCTATTGATACTGTCGTTGTAAATCATCCAGCTAATCCAACTACCACACTTACTAGTCTTTGGGTTGAAACTTATACTACAACTTTGACAGAAACCCATGGAGTAGGTGAAGC

**>*CtrALS941*_951**

ATGTTGTTGCTACAACTAGTAATTACATTATTGACCTCTGTTAAAGCTGTGCTTGCAGACGAAATATCGGGTGTTTTTACCAGTTTTGATTCATTAACGTTTTCCCATCCTAGACTTACTTATACTCCACCAAATTTCCCAACATGGACAGCTGTTTTTGGTTGGGATCTTGAAGGTAGCACTGCTCGTCCAGGAGATGTGTTCACCTTGGTTATGCCATGTGTTTTCAAATTCCTTACCAGTACTCCATATGTTGAGTTGACGGCAGATGGTGTCACTTATGCAACTTGTAGATTAAATTCTGGAGAAGAATTTGTTCTGTTTTCAAGTATGGAATGTACTGTTTCTGAAAACTTGACTCCTTCATCAATTGTCTACGGACAAGTTTCTGTTCCACTTACTTTTAATGCTGGTGGATCTGGTTCAGAAACAGATATTGAAGCTTCTACATGTTTTGTTGTTGGAGAAAACACTGTGACCTTTACCGATGGTGATAATAGTCTTTCTATCCAAGTTAATTTTGAAGCAAATCCTGCAGATCCATCTGGATTACTTAGTTCTCAAAGAGTTATTCAATCACTTGCTAAATCATTAGCTTTGGTAATAATTCCAGACTGTCCCAACGGATATGCTTCCGGTACTCTTGGTATTTCTTCTACTGCGGATGGATACCAATTAGATTGTAATTCTATTGAAGCTGGCTTAACTAGTGGATTAAATGCTTGGAACAATCCTATTGATAACATTGACTTCCCACATACTTCACAATGTACTACGAAAGGTTTTAGTATATCATTTCTGAACATACCAGCAGGGTATAGACCATTTATTAATGCGCTTGCAACAGTTCCAAGTACTGAACAATATAGGGTTGCTTATGAGGTTAAATATACTTGTGTTGGTGGATCATATCGTGACGATTCGATGACAAGATTATGGAATCCATACCAAAGAAGTGAAGCTGATCTGTATGGGCAACCAATTGAAATCATCACTAGAACAGTTACAGAAGCAACTACCTACGTTACAACTTTACCATTTGATTCAGCTACCCAAAGAACAAGAACCATTGAAATTGTTAAACAAATGCCTTTAACAACAATAACAGGCTCATATGTGGGTGTGACAACAAGAAGTACAACACTTCCTTTTGTACTTGGAGAGACTGCTACTGTATTGGTGGAATCGCCATACCATATTACTACAACAGTCACAACTTTTTGGGATGAAGAATACACTTCTACTTTGACAGTTATTGATCCATCAGAATCTATTGATACTGTCGTTGTAAATCATCCAGCTAATCCAACTACCACACTTACTAGTCTTTGGGTTGAAACTTATACTACAACTTTGACAGAAACCCATGGAGTAGGTGAAGC

**>*CtrALS941*_952**

ATGTTGTTGCTACAACTAGTAATTACATTATTGACCTCTGTTAAAGCTGTGCTTGCAGACGAAATATCGGGTGTTTTTACCAGTTTTGATTCATTAACGTTTTCCCATCCTAGACTTACTTATACTCCACCAAATTTCCCAACATGGACAGCTGTTTTTGGTTGGGATCTTGAAGGTAGCACTGCTCGTCCAGGAGATGTGTTCACCTTGGTTATGCCATGTGTTTTCAAATTCCTTACCAGTACTCCATATGTTGAGTTGACGGCAGATGGTGTCACTTATGCAACTTGTAGATTAAATTCTGGAGAAGAATTTGTTCTGTTTTCAAGTATGGAATGTACTGTTTCTGAAAACTTGACTCCTTCATCAATTGTCTACGGACAAGTTTCTGTTCCACTTACTTTTAATGCTGGTGGATCTGGTTCAGAAACAGATATTGAAGCTTCTACATGTTTTGTTGTTGGAGAAAACACTGTGACCTTTACCGATGGTGATAATAGTCTTTCTATCCAAGTTAATTTTGAAGCAAATCCTGCAGATCCATCTGGATTACTTAGTTCTCAAAGAGTTATTCAATCACTTGCTAAATCATTAGCTTTGGTAATAATTCCAGACTGTCCCAACGGATATGCTTCCGGTACTCTTGGTATTTCTTCTACTGCGGATGGATACCAATTAGATTGTAATTCTATTGAAGCTGGCTTAACTAGTGGATTAAATGCTTGGAACAATCCTATTGATAACATTGACTTCCCACATACTTCACAATGTACTACGAAAGGTTTTAGTATATCATTTCTGAACATACCAGCAGGGTATAGACCATTTATTAATGCGCTTGCAACAGTTCCAAGTACTGAACAATATAGGGTTGCTTATGAGGTTAAATATACTTGTGTTGGTGGATCATATCGTGACGATTCGATGACAAGATTATGGAATCCATACCAAAGAAGTGAAGCTGATCTGTATGGGCAACCAATTGAAATCATCACTAGAACAGTTACAGAAGCAACTACCTACGTTACAACTTTACCATTTGATTCAGCTACCCAAAGAACAAGAACCATTGAAATTGTTAAACAAATGCCTTTAACAACAATAACAGGCTCATATGTGGGTGTGACAACAAGAAGTACAACACTTCCTTTTGTACTTGGAGAGACTGCTACTGTATTGGTGGAATCGCCATACCATATTACTACAACAGTCACAACTTTTTGGGATGAAGAATACACTTCTACTTTGACAGTTATTGATCCATCAGAATCTATTGATACTGTCGTTGTAAATCATCCAGCTAATCCAACTACCACACTTACTAGTCTTTGGGTTGAAACTTATACTACAACTTTGACAGAAACCCATGGAGTAGGTGAAGC

**>*CtrALS941*_1019**

ATGTTGTTGCTACAACTAGTAATTACATTATTGACCTCTGTTAAAGCTGTGCTTGCAGACGAAATATCGGGTGTTTTTACCAGTTTTGATTCATTAACGTTTTCCCATCCTAGACTTACTTATACTCCACCAAATTTCCCAACATGGACAGCTGTTTTTGGTTGGGATCTTGAAGGTAGCACTGCTCGTCCAGGAGATGTGTTCACCTTGGTTATGCCATGTGTTTTCAAATTCCTTACCAGTACTCCATATGTTGAGTTGACGGCAGATGGTGTCACTTATGCAACTTGTAGATTAAATTCTGGAGAAGAATTTGTTCTGTTTTCAAGTATGGAATGTACTGTTTCTGAAAACTTGACTCCTTCATCAATTGTCTACGGACAAGTTTCTGTTCCACTTACTTTTAATGCTGGTGGATCTGGTTCAGAAACAGATATTGAAGCTTCTACATGTTTTGTTGTTGGAGAAAACACTGTGACCTTTACCGATGGTGATAATAGTCTTTCTATCCAAGTTAATTTTGAAGCAAATCCTGCAGATCCATCTGGATTACTTAGTTCTCAAAGAGTTATTCAATCACTTGCTAAATCATTAGCTTTGGTAATAATTCCAGACTGTCCCAACGGATATGCTTCCGGTACTCTTGGTATTTCTTCTACTGCRGATGGATACCAATTAGATTGTAATTCTATTGAAGCTGGCTTAACTAGTGGATTAAATGCTTGGAACAATCCTATTGATAACATTGACTTCCCACATACTTCACAATGTACTACGAAAGGTTTTAGTATATCATTTCTGAACATACCAGCAGGGTATAGACCATTTATTAATGCGCTTGCAACAGTTCCAAGTACTGAACAATATAGGGTTGCTTATGAGGTTAAATATACTTGTGTTGGTGGATCATATCGTGACGATTCGATGACAAGATTATGGAATCCATACCAAAGAAGYGAAGCTGATCTGTATGGGCAACCAATTGAAATCATCACTAGAACAGTTACAGAAGCAACTACCTACGTTACAACTTTACCATTTGATTCAGCTACCCAAAGAACAAGAACCATTGAAATTGTTAAACAAATGCCTTTAACAACAATAACAGGCTCATATGTGGGTGTGACAACAAGAAGTACAACACTTCCTTTTGTACTTGGAGAGACTGCTACTGTATTGGTGGAATCGCCATACCATATTACTACAACAGTCACAACTTTTTGGGATGAAGAATACACTTCTACTTTGACAGTTATTGATCCATCAGAATCTATTGATACTGTCGTTGTAAATCATCCAGCTAATCCAACTACCACACTTACTAGTCTTTGGGTTGAAACTTATACTACAACTTTGACAGAAACCCATGGAGTAGGTGAAGC

**>*CtrALS941*_1020**

ATGTTGTTGCTACAACTAGTAATTACATTATTGACCTCTGTTAAAGCTGTGCTTGCAGACGAAATATCGGGTGTTTTTACCAGTTTTGATTCATTAACGTTTTCCCATCCTAGACTTACTTATACTCCACCAAATTTCCCAACATGGACAGCTGTTTTTGGTTGGGATCTTGAAGGTAGCACTGCTCGTCCAGGAGATGTGTTCACCTTGGTTATGCCATGTGTTTTCAAATTCCTTACCAGTACTCCATATGTTGAGTTGACGGCAGATGGTGTCACTTATGCAACTTGTAGATTAAATTCTGGAGAAGAATTTGTTCTGTTTTCAAGTATGGAATGTACTGTTTCTGAAAACTTGACTCCTTCATCAATTGTCTACGGACAAGTTTCTGTTCCACTTACTTTTAATGCTGGTGGATCTGGTTCAGAAACAGATATTGAAGCTTCTACATGTTTTGTTGTTGGAGAAAACACTGTGACCTTTACCGATGGTGATAATAGTCTTTCTATCCAAGTTAATTTTGAAGCAAATCCTGCAGATCCATCTGGATTACTTAGTTCTCAAAGAGTTATTCAATCACTTGCTAAATCATTAGCTTTGGTAATAATTCCAGACTGTCCCAACGGATATGCTTCCGGTACTCTTGGTATTTCTTCTACTGCAGATGGATACCAATTAGATTGTAATTCTATTGAAGCTGGCTTAACTAGTGGATTAAATGCTTGGAACAATCCTATTGATAACATTGACTTCCCACATACTTCACAATGTACTACGAAAGGTTTTAGTATATCATTTCTGAACATACCAGCAGGGTATAGACCATTTATTAATGCGCTTGCAACAGTTCCAAGTACTGAACAATATAGGGTTGCTTATGAGGTTAAATATACTTGTGTTGGTGGATCATATCGTGACGATTCGATGACAAGATTATGGAATCCATACCAAAGAAGTGAAGCTGATCTGTATGGGCAACCAATTGAAATCATCACTAGAACAGTTACAGAAGCAACTACCTACGTTACAACTTTACCATTTGATTCAGCTACCCAAAGAACAAGAACCATTGAAATTGTTAAACAAATGCCTTTAACAACAATAACAGGCTCATATGTGGGTGTGACAACAAGAAGTACAACACTTCCTTTTGTACTTGGAGARACTGCTACTGTATTGGTGGAATCGCCATACCATATTACTACAACAGTCACAACTTTTTGGGATGAAGAATACACTTCTACTTTGACAGTTATTGATCCATCAGAATCTATTGATACTGTCGTTGTAAATCATCCAGCTAATCCAACTACCACACTTACTAGTCTTTGGGTTGAAACTTATACTACAACTTTGACAGAAACCCATGGAGTAGGTGAAGC

**>*CtrALS941*_1021**

ATGTTGTTGCTACAACTAGTAATTACATTATTGACCTCTGTTAAAGCTGTGCTTGCAGACGAAATATCGGGTGTTTTTACCAGTTTTGATTCATTAACGTTTTCCCATCCTAGACTTACTTATACTCCACCAAATTTCCCAACATGGACAGCTGTTTTTGGTTGGGATCTTGAAGGTAGCACTGCTCGTCCAGGAGATGTGTTCACCTTGGTTATGCCATGTGTTTTCAAATTCCTTACCAGTACTCCATATGTTGAGTTGACGGCAGATGGTGTCACTTATGCAACTTGTAGATTAAATTCTGGAGAAGAATTTGTTCTGTTTTCAAGTATGGAATGTACTGTTTCTGAAAACTTGACTCCTTCATCAATTGTCTACGGACAAGTTTCTGTTCCACTTACTTTTAATGCTGGTGGATCTGGTTCAGAAACAGATATTGAAGCTTCTACATGTTTTGTTGTTGGAGAAAACACTGTGACCTTTACCGATGGTGATAATAGTCTTTCTATCCAAGTTAATTTTGAAGCAAATCCTGCAGATCCATCTGGATTACTTAGTTCTCAAAGAGTTATTCAATCACTTGCTAAATCATTAGCTTTGGTAATAATTCCAGACTGTCCCAACGGATATGCTTCCGGTACTCTTGGTATTTCTTCTACTGCGGATGGATACCAATTAGATTGTAATTCTATTGAAGCTGGCTTAACTAGTGGATTAAATGCTTGGAACAATCCTATTGATAACATTGACTTCCCACATACTTCACAATGTACTACGAAAGGTTTTAGTATATCATTTCTGAACATACCAGCAGGGTATAGACCATTTATTAATGCGCTTGCAACAGTTCCAAGTACTGAACAATATAGGGTTGCTTATGAGGTTAAATATACTTGTGTTGGTGGATCATATCGTGACGATTCGATGACAAGATTATGGAATCCATACCAAAGAAGTGAAGCTGATCTGTATGGGCAACCAATTGAAATCATCACTAGAACAGTTACAGAAGCAACTACCTACGTTACAACTTTACCATTTGATTCAGCTACCCAAAGAACAAGAACCATTGAAATTGTTAAACAAATGCCTTTAACAACAATAACAGGCTCATATGTGGGTGTGACAACAAGAAGTACAACACTTCCTTTTGTACTTGGAGAGACTGCTACTGTATTGGTGGAATCGCCATACCATATTACTACAACAGTCACAACTTTTTGGGATGAAGAATACACTTCTACTTTGACAGTTATTGATCCATCAGAATCTATTGATACTGTCGTTGTAAATCATCCAGCTAATCCAACTACCACACTTACTAGTCTTTGGGTTGAAACTTATACTACAACTTTGACAGAAACCCATGGAGTAGGTGAAGC

**>*CtrALS941*_3242**

ATGTTGTTGCTACAACTAGTAATTACATTATTGACCTCTGTTAAAGCTGTGCTTGCAGACGAAATATCGGGTGTTTTTACCAGTTTTGATTCATTAACGTTTTCCCATCCTAGACTTACTTATACTCCACCAAATTTCCCAACATGGACAGCTGTTTTTGGTTGGGATCTTGAAGGTAGCACTGCTCGTCCAGGAGATGTGTTCACCTTGGTTATGCCATGTGTTTTCAAATTCCTTACCAGTACTCCATATGTTGAGTTGACGGCAGATGGTGTCACTTATGCAACTTGTAGATTAAATTCTGGAGAAGAATTTGTTCTGTTTTCAAGTATGGAATGTACTGTTTCTGAAAACTTGACTCCTTCATCAATTGTCTACGGACAAGTTTCTGTTCCACTTACTTTTAATGCTGGTGGATCTGGTTCAGAAACAGATATTGAAGCTTCTACATGTTTTGTTGTTGGAGAAAACACTGTGACCTTTACCGATGGTGATAATAGTCTTTCTATCCAAGTTAATTTTGAAGCAAATCCTGCAGATCCATCTGGATTACTTAGTTCTCAAAGAGTTATTCAATCACTTGCTAAATCATTAGCTTTGGTAATAATTCCAGACTGTCCCAACGGATATGCTTCCGGTACTCTTGGTATTTCTTCTACTGCAGATGGATACCAATTAGATTGTAATTCTATTGAAGCTGGCTTAACTAGTGGATTAAATGCTTGGAACAATCCTATTGATAACATTGACTTCCCACATACTTCACAATGTACTACGAAAGGTTTTAGTATATCATTTCTGAACATACCAGCAGGGTATAGACCATTTATTAATGCGCTTGCAACAGTTCCAAGTACTGAACAATATAGGGTTGCTTATGAGGTTAAATATACTTGTGTTGGTGGATCATATCGTGACGATTCGATGACAAGATTATGGAATCCATACCAAAGAAGTGAAGCTGATCTGTATGGGCAACCAATTGAAATCATCACTAGAACAGTTACAGAAGCAACTACCTACGTTACAACTTTACCATTTGATTCAGCTACCCAAAGAACAAGAACCATTGAAATTGTTAAACAAATGCCTTTAACAACAATAACAGGCTCATATGTGGGTGTGACAACAAGAAGTACAACACTTCCTTTTGTACTTGGAGAAACTGCTACTGTATTGGTGGAATCGCCATACCATATTACTACAACAGTCACAACTTTTTGGGATGAAGAATACACTTCTACTTTGACAGTTATTGATCCATCAGAATCTATTGATACTGTCGTTGTAAATCATCCAGCTAATCCAACTACCACACTTACTAGTCTTTGGGTTGAAACTTATACTACAACTTTGACAGAAACCCATGGAGTAGGTGAAGC

***CtrALS1028* (Amplified with primers Ctr1028NT-F and Ctr1028NT-R)**

**>*CtrALS1028*_MYA-3404**

ATGAAGTTTTTGGGATTAGTTTTATTATTCTTGTCCTTGATTAATCAAGTGACTCCTAAGGAAGTATCGGGAATATTCACCAGTTTTAATTCATTAACTTGGTCAGATGCCGGTAATTATGGTTATCGTGGTCCTGCTAATCCAGCTTGGCAAGCTAAATTAAGTTGGTCTTTAGAAGGTAAAAARGTTAACCCAGGTGATACTTTTACTTTAACAATGCCTTGTGTTTTTAAATTTGTTACTACTCAACCTTCTATTGATTTGGCTGCTAATGGTATTACTTATGCTACTTGTACATTTCATTCGGGTGAAGAATTTACCACTTTTTCAACTGTTAGTTGTATTGTTAGCGATGCATTGACTTCCTCAACTCAAGCTTTTGGTACGGTCAGTATTCCATTTTCTTTCAATATTGGTGGTTCTGGTTCGGATGTTGATTTAACTGATTCAACTTGTTTCACTACTGGCTCCAATACTGTTACATTTAAAGATGGTGATAATGAACTTTCAATTCAAACAAATTTTGAACAAACTAAAGATTCACAATCTGGTTTAATCACTAATGCCAGAGTTATTCCATCACTTGGTCAATTGTCMCATCTTGTTGTTGCTCCAGATTGTCCAAATGGTTATGCYAGTGGTGAATTAGGAATCTATGCAAGAGACAATTCAGTTACTATTAATTGTGAAAATATTCACATTGGTATTACTGATAAATTAAATGCATGGAATAATCCAACAAACTCTAATGGTTTCACCTACACTAAGAAATGTGATTCCAATGGATTTTCAATTTCTTTTAAAAACRTTCCAACTGGTTATAGACCATTTTTGGATTCATTGATTAATGCTGCTACTGATTATACTTTTACTATTAGTTACATTTCCAAGTACACATGTGCTACCGGTGATTATCATGATAAATCTATTACTAAAAATTGGGCTCCATATAAAAATGGTCTTGCTGATAGTGATGGTGCTGTTGTTTTTGTTACAACTAGTACTTATCTTGAATCAACTACTGGCGTCACAACTTTACCATTTGATTCAAACAATGATAAAASCAAAACAATTGAAGTTCTTGTTCCTATTCCAACCACAACAGTAACTGAATCATATGTTGGCGTAACTACTTCTTATACCACCATTTCTGCTCCAATTGGTGGAACTGCTACCGTTATTGTTGACGAACCATATCATATCACAACCACCGTTTACACATCTTGGACTGGTGAAGGAACCACATCATATACTGTTACTGCTTCAACTGATTCAGTAGACACTGTATATGTTGAAACCCCGGTTCCAAATCCAACAGTCACCACCACTGAATATGGTT

**>*CtrALS1028*_951**

ATGAAGTTTTTGGGATTAGTTTTATTATTCTTGTCCTTGATTAATCAAGTGACTCCTAAGGAAGTATCTGGCATATTTACCAGTTTTAATTCATTAACTTGGTCAGATGCCGGTAATTATGGTTATCGTGGTCCTGCTAATCCAACTTGGCAAGCTAAATTAAGTTGGTCGTTAGAAGGTAAAAAAGTTAACCCCGGTGATACTTTTACTTTAACGATGCCTTGTGTTTTCAAATTTGTCACTACTCAACCTTCTATTGATTTGGCTGCTAATGGTATTACTTATGCTACTTGTACATTTCATTCGGGTGAAGAATTCACCACATTTTCAACTGTTGGTTGTGTTGTTAGTGATTCATTGACTTCTTCAACTCAAGCTTTTGGTACTGTTAGTATTCCATTTTCTTTCAATATTGGTGGTTCTGGTTCTGATGTCGACTTAACAGATTCAACTTGTTTTACTACTGGTTCCAATACTGTTACATTTAAAGATGGTGATAACGAACTTTCAATTCAAACAAATTTTGAACAAACTAAAGATCCACAATCTGGTTTAATCACTAATGCCAGAGTTATTCCATCACTTGGCCAATTGTCTCATCTTGTTGTTGCTCCAGATTGTCCTAATGGTTATGCTAGTGGTGAATTAGGAATTTATGCCAGAGACAATTCAGTTACTATTAATTGTGAAAATATTCATATTGGTATTACTGATAAATTAAATGCATGGAATAATCCAACAAACTCGAATGGTTTCACTTGCACTAAGAAATGTGATTCCAATGGATTTTCTATTTCTTTTAGTAACATTCCAGCTGGTTATAGACCATTTTTAGATTCATTGATTAATGCTGCTACTGATTATACTTTTACTATCAGTTATATTTCCAAGTACACATGCGCTACTGGTGATTATCATGATAAATCTATTACTAAAAATTGGGCTCCATATAAAAATGGTCTTGCCGATAGTGATGGAGCTGTTGTTTTTGTTACAACTAGTACTTATCTTGAATCCACTACTGGTGTTACTACTTTACCATTTGATGCAAACCATGATAAAACCAAAACAATTGAAGTTCTTGTTCCTATTCCAACCACAACAGTAACTGAATCATATGTTGGCGTCACAACTTCTTATACCACCATTACTGCTCCAATTGGTGGGACTGCTACTGTCATTGTCGATGAACCGTATCATATCACAACCACGGTTTACAAATCTTGGACTGGAGAAGGAACTACTTCATATACCGTTACCGCTTCAACTGATTCAGTAGATACTGTATATGTTGAAACCCCCGTACCAAATCCAACTGTCACCACCACTGAATATGACT

**>*CtrALS1028*_952**

ATGAAGTTTTTGGGATTAGTTTTATTATTCTTGTCCTTGATTAATCAAGTGACTCCTAAGGAAGTATCGGGAATATTCACCAGTTTTAATTCATTAACTTGGTCAGATGCCGGTAATTATGGTTATCGTGGTCCTGCTAATCCAGCTTGGCAAGCTAAATTAAGTTGGTCTTTAGAAGGTAAAAAGGTTAACCCAGGTGATACTTTTACTTTAACAATGCCTTGTGTTTTTAAATTTGTTACTACTCAACCTTCTATTGATTTGGCTGCTAATGGTATTACTTATGCTACTTGTACATTTCATTCGGGTGAAGAATTTACCACTTTTTCAACTGTTAGTTGTATTGTTAGCGATGCATTGACTTCCTCAACTCAAGCTTTTGGTACGGTCAGTATTCCATTTTCTTTCAATATTGGTGGTTCTGGTTCGGATGTTGATTTAACTGATTCAACTTGTTTCACTACTGGCTCCAATACTGTTACATTTAAAGATGGTGATAATGAACTTTCAATTCAAACAAATTTTGAACAAACTAAAGATTCACAATCTGGTTTAATCACTAATGCCAGAGTTATTCCATCACTTGGTCAATTGTCCCATCTTGTTGTTGCTCCAGATTGTCCAAATGGTTATGCCAGTGGTGAATTAGGAATCTATGCAAGAGACAATTCAGTTACTATTAATTGTGAAAATATTCACATTGGTATTACTGATAAATTAAATGCATGGAATAATCCAACAAACTCTAATGGTTTCACCTACACTAAGAAATGTGATTCCAATGGATTTTCAATTTCTTTTAAAAACGTTCCAACTGGTTATAGACCATTTTTGGATTCATTGATTAATGCTGCTACTGATTATACTTTTACTATTAGTTACATTTCCAAGTACACATGTGCTACCGGTGATTATCATGATAAATCTATTACTAAAAATTGGGCTCCATATAAAAATGGTCTTGCTGATAGTGATGGTGCTGTTGTTTTTGTTACAACTAGTACTTATCTTGAATCAACTACTGGCGTCACAACTTTACCATTTGATTCAAACAATGATAAAACCAAAACAATTGAAGTTCTTGTTCCTATTCCAACCACAACAGTAACTGAATCATATGTTGGCGTAACTACTTCTTATACCACCATTTCTGCTCCAATTGGTGGAACTGCTACCGTTATTGTTGACGAACCATATCATATCACAACCACCGTTTACACATCTTGGACTGGTGAAGGAACCACATCATATACTGTTACTGCTTCAACTGATTCAGTAGACACTGTATATGTTGAAACCCCGGTTCCAAATCCAACAGTCACCACCACTGAATATGGTT

**>*CtrALS1028*_1019**

ATGAAGTTTTTGGGATTAGTTTTATTATTCTTGTCCTTGATTAATCAAGTGACTCCTAAGGAAGTATCGGGAATATTCACCAGTTTTAATTCATTAACTTGGTCAGATGCCGGTAATTATGGTTATCGTGGTCCTGCTAATCCAGCTTGGCAAGCTAAATTAAGTTGGTCTTTAGAAGGTAAAAAGGTTAACCCAGGTGATACTTTTACTTTAACAATGCCTTGTGTTTTTAAATTTGTTACTACTCAACCTTCTATTGATTTGGCTGCTAATGGTATTACTTATGCTACTTGTACATTTCATTCGGGTGAAGAATTTACCACTTTTTCAACTGTTAGTTGTATTGTTAGCGATGCATTGACTTCCTCAACTCAAGCTTTTGGTACGGTCAGTATTCCATTTTCTTTCAATATTGGTGGTTCTGGTTCGGATGTTGATTTAACTGATTCAACTTGTTTCACTACTGGCTCCAATACTGTTACATTTAAAGATGGTGATAATGAACTTTCAATTCAAACAAATTTTGAACAAACTAAAGATTCACAATCTGGTTTAATMACTAATGCCAGAGTTATTCCATCACTTGGTCAATTGTCCCATCTTGTTGTTGCTCCAGATTGTCCAAATGGTTATGCYAGTGGTGAATTAGGAATCTATGCAAGAGACAATTCAGTTACTATTAATTGTGAAAATATTCACATTGGTATTACTGATAAATTAAATGCATGGAATAATCCAACAAACTCTAATGGTTTCACCTACACTAAGAAATGTGATTCCAATGGATTTTCAATTTCTTTTAAAAACGTTCCAACTGGTTATAGACCATTTTTGGATTCATTGATTAATGCTGCTACTGATTATACTTTTACTATTAGTTACATTTMCAAGTACACATGTGCTACCGGTGATTATCATGATAAATCTATTACTAAAAATTGGGCTCCATATAAAAATGGTCTTGCTGRTAGTGATGGTGCTGTTGTTTTTGTTACAACTAGTACTTATCTTGAATCAACTACTGGCGTCACAACTTTACCATTTGATTCAAACAATGATAAAACCAAAACAATTGAAGTTCTTGTTCCTATTCCAACCACAACAGTAACTGAATCATATGTTGGCGTAACTACTTCTTATACCACCATTTCTGCTCCAATTGGTGGAACTGCTACCGTTATTGTTGACGAACCATATCATATCACAACCACCGTTTACACATCTTGGACTGGTGAAGGAACCACATCATATACTGTTACTGCTTCAACTGATTCAGTAGACACTGTATATGTTGAAACCCCGGTTCCAAATCCAACAGTCACCACCACTGAATATGGTT

**>*CtrALS1028*_1020**

ATGAAGTTTTTGGGATTAGTTTTATTATTCTTGTCCTTGATTAATCAAGTGACTCCTAAGGAAGTATCGGGAATATTCACCAGTTTTAATTCATTAACTTGGTCAGATGCCGGTAATTATGGTTATCGTGGTCCTGCTAATCCAGCTTGGCAAGCTAAATTAAGTTGGTCTTTAGAAGGTAAAATGGTTAACCCAGGTGATACTTTTACTTTAACAATGCCTTGTGTTTTTAAATTTGTTACTACTCAACCTTCTATTGATTTGGCTGCTAATGGTATTACTTATGCTACTTGTACATTTCATTCGGGTGAAGAATTTACCACTTTTTCAACTGTTAGTTGTATTGTTAGCGATGCATTGACTTCCTCAACTCAAGCTTTTGGTACGGTCAGTATTCCATTTTCTTTCAATATTGGTGGTTCTGGTTCGGATGTTGATTTAACTGATTCAACTTGTTTCACTACTGGCTCCAATACTGTTACATTTAAAGATGGTGATAATGAACTTTCAATTCAAACAAATTTTGAACAAACTAAAGATTCACAATCTGGTTTAATCACTAATGCCAGAGTTATTCCATCACTTGGTCAATTGTCCCATCTTGTTGTTGCTCCAGATTGTCCAAATGGTTATGCCAGTGGTGAATTAGGAATCTATGCAAGAGACAATTCAGTTACTATTAATTGTGAAAATATTCACATTGGTATTACTGATAAATTAAATGCATGGAATAATCCAACAAACTCTAATGGTTTCACCTACACTAAGAAATGTGATTCCAATGGATTTTCAATTTCTTTTAAAAACGTTCCAACTGGTTATAGACCATTTTTGGATTCATTGATTAATGCTGCTACTGATTATACTTTTACTATTAGTTACATTTCCAAGTACACATGTGCTACCGGTGATTATCATGATAAATCTATTACTAAAAATTGGGCTCCATATAAAAATGGTCTTGCTGATAGTGATGGTGCTGTTGTTTTTGTTACAACTAGTACTTATCTTGAATCAACTACTGGCGTCACAACTTTACCATTTGATTCAAACAATGATAAAACCAAAACAATTGAAGTTCTTGTTCCTATTCCAACCACAACAGTAACTGAATCATATGTTGGCGTAACTACTTCTTATACCACCATTTCTGCTCCAATTGGTGGAACTGCTACCGTTATTGTTGACGAACCATATCATATCACAACCACCGTTTACACATCTTGGACTGGTGAAGGAACCACATCATATACTGTTACTGCTTCAACTGATTCAGTAGACACTGTATATGTTGAAACCCCGGTTCCAAATCCAACAGTCACCACCACTGAATATGGTT

**>*CtrALS1028*_1021**

ATGAAGTTTTTGGGATTAGTTTTATTATTCTTGTCCTTGATTAATCAAGTGACTCCTAAGGAAGTATCGGGAATATTCACCAGTTTTAATTCATTAACTTGGTCAGATGCCGGTAATTATGGTTATCGTGGTCCTGCTAATCCAGCTTGGCAAGCTAAATTAAGTTGGTCTTTAGAAGGTAAAAAGGTTAACCCAGGTGATACTTTTACTTTAACAATGCCTTGTGTTTTTAAATTTGTTACTACTCAACCTTCTATTGATTTGGCTGCTAATGGTATTACTTATGCTACTTGTACATTTCATTCGGGTGAAGAATTTACCACTTTTTCAACTGTTAGTTGTATTGTTAGCGATGCATTGACTTCCTCAACTCAAGCTTTTGGTACGGTCAGTATTCCATTTTCTTTCAATATTGGTGGTTCTGGTTCGGATGTTGATTTAACTGATTCAACTTGTTTCACTACTGGCTCCAATACTGTTACATTTAAAGATGGTGATAATGAACTTTCAATTCAAACAAATTTTGAACAAACTAAAGATTCACAATCTGGTTTAATCACTAATGCCAGAGTTATTCCATCACTTGGTCAATTGTCCCATCTTGTTGTTGCTCCAGATTGTCCAAATGGTTATGCCAGTGGTGAATTAGGAATCTATGCAAGAGACAATTCAGTTACTATTAATTGTGAAAATATTCACATTGGTATTACTGATAAATTAAATGCATGGAATAATCCAACAAACTCTAATGGTTTCACCTACACTAAGAAATGTGATTCCAATGGATTTTCAATTTCTTTTAAAAACGTTCCAACTGGTTATAGACCATTTTTGGATTCATTGATTAATGCTGCTACTGATTATACTTTTACTATTAGTTACATTTCCAAGTACACATGTGCTACCGGTGATTATCATGATAAATCTATTACTAAAAATTGGGCTCCATATAAAAATGGTCTTGCTGATAGTGATGGTGCTGTTGTTTTTGTTACAACTAGTACTTATCTTGAATCAACTACTGGCGTCACAACTTTACCATTTGATTCAAACAATGATAAAACCAAAACAATTGAAGTTCTTGTTCCTATTCCAACCACAACAGTAACTGAATCATATGTTGGCGTAACTACTTCTTATACCACCATTTCTGCTCCAATTGGTGGAACTGCTACCGTTATTGTTGACGAACCATATCATATCACAACCACCGTTTACACATCTTGGACTGGTGAAGGAACCACATCATATACTGTTACTGCTTCAACTGATTCAGTAGACACTGTATATGTTGAAACCCCGGTTCCAAATCCAACAGTCACCACCACTGAATATGGTT

**>*CtrALS1028*_3242**

ATGAAGTTTTTGGGATTAGTTTTATTATTCTTGTCCTTGATTAATCAAGTGACTCCTAAGGAAGTATCGGGAATATTCACCAGTTTTAATTCATTAACTTGGTCAGATGCCGGTAATTATGGTTATCGTGGTCCTGCTAATCCAGCTTGGCAAGCTAAATTAAGTTGGTCTTTAGAAGGTAAAAAGGTTAACCCAGGTGATACTTTTACTTTAACAATGCCTTGTGTTTTTAAATTTGTTACTACTCAACCTTCTATTGATTTGGCTGCTAATGGTATTACTTATGCTACTTGTACATTTCATTCGGGTGAAAAATTTACCACTTTTTCAACTGTTAGTTGTATTGTTAGCGATGCATTGACTTCCTCAACTCAAGCTTTTGGTACGGTCAGTATTCCATTTTCTTTCAATATTGGTGGTTCTGGTTCGGATGTTGATTTAACTGATTCAACTTGTTTCACTACTGGCTCCAATACTGTTACATTTAAAGATGGTGATAATGAACTTTCAATTCAAACAAATTTTGAACAAACTAAAGATTCACAATCTGGTTTAATCACTAATGCCAGAGTTATTCCATCACTTGGTCAATTGTCCCATCTTGTTGTTGCTCCAGATTGTCCAAATGGTTATGCCAGTGGTGAATTAGGAATCTATGCAAGAGACAATTCAGTTACTATTAATTGTGAAAATATTCACATTGGTATTACTGATAAATTAAATGCATGGAATAATCCAACAAACTCTAATGGTTTCACCTACACTAAGAAATGTGATTCCAATGGATTTTCAATTTCTTTTAAAAACGTTCCAACTGGTTATAGACCATTTTTGGATTCATTGATTAATGCTGCTACTGATTATACTTTTACTATTAGTTACATTTCCAAGTACACATGTGCTACCGGTGATTATCATGATAAATCTATTACTAAAAATTGGGCTCCATATAAAAATGGTCTTGCTGATAGTGATGGTGCTGTTGTTTTTGTTACAACTAGTACTTATCTTGAATCAACTACTGGCGTCACAACTTTACCATTTGATTCAAACAATGATAAAACCAAAACAATTGAAGTTCTTGTTCCTATTCCAACCACAACAGTAACTGAATCATATGTTGGCGTAACTACTTCTTATACCACCATTTCTGCTCCAATTGGTGGAACTGCTACCGTTATTGTTGACGAACCATATCATATCACAACCACCGTTTACACATCTTGGACTGGTGAAGGAACCACATCATATACTGTTACTGCTTCAACTGATTCAGTAGACACTGTATATGTTGAAACCCCGGTTCCAAATCCAACAGTCACCACCACTGAATATGGTT

***CtrALS1030* (Amplified with primers Ctr1030NT-F and Ctr1030NT-R)**

**>*CtrALS1030*_MYA-3404**

ATGTTCGTTTTTAGATTATATTTACTACTATTAGTATTTCTTTCTGAAGTAACTCCAAAAGAACTATCTGATGTCTTCACYAGTTTTAATTCATTGACGTTTACAGATGCTGGATATGGATATAGAGGTCCATCGAATCCAACATGGCATGCTAAATTAAGTTGGAATTTAAATGGTGCGTATGCTCGACCTGGTGATACTTTTGGTTTAGTTTTACCACATGTATTTAAGTTTGTCACTGCTCAATCGTATTTTACTTTATCTGCAGGGGGTGTGACATATGCAATCTGTGATTTCCAACCAGGTGAGTTATTCACAACATTTTCCTCGATCAAATGTACTGTTAGTGAGAAATTGAACCCAAACATTGAAGCTTTTGGTACAATTACTTTCCCGTTTGCTTTTGGTGTTGGTGGTTCAGGATCAGATACAGACTTAGTAAATTCTAATAGTTTCACCACTGGTGAGAATAGAGTCACTTTCAAGCATGGTTCGAAAGATCTTTGTATTGATGTTGATTTTCAAGGAAGTCCTGCCAAGACGACGGATTTACTTAGTTATGGTAGAATTATTCCTTCACTAAGAAAAATATCACATCTATTAACTAGTGCTGATAGTCCAAATGGTTATAAAAGTGGCAAATTAGGACTCGCGTCATCTGACGCAGGCCTCGGCATTGATTGTGATTCTGTTCATGTTGGTATTACCAATATGTTGAATCCTTGGAATCAACCAATGAATGCAGAATCGTTTTCTTATACTACACAATGTTCTGAAGAAGAAATTATGATAACTTTCAATGAAGTTCCAGAAGGGTATCGCCCATTTTTTGATGTTTTGTTTTCTCATACTGCCAGTGATATTTTTACCATGTTGTACACAAATGAATATGTTGGGGCTGATGGGGTTACATATGATGCTTCGATGAAAAAAGCTTGGAAATCCTATCAAGATAGTCTTCCACTGGGTGATGGCGCCATTATTATTGTTACTACTAGAACCGGCACTCAGTCAACCACTGCCGTTAGTACTTTGCCATATGATCCGGAAGTAGACCTTACAAAAACCATTGAAGTACTCGTGCCTATACCTACTACTACTACAACCACATCTTACCTTGGTGTATCTACATATTATTCCACCATTACGGCAACAATTGGAGCCACAGCTACAGTAATTGTTGATGAACCATATCATACCACAACTACTATCACTACATGTTGGGATGATAAAGGAGCGACCACATTTACACAAATAGCTGAATCTCACTCCGTAG

**>*CtrALS1030*_951**

ATGTTCGTTTTTAGATTATATTTACTACTATTAGTATTTCTTTCTGAAGTAACTCCAAAAGAACTATCTGATGTCTTCACCAGTTTTAATTCATTGACGTTTACAGATGCTGGATATGGATATAGAGGTCCATCGAATCCAACATGGCATGCTAAATTAAGTTGGAATTTAAATGGTGCGTATGCTCGACCTGGTGATACTTTTGGTTTAGTTTTACCACATGTATTTAAGTTTGTCACTGCTCAATCGTATTTTACTTTATCTGCAGGGGGTGTGACATATGCAATCTGTGATTTCCAACCAGGTGAGTTATTCACAACATTTTCCTCGATCAAATGTACTGTTAGTGAGAAATTGAACCCAAACATTGAAGCTTTTGGTACAATTACTTTCCCGTTTGCTTTTGGTGTTGGTGGTTCAGGATCAGATACAGACTTAGTAAATTCTAATAGTTTCACCACTGGTGAGAATAGAGTCACTTTCAAGCATGGTTCGAAAGATCTTTGTATTGATGTTGATTTTCAAGGAAGTCCTGCCAAGACGACGGATTTACTTAGTTATGGTAGAATTATTCCTTCACTAAGAAAAATATCACATCTATTAACTAGTGCTGATAGTCCAAATGGTTATAAAAGTGGCAAATTAGGACTCGCGTCATCTGACGCAGGCCTCGGCATTGATTGTGATTCTGTTCATGTTGGTATTACCAATATGTTGAATCCTTGGAATCAACCAATGAATGCAGAATCGTTTTCTTATACTACACAATGTTCTGAAGAAGAAATTATGATAACTTTCAATGAAGTTCCAGAAGGGTATCGCCCATTTTTTGATGTTTTGTTTTCTCATACTGCCAGTGATATTTTTACCATGTTGTACACAAATGAATATGTTGGGGCTGATGGGGTTACATATGATGCTTCGATGAAAAAAGCTTGGAAATCCTATCAAGATAGTCTTCCACTGGGTGATGGCGCCATTATTATTGTTACTACTAGAACCGGCACTCAGTCAACCACTGCCGTTAGTACTTTGCCATATGAGCCGGAAATAGACCTTACAAAAACCATTGAAGTACTCGTGCCTATACCTACTACTACTACAACCACATCTTACCTTGGTGTATCTACATATTATTCCACCATTACGGCAACAATTGGAGCCACAGCTACAGTAATTGTTGATGAACCATATCATACCACAACTACTATCACTACATGTTGGGATGATAAAGGAGCGACCACATTTACACAAATAGCTGAATCTCACTCCGTAG

**>*CtrALS1030*_952**

ATGTTCGTTTTTAGATTATATTTACTACTATTAGTATTTCTTTCTGAAGTAACTCCAAAAGAACTATCTGATGTCTTCACCAGTTTTAATTCATTGACGTTTACAGATGCTGGATATGGATATAGAGGTCCATCGAATCCAACATGGCATGCTAAATTAAGTTGGAATTTAAATGGTGCGTATGCTCGACCTGGTGATACTTTTGGTTTAGTTTTACCACATGTATTTAAGTTTGTCACTGCTCAATCGTATTTTACTTTATCTGCAGGGGGTGTGACATATGCAATCTGTGATTTCCAACCAGGTGAGTTATTCACAACATTTTCCTCGATCAAATGTACTGTTAGTGAGAAATTGAACCCAAACATTGAAGCTTTTGGTACAATTACTTTCCCGTTTGCTTTTGGTGTTGGTGGTTCAGGATCAGATACAGACTTAGTAAATTCTAATAGTTTCACCACTGGTGAGAATAGAGTCACTTTCAAGCATGGTTCGAAAGATCTTTGTATTGATGTTGATTTTCAAGGAAGTCCTGCCAAGACGACGGATTTACTTAGTTATGGTAGAATTATTCCTTCACTAAGAAAAATATCACATCTATTAACTAGTGCTGATAGTCCAAATGGTTATAAAAGTGGCAAATTAGGACTCGCGTCATCTGACGCAGGCCTCGGCATTGATTGTGATTCTGTTCATGTTGGTATTACCAATATGTTGAATCCTTGGAATCAACCAATGAATGCAGAATCGTTTTCTTATACTACACAATGTTCTGAAGAAGAAATTATGATAACTTTCAATGAAGTTCCAGAAGGGTATCGCCCATTTTTTGATGTTTTGTTTTCTCATACTGCCAGTGATATTTTTACCATGTTGTACACAAATGAATATGTTGGGGCTGATGGGGTTACATATGATGCTTCGATGAAAAAAGCTTGGAAATCCTATCAAGATAGTCTTCCACTGGGTGATGGCGCCATTATTATTGTTACTACTAGAACCGGCACTCAGTCAACCACTGCCGTTAGTACTTTGCCATATGAGCCGGAAATAGACCTTACAAAAACCATTGAAGTACTCGTGCCTATACCTACTACTACTACAACCACATCTTACCTTGGTGTATCTACATATTATTCCACCATTACGGCAACAATTGGAGCCACAGCTACAGTAATTGTTGATGAACCATATCATACCACAACTACTATCACTACATGTTGGGATGATAAAGGAGCGACCACATTTACACAAATAGCTGAATCTCACTCCGTAG

**>*CtrALS1030*_1019**

ATGTTCGTTTTTAGATTATATTTACTACTATTAGTATTTCTTTCTGAAGTAACTCCAAAAGAACTATCTGATGTCTTCACYAGTTTTAATTCATTGACGTTTACAGATGCTGGATATGGATATAGAGGTCCATCGAATCCAACATGGCATGCTAAATTAAGTTGGAATTTAAATGGTGCGTATGCTCGACCTGGTGATACTTTTGGTTTAGTTTTACCACATGTATTTAAGTTTGTCACTGCTCAATCGTATTTTACTTTATCTGCAGGGGGTGTGACATATGCAATCTGTGATTTCCAACCAGGTGAGTTATTCACAACATTTTCCTCGATCAAATGTACTGTTAGTGAGAAATTGAACCCAAACATTGAAGCTTTTGGTACAATTACTTTCCCGTTTGCTTTTGGTGTTGGTGGTTCAGGATCAGRTACAGACTTAGTAAATTCTAATAGTTTCACCACTGGTGAGAATAGAGTCACTTTCAAGCATGGTTCGAAAGATCTTTGTATTGATGTTGATTTTCAAGGAAGTCCTGCCAAGACGACGGATTTACTTAGTTATGGTAGAATTATTCCTTCACTAAGAAAAATATCACATCTATTAACTAGTGCTGATAGTCCAAATGGTTATAAAAGTGGCAAATTAGGACTCGCGTCATCTGACGCAGGCCTCGGCATTGATTGTGATTCTGTTCATGTTGGTATTACCAATATGTTGAATCCTTGGAATCAACCAATGAATGCAGAATCGTTTTCTTATACTACACAATGTTCTGAAGAAGAAATTATGATAACTTTCAATGAAGTTCCAGAAGGGTATCGCCCATTTTTTGATGTTTTGTTTTCTCATACTGCCAGTGATATTTTTACCATGTTGTACACAAATGAATATGTTGGGGCTGATGGGGTTACATATGATGCTTCGATGAAAAAAGCTTGGAAATCCTATCAAGATAGTCTTCCACTGGGTGATGGYGCCATTATTATTGTTACTACTAGAACCGGCACTCAGTCAACCACTGCCGTTAGTACTTTGCCATATGATCCGGAAATAGACCTTACAAAAACCATTGAAGTACTCGTGCCTATACCTACTACTACTACAACCACATCTTACCTTGGTGTATCTACATATTATTCCACCATTACGGCAACAATTGGAGCCACAGCTACAGTAATTGTTGATGAACCATATCATACCACAACTACTATCACTACATGTTGGGATGATAAAGGAGCGACCACATTTACACAAATAGCTGAATCTCACTCCGTAG

**>*CtrALS1030*_1020**

ATGTTCGTTTTTAGATTATATTTACTACTATTAGTATTTCTTTCTGAAGTAACTCCAAAAGAACTATCTGATGTCTTCACCAGTTTTAATTCATTGACGTTTACAGATGCTGGATATGGATATAGAGGTCCATCGAATCCAACATGGCATGCTAAATTAAGTTGGAATTTAAATGGTGCGTATGCTCGACCTGGTGATACTTTTGGTTTAGTTTTACCACATGTATTTAAGTTTGTCACTGCTCAATCGTATTTTACTTTATCTGCAGGGGGTGTGACATATGCAATCTGTGATTTCCAACCAGGTGAGTTATTCACAACATTTTCCTCGATCAAATGTACTGTTAGTGAGAAATTGAACCCAAACATTGAAGCTTTTGGTACAATTACTTTCCCGTTTGCTTTTGGTGTTGGTGGTTCAGGATCAGATACAGACTTAGTAAATTCTAATAGTTTCACCACTGGTGAGAATAGAGTCACTTTCAAGCATGGTTCGAAAGATCTTTGTATTGATGTTGATTTTCAAGGAAGTCCTGCCAAGACGACGGATTTACTTAGTTATGGTAGAATTATTCCTTCACTAAGAAAAATATCACATCTATTAACTAGTGCTGATAGTCCAAATGGTTATAAAAGTGGCAAATTAGGACTCGCGTCATCTGACGCAGGCCTCGGCATTGATTGTGATTCTGTTCATGTTGGTATTACCAATATGTTGAATCCTTGGAATCAACCAATGAATGCAGAATCGTTTTCTTATACTACACAATGTTCTGAAGAAGAAATTATGATAACTTTCAATGAAGTTCCAGAAGGGTATCGCCCATTTTTTGATGTTTTGTTTTCTCATACTGCCAGTGATATTTTTACCATGTTGTACACAAATGAATATGTTGGGGCTGATGGGGTTACATATGATGCTTCGATGAAAAAAGCTTGGAAATCCTATCAAGATAGTCTTCCACTGGGTGATGGCGCCATTATTATTGTTACTACTAGAACCGGCACTCAGTCAACCACTGCCGTTAGTACTTTGCCATATGAGCCGGAAATAGACCTTACAAAAACCATTGAAGTACTCGTGCCTATACCTACTACTACTACAACCACATCTTACCTTGGTGTATCTACATATTATTCCACCATTACGGCAACAATTGGAGCCACAGCTACAGTAATTGTTGATGAACCATATCATACCACAACTACTATCACTACATGTTGGGATGATAAAGGAGCGACCACATTTACACAAATAGCTGAATCTCACTCCGTAG

**>*CtrALS1030*_1021**

ATGTTCGTTTTTAGATTATATTTACTACTATTAGTATTTCTTTCTGAAGTAACTCCAAAAGAACTATCTGATGTCTTCACCAGTTTTAATTCATTGACGTTTACAGATGCTGGATATGGATATAGAGGTCCATCGAATCCAACATGGCATGCTAAATTAAGTTGGAATTTAAATGGTGCGTATGCTCGACCTGGTGATACTTTTGGTTTAGTTTTACCACATGTATTTAAGTTTGTCACTGCTCAATCGTATTTTACTTTATCTGCAGGGGGTGTGACATATGCAATCTGTGATTTCCAACCAGGTGAGTTATTCACAACATTTTCCTCGATCAAATGTACTGTTAGTGAGAAATTGAACCCAAACATTGAAGCTTTTGGTACAATTACTTTCCCGTTTGCTTTTGGTGTTGGTGGTTCAGGATCAGATACAGACTTAGTAAATTCTAATAGTTTCACCACTGGTGAGAATAGAGTCACTTTCAAGCATGGTTCGAAAGATCTTTGTATTGATGTTGATTTTCAAGGAAGTCCTGCCAAGACGACGGATTTACTTAGTTATGGTAGAATTATTCCTTCACTAAGAAAAATATCACATCTATTAACTAGTGCTGATAGTCCAAATGGTTATAAAAGTGGCAAATTAGGACTCGCGTCATCTGACGCAGGCCTCGGCATTGATTGTGATTCTGTTCATGTTGGTATTACCAATATGTTGAATCCTTGGAATCAACCAATGAATGCAGAATCGTTTTCTTATACTACACAATGTTCTGAAGAAGAAATTATGATAACTTTCAATGAAGTTCCAGAAGGGTATCGCCCATTTTTTGATGTTTTGTTTTCTCATACTGCCAGTGATATTTTTACCATGTTGTACACAAATGAATATGTTGGGGCTGATGGGGTTACATATGATGCTTCGATGAAAAAAGCTTGGAAATCCTATCAAGATAGTCTTCCACTGGGTGATGGCGCCATTATTATTGTTACTACTAGAACCGGCACTCAGTCAACCACTGCCGTTAGTACTTTGCCATATGAGCCGGAAATAGACCTTACAAAAACCATTGAAGTACTCGTGCCTATACCTACTACTACTACAACCACATCTTACCTTGGTGTATCTACATATTATTCCACCATTACGGCAACAATTGGAGCCACAGCTACAGTAATTGTTGATGAACCATATCATACCACAACTACTATCACTACATGTTGGGATGATAAAGGAGCGACCACATTTACACAAATAGCTGAATCTCACTCCGTAG

**>*CtrALS1030*_3242**

ATGTTCGTTTTTAGATTATATTTACTACTATTAGTATTTCTTTCTGAAGTAACTCCAAAAGAACTATCTGATGTCTTCACCAGTTTTAATTCATTGACGTTTACAGATGCTGGATATGGATATAGAGGTCCATCGAATCCAACATGGCATGCTAAATTAAGTTGGAATTTAAATGGTGCGTATGCTCGACCTGGTGATACTTTTGGTTTAGTTTTACCACATGTATTTAAGTTTGTCACTGCTCAATCGTATTTTACTTTATCTGCAGGGGGTGTGACATATGCAATCTGTGATTTCCAACCAGGTGAGTTATTCACAACATTTTCCTCGATCAAATGTACTGTTAGTGAGAAATTGAACCCAAACATTGAAGCTTTTGGTACAATTACTTTCCCGTTTGCTTTTGGTGTTGGTGGTTCAGGATCAGATACAGACTTAGTAAATTCTAATAGTTTCACCACTGGTGAGAATAGAGTCACTTTCAAGCATGGTTCGAAAGATCTTTGTATTGATGTTGATTTTCAAGGAAGTCCTGCCAAGACGACGGATTTACTTAGTTATGGTAGAATTATTCCTTCACTAAGAAAAATATCACATCTATTAACTAGTGCTGATAGTCCAAATGGTTATAAAAGTGGCAAATTAGGACTCGCGTCATCTGACGCAGGCCTCGGCATTGATTGTGATTCTGTTCATGTTGGTATTACCAATATGTTGAATCCTTGGAATCAACCAATGAATGCAGAATCGTTTTCTTATACTACACAATGTTCTGAAGAAGAAATTATGATAACTTTCAATGAAGTTCCAGAAGGGTATCGCCCATTTTTTGATGTTTTGTTTTCTCATACTGCCAGTGATATTTTTACCATGTTGTACACAAATGAATATGTTGGGGCTGATGGGGTTACATATGATGCTTCGATGAAAAAAGCTTGGAAATCCTATCAAGATAGTCTTCCACTGGGTGATGGCGCCATTATTATTGTTACTACTAGAACCGGCACTCAGTCAACCACTGCCGTTAGTACTTTGCCATATGAKCCGGAARTAGACCTTACAAAAACCATTGAAGTACTCGTGCCTATACCTACTACTACTACAACCACATCTTACCTTGGTGTATCTACATATTATTCCACCATTACGGCAACAATTGGAGCCACAGCTACAGTAATTGTTGATGAACCATATCATACCACAACTACTATCACTACATGTTGGGATGATAAAGGAGCGACCACATTTACACAAATAGCTGAATCTCACTCCGTAG

***CtrALS1038* (Amplified with primers Ctr1038NT-F and Ctr1038NT-R)**

**>*CtrALS1038*_MYA-3404**

ATGAAGCTCATTGGATTTGGTTTGCTATTACTGTCTTTGGCTACTTTGGTGGCACCGAAGGAAATTTCTGGTGTTTTCACTTCCTTTGATTCATTAAAATGGAACGAAGATCTGAATGATTTTAGAGGTCCAGCTAGTCCTACTTGGAAAGCAACATTGGGATGGTCTTTGGATGGTACTAAGGTCAATCCAGGTGATACATTCACCTTGATTATGCCTTGTGTTTTTAAATTTATCACTGAACAGACTACTATTGATTTAACAGCAAACGGTGTGAACTATGCTACATGTACCTTTCATGCTGGTGAAGAATTTACTGCATTTTCAAGTGTTGGATGTGTTGTTAAGGACGCTTTGAAATCTAACATCCAGGCCTTTGGTACTGTTACAATTCCATTTACTTTCAATGTTGGTGGCACTGGTACTTCTGTCAGTTTGCAAGATTCTACTTGTTTTACTACTGGTGATAATATTGTCACTTTCCAAGATGGTGATAACAAACTTTCGATTACTGCCAATTTCGAACCTACAGGTGCTTCTAGAAGCGATTTAATTGCCAATGCCAGATCAATTCCATCACTTGAGAAAATGACCCACCTTGTCATTGCTCCTGATTGCCCAAGTGGTTTTAAAAGTGGTACTATATCTTTGAATACAAATAATGGTGCTGATATTGACTGTGCCCAGGTACATGTTGGTATGACTAATTTTATTAATCCGTGGAACTACCCAACTAATTCCGAACAGAATTTCTCAAAACAACCAACCTGTAATGAAGGAAGTTTCACTCTTTCATTTGAAAGTGTTCCTGCCGGTTTCAGACCATTCTTTGATGTTATGGTTACTCCTAAAGGGAAAATGGGTTTTGACTACAAGTATAGTGTTGTCTGTGCAGATGGTGAATCTCTTGAGCATCCTACTCACTATGACTGGGGTACTTATAACACTCAAACAGCCGATAGTAATGGGGCTATTTTAGTTATCACTACAAGAACCGGTACACAATCAACTACTRGTGTTACTACTTTACCTTTTGATCCAAGTAAAGATCATACTAAAACAGTCGAAGTTATTGAACCAATTCCAACAACAACAATCACAACTTCATATGTCGGTGTCACTACTTCCTATACCACCATTACAGGTACAATTGGTGGAACTGCTACCGTTATTGTTGATGAACCGTATCATACCACAACCACCGTTTACACATCTTGGACTGGTGAAGGAACCACATYATATACTGTTACCGCTTCAACTGATTCAGTAGACACTGTGTATGTTGAAACCCCCGTACCA

**>*CtrALS1038*_951**

ATGAAGCTCATTGGGTTTGGTTTGCTATTACTGTCCTTGGCTACTTTGGTGACACCGAAGGAAATTTCTGGTGTTTTCACTTCCTTTGATTCATTAAAATGGAACGAAGATCTGAACGATTTTAGAGGTCCAGCTAGTCCTACTTGGAAAGCAACATTGGGATGGTCTTTAGATGGTGCTAAAGTCAATCCAGGCGATACATTCACCTTGATGATGCCTTGTGTTTTCAAGTTTATCACTGAACAAACTACCATTGATTTAACAGCAAATGGTGTGAGCTATGCTACCTGTACCTTTCATGCTGGTGAAGAATTTACTGCCTTTTCAAGTGTTGGATGTGTTGTTAAGGATGCCTTGAAATCCAACGTTCAGGCCTTTGGTACTGTCACAATCCCATTTACTTTCAATGTTGGTGGTACTGGTACTTCAGTCAGTTTGCAGGATTCTACTTGTTTTACTACTGGTGATAACGTTGTCACTTTCCAAGATGGTGATAACAAACTTTCGATCACTGCCAATTTCGAACCTACAGGTGCTTCCAGAAGCGATTTAATTGCCAATGCCAGATCAATTCCATCACTCGAGAAAATGACACATCTTGTCATTGCTCCTGATTGTCCAAGTGGTTTTAAAAGTGGTACTATATCTTTGAATACAAATAATGGTGCTGATATTGACTGTGCCCAGGTCCATGTTGGTATGACTAAGCTTATTAATCCGTGGAACTACCCAACTACTTCCGACCAGAATTTCTCAAAACAACCAACCTGTAATAAAGGAAGTTTCACACTTTCATTTGAAGATGTTCCTGCCGGTTATAGACCATTCTTTGATGTTATGGTTACTCCAAAAGGGGATATGGGGTTTGACTATAAGTATAGTGTTGTTTGCGCTGACGGTGAGTCTCGTGAGCATCCTACTCACTATAACTGGGGTACTTATAACACGCAGACAGCTGATAGTAATGGTGCTGTTTTGGTTATCACTACAAGAACAGGTACACAATCCACTACTGCTGTTTCTACTTTACCATTTGATCCAAGTAAAGATCATACTAAAACAGTTGAAGTTATCGAACCAATTCCAACAACTACAGTCACCACTTCATACCTCGGTGTCACTACTTCTTATACCACCATTACAGGTACTATAGGTGGAACTGCTACCGTTATTGTTGATGAACCGTATCATACCACAACCACCGTTTACACATCCTGGACTGGTGAAGGAACCACGTCATATACCGTTACCGCTTCAACTGATTCAGTAGATACTGTTTATGTTGAAACCCCCGTACCA

**>*CtrALS1038*_952**

ATGAAGCTCATTGGATTTGGTTTGCTATTACTGTCTTTGGCTACTTTGGTGGCACCGAAGGAAATTTCTGGTGTTTTCACTTCCTTTGATTCATTAAAATGGAACGAAGATCTGAATGATTTTAGAGGTCCAGCTAGTCCTACTTGGAAAGCAACATTGGGATGGTCTTTGGATGGTACTAAGGTCAATCCAGGTGATACATTCACCTTGATTATGCCTTGTGTTTTTAAATTTATCACTGAACAGACTACTATTGATTTAACAGCAAACGGTGTGAACTATGCTACATGTACCTTTCATGCTGGTGAAGAATTTACTGCATTTTCAAGTGTTGGATGTGTTGTTAAGGACGCTTTGAAATCTAACATCCAGGCCTTTGGTACTGTTACAATTCCATTTACTTTCAATGTTGGTGGCACTGGTACTTCTGTCAGTTTGCAAGATTCTACTTGTTTTACTACTGGTGATAATATTGTCACTTTCCAAGATGGTGATAACAAACTTTCGATTACTGCCAATTTCGAACCTACAGGTGCTTCTAGAAGCGATTTAATTGCCAATGCCAGATCAATTCCATCACTTGAGAAAATGACCCACCTTGTCATTGCTCCTGATTGTCCAAGTGGTTTTAAAAGTGGTACTATATCTTTGAATACAAATAATGGTGCTGATATTGACTGTGCCCAGGTACATGTTGGTATGACTAATTTTATTAATCCGTGGAACTACCCAACTAATTCCGAACAGAATTTCTCAAAACAACCAACCTGTAATGAAGGAAGTTTCACTCTTTCATTTGAAAGTGTTCCTGCCGGTTTCAGACCATTCTTTGATGTTATGGTTACTCCTAAAGGGAAAATGGGTTTTGACTACAAGTATAGTGTTGTCTGTGCAGATGGTGAATCTCTTGAGCATCCTACTCACTATGACTGGGGTACTTATAACACTCAAACAGCCGATAGTAATGGGGCTATTTTAGTTATCACTACAAGAACCGGTACACAATCAACTACTGGTGTTACTACTTTACCTTTTGATCCAAGTAAAGATCATACTAAAACAGTCGAAGTTATTGAACCAATTCCAACAACAACAATCACAACTTCATATGTCGGTGTCACTACTTCCTATACCACCATTACAGGTACAATTGGTGGAACTGCTACCGTTATTGTTGATGAACCGTATCATACCACAACCACCGTTTACACATCTTGGACTGGTGAAGGAACCACATCATATACTGTTACCGCTTCAACTGATTCAGTAGACACTGTGTATGTTGAAACCCCCGTACCA

**>*CtrALS1038*_1019**

ATGAAGCTCATTGGATTTGGTTTGCTATTACTGTCTTTGGCTACTTTGGTGGCACCGAAGGAAATTTCTGGTGTTTTCACTTCCTTTGATTCATTAAAATGGAACGAAGATCTGAATGATTTTAGAGGTCCAGCTAGTCCTACTTGGAAAGCAACATTGGGATGGTCTTTGGATGGTACTAAGGTCAATCCAGGTGATACATTCACCTTGATTATGCCTTGTGTTTTTAAATTTATCACTGAACAGACTACTATTGATTTAACAGCAAACGGTGTGAACTATGCTACATGTACCTTTCATGCTGGTGAAGAATTTACTGCATTTTCAAGTGTTGGATGTGTTGTTAAGGACGCTTTGAAATCTAACATCCAGGCCTTTGGTACTGTTACAATTCCATTTACTTTCAATGTTGGTGGCACTGGTACTTCTGTCAGTTTGCAAGATTCTACTTGTTTTACTACTGGTGATAATATTGTCACTTTCCAAGATGGTGATAACAAACTTTCGATTACTGCCAATTTCGAACCTACAGGTGCTTCTAGAAGCGATTTAATTGCCAATGCCAGATCAATTCCATCACTTGAGAAAATGACCCACCTTGTCATTGCTCCTGATTGTCCAAGTGGTTTTAAAAGTGGTACTATATCTTTGAATACAAATAATGGTGCTGATATTGACTGTGCCCAGGTACATGTTGGTATGACTAATTTTATTAATCCGTGGAACTACCCAACTAATTCCGAACAGAATTTCTCAAAACAACCAACCTGTAATGAAGGAAGTTTCACTCTTTCATTTGAAAGTGTTCCTGCCGGTTTCAGACCATTCTTTGATGTTATGGTTACTCCTAAAGGGAAAATGGGTTTTGACTACAAGTATAGTGTTGTCTGTGCAGATGGTGAATCTCTTGAGCATCCTACTCACTATGACTGGGGTACTTATAACACTCAAACAGCCGATAGTAATGGGGCTATTTTAGTTATCACTACAAGAACCGGTACACAATCAACTACTRGTGTTACTACTTTACCTTTTGATCCAAGTAAAGATCATACTAAAACAGTCGAAGTTATTGAACCAATTCCAACAACAACAATCACWACTTCATATGTMGGTRTCACWACTTCCTATACCACCATTACAGGTACAATTGGTGGAACTGCTACCGTTATTGTTGATGAACCGTATCATACCACAACCACCGTTTACACATCTTGGACTGGTGAAGGAACCACATCATATACTGTTACCGCTTCAACTGATTCAGTAGACACTGTGTATGTTGAAACCCCCGTACCA

**>*CtrALS1038*_1020**

ATGAAGCTCATTGGATTTGGTTTGCTATTACTGTCYTTGGCTACTTTGGTGRCACCGAAGGAAATTTCTGGTGTTTTCACTTCCTTTGATTCATTAAAATGGAACGAAGATCTGAAYGATTTTAGAGGTCCAGCTAGTCCTACTTGGAAAGCAACATTGGGATGGTCTTTRGATGGTRCTAARGTCAATCCAGGYGATACATTCACCTTGATKATGCCTTGTGTTTTYAARTTTATCACTGAACARACTACYATTGATTTAACAGCAAAYGGTGTGAACTATGCTACATGTACCTTTCATGCTGGTGAAGAATTTACTGCATTTTCAAGTGTTGGATGTGTTGTTAAGGACGCTTTGAAATCTAACATCCAGGCCTTTGGTACTGTYACAATTCCATTTACTTTCAATGTTGGTGGCACTGGTACTTCTGTCAGTTTGCARGATTCTACTTGTTTTACTACTGGTGATAAYRTTGTCACTTTCCAAGATGGTGATAACAAACTTTCGATYACTGCCAATTTCGAACCTACAGGTGCTTCTAGAAGCGATTTAATTGCCAATGCCAGATCAATTCCATCACTTGAGAAAATGACCCACCTTGTCATTGCTCCTGATTGTCCAAGTGGTTTTAAAAGTGGTACTATATCTTTGAATACAAATAATGGTGCTGATATTGACTGTGCCCAGGTACATGTTGGTATGACTAATTTTATTAATCCGTGGAACTACCCAACTAATTCCGAACAGAATTTCTCAAAACAACCAACCTGTAATGAAGGAAGTTTCACTCTTTCATTTGAAARTGTTCCTGCCGGTTTCAGACCATTCTTTGATGTTATGGTTACTCCTAAAGGGAAAATGGGTTTTGACTACAAGTATAGTGTTGTCTGTGCAGATGGTGAATCTCTTGAGCATCCTACTCACTATGACTGGGGTACTTATAACACTCAAACAGCCGATAGTAATGGGGCTATTTTAGTTATCACTACAAGAACCGGTACACAATCAACTACTGGTGTTACTACTTTACCTTTTGATCCAAGTAAAGATCATACTAAAACAGTCGAAGTTATTGAACCAATTCCAACAACAACAATCACAACTTCATATGTCGGTGTCACTACTTCYTATACCACCATTACAGGTACAATTGGTGGAACTGCTACCGTTATTGTTGATGAACCGTATCATACCACAACCACCGTTTACACATCTTGGACTGGTGAAGGAACCACATTATATACTGTTACCGCTTCAACTGATTCAGTAGACACTGTGTATGTTGAAACCCCCGTACCA

**>*CtrALS1038*_1021**

ATGAAGCTCATTGGATTTGGTTTGCTATTACTGTCTTTGGCTACTTTGGTGGCACCGAAGGAAATTTCTGGTGTTTTCACTTCCTTTGATTCATTAAAATGGAACGAAGATCTGAATGATTTTAGAGGTCCAGCTAGTCCTACTTGGAAAGCAACATTGGGATGGTCTTTGGATGGTACTAAGGTCAATCCAGGTGATACATTCACCTTGATTATGCCTTGTGTTTTTAAATTTATCACTGAACAGACTACTATTGATTTAACAGCAAACGGTGTGAACTATGCTACATGTACCTTTCATGCTGGTGAAGAATTTACTGCATTTTCAAGTGTTGGATGTGTTGTTAAGGACGCTTTGAAATCTAACATCCAGGCCTTTGGTACTGTTACAATTCCATTTACTTTCAATGTTGGTGGCACTGGTACTTCTGTCAGTTTGCAAGATTCTACTTGTTTTACTACTGGTGATAATATTGTCACTTTCCAAGATGGTGATAACAAACTTTCGATTACTGCCAATTTCGAACCTACAGGTGCTTCTAGAAGCGATTTAATTGCCAATGCCAGATCAATTCCATCACTTGAGAAAATGACCCACCTTGTCATTGCTCCTGATTGTCCAAGTGGTTTTAAAAGTGGTACTATATCTTTGAATACAAATAATGGTGCTGATATTGACTGTGCCCAGGTACATGTTGGTATGACTAATTTTATTAATCCGTGGAACTACCCAACTAATTCCGAACAGAATTTCTCAAAACAACCAACCTGTAATGAAGGAAGTTTCACTCTTTCATTTGAAAGTGTTCCTGCCGGTTTCAGACCATTCTTTGATGTTATGGTTACTCCTAAAGGGAAAATGGGTTTTGACTACAAGTATAGTGTTGTCTGTGCAGATGGTGAATCTCTTGAGCATCCTACTCACTATGACTGGGGTACTTATAACACTCAAACAGCCGATAGTAATGGGGCTATTTTAGTTATCACTACAAGAACCGGTACACAATCAACTACTRGTGTTACTACTTTACCTTTTGATCCAAGTAAAGATCATACTAAAACAGTCGAAGTTATTGAACCAATTCCAACAACAACAATCACAACTTCATATGTCGGTGTCACTACTTCCTATACCACCATTACAGGTACAATTGGTGGAACTGCTACCGTTATTGTTGATGAACCGTATCATACCACAACCACCGTTTACACATCTTGGACTGGTGAAGGAACCACATCATATACTGTTACCGCTTCAACTGATTCAGTAGACACTGTGTATGTTGAAACCCCCGTACCA

**>*CtrALS1038*_3242**

ATGAAGCTCATTGGATTTGGTTTGCTATTACTGTCTTTGGCTACTTTGGTGGCACCGAAGGAAATTTCTGGTGTTTTCACTTCCTTTGATTCATTAAAATGGAACGAAGATCTGAATGATTTTAGAGGTCCAGCTAGTCCTACTTGGAAAGCAACATTGGGATGGTCTTTGGATGGTACTAAGGTCAATCCAGGTGATACATTCACCTTGATTATGCCTTGTGTTTTTAAATTTATCACTGAACAGACTACTATTGATTTAACAGCAAACGGTGTGAACTATGCTACATGTACCTTTCATGCTGGTGAAGAATTTACTGCATTTTCAAGTGTTGGATGTGTTGTTAAGGACGCTTTGAAATCTAACATCCAGGCCTTTGGTACTGTTACAATTCCATTTACTTTCAATGTTGGTGGCACTGGTACTTCTGTCAGTTTGCAAGATTCTACTTGTTTTACTACTGGTGATAATATTGTCACTTTCCAAGATGGTGATAACAAACTTTCGATTACTGCCAATTTCGAACCTACAGGTGCTTCTAGAAGCGATTTAATTGCCAATGCCAGATCAATTCCATCACTTGAGAAAATGACCCACCTTGTCATTGCTCCTGATTGTCCAAGTGGTTTTAAAAGTGGTACTATATCTTTGAATACAAATAATGGTGCTGATATTGACTGTGCCCAGGTACATGTTGGTATGACTAATTTTATTAATCCGTGGAACTACCCAACTAATTCCGAACAGAATTTCTCAAAACAACCAACCTGTAATGAAGGAAGTTTCACTCTTTCATTTGAAAGTGTTCCTGCCGGTTTCAGACCATTCTTTGATGTTATGGTTACTCCTAAAGGGAAAATGGGTTTTGACTACAAGTATAGTGTTGTCTGTGCAGATGGTGAATCTCTTGAGCATCCTACTCACTATGACTGGGGTACTTATAACACTCAAACAGCCGATAGTAATGGGGCTATTTTAGTTATCACTACAAGAACCGGTACACAATCAACTACTRGTGTTACTACTTTACCTTTTGATCCAAGTAAAGATCATACTAAAACAGTCGAAGTTATTGAACCAATTCCAACAACAACAATCACAACTTCATATGTCGGTGTCACTACTTCCTATACCACCATTACAGGTACAATTGGTGGAACTGCTACCGTTATTGTTGATGAACCGTATCATACCACAACCACCGTTTACACATCTTGGACTGGTGAAGGAACCACATCATATACTGTTACCGCTTCAACTGATTCAGTAGACACTGTGTATGTTGAAACCCCCGTACCA

***CtrALS1041* (Amplified with primers Ctr1041NT-F and Ctr1041NT-R)**

**>*CtrALS1041*_MYA-3404**

ATGAGTTATTTTGGATTTGCTATACTATTGTTGGCCTTGTTCACAAGAGTAACCCCTAAAGAAATCACGGGGATATTCACCAGTTTTGACTCGTTAACTTATTCTGATGCAGGTAATTATGGATACCAAGGTCCAGGGAATCCAACATGGACTGCAACTTTAGGTTGGTCATTAGATGGTTCTGTTGCCTCTCCTGGTGATACATTCACCTTAATTATGCCTTGTGTTTTCAAATTTACTAGTTCATCAACATCAGTGGACTTAACTGCTGATGGTGTAACTTATGCTACTTGTAAATTGAATAATGGAGAAGAATTTACCACATTTTCTAGTATGTCTTGTGTTGTTAATAGTGCTTTAACTTCTGATACTCAAGCATTTGGAACTGTCACTGTACCATTCTCGTTTAATGTTGGTGGTACTGGTTCTTCTGTTGATTTAGAGGACTCCACTTGTTTTACTGCTGGTACCAATACTGTTACTTTTAAAGATGGTGATAATGAACTCTCGATCAATGCCGTTTTTGAGAAAACCACTGCTTCAGTATCAGATGAAATTATTTTTGTCAGATCAGTTCCTTCCATTGGTAAATTGCAACAGATTTCTATTGCAAAAGATTGTCCAAGTGGGTATGAAAGTGGTTATATGAGTATAATTATTAGAGATAATTCTGCTGTTATGGATTGTTCTTCAACTCATATTGGTATTACTAATGATCTTAATGATTGGAATCAACCAACAAATTCTGAAACATTTTCTTATACTGAGAGCTGTTCTGCTACAAACTTTACTATTTCATTCACTGATATCGAAGCTGGTTATAGACCATTTATGGATTCATTCCTCACTGCAACCGCCAATGCAAGATTTAATGTTGACTATATTTACAAGTATACTTGTAAAAATGGTGACACGGTTGATAAAACTAATAGTAGAGTTTATGCTCCTTATATCAACAGTAATACTGATAGTAATGGGGCTATTTTAGTTATCACTACAAGAACCGGTACACAATCAACTACTGGTGTTACTACTTTACCTTTTGATTCAAGCGTAGACCAAACTAAAACAGTTGAAGTTATTGTACCTATTCCAACAACCACAATCACTACTTCATATGTAGGTATCACAACTTCCTATACCACCATTACAGGTACAATTGGTGGAACTGCTACCGTTATTGTTGATGAACCGTATCATACCACAACCACCGTTTACACATCTTGGACTGGTGAAGGAACCACATCATATACTGTTACCGCTTCAACTGATTCAGTAGACACTGTGTATGTTGAAACCCCCGTACCAAATCCAACTGTTACTACCACTGAATATGGCTCTGTTTCTGCTGCTACTACCTACACCGAAACTGC

**>*CtrALS1041*_951**

ATGAGTTATTTTGGATTTGCTATACTATTGTTGRCSTTGTTCACAAGAGTAACACCTAAAGAARTCACAGGGATATTYACMAGTTTTGACTCSTTAACATATTCTGATGCAGGTAATTATGGATACCAAGGTCCAGGGAATCCAACATGGACTGCAACTTTAGGTTGGTCWTTAGATGGTTCTGTTGCCTCTCCCGGTGATACRTTCACCTTRATTATGCCKTGTGTTTTCAARTTTACTAGTYCAKMAACWTCAGTKGAYTTAACTGYTGATGGWGTAACTTATGCTACTTGTAAMTTGAATAAYGGRGAAGAATTTACCACATTTTCTAGTATGTCTTGTGTTGTTAATAGTGCYTTAACTTCTGATACTCAAGCRTTTGGAACTGTCACTGTACCATTCTCRTTTAAYGTTGGTGGTACTGGTTCWTCTGTTGATTTRGAGGACTCSACTTGTTTTACTGCTGGTACCAATACTGTTACTTTTAAAGATGGTGATAATGAWCTCTCGATCAATGCCGTTTTTGAGAAAACCACTGCTTCGGTATCAGATGAAATTATTTKTGTCAGATCMGTTCCTTCCATTGGTAARTTGCAACAGATTTCTATTGCAAAAGATTGTCCAAGTGGGTATGAAAGTGGTTATATGAGTATAATTATTAGAGATRATTCTGCTGTTATGGATTGTTCTTCAACTCATATTGGTATTACTAATGATCTYAATGATTGGAATCAACCAAYRAATTCTGAAWCATTTTCTTATACTGAGAGCTGTTCTGCTACAAACTTTACTATTTCATTCACTGATATCGAAGCTGGTTATAGACCATTYATGGATTCATTCCTCACTGCAACCGCCAATGCAAGATTTAATGTYGACTATATTTACAAGTATACTTGTAAAAATGGTGACACGGTTGATAMAACTAAYAGTAGAGTTTATGCTCCTTATATCAACAGTAATACTGATAGTAATGGGGCTRTTTTRGTTATCACTACAAGAACCGGTACACAATCAACTACTGGTGTTACTACTTTACCTTTTGATTCAAGCGTRGACCAAACTAAAACAGTTGAAGTTATTGTACCTATTCCAACAACCACAATCACTACTTCATATGTAGGTRTCACAACTTCCTATACCACCATTACAGGTACAATTGGTGGAACTGCTACCGTTATTGTTGATGAACCGTATCATACCACAACCACCGTTTACACATCTTGGACTGGTGAAGCAACCACATCATATACTGTTACCGCTTCAACTGATTCAGTAGACACTGTGTATGTTGAAACCCCCGTACCAAATCCAACTGTTACTACCACTGAATATGGCTCTGTTTCTGCTGCTACTACCTACACCGAAACTGC

**>*CtrALS1041*_952**

ATGAGTTATTTTGGATTTGCTATACTATTGTTGGCCTTGTTCACAAGAGTAACCCCTAAAGAAATCACGGGGATATTCACCAGTTTTGACTCGTTAACTTATTCTGATGCAGGTAATTATGGATACCAAGGTCCAGGGAATCCAACATGGACTGCAACTTTAGGTTGGTCATTAGATGGTTCTGTTGCCTCTCCTGGTGATACATTCACCTTAATTATGCCTTGTGTTTTCAAATTTACTAGTTCATCAACATCAGTGGACTTAACTGCTGATGGTGTAACTTATGCTACTTGTAAATTGAATAATGGAGAAGAATTTACCACATTTTCTAGTATGTCTTGTGTTGTTAATAGTGCTTTAACTTCTGATACTCAAGCATTTGGAACTGTCACTGTACCATTCTCGTTTAATGTTGGTGGTACTGGTTCTTCTGTTGATTTAGAGGACTCCACTTGTTTTACTGCTGGTACCAATACTGTTACTTTTAAAGATGGTGATAATGAACTCTCGATCAATGCCGTTTTTGAGAAAACCACTGCTTCAGTATCAGATGAAATTATTTTTGTCAGATCAGTTCCTTCCATTGGTAAATTGCAACAGATTTCTATTGCAAAAGATTGTCCAAGTGGGTATGAAAGTGGTTATATGAGTATAATTATTAGAGATAATTCTGCTGTTATGGATTGTTCTTCAACTCATATTGGTATTACTAATGATCTTAATGATTGGAATCAACCAACAAATTCTGAAACATTTTCTTATACTGAGAGCTGTTCTGCTACAAACTTTACTATTTCATTCACTGATATCGAAGCTGGTTATAGACCATTTATGGATTCATTCCTCACTGCAACCGCCAATGCAAGATTTAATGTTGACTATATTTACAAGTATACTTGTAAAAATGGTGACACGGTTGATAAAACTAATAGTAGAGTTTATGCTCCTTATATCAACAGTAATACTGATAGTAATGGGGCTATTTTAGTTATCACTACAAGAACCGGTACACAATCAACTACTGGTGTTACTACTTTACCTTTTGATTCAAGCGTAGACCAAACTAAAACAGTTGAAGTTATTGTACCTATTCCAACAACCACAATCACTACTTCATATGTAGGTATCACAACTTCCTATACCACCATTACAGGTACAATTGGTGGAACTGCTACCGTTATTGTTGATGAACCGTATCATACCACAACCACCGTTTACACATCTTGGACTGGTGAAGCAACCACATCATATACTGTTACCGCTTCAACTGATTCAGTAGACACTGTGTATGTTGAAACCCCCGTACCAAATCCAACTGTTACTACCACTGAATATGGCTCTGTTTCTGCTGCTACTACCTACACCGAAACTGC

**>*CtrALS1041*_1019**

ATGAGTTATTTTGGATTTGCGATACTATTGTTGGCCTTGTTCACAAGAGTAACCCCTAAAGAAATCACGGGGATATTCACCAGTTTTGACTCGTTAACTTATTCTGATGCAGGTAATTATGGATACCAAGGTCCAGGGAATCCAACATGGACTGCAACTTTAGGTTGGTCATTAGATGGTTCTGTTGCCTCTCCTGGTGATACATTCACCTTAATTATGCCTTGTGTTTTCAAATTTACTAGTTCATCAACATCAGTGGACTTAACTGCTGATGGTGTAACTTATGCTACTTGTAAATTGAATAATGGAGAAGAATTTACCACATTTTCTAGTATGTCTTGTGTTGTTAATAGTGCTTTAACTTCTGATACTCAAGCATTTGGAACTGTCACTGTACCATTCTCGTTTAATGTTGGTGGTACTGGTTCTTCTGTTGATTTAGAGGACTCCACTTGTTTTACTGCTGGTACCAATACTGTTACTTTTAAAGATGGTGATAATGAACTCTCGATCAATGCCGTTTTTGAGAAAACCACTGCTTCAGTATCAGATGAAATTATTTTTGTCAGATCAGTTCCTTCCATTGGTAAATTGCAACAGATTTCTATTGCAAAAGATTGTCCAAGTGGGTATGAAAGTGGTTATATGAGTATAATTATTAGAGATAATTCTGCTGTTATGGATTGTTCTTCAACTCATATTGGTATTACTAATGATCTTAATGATTGGAATCAACCAACAAATTCTGAAACATTTTCTTATACTGAGAGCTGTTCTGCTACAAACTTTACTATTTCATTCACTGATATCGAAGCTGGTTATAGACCATTTATGGATTCATTCCTCACTGCAACCGCCAATGCAAGATTTAATGTTGACTATATTTACAAGTATACTTGTAAAAATGGTGACACGGTTGATAAAACTAATAGTAGAGTTTATGCTCCTTATATCAACAGTAATACTGATAGTAATGGGGCTATTTTAGTTATCACTACAAGAACCGGTACACAATCAACTACTGGTGTTACTACTTTACCTTTTGATTCAAGCGTAGACCAAACTAAAACAGTTGAAGTTATTGTACCTATTCCAACAACCACAATCACTACTTCATATGTAGGTATCACAACTTCCTATACCACCATTACAGGTACAATTGGTGGAACTGCTACCGTTATTGTTGATGAACCGTATCATACCACAACCACCGTTTACACATCTTGGACTGGTGAAGGAACCACATCATATACTGTTACCGCTTCAACTGATTCAGTAGACACTGTGTATGTTGAAACCCCCGTACCAAATCCAACTGTTACTACCACTGAATATGGCTCTGTTTCTGCTGCTACTACCTACACCGAAACTGC

**>*CtrALS1041*_1020**

ATGAGTTATTTTGGATTTGCTATACTATTGTTGGCCTTGTTCACAAGAGTAACCCCTAAAGAAATCACGGGGATATTCACCAGTTTTGACTCGTTAACTTATTCTGATGCAGGTAATTATGGATACCAAGGTCCAGGGAATCCAACATGGACTGCAACTTTAGGTTGGTCATTAGATGGTTCTGTTGCCTCTCCTGGTGATACATTCACCTTAATTATGCCTTGTGTTTTCAAATTTACTAGTTCATCAACATCAGTGGACTTAACTGCTGATGGTGTAACTTATGCTACTTGTAAATTGAATAATGGAGAAGAATTTACCACATTTTCTAGTATGTCTTGTGTTGTTAATAGTGCTTTAACTTCTGATACTCAAGCATTTGGAACTGTCACTGTACCATTCTCGTTTAATGTTGGTGGTACTGGTTCTTCTGTTGATTTAGAGGACTCCACTTGTTTTACTGCTGGTACCAATACTGTTACTTTTAAAGATGGTGATAATGAACTCTCGATCAATGCCGTTTTTGAGAAAACCACTGCTTCAGTATCAGATGAAATTATTTTTGTCAGATCAGTTCCTTCCATTGGTAAATTGCAACAGATTTCTATTGCAAAAGATTGTCCAAGTGGGTATGAAAGTGGTTATATGAGTATAATTATTAGAGATAATTCTGCTGTTATGGATTGTTCTTCAACTCATATTGGTATTACTAATGATCTTAATGATTGGAATCAACCAACAAATTCTGAAACATTTTCTTATACTGAGAGCTGTTCTGCTACAAACTTTACTATTTCATTCACTGATATCGAAGCTGGTTATAGACCATTTATGGATTCATTCCTCACTGCAACCGCCAATGCAAGATTTAATGTTGACTATATTTACAAGTATACTTGTAAAAATGGTGACACGGTTGATAAAACTAATAGTAGAGTTTATGCTCCTTATATCAACAGTAATACTGATAGTAATGGGGCTATTTTAGTTATCACTACAAGAACCGGTACACAATCAACTACTGGTGTTACTACTTTACCTTTTGATTCAAGCGTAGACCAAACTAAAACAGTTGAAGTTATTGTACCTATTCCAACAACCACAATCACTACTTCATATGTAGGTATCACAACTTCTTATACCACCATTACAGGTACTATAGGTGGAACTGCTACCGTTATTGTTGATGAACCGTATCATACCACAACCACCGTTTACACATCTTGGACTGGTGAAGGAACCACATCATATACTGTTACCGCTTCAACTGATTCAGTAGACACTGTGTATGTTGAAACCCCCGCACCAAATCCAACTGTTACTACCACTGAATATGGCTCTGTTTCTGCTGCTACTACCTACACCGAAACTGC

**>*CtrALS1041*_1021**

ATGAGTTATTTTGGATTTGCKATACTATTGTTGGCCTTGTTCACAAGAGTAACCCCTAAAGAAATCACGGGGATATTCACCAGTTTTGACTCGTTAACTTATTCTGATGCAGGTAATTATGGATACCAAGGTCCAGGGAATCCAACATGGACTGCAACTTTAGGTTGGTCATTAGATGGTTCTGTTGCCTCTCCTGGTGATACATTCACCTTAATTATGCCTTGTGTTTTCAAATTTACTAGTTCATCAACATCAGTGGACTTAACTGCTGATGGTGTAACTTATGCTACTTGTAAATTGAATAATGGAGAAGAATTTACCACATTTTCTAGTATGTCTTGTGTTGTTAATAGTGCTTTAACTTCTGATACTCAAGCATTTGGAACTGTCACTGTACCATTCTCGTTTAATGTTGGTGGTACTGGTTCTTCTGTTGATTTAGAGGACTCCACTTGTTTTACTGCTGGTACCAATACTGTTACTTTTAAAGATGGTGATAATGAACTCTCGATCAATGCCGTTTTTGAGAAAACCACTGCTTCAGTATCAGATGAAATTATTTTTGTCAGATCAGTTCCTTCCATTGGTAAATTGCAACAGATTTCTATTGCAAAAGATTGTCCAAGTGGGTATGAAAGTGGTTATATGAGTATAATTATTAGAGATAATTCTGCTGTTATGGATTGTTCTTCAACTCATATTGGTATTACTAATGATCTTAATGATTGGAATCAACCAACAAATTCTGAAACATTTTCTTATACTGAGAGCTGTTCTGCTACAAACTTTACTATTTCATTCACTGATATCGAAGCTGGTTATAGACCATTTATGGATTCATTCCTCACTGCAACCGCCAATGCAAGATTTAATGTTGACTATATTTACAAGTATACTTGTAAAAATGGTGACACGGTTGATAAAACTAATAGTAGAGTTTATGCTCCTTATATCAACAGTAATACTGATAGTAATGGGGCTATTTTAGTTATCACTACAAGAACCGGTACACAATCAACTACTGGTGTTACTACTTTACCTTTTGATTCAAGCGTAGACCAAACTAAAACAGTTGAAGTTATTGTACCTATTCCAACAACCACAATCACTACTTCATATGTAGGTATCACAACTTCCTATACCACCATTACAGGTACAATTGGTGGAACTGCTACCGTTATTGTTGATGAACCGTATCATACCACAACCACCGTTTACACATCTTGGACTGGTGAAGSAACCACATCATATACTGTTACCGCTTCAACTGATTCAGTAGACACTGTGTATGTTGAAACCCCCGTACCAAATCCAACTGTTACTACCACTGAATATGGCTCTGTTTCTGCTGCTACTACCTACACCGAAACTGC

**>*CtrALS1041*_3242**

ATGAGTTATTTTGGATTTGCKATACTATTGTTGGCCTTGTTCACAAGAGTAACCCCTAAAGAAATCACGGGGATATTCACCAGTTTTGACTCGTTAACTTATTCTGATGCAGGTAATTATGGATACCAAGGTCCAGGGAATCCAACATGGACTGCAACTTTAGGTTGGTCATTAGATGGTTCTGTTGCCTCTCCTGGTGATACATTCACCTTAATTATGCCTTGTGTTTTCAAATTTACTAGTTCATCAACATCAGTGGACTTAACTGCTGATGGTGTAACTTATGCTACTTGTAAATTGAATAATGGAGAAGAATTTACCACATTTTCTAGTATGTCTTGTGTTGTTAATAGTGCTTTAACTTCTGATACTCAAGCATTTGGAACTGTCACTGTACCATTCTCGTTTAATGTTGGTGGTACTGGTTCTTCTGTTGATTTAGAGGACTCCACTTGTTTTACTGCTGGTACCAATACTGTTACTTTTAAAGATGGTGATAATGAACTCTCGATCAATGCCGTTTTTGAGAAAACCACTGCTTCAGTATCAGATGAAATTATTTTTGTCAGATCAGTTCCTTCCATTGGTAAATTGCAACAGATTTCTATTGCAAAAGATTGTCCAAGTGGGTATGAAAGTGGTTATATGAGTATAATTATTAGAGATAATTCTGCTGTTATGGATTGTTCTTCAACTCATATTGGTATTACTAATGATCTTAATGATTGGAATCAACCAACAAATTCTGAAACATTTTCTTATACTGAGAGCTGTTCTGCTACAAACTTTACTATTTCATTCACTGATATCGAAGCTGGTTATAGACCATTTATGGATTCATTCCTCACTGCAACCGCCAATGCAAGATTTAATGTTGACTATATTTACAAGTATACTTGTAAAAATGGTGACACGGTTGATAAAACTAATAGTAGAGTTTATGCTCCTTATATCAACAGTAATACTGATAGTAATGGGGCTATTTTAGTTATCACTACAAGAACCGGTACACAATCAACTACTGGTGTTACTACTTTACCTTTTGATTCAAGCGTAGACCAAACTAAAACAGTTGAAGTTATTGTACCTATTCCAACAACCACAATCACTACTTCATATGTAGGTATCACAACTTCCTATACCACCATTACAGGTACAATTGGTGGAACTGCTACCGTTATTGTTGATGAACCGTATCATACCACAACCACCGTTTACACATCTTGGACTGGTGAAGSAACCACATCATATACTGTTACCGCTTCAACTGATTCAGTAGACACTGTGTATGTTGAAACCCCCGTACCAAATCCAACTGTTACTACCACYGAATAYGRMTMTGTTTCTGYTGCTACTACCTAYACCGAAACTGC

***CtrALS2228* (Amplified with primers Ctr2228NT-F and Ctr2228NT-R)**

**>*CtrALS2228*_MYA-3404**

ATGGTTCTCATACAAGCCATTGTGCTATTAATATGGATTCAATTGGTATCTTCAAAAGAGATATCTGGGATTTTTATTGGTTTCGATTCATTAACATGGAATGCTGCTTCGGATTTACCCAGTGCATACCAAGGTCCACAAATTCCTACCTGGACTGCAGAACTAACATGGTTTTTGAATGGAGAATCTGCTGAACCAGGTGATACATTCACATTAATAATGCCATGTGTATTCAAGTTCATTACAAATCAAAACACTGTTGATTTGATTGCTGATGGCACTACTTATGCGACCTGTAATCTCAATTCTGGAGAAGAATTTACTACTTTCTCAAGTTTAAGCTGTACTGTTTCATCTACCTTGACAACATATACCCAAGCTCGTGGTACTTTACATGTTCCTTTGACATTTAATGTGGGTAGTTCGGGAACTTCTGTTTCGTTGACTGATTCTACTTGCTTCAGACCAGGTGTAAACACAGTAACTTTCAGAGATGGAGACAATGAAATTTCAACACAAGCAACTTTTCAAGGTAGTCCAGATGACCCATCACCAGACCGTTATTATCAAAGAGTTATTCCATCCCTTAACAAAGTGTCAAATCTAGTCTATCCTCCTAATTGTCCAAATGGGTATTCTTCCGGTGTTATCAGCTTCTCCTCGAGTGATTCTAATTTCCAACTTGACTGTTCCTCACTTGATGTTGGTATGACAAATCAATTAAATGCATGGAATTTCCCTACTAGTAGAGATTCTTTGTCTTATACAAGGAGTTGTAGTGACAAAAGTATTACTGTTAACTTTCAGAATGTTCCTGCGGGTTCGCGTCCCTACATTAGTGTCCTATCTGCTTTTCTTGGTACCAAAACCTATTCAATTAATTACAATTTGAGATACACTTGCAGTGGTAGTAGATCAAGTTCTGTGACAAAAACTATAAGCTGGGCTCCATATAGAAAYAGTTTGGCCGATTCCAATGGGGCAGTGGTCGTGTACACTACCAGCACAGTCACYGCGACAACGACCGGAGTTACAACACTTCCATTCAATCCATCAACAGACAAAACCGAAACTATTCTTGTTCTTGTACCTATTCCTACAACTACCATCACTACTTCATATGTAGGTGTTACTACTTCATACTCCACTATATCTGCACCCATTGGTGGTACTGCTACTGTCATTGTTGACAAACCATACCATTCCACAACTACAATTTACACAGGATGGACAGGTGAAAGAACCACGACAACAACACTCATTGCGTCGACTGATTCAATAGACACCATTTTAATTGAAACACCGCTT

**>*CtrALS2228*_951**

ATGGTTCTCATACAAGCCATTGTGCTATTAATATGGATTCAATTGGTATCTTCAAAAGAGATATCTGGGATTTTTATTGGTTTCGATTCATTAACATGGAATGCTGCTTCGGATTTACCCAGTGCATACCAAGGTCCACAAATTCCTACCTGGACTGCAGAACTAACATGGTTTTTGAATGGAGAATCTGCTGAACCAGGTGATACATTCACATTAATAATGCCATGTGTATTCAAGTTCATTACAAATCAAAACACTGTTGATTTGATTGCTGATGGCACTACTTATGCGACCTGTAATCTCAATTCTGGAGAAGAATTTACTACTTTCTCAAGTTTAAGCTGTACTGTTTCATCTACCTTGACAACATATACCCAAGCTCGTGGTACTTTACATGTTCCTTTGACATTTAATGTGGGTAGTTCGGGAACTTCTGTTTCGTTGACTGATTCTACTTGCTTCAGACCAGGTGTAAACACAGTAACTTTCAGAGATGGAGACAATGAAATTTCAACACAAGCAACTTTTCAAGGTAGTCCAGATGACCCATCACCAGACCGTTATTATCAAAGAGTTATTCCATCCCTTAACAAAGTGTCAAATCTAGTCTATCCTCCTAATTGTCCAAATGGGTATTCTTCCGGTGTTATCAGCTTCTCCTCGAGTGATTCTAATTTCCAACTTGACTGTTCCTCACTTGATGTTGGTATGACAAATCAATTAAATGCATGGAATTTCCCTACTAGTAGAGATTCTTTGTCTTATACAAGGAGTTGTAGTGACAAAAGTATTACTGTTAACTTTCAGAATGTTCCTGCGGGTTCGCGTCCCTACATTAGTGTCCTATCTGCTTTTCTTGGTACCAAAACCTATTCAATTAATTACAATTTGAGATACACTTGCAGTGGTAGTAGATCAAGTTCTGTGACAAAAACTATAAGCTGGGCTCCATATAGAAATAGTTTGGCCGATTCCAATGGGGCAGTGGTCGTGTACACTACCAGCACAGTCACCGCGACAACGACCGGAGTTACAACACTTCCATTCAATCCATCAACAGACAAAACCGAAACTATTCTTGTTCTTGTACCTATTCCTACAACTACCATCACTACTTCATATGTAGGTGTTACTACTTCATACTCCACTATATCTGCACCCATTGGTGGTACTGCTACTGTCATTGTTGACAAACCATACCATTCCACAACTACAATTTACACAGGATGGACAGGTGAAAGAACCACGACAACAACACTCATTTCGTCGACTGATTCAATAGACACCATTTTAATTGAAACACCGCTT

**>*CtrALS2228*_952**

ATGGTTCTCATACAAGCCATTGTGCTATTAATATGGATTCAATTGGTATCTTCAAAAGAGATATCTGGGATTTTTATTGGTTTCGATTCATTAACATGGAATGCTGCTTCGGATTTACCCAGTGCATACCAAGGTCCACAAATTCCTACCTGGACTGCAGAACTAACATGGTTTTTGAATGGAGAATCTGYTGAACCAGGTGATACWTTCACATTAATAATGCCATGTGTATTCAAGTTCATTACAAATCAAAACACTGTTGATTTGATTGCTGATGGCACTACTTATGCGACCTGTAATCTCAATTCTGGAGAAGAATTTACTACTTTCTCAAGTTTAAGCTGTWCTGTTTCATCTACCTTGACAACATATACCCAAGCTCGTGGTACTTTACATGTTCCTTTGACATTTAATGTGGGTAGTTCRGGAACTTCTGTTTCGTTGACTGATTCTACTTGCTTCAGACCAGGTGTAAACACAGTAACTTTCAGAGATGGAGACAATGAAATTTCAACACAAGCAACTTTTCAAGGTAGTCCAGATGACCCATCACCAGACCGTTATTATCAAAGAGTTATTCCATCCCTTAACAAAGTGTCAAATCTAGTCTATCCTCCTAATTGTCCAAATGGGTATTCWTCYGGTGTTATCAGCTTCTCCTCGAGTGATTCTAATTTCCAACTTGACTGYTCCTCACTTGATGTTGGTATGACAAATCAATTAAATGCATGGAATTTCCCTACYAGTAGAGATTCTTTGTCTTATACAAGGAGTTGTAGTGACAAAAGTATTACTGTTAACTTTCAGAATGTTCCTGCGGGTTCGCGTCCCTACATTAGTGTCCTATCTGCTTTTCTTGGTACCAAAACCTATTCAATTAATTACAATTTGAGATACACTTGCAGTGGTAGTAGATCAAGTTCTGTGACAAAAACTATAAGCTGGGCTCCATATAGAAAYAGTTTGGCCGATTCCAATGGGGCAGTGGTCGTGTACACTACCAGCACAGTCACCGCGACAACGACYGGAGTTACAACACTTCCATTYAATCCATCAACAGACAAAACCGAAACTATTCTTRTTCTTGTACCTATTCCTACAACTACCATCACTACTTCATATGTAGGTGTTACTACTTCATACTCCACTATATCTGCACCYATTGGTGGTACTGCTACTGTCATTGTTGACAAACCATACCATTCCACAACTACAATTTACACAGGATGGACAGGTGAAAGAACCACGACAACAACACTCATTGCGTCGACTGATTCAATAGACACCATTTTAATTGAAACACCGCTT

**>*CtrALS2228*_1019**

ATGGTTCTCATACAAGCCATTGTGCTATTAATATGGATTCAATTGGTATCTTCAAAAGAGATATCTGGGATTTTTATTGGTTTCGATTCATTAACATGGAATGCTGCTTCGGATTTACCCAGTGCATACCAAGGTCCACAAATTCCTACCTGGACTGCAGAACTAACATGGTTTTTGAATGGAGAATCTGCTGAACCAGGTGATACATTCACATTAATAATGCCATGTGTATTCAAGTTCATTACAAATCAAAACACTGTTGATTTGATTGCTGATGGCACTACTTATGCGACCTGTAATCTCAATTCTGGAGAAGAATTTACTACTTTCTCAAGTTTAAGCTGTACTGTTTCATCTACCTTGACAACATATACCCAAGCTCGTGGTACTTTACATGTTCCTTTGACATTTAATGTGGGTAGTTCGGGAACTTCTGTTTCGTTGACTGATTCTACTTGCTTCAGACCAGGTGTAAACACAGTAACTTTCAGAGATGGAGACAATGAAATTTCAACACAAGCAACTTTTCAAGGTAGTCCAGATGACCCATCACCAGACCGTTATTATCAAAGAGTTATTCCATCCCTTAACAAAGTGTCAAATCTAGTCTATCCTCCTAATTGTCCAAATGGGTATTCTTCCGGTGTTATCAGCTTCTCCTCGAGTGATTCTAATTTCCAACTTGACTGTTCCTCACTTGATGTTGGTATGACAAATCAATTAAATGCATGGAATTTCCCTACTAGTAGAGATTCTTTGTCTTATACAAGGAGTTGTAGTGACAAAAGTATTACTGTTAACTTTCAGAATGTTCCTGCGGGTTCGCGTCCCTACATTAGTGTCCTATCTGCTTTTCTTGGTACCAAAACCTATTCAATTAATTACAATTTGAGATACACTTGCAGTGGTAGTAGATCAAGTTCTGTGACAAAAACTATAAGCTGGGCTCCATATAGAAATAGTTTGGCCGATTCCAATGGGGCAGTGGTCGTGTACACTACCAGCACAGTCACCGCGACAACGACCGGAGTTACAACACTTCCATTCAATCCATCAACAGACAAAACCGAAACTATTCTTGTTCTTGTACCTATTCCTACAACTACCATCACTACTTCATATGTAGGTGTTACTACTTCATACTCCACTATATCTGCACCCATTGGTGGTACTGCTACTGTCATTGTTGACAAACCATACCATTCCACAACTACAATTTACACAGGATGGACAGGTGAAAGAACCACGACAACAACACTCATTGCGTCGACTGATTCAATAGACACCATTTTAATTGAAACACCGCTT

**>*CtrALS2228*_1020**

ATGGTTCTCATACAAGCCATTGTGCTATTAATATGGATTCAATTGGTATCTTCAAAAGAGATATCTGGGATTTTTATTGGTTTCGATTCATTAACATGGAATGCTGCTTCGGATTTACCCAGTGCATACCAAGGTCCACAAATTCCTACCTGGACTGCAGAACTAACATGGTTTTTGAATGGAGAATCTGCTGAACCAGGTGATACATTCACATTAATAATGCCATGTGTATTCAAGTTCATTACAAATCAAAACACTGTTGATTTGATTGCTGATGGCACTACTTATGCGACCTGTAATCTCAATTCTGGAGAAGAATTTACTACTTTCTCAAGTTTAAGCTGTACTGTTTCATCTACCTTGACAACATATACCCAAGCTCGTGGTACTTTACATGTTCCTTTGACATTTAATGTGGGTAGTTCGGGAACTTCTGTTTCGTTGACTGATTCTACTTGCTTCAGACCAGGTGTAAACACAGTAACTTTCAGAGATGGAGACAATGAAATTTCAACACAAGCAACTTTTCAAGGTAGTCCAGATGACCCATCACCAGACCGTTATTATCAAAGAGTTATTCCATCCCTTAACAAAGTGTCAAATCTAGTCTATCCTCCTAATTGTCCAAATGGGTATTCTTCCGGTGTTATCAGCTTCTCCTCGAGTGATTCTAATTTCCAACTTGACTGTTCCTCACTTGATGTTGGTATGACAAATCAATTAAATGCATGGAATTTCCCTACTAGTAGAGATTCTTTGTCTTATACAAGGAGTTGTAGTGACAAAAGTATTACTGTTAACTTTCAGAATGTTCCTGCGGGTTCGCGTCCCTACATTAGTGTCCTATCTGCTTTTCTTGGTACCAAAACCTATTCAATTAATTACAATTTGAGATACACTTGCAGTGGTAGTAGATCAAGTTCTGTGACAAAAACTATAAGCTGGGCTCCATATAGAAATAGTTTGGCCGATTCCAATGGGGCAGTGGTCGTGTACACTACCAGCACAGTCACCGCGACAACGACCGGAGTTACAACACTTCCATTCAATCCATCAACAGACAAAACCGAAACTATTCTTGTTCTTGTACCTATTCCTACAACTACCATCACTACTTCATATGTAGGTGTTACTACTTCATACTCCACTATATCTGCACCCATTGGTGGTACTGCTACTGTCATTGTTGACAAACCATACCATTCCACAACTACAATTTACACAGGATGGACAGGTGAAAGAACCACGACAACAACACTCATTGCGTCGACTGATTCAATAGACACCATTTTAATTGAAACACCGCTT

**>*CtrALS2228*_1021**

ATGGTTCTCATACAAGCCATTGTGCTATTAATATGGATTCAATTGGTATCTTCAAAAGAGATATCTGGGATTTTTATTGGTTTCGATTCATTAACATGGAATGCTGCTTCGGATTTACCCAGTGCATACCAAGGTCCACAAATTCCTACCTGGACTGCAGAACTAACATGGTTTTTGAATGGAGAATCTGYTGAACCAGGTGATACWTTCACATTAATAATGCCATGTGTATTCAAGTTCATTACAAATCAAAACACTGTTGATTTGATTGCTGATGGCACTACTTATGCGACCTGTAATCTCAATTCTGGAGAAGAATTTACTACTTTCTCAAGTTTAAGCTGTWCTGTTTCATCTACCTTGACAACATATACCCAAGCTCGTGGTACTTTACATGTTCCTTTGACATTTAATGTGGGTAGTTCRGGAACTTCTGTTTCGTTGACTGATTCTACTTGCTTCAGACCAGGTGTAAACACAGTAACTTTCAGAGATGGAGACAATGAAATTTCAACACAAGCAACTTTTCAAGGTAGTCCAGATGACCCATCACCAGACCGTTATTATCAAAGAGTTATTCCATCCCTTAACAAAGTGTCAAATCTAGTCTATCCTCCTAATTGTCCAAATGGGTATTCWTCYGGTGTTATCAGCTTCTCCTCGAGTGATTCTAATTTCCAACTTGACTGYTCCTCACTTGATGTTGGTATGACAAATCAATTAAATGCATGGAATTTCCCTACYAGTAGAGATTCTTTGTCTTATACAAGGAGTTGTAGTGACAAAAGTATTACTGTTAACTTTCAGAATGTTCCTGCGGGTTCGCGTCCCTACATTAGTGTCCTATCTGCTTTTCTTGGTACCAAAACCTATTCAATTAATTACAATTTGAGATACACTTGCAGTGGTAGTAGATCAAGTTCTGTGACAAAAACTATAAGCTGGGCTCCATATAGAAAYAGTTTGGCYGATTCCAATGGGGCAGTGGTCGTGTACACTACCAGCACAGTCACCGCGACAACGACYGGAGTTACAACACTTCCATTYAATCCATCAACAGACAAAACCGAAACTATTCTTGTTCTTGTACCTATTCCTACAACTACCATCACTACTTCATATGTAGGTGTTACTACTTCATACTCCACTATATCTGCACCYATTGGTGGTACTGCTACTGTCATTGTTGACAAACCATACCATTCCACAACTACAATTTACACAGGATGGACAGGTGAAAGAACCACGACAACAACACTCATTGCGTCGACTGATTCAATAGACACCATTTTAATTGAAACACCGCTT

**>*CtrALS2228*_3242**

ATGGTTCTCATACAAGCCATTGTGCTATTAATATGGATTCAATTGGTATCTTCAAAAGAGATATCTGGGATTTTTATTGGTTTCGATTCATTAACATGGAATGCTGCTTCGGATTTACCCAGTGCATACCAAGGTCCACAAATTCCTACCTGGACTGCAGAACTAACATGGTTTTTGAATGGAGAATCTGCTGAACCAGGTGATACATTCACATTAATAATGCCATGTGTATTCAAGTTCATTACAAATCAAAACACTGTTGATTTGATTGCTGATGGCACTACTTATGCGACCTGTAATCTCAATTCTGGAGAAGAATTTACTACTTTCTCAAGTTTAAGCTGTACTGTTTCATCTACCTTGACAACATATACCCAAGCTCGTGGTACTTTACATGTTCCTTTGACATTTAATGTGGGTAGTTCGGGAACTTCTGTTTCGTTGACTGATTCTACTTGCTTCAGACCAGGTGTAAACACAGTAACTTTCAGAGATGGAGACAATGAAATTTCAACACAAGCAACTTTTCAAGGTAGTCCAGATGACCCATCACCAGACCGTTATTATCAAAGAGTTATTCCATCCCTTAACAAAGTGTCAAATCTAGTCTATCCTCCTAATTGTCCAAATGGGTATTCTTCCGGTGTTATCAGCTTCTCCTCGAGTGATTCTAATTTCCAACTTGACTGTTCCTCACTTGATGTTGGTATGACAAATCAATTAAATGCATGGAATTTCCCTACTAGTAGAGATTCTTTGTCTTATACAAGGAGTTGTAGTGACAAAAGTATTACTGTTAACTTTCAGAATGTTCCTGCGGGTTCGCGTCCCTACATTAGTGTCCTATCTGCTTTTCTTGGTACCAAAACCTATTCAATTAATTACAATTTGAGATACACTTGCAGTGGTAGTAGATCAAGTTCTGTGACAAAAACTATAAGCTGGGCTCCATATAGAAATAGTTTGGCCGATTCCAATGGGGCAGTGGTCGTGTACACTACCAGCACAGTCACCGCGACAACGACCGGAGTTACAACACTTCCATTCAATCCATCAACAGACAAAACCGAAACTATTCTTGTTCTTGTACCTATTCCTACAACTACCATCACTACTTCATATGTAGGTGTTACTACTTCATACTCCACTATATCTGCACCCATTGGTGGTACTGCTACTGTCATTGTTGACAAACCATACCATTCCACAACTACAATTTACACAGGATGGACAGGTGAAAGAACCACGACAACAACACTCATTGCGTCGACTGATTCAATAGACACCATTTTAATTGAAACACCGCTT

***CtrALS2229* (Amplified with primers Ctr2229NT-F and Ctr2229NT-R)**

**>*CtrALS2229*_MYA-3404**

ATGTTGCGTCTATCAATTACTTATTCCATTTTCACATTAATTAACGTTATAAATGCCAAGGTCCTCTCCGGCATATTCACCAGATTTGTTTCATTAACACAATCATCTTATAATTCGTACAGTTTCGACGGTCCAATGCTGACAACTTGGATTGCAGCTCTAGGGTGGGAAATCAATGGAACAAAAGCAAAACCAGGTGATACATTTACTTTAGAGATGCCCTGTGTTTATAAAATTTTTATAAATGAAGAATCTATTGACTTAATTGGAAATGATATTAGTTTAGCAACTTGTGAAGTCCACTCTGGTGAACATATATCCACAAATTCGTATTTGAATTGTGTTATGAATGATTCTTTAGACGAAAGAACCAATATTGATGGAATTTTGAGACTCCCTATTATGTTCAATGTTGGAGGTTCCGGTCAAGATACTGACTTAGATGCGGCATCATCGTGTTTCAATACTGATTCCAACATAATTGCTTTCAACAATGGCGACACAAGTATTTCCATTCAACGAAGCTTTTCAGTAAGAGCAATTTTTGATACTGTACCAATAAACTTTGTTCGGGTAGGAAAAACTATTAGTGAATTAGAGGTACTCATTGTTGCTCCAAATTGTCCACAAGGTTACACAAATGGTAGACTAGCAATTGCTGCTTCAGACCGTGACGTTATCATAAAATGCTCTACTATAACATCGGGATTTGCCAGTAAACTTAACAAGTGGAACCTTCCAGAAAATTTAGTACAACTTTACCACGGCAGTCTTTGTACTTCACGTCAATTTTCTATTAGTTATACTAATATTCCAGAAGGGTATCGTCCATTTCTACTGGTAACTTTAAGCAATGCTGTTAGTTCTTCATTTAACCTTCGTTACACGATTCAGAATACATGTGAAAAAGACACGTTTAATGATCAATCAAGATCAGTTTCCTGGAGAAAGTTAAACTATGGATCTATCGATTCATATGGTGGTATCATGATTCCCGTCACAAGAACTAGAACAGGCACCGCCTCAACTAGATTGGTCACTACACGACCTTTTGATCCTTCGACGCACAGAACAAAAACAATCGAAATTATTGTGCCGATTCCAACCAGAACACGAACTTCTTCATACCTTGGAATCACGACGTCCATATCTACAATCAATGCTTATATTGGTGCCACCGCTACAGTAATTGTTAACAACCCTTATCATGTTACCACGACGTTGACTACATATTGGACAGGTTCAACAACTTCTACAACAACATCTATTGCTCAAACTGATTCCATTGATGAGGTTTATG

**>*CtrALS2229*_951**

ATGTTGCGTCTATCAATTACTTATTCCATTTTCACATTAATTAACGTTATAAATGCCAAGGTCCTCTCCGGCATATTCACCAGATTTGTTTCATTAACACAATCATCTTATAATTCGTACAGTTTCGACGGTCCAATGCTGACAACTTGGATTGCAGCTCTAGGGTGGGAAATCAATGGAACAAAAGCAAAACCAGGTGATACATTTACTTTAGAGATGCCCTGTGTTTATAAAATTTTTATAAATGAAGAATCTATTGACTTAATTGGAAATGATATTAGTTTAGCAACTTGTGAAGTCCACTCTGGTGAACATATATCCACAAATTCGTATTTGAATTGTGTTATGAATGATTCTTTAGACGAAAGAACCAATATTGATGGAATTTTGAGACTCCCTATTATGTTCAATGTTGGAGGTTCCGGTCAAGATACTGACTTAGATGCGGCATCATCGTGTTTCAATACTGATTCCAACATAATTGCTTTCAACAATGGCGACACAAGTATTTCCATTCAACGAAGCTTTTCAGTAAGAGCAATTTTTGATACTGTACCAATAAACTTTGTTCGGGTAGGAAAAACTATTAGTGAATTAGAGGTACTCATTGTTGCTCCAAATTGTCCACAAGGTTACACAAATGGTAGACTAGCAATTGCTGCTTCAGACCGTGACGTTATCATAAAATGCTCTACTATAACATCGGGATTTGCCAGTAAACTTAACAAGTGGAACCTTCCAGAAAATTTAGTACAACTTTACCACGGCAGTCTTTGTACTTCACGTCAATTTTCTATTAGTTATACTAATATTCCAGAAGGGTATCGTCCATTTCTACTGGTAACTTTAAGCAATGCTGTTAGTTCTTCATTTAACCTTCGTTACACGATTCAGAATACATGTGAAAAAGACACGTTTAATGATCAATCAAGATCAGTTTCCTGGAGAAAGTTAAACTATGGATCTATCGATTCATATGGTGGTATCATGATTCCCGTCACAAGAACTAGAACAGGCACCGCCTCAACTAGATTGGTCACTACACGACCTTTTGATCCTTCGACGCACAGAACAAAAACAATCGAAATTATTGTGCCGATTCCAACCAGAACACGAACTTCTTCATACCTTGGAATCACGACGTCCATATCTACAATCAATGCTTATATTGGTGCCACCGCTACAGTAATTGTTAACAACCCTTATCATGTTACCACGACGTTGACTACATATTGGACAGGTTCAACAACTTCTACAACAACATCTATTGCTCAAACTGATTCCATTGATGAGGTTTATG

**>*CtrALS2229*_952**

ATGTTGCGTCTATCAATTACTTATTCCATTTTCACATTAATTAACGTTATAAATGCCAAGGTCCTCTCCGGCATATTCACCAGATTTGTTTCATTAACACAATCATCTTATAATTCGTACAGTTTCGACGGTCCAATGCTGACAACTTGGATTGCAGCTCTAGGGTGGGAAATCAATGGAACAAAAGCAAAACCAGGTGATACATTTACTTTAGAGATGCCCTGTGTTTATAAAATTTTTATAAATGAAGAATCTATTGACTTAATTGGAAATGATATTAGTTTAGCAACTTGTGAAGTCCACTCTGGTGAACATATATCCACAAATTCGTATTTGAATTGTGTTATGAATGATTCTTTAGACGARAGAACCAATATTGATGGAATTTTGAGACTCCCTATTATGTTCAATGTTGGAGGTTCCGGTCAAGATACTGACTTAGATGCGGCATCATCGTGTTTCAATACTGATTCCAACATAATTGCTTTCAACAATGGCGACACAAGTATTTCCATTCAACGAAGCTTTTCAGTAAGAGCAATTTTTGATACTGTACCAATWAACTTTGTTCGGGTAGGAAAAACTAYTAGTGAATTAGAGGTACTCATTGTTGCTCCAAATTGTCCACAAGGTTACACAAATGGTAGACTAGCAATTGCTGCTTCAGACCGTGACGTTATCATAAAATGCTCTACTATAACATCGGGATTTGCCAGTAAACTTAACAAGTGGAACCTTCCAGAAAATTTAGTACAACTTTACCACGGCAGTCTTTGTACTTCACGTCAATTTTCTATTAGTTATACTAATATTCCAGAAGGGTATCGTCCATTTCTACTGGTAACTTTAAGCAATGCTGTTAGTTCTTCATTTAACCTTCGTTACACGATTCAGAATACATGTGAAAAAGACACGTTTAATGATCAATCAAGATCAGTTTCCTGGAGAAAGTTAAACTATGGATCTMTCGATTCATATGGTGGTATCATGATTCCCGTCACAAGAACTAGAACAGGCACCGCYTCAACTAGRTTGGTCACTACACGACCTTTTGATCCTTCGACGCACAGAACAAAAACAATCGAAATTATTGTGCCGATTCCAACCAGAACACGAACTTCTTCATACCTTGGAATCACGACGTCCATATCTACAATCAATGCTTATATTGGTGCCACCGCTACAGTAATTGTTAACAACCCTTATCATGTYACCACGACGTTGACTACATATTGGACAGGTTCAACAACTTCTACAACAACATCTATTGCTCAAACTGATTCCATTGATGAGGTTTATG

**>*CtrALS2229*_1019**

ATGTTGCGTCTATCAATTACTTATTCCATTTTCACATTAATTAACGTTATAAATGCCAAGGTCCTCTCCGGCATATTCACCAGATTTGTTTCATTAACACAATCATCTTATAATTCGTACAGTTTCGACGGTCCAATGCTGACAACTTGGATTGCAGCTCTAGGGTGGGAAATCAATGGAACAAAAGCAAAACCAGGTGATACATTTACTTTAGAGATGCCCTGTGTTTATAAAATTTTTATAAATGAAGAATCTATTGACTTAATTGGAAATGATATTAGTTTAGCAACTTGTGAAGTCCACTCTGGTGAACATATATCCACAAATTCGTATTTGAATTGTGTTATGAATGATTCTTTAGACGAAAGAACCAATATTGATGGAATTTTGAGACTCCCTATTATGTTCAATGTTGGAGGTTCCGGTCAAGATACTGACTTAGATGCGGCATCATCGTGTTTCAATACTGATTCCAACATAATTGCTTTCAACAATGGCGACACAAGTATTTCCATTCAACGAAGCTTTTCAGTAAGAGCAATTTTTGATACTGTACCAATAAACTTTGTTCGGGTAGGAAAAACTATTAGTGAATTAGAGGTACTCATTGTTGCTCCAAATTGTCCACAAGGTTACACAAATGGTAGACTAGCAATTGCTGCTTCAGACCGTGACGTTATCATAAAATGCTCTACTATAACATCGGGATTTGCCAGTAAACTTAACAAGTGGAACCTTCCAGAAAATTTAGTACAACTTTACCACGGCAGTCTTTGTACTTCACGTCAATTTTCTATTAGTTATACTAATATTCCAGAAGGGTATCGTCCATTTCTACTGGTAACTTTAAGCAATGCTGTTAGTTCTTCATTTAACCTTCGTTACACGATTCAGAATACATGTGAAAAAGACACGTTTAATGATCAATCAAGATCAGTTTCCTGGAGAAAGTTAAACTATGGATCTATCGATTCATATGGTGGTATCATGATTCCCGTCACAAGAACTAGAACAGGCACCGCCTCAACTAGATTGGTCACTACACGACCTTTTGATCCTTCGACGCACAGAACAAAAACAATCGAAATTATTGTGCCGATTCCAACCAGAACACGAACTTCTTCATACCTTGGAATCACGACGTCCATATCTACAATCAATGCTTATATTGGTGCCACCGCTACAGTAATTGTTAACAACCCTTATCATGTTACCACGACGTTGACTACATATTGGACAGGTTCAACAACTTCTACAACAACATCTATTGCTCAAACTGATTCCATTGATGAGGTTTATG

**>*CtrALS2229*_1020**

ATGTTGCGTCTATCAATTACTTATTCCATTTTCACATTAATTAACGTTATAAATGCCAAGGTCCTCTCCGGCATATTCACCAGATTTGTTTCATTAACACAATCATCTTATAATTCGTACAGTTTCGACGGTCCAATGCTGACAACTTGGATTGCAGCTCTAGGGTGGGAAATCAATGGAACAAAAGCAAAACCAGGTGATACATTTACTTTAGAGATGCCCTGTGTTTATAAAATTTTTATAAATGAAGAATCTATTGACTTAATTGGAAATGATATTAGTTTAGCAACTTGTGAAGTCCACTCTGGTGAACATATATCCACAAATTCGTATTTGAATTGTGTTATGAATGATTCTTTAGACGAAAGAACCAATATTGATGGAATTTTGAGACTCCCTATTATGTTCAATGTTGGAGGTTCCGGTCAAGATACTGACTTAGATGCGGCATCATCGTGTTTCAATACTGATTCCAACATAATTGCTTTCAACAATGGCGACACAAGTATTTCCATTCAACGAAGCTTTTCAGTAAGAGCAATTTTTGATACTGTACCAATAAACTTTGTTCGGGTAGGAAAAACTATTAGTGAATTAGAGGTACTCATTGTTGCTCCAAATTGTCCACAAGGTTACACAAATGGTAGACTAGCAATTGCTGCTTCAGACCGTGACGTTATCATAAAATGCTCTACTATAACATCGGGATTTGCCAGTAAACTTAACAAGTGGAACCTTCCAGAAAATTTAGTACAACTTTACCACGGCAGTCTTTGTACTTCACGTCAATTTTCTATTAGTTATACTAATATTCCAGAAGGGTATCGTCCATTTCTACTGGTAACTTTAAGCAATGCTGTTAGTTCTTCATTTAACCTTCGTTACACGATTCAGAATACATGTGAAAAAGACACGTTTAATGATCAATCAAGATCAGTTTCCTGGAGAAAGTTAAACTATGGATCTATCGATTCATATGGTGGTATCATGATTCCCGTCACAAGAACTAGAACAGGCACCGCCTCAACTAGATTGGTCACTACACGACCTTTTGATCCTTCGACGCACAGAACAAAAACAATCGAAATTATTGTGCCGATTCCAACCAGAACACGAACTTCTTCATACCTTGGAATCACGACGTCCATATCTACAATCAATGCTTATATTGGTGCCACCGCTACAGTAATTGTTAACAACCCTTATCATGTTACCACGACGTTGACTACATATTGGACAGGTTCAACAACTTCTACAACAACATCTATTGCTCAAACTGATTCCATTGATGAGGTTTATG

**>*CtrALS2229*_1021**

ATGTTGCGTCTATCAATTACTTATTCCATTTTCACATTAATTAACGTTATAAATGCCAAGGTCCTCTCCGGCATATTCACCAGATTTGTTTCATTAACACAATCATCTTATAATTCGTACAGTTTCGACGGTCCAATGCTGACAACTTGGATTGCAGCTCTAGGGTGGGAAATCAATGGAACAAAAGCAAAACCAGGTGATACATTTACTTTAGAGATGCCCTGTGTTTATAAAATTTTTATAAATGAAGAATCTATTGACTTAATTGGAAATGATATTAGTTTAGCAACTTGTGAAGTCCACTCTGGTGAACATATATCCACAAATTCGTATTTGAATTGTGTTATGAATGATTCTTTAGACGARAGAACCAATATTGATGGAATTTTGAGACTCCCTATTATGTTCAATGTTGGAGGTTCCGGTCAAGATACTGACTTAGATGCGGCATCATCGTGTTTCAATACTGATTCCAACATAATTGCTTTCAACAATGGCGACACAAGTATTTCCATTCAACGAAGCTTTTCAGTAAGAGCAATTTTTGATACTGTACCAATWAACTTTGTTCGGGTAGGAAAAACTAYTAGTGAATTAGAGGTACTCATTGTTGCTCCAAATTGTCCACAAGGTTACACAAATGGTAGACTAGCAATTGCTGCTTCAGACCGTGACGTTATCATAAAATGCTCTACTATAACATCGGGATTTGCCAGTAAACTTAACAAGTGGAACCTTCCAGAAAATTTAGTACAACTTTACCACGGCAGTCTTTGTACTTCACGTCAATTTTCTATTAGTTATACTAATATTCCAGAAGGGTATCGTCCATTTCTACTGGTAACTTTAAGCAATGCTGTTAGTTCTTCATTTAACCTTCGTTACACGATTCAGAATACATGTGAAAAAGACACGTTTAATGATCAATCAAGATCAGTTTCCTGGAGAAAGTTAAACTATGGATCTMTCGATTCATATGGTGGTATCATGATTCCCGTCACAAGAACTAGAACAGGCACCGCYTCAACTAGRTTGGTCACTACACGACCTTTTGATCCTTCGACGCACAGAACAAAAACAATCGAAATTATTGTGCCGATTCCAACCAGAACACGAACTTCTTCATACCTTGGAATCACGACGTCCATATCTACAATCAATGCTTATATTGGTGCCACCGCTACAGTAATTGTTAACAACCCTTATCATGTYACCACGACGTTGACTACATATTGGACAGGTTCAACAACTTCTACAACAACATCTATTGCTCAAACTGATTCCATTGATGAGGTTTATG

**>*CtrALS2229*_3242**

ATGTTGCGTCTATCAATTACTTATTCCATTTTCACATTAATTAACGTTATAAATGCCAAGGTCCTCTCCGGCATATTCACCAGATTTGTTTCATTAACACAATCATCTTATAATTCGTACAGTTTCGACGGTCCAATGCTGACAACTTGGATTGCAGCTCTAGGGTGGGAAATCAATGGAACAAAAGCAAAACCAGGTGATACATTTACTTTAGAGATGCCCTGTGTTTATAAAATTTTTATAAATGAAGAATCTATTGACTTAATTGGAAATGATATTAGTTTAGCAACTTGTGAAGTCCACTCTGGTGAACATATATCCACAAATTCGTATTTGAATTGTGTTATGAATGATTCTTTAGACGAAAGAACCAATATTGATGGAATTTTGAGACTCCCTATTATGTTCAATGTTGGAGGTTCCGGTCAAGATACTGACTTAGATGCGGCATCATCGTGTTTCAATACTGATTCCAACATAATTGCTTTCAACAATGGCGACACAAGTATTTCCATTCAACGAAGCTTTTCAGTAAGAGCAATTTTTGATACTGTACCAATAAACTTTGTTCGGGTAGGAAAAACTATTAGTGAATTAGAGGTACTCATTGTTGCTCCAAATTGTCCACAAGGTTACACAAATGGTAGACTAGCAATTGCTGCTTCAGACCGTGACGTTATCATAAAATGCTCTACTATAACATCGGGATTTGCCAGTAAACTTAACAAGTGGAACCTTCCAGAAAATTTAGTACAACTTTACCACGGCAGTCTTTGTACTTCACGTCAATTTTCTATTAGTTATACTAATATTCCAGAAGGGTATCGTCCATTTCTACTGGTAACTTTAAGCAATGCTGTTAGTTCTTCATTTAACCTTCGTTACACGATTCAGAATACATGTGAAAAAGACACGTTTAATGATCAATCAAGATCAGTTTCCTGGAGAAAGTTAAACTATGGATCTATCGATTCATATGGTGGTATCATGATTCCCGTCACAAGAACTAGAACAGGCACCGCCTCAACTAGATTGGTCACTACACGACCTTTTGATCCTTCGACGCACAGAACAAAAACAATCGAAATTATTGTGCCGATTCCAACCAGAACACGAACTTCTTCATACCTTGGAATCACGACGTCCATATCTACAATCAATGCTTATATTGGTGCCACCGCTACAGTAATTGTTAACAACCCTTATCATGTYACCACGACGTTGACTACATATTGGACAGGTTCAACAACTTCTACAACAACATCTATTGCTCAAACTGATTCCATTGATGAGGTTTATG

***CtrALS2293* (Amplified with primers Ctr2293NT-F and Ctr2293NT-R)**

**>*CtrALS2293*_MYA-3404**

ATGTTTCTATTACAAGCAGTAATTTTATACTGTTCATTCATTGTTACTGCGGTTGCTAAAGAAATTTCTGGTGTATTTACTGGTTTTGAATCCTTAACGTGGGATAAAGCTGCTAATTACGGCTTTCAGGGTCCACAATATCCAACATGGAATGCCGTTCTTGATTGGTCGTTAGATGGTACAACCACGTCCCCAGGTGACACGTTCACTTTGATCATGCCTTGTGTCTTCAAATTCACTACATCTGCCACTTCTATCGATTTAAGAGCTAATGGTATTACATATGCCACATGTGATCTTCATGCCGGTGAAGAGTTTACTACTTACTCCAGTTTGACTTGTACTGTTACYGATTCGTTAAGTTCTGTYCATSAAGCWRYGGGTACAGTCACAATTCCTTTGGCATTTAACGTTGGTGGTTCTGGTTCTTCTGTTGATATTGCTGATTCTACTTGTTTCACTGCTGGCACAAACACTGTTACTTTCCAAGATGGTGATACATCAATCAGTACCCAAGCCTATTTTGCTGCAGCTACTGAATCTTCTTCTGGTCTCCTTTACTTCCAAAGAAGTGTTCCTTCATTGAACAAGCTTAATGCACTTGCAATTCTCCCAGATTGTCCCAATGGTTACACTTCTGGTACTCTTGGTTTCTCATCTTCTAATTCCAGATTTCTGATTGATTGTTCCAGTGCTGAAGCTTACATTACAAATCTTTTAAATTCCTGGAACTACCCAACTTCAGCGGATTCCTTTTCTTATACCCAAACTTGTACTTCCAAAAGTTTTCAAATAACATTCAATAATATTCCAGCYGGTTATCGTCCATATATTGCTGCATTGGTTCAAGCTCCATCTTCGGATTATGCTATACAATATACTGCAAAATACAGGTGTGAAGGTTCTGTCCAAAGAGATGATTCACAAAAGATATCTTGGGCCGGTTACACAAATAGTGACCCAGATTCAAATGGTGCTGTAGTTGTTCTTACAACTATTACAGGTACTCAGTCCAATACTATTGTTACCACATTGCCTTTCAACCCGACTGCTGATCACACAAAAACGATCGAAGTGATTGTTCCAATTCCAACAGTAACTACTACCACTTCCTATATTGGAGTTACTACTTCTTATACCACAATTACTGGTACTATTGGTGGAACGGCTACTGTTATTGTAGATGAACCGTATCATACTACTACCACTGTTACTAGTCCCTGGACTGGAA

**>*CtrALS2293*_951**

ATGTTTCTATTACAAGCAGTAATTTTATACTGTTCATTCATTGTTACTGCGGTTGCTAAAGAAATTTCTGGTGTATTTACTGGTTTTGAATCCTTAACGTGGGATAAAGCTGCTAATTACGGCTTTCAGGGTCCACAATATCCAACATGGAATGCCGTTCTTGATTGGTCGTTAGATGGTACAACCACGTCCCCAGGTGACACGTTCACTTTGATCATGCCTTGTGTCTTCAAATTCACTACATCTGCCACTTCTATCGATTTAAGAGCTAATGGTATTACATATGCCACATGTGATCTTCATGCCGGTGAAGAGTTTACTACTTACTCCAGTTTGACTTGTACTGTTACCGATTCGTTAAGTTCTGTTCATGAAGCTATGGGTACAGTCACAATTCCTTTGGCATTTAACGTTGGTGGTTCTGGTTCTTCTGTTGATATTGCTGATTCTACTTGTTTCACTGCTGGCACAAAYACTGTTACTTTCCAAGATGGTGATACATCAATCAGTACCCAAGCCTATTTTGCTGCAGCTACTGRATCTTCTTCTGRTCTCCTTTACTTCCAAAGARKTGTTCCTTCATTGAACAAGCTTAATGCACTTGYAATTCTCCCAGATTGTCCMAATGGTTACACTTCTGGTACTCTTGGTTTCTCATMTTCYAATTCCAGATTTCTGATTGATTGTTCCAGTGCTGAAGCTTACATTACAAATCTTTTAAATTCCTGGAACTACCCAACTTCAGCGGATTCCTTTTCTTATACCCAAACTTGTACTTCCAAAAGTTTTCAAATAACATTCAATAATATTCCAGCCGGYTATCGTCCATATATTGCTGCATTGGTTCAAGCTCCATCTTCGGATTATGCTATACAATATACTGCAAAATACAGGTGTGAAGGTTCTGTCCAAAGAGATGATTCACAAAAGATATCTTGGGCCGGTTACACAAATAGTGACCCAGATTCAAATGGTGCTGTAGTTGTTCTTACAACTATTACAGGTACTCAGTCCAATACTATTGTTACCACATTGCCTTTCAACCCGACTGCTGATCACACAAAAACGATCGAAGTGATTGTTCCAATTCCAACAGTAACTACTACCACTTCCTATATTGGAGTTACTACTTCTTATACCACAATTACTGGTACTATTGGTGGAACGGCTACTGTTATTGTAGATGAACCGTATCATACTACTACCACTGTTACTAGTCCCTGGACTGGAA

**>*CtrALS2293*_952**

ATGTTTCTATTACAAGCAGTAATTTTATACTGTTCATTCATTGTTACTGCGGTTGCTAAAGAAATTTCTGGTGTATTTACTGGTTTTGAATCCTTAACGTGGGATAAAGCTGCTAATTACGGCTTTCAGGGTCCACAATATCCAACATGGAATGCCGTTCTTGATTGGTCGTTAGATGGTACAACCACGTCCCCAGGTGACACGTTCACTTTGATCATGCCTTGTGTCTTCAAATTCACTACATCTGCCACTTCTATCGATTTAAGAGCTAATGGTATTACATATGCCACATGTGATCTTCATGCCGGTGAAGAGTTTACTACTTACTCCAGTTTGACTTGTACTGTTACCGATTCGTTAAGTTCTGTTCATGAAGCTATGGGTACAGTCACAATTCCTTTGGCATTTAACGTTGGTGGTTCTGGTTCTTCTGTTGATATTGCTGATTCTACTTGTTTCACTGCTGGCACAAACACTGTTACTTTCCAAGATGGTGATACATCAATCAGTACCCAAGCCTATTTTGCTGCAGCTACTGAATCTTCTTCTGGTCTCCTTTACTTCCAAAGAAGTGTTCCTTCATTGAACAAGCTTAATGCACTTGCAATTCTCCCAGATTGTCCCAATGGTTACACTTCTGGTACTCTTGGTTTCTCATCTTCTAATTCCAGATTTCTGATTGATTGTTCCAGTGCTGAAGCTTACATTACAAATCTTTTAAATTCCTGGAACTACCCAACTTCAGCGGATTCCTTTTCTTATACCCAAACTTGTACTTCCAAAAGTTTTCAAATAACATTCAATAATATTCCAGCCGGTTATCGTCCATATATTGCTGCATTGGTTCAAGCTCCATCTTCGGATTATGCTATACAATATACTGCAAAATACAGGTGTGAAGGTTCTGTCCAAAGAGATGATTCACAAAAGATATCTTGGGCCGGTTACACAAATAGTGACCCAGATTCAAATGGTGCTGTAGTTGTTCTTACAACTATTACAGGTACTCAGTCCAATACTATTGTTACCACATTGCCTTTCAACCCGACTGCTGATCACACAAAAACGATCGAAGTGATTGTTCCAATTCCAACAGTAACTACTACCACTTCCTATATTGGAGTTACTACTTCTTATACCACAATTACTGGTACTATTGGTGGAACGGCTACTGTTATTGTAGATGAACCGTATCATACTACTACCACTGTTACTAGTCCCTGGACTGGAA

**>*CtrALS2293*_1019**

ATGTTTCTATTACAAGCAGTAATTTTATACTGTTCATTCATTGTTACTGCGGTTGCTAAAGAAATTTCTGGTGTATTTACTGGTTTTGAATCCTTAACGTGGGATAAAGCTGCTAATTACGGCTTTCAGGGTCCACAATATCCAACATGGAATGCCGTTCTTGATTGGTCGTTAGATGGTACAACCACGTCCCCAGGTGACACGTTCACTTTGATCATGCCTTGTGTCTTCAAATTCACTACATCTGCCACTTCTATCGATTTAAGAGCTAATGGTATTACATATGCCACATGTGATCTTCATGCCGGTGAAGAGTTTACTACTTACTCCAGTTTGACTTGTACTGTTACCGATTCGTTAAGTTCTGTTCATGAAGCTATGGGTACAGTCACAATTCCTTTGGCATTTAACGTTGGTGGTTCTGGTTCTTCTGTTGATATTGCTGATTCTACTTGTTTCACTGCTGGCACAAACACTGTTACTTTCCAAGATGGTGATACATCAATCAGTACCCAAGCCTATTTTGCTGCAGCTACTGAATCTTCTTCTGGTCTCCTTTACTTCCAAAGAAGTGTTCCTTCATTGAACAAGCTTAATGCACTTGCAATTCTCCCAGATTGTCCCAATGGTTACACTTCTGGTACTCTTGGTTTCTCATCTTCTAATTCCAGATTTCTGATTGATTGTTCCAGTGCTGAAGCTTACATTACAAATCTTTTAAATTCCTGGAACTACCCAACTTCAGCGGATTCCTTTTCTTATACCCAAACTTGTACTTCCAAAAGTTTTCAAATAACATTCAATAATATTCCAGCCGGCTATCGTCCATATATTGCTGCATTGGTTCAAGCTCCATCTTCGGATTATGCTATACAATATACTGCAAAATACAGGTGTGAAGGTTCTGTCCAAAGAGATGATTCACAAAAGATATCTTGGGCCGGTTACACAAATAGTGACCCAGATTCAAATGGTGCTGTAGTTGTTCTTACAACTATTACAGGTACTCAGTCCAATACTATTGTTACCACATTGCCTTTCAACCCGACTGCTGATCACACAAAAACGATCGAAGTGATTGTTCCAATTCCAACAGTAACTACTACCACTTCCTATATTGGAGTTACTACTTCTTATACCACAATTACTGGTACTATTGGTGGAACGGCTACTGTTATTGTAGATGAACCGTATCATACTACTACCACTGTTACTAGTCCCTGGACTGGAA

**>*CtrALS2293*_1020**

ATGTTTCTATTACAAGCAGTAATTTTATACTGTTCATTCATTGTWACTGCGGTTGCTAAAGAAATTTCTGGTGTATTTACTGGTTTTGAATCCTTAACGTGGGATAAAGCTGCTAATTACGGCTTTCAGGGTCCACAATATCCAACATGGAATGCCGTTCTTGATTGGTCGTTAGATGGTACAACCACGTCCCCAGGTGACACGTTCACTTTGATCATGCCTTGTGTCTTCAAATTCACTACATCTGCCACTTCTATCGATTTAAGAGCTAATGGTATTACATATGCCACATGTGATCTTCATGCCGGTGAAGAGTTTACTACTTACTCCAGTTTGACTTGTACTGTTACCGATTCGTTAAGTTCTGTTCATGAAGCTATGGGTACAGTCACAATTCCTTTGGCATTTAACGTTGGTGGTTCTGGTTCTTCTGTTGATATTGCTGATTCTACTTGTTTCACTGCTGGCACAAACACTGTTACTTTCCAAGATGGTGATACATCAATCAGTACCCAAGCCTATTTTGCTGCAGCTACTGGATCTTCTTCTGATCTCCTTTACTTCCAAAGAGTTGTTCCTTCATTGAACAAGCTTAATGCACTTGTAATTCTCCCAGATTGTCCAAATGGTTACACTTCTGGTACTCTTGGTTTCTCATCTTCCAATTCCAGATTTCTGATTGATTGTTCCAGTGCTGAAGCTTACATTACAAATCTTTTAAATTCCTGGAACTACCCAACTTCAGCGGATTCCTTTTCTTATACCCAAACTTGTACTTCCAAAAGTTTTCAAATAACATTCAATAATATTCCAGCCGGTTATCGTCCATATATTGCTGCATTGGTTCAAGCTCCATCTTCGGATTATGCTATACAATATACTGCAAAATACAGGTGTGAAGGTTCTGTCCAAAGAGATGATTCACAAAAGATATCTTGGGCCGGTTACACAAATAGTGACCCAGATTCAAATGGTGCTGTAGTTGTTCTTACAACTAGTACAGGTACTCAGTCCAATACTATTGTTACCACATTGCCTTTCAACCCGACTGTTGATCACACAAAAACGATCGAAGTGATTGTTCCAATTCCAACAGTAACTACTACCACTTCCTATATTGGAGTTACTACTTCTTATACCACAATTACTGGTACTATTGGTGGAACGGCTACTGTTATTGTAGATGAACCGTATCATACTACTACCACTGTTACTAGTCCCTGGACTGGAA

**>*CtrALS2293*_1021**

ATGTTTCTATTACAAGCAGTAATTTTATACTGTTCATTCATTGTTACTGCGGTTGCTAAAGAAATTTCTGGTGTATTTACTGGTTTTGAATCCTTAACGTGGGATAAAGCTGCTAATTACGGCTTTCAGGGTCCACAATATCCAACATGGAATGCCGTTCTTGATTGGTCGTTAGATGGTACAACCACGTCCCCAGGTGACACGTTCACTTTGATCATGCCTTGTGTCTTCAAATTCACTACATCTGCCACTTCTATCGATTTAAGAGCTAATGGTATTACATATGCCACATGTGATCTTCATGCCGGTGAAGAGTTTACTACTTACTCCAGTTTGACTTGTACTGTTACYGATTCGTTAAGTTCTGTYCATSAAGCWRYGGGTACAGTCACAATTCCTTTGGCATTTAACGTTGGTGGTTCTGGTTCTTCTGTTGATATTGCWGATTCTACTTGTTTCACTGCTGGYACAAACACTGTTACTTTCCAAGATGGTGATACATCAATCAGTACCCAAGCCTATTTTGCTGCAGCTACTGGATCTTCTTCTGATCTCCTTTACTTCCAAAGAGTTGTTCCTTCATTGAACAAGCTTAATGCACTTGTAATTCTCCCAGATTGTCCAAATGGTTACACTTCTGGTACTCTTGGTTTCTCATCTTCCAATTCCAGATTTCTGATTGATTGTTCCAGTGCTGAAGCTTACATTACAAATCTTTTAAATTCCTGGAACTACCCAACTTCAGCGGATTCCTTTTCTTATACCCAAACTTGTACTTCCAAAAGTTTTCAAATAACATTCAATAATATTCCAGCCGGTTATCGTCCATATATTGCTGCATTGGTTCAAGCTCCATCTTCGGATTATGCTATACAATATACTGCAAAATACAGGTGTGAAGGTTCTGTCCAAAGAGATGATTCACAAAAGATATCTTGGGCCGGTTACACAAATAGTGACCCAGATTCAAATGGTGCTGTAGTTGTTCTTACAACTAKTACAGGTACTCAGTCCAATACTATTGTTACCACATTGCCTTTCAACCCGACTGTTGATCACACAAAAACGATCGAAGTGATTGTTCCAATTCCAACAGTAACTACTACCACTTCCTATATTGGAGTTACTACTTCTTATACCACAATTACTGGTACTATTGGTGGAACGGCTACTGTTATTGTAGATGAACCGTATCATACTACTACCACTGTTACTAGTCCCTGGACTGGAA

**>*CtrALS2293*_3242**

ATGTTTCTATTACAAGCAGTAATTTTATACTGTTCATTCATTGTTACTGCGGTTGCTAAAGAAATTTCTGGTGTATTTACTGGTTTTGAATCCTTAACGTGGGATAAAGCTGCTAATTACGGCTTTCAGGGTCCACAATATCCAACATGGAATGCCGTTCTTGATTGGTCGTTAGATGGTACAACCACGTCCCCAGGTGACACGTTCACTTTGATCATGCCTTGTGTCTTCAAATTCACTACATCTGCCACTTCTATCGATTTAAGAGCTAATGGTATTACATATGCCACATGTGATCTTCATGCCGGTGAAGAGTTTACTACTTACTCCAGTTTGACTTGTACTGTTACCGATTCGTTAAGTTCTGTTCATGAAGCTATGGGTACAGTCACAATTCCTTTGGCATTTAACGTTGGTGGTTCTGGTTCTTCTGTTGATATTGCTGATTCTACTTGTTTCACTGCTGGCACAAAYACTGTTACTTTCCAAGATGGTGATACATCAATCAGTACCCAAGCCTATTTTGCTGCAGCTACTGRATCTTCTTCTGRTCTCCTTTACTTCCAAAGARKTGTTCCTTCATTGAACAAGCTTAATGCACTTGYAATTCTCCCAGATTGTCCMAATGGTTACACTTCTGGTACTCTTGGTTTCTCATMTTCYAATTCCAGATTTCTGATTGATTGTTCCAGTGCTGAAGCTTACATTACAAATCTTTTAAATTCCTGGAACTACCCAACTTCAGCGGATTCCTTTTCTTATACCCAAACTTGTACTTCCAAAAGTTTTCAAATAACATTCAATAATATTCCAGCCGGYTATCGTCCATATATTGCTGCATTGGTTCAAGCTCCATCTTCGGATTATGCTATACAATATACTGCAAAATACAGGTGTGAAGGTTCTGTCCAAAGAGATGATTCACAAAAGATATCTTGGGCCGGTTACACAAATAGTGACCCAGATTCAAATGGTGCTGTAGTTGTTCTTACAACTAKTACAGGTACTCAGTCCAATACTATTGTTACCACATTGCCTTTCAACCCGACTGYTGATCACACAAAAACGATCGAAGTGATTGTTCCAATTCCAACAGTAACTACTACCACTTCCTATATTGGAGTTACTACTTCTTATACCACAATTACTGGTACTATTGGTGGAACGGCTACTGTTATTGTAGATGAACCGTATCATACTACTACCACTGTTACTAGTCCCTGGACTGGAA

***CtrALS3786* (Amplified with primers Ctr3786NT-F and Ctr3786NT-R)**

**>*CtrALS3786*_MYA-3404**

ATGATTTTTTCTGAGTTTTTAATATTGTCGCTAACATTCATTGCTACTTCAGTTGCTAAAGAAATTTCCGGTGTGTTCATAGGATTTGAATCCTTAACTTGGGATAAAGCTGGTGATTATGCTTATCAAGGTCCTCAATATCCAACATGGAATGCCGTGCTTGATTGGTCGTTAGATGGCACAACAACTTCCCCAGGTGATACGTTCACTTTGATCATGCCCTGTGTCTTCAAGTTCACTACGTCTGCCACTTCTGTTGATTTGACTGCCAATGGTATTACATATGCCACATGTGATCTTCATGCCGGTGAAGAGTTTACTACTTACTCTAGTTTGACTTGTACTGTTACTGATTCGTTAAGTTCTGTTCATGAAGCTATGGGTACAGTCACAATTCCTTTGGCATTTAACGTTGGTGGTTCTGGTTCTTCTGTTGATATTGCTGATTCTACTTGTTTCACTGCTGGCACAAACACTGTTACTTTCCAAGATGGTGATACATCAATCAGTACCCAAGCCTATTTTGCTGCAGCTACTGGATCTTCTTCTGATCTCCTTTACTTCCAAAGAGTTGTTCCTTCATTGAACAAGCTTAATGCACTTGTAATTCTCCCAGATTGTCCAAATGGTTACACTTCTGGTACTCTTGGTTTCTCATCTTCCAATTCCAGATTTCTGATTGATTGTTCCAGTGCTGAAGCTTACATTACAAATCTTTTAAATTCCTGGAACAAGCCAACTACGGCTGATTCTTTTACATATACCCAAACTTGTACTTCCAAAAGTTTTCAAATAACATTCAATAATATTCCAGCTGGTTATCGTCCATATATTGCTGCATTGGTTCAAGCTCCATCTTCGGATTATGCTATACAATATACTGCAAAATATCAATGTACTGGATCTACCCAAAAAGATATTACGAAATCGGTCACTTGGTCAGGCTATACAAATAGTGACACAGATTCAAATGGTGCTGTAGTTGTTCTTACAACTATTACAGGTACTCAGTCCAATACTATTGTTACCACATTGCCTTTCAACCCGACTGTTGATCACACAAAAACGATCGAAGTGATTGTTCCAATTCCAACAGTAACAACTACCACTTCCTACATTGGAGTTACTACTTCTTATACCACAATTACTGGTACTATTGGTGGAACGGCTACTGTTATTGTMGATGAACCGTATCATACTACTACYACTGTTACTAGTCCCTGGACTGGAACCTTCACCACTTCAACTACTGTCATTGCYTCTACTGACTCGGT

**>*CtrALS3786*_951**

ATGATTTTTTCTGAGTTTTTAATATTGTCGCTAACATTCATTGCTACTTCAGTTGCTAAAGAAATTTCCGGTGTGTTCATAGGATTTGAATCCTTAACTTGGGATAAAGCTGGTGATTATGCTTATCAAGGTCCTCAATATCCAACATGGAATGCCGTGCTTGATTGGTCGTTAGATGGCACAACAACTTCCCCAGGTGATACGTTCACTTTGATCATGCCCTGTGTCTTCAAGTTCACTACGTCTGCCACTTCTGTTGATTTGACTGCCAATGGTATTACATATGCCACATGTGATCTTCATGCCGGTGAAGAGTTTACTACTTACTCTAGTTTGACTTGTACTGTTACTGATTCGTTAAGTTCTGTTCATGAAGCTATGGGTACAGTCACAATTCCTTTGGCATTTAACGTTGGTGGTTCTGGTTCTTCTGTTGATATTGCTGATTCTACTTGTTTCACTGCTGGCACAAACACTGTTACTTTCCAAGATGGTGATACATCAATCAGTACCCAAGCCTATTTTGCTGCAGCTACTGGATCTTCTTCTGATCTCCTTTACTTCCAAAGAGTTGTTCCTTCATTGAACAAGCTTAATGCACTTGTAATTCTCCCAGATTGTCCAAATGGTTACACTTCTGGTACTCTTGGTTTCTCATCTTCCAATTCCAGATTTCTGATTGATTGTTCCAGTGCTGAAGCTTACATTACAAATCTTTTAAATTCCTGGAACAAGCCAACTACGGCTGATTCTTTTACATATACCCAAACTTGTACTTCCAAAAGTTTTCAAATAACATTCAATAATATTCCAGCTGGTTATCGTCCATATATTGCTGCATTGGTTCAAGCTCCATCTTCGGATTATGCTATACAATATACTGCAAAATATCAATGTACTGGATCTACCCAAAAAGATATTACGAAATCGGTCACTTGGTCAGGCTATACAAATAGTGACACAGATTCAAATGGTGCTGTAGTTGTTCTTACAACTATTACAGGTACTCAGTCCAATACTATTGTTACCACATTGCCTTTCAACCCGACTGTTGATCACACAAAAACGATCGAAGTGATTGTTCCAATTCCAACAGTAACAACTACCACTTCCTACATTGGAGTTACTACTTCTTATACCACAATTACTGGTACTATTGGTGGAACGGCTACTGTTATTGTCGATGAACCGTATCATACTACTACCACTGTTACTAGTCCCTGGACTGGAACCTTCACCACTTCAACTACTGTCATTGCTTCTACTGACTCGGT

**>*CtrALS3786*_952**

ATGATTTTTTCTGAGTTTTTAATATTGTCGCTAACATTCATTGCTACTTCAGTTGCTAAAGAAATTTCCGGTGTGTTCATAGGATTTGAATCCTTAACTTGGGATAAAGCTGGTGATTATGCTTATCAAGGTCCTCAATATCCAACATGGAATGCCGTGCTTGATTGGTCGTTAGATGGCACAACAACTTCCCCAGGTGATACGTTCACTTTGATCATGCCCTGTGTCTTCAAGTTCACTACGTCTGCCACTTCTGTTGATTTGACTGCCAATGGTATTACATATGCCACATGTGATCTTCATGCCGGTGAAGAGTTTACTACTTACTCTAGTTTGACTTGTACTGTTACTGATTCGTTAAGTTCTGTTCATGAAGCTATGGGTACAGTCACAATTCCTTTGGCATTTAACGTTGGTGGTTCTGGTTCTTCTGTTGATATTGCTGATTCTACTTGTTTCACTGCTGGCACAAACACTGTTACTTTCCAAGATGGTGATACATCAATCAGTACCCAAGCCTATTTTGCTGCAGCTACTGGATCTTCTTCTGATCTCCTTTACTTCCAAAGAGTTGTTCCTTCATTGAACAAGCTTAATGCACTTGTAATTCTCCCAGATTGTCCAAATGGTTACACTTCTGGTACTCTTGGTTTCTCATCTTCCAATTCCAGATTTCTGATTGATTGTTCCAGTGCTGAAGCTTACATTACAAATCTTTTAAATTCCTGGAACAAGCCAACTACGGCTGATTCTTTTACATATACCCAAACTTGTACTTCCAAAAGTTTTCAAATAACATTCAATAATATTCCAGCTGGTTATCGTCCATATATTGCTGCATTGGTTCAAGCTCCATCTTCGGATTATGCTATACAATATACTGCAAAATATCAATGTACTGGATCTACCCAAAAAGATATTACGAAATCGGTCACTTGGTCAGGCTATACAAATAGTGACACAGATTCAAATGGTGCTGTAGTTGTTCTTACAACTATTACAGGTACTCAGTCCAATACTATTGTTACCACATTGCCTTTCAACCCGACTGTTGATCACACAAAAACGATCGAAGTGATTGTTCCAATTCCAACAGTAACAACTACCACTTCCTACATTGGAGTTACTACTTCTTATACCACAATTACTGGTACTATTGGTGGAACGGCTACTGTTATTGTCGATGAACCGTATCATACTACTACCACTGTTACTAGTCCCTGGACTGGAACCTTCACCACTTCAACTACTGTCATTGCTTCTACTGACTCGGT

**>*CtrALS3786*_1019**

ATGATTTTTTCTGAGTTTTTAATATTGTCGCTAACATTCATTGCTACTTCAGTTGCTAAAGAAATTTCCGGTGTGTTCATAGGATTTGAATCCTTAACTTGGGATAAAGCTGGTGATTATGCTTATCAAGGTCCTCAATATCCAACATGGAATGCCGTGCTTGATTGGTCGTTAGATGGCACAACAACTTCCCCAGGTGATACGTTCACTTTGATCATGCCCTGTGTCTTCAAGTTCACTACGTCTGCCACTTCTRTTGATTTGACTGCCAATGGTATTACATATGCCACATGTGATCTTCATGCCGGTGAAGAGTTTACTACTTACTCTAGTTTGACTTGTACTGTTACTGATTCGTTAAGTTCTGTTCATGAAGCTATGGGTACAGTCACAATTCCTTTGGCATTTAACGTTGGTGGTTCTGGTTCTTCTGTTGATATTGCTGATTCTACTTGTTTCACTGCTGGCACAAACACTGTTACTTTCCAAGATGGTGATACATCAATCAGTACCCAAGCCTATTTTGCTGCAGCTACTGGATCTTCTTCTGATCTCCTTTACTTCCAAAGAGTTGTTCCTTCATTGAACAAGCTTAATGCACTTGTAATTCTCCCAGATTGTCCAAATGGTTACACTTCTGGTACTCTTGGTTTCTCATCTTCCAATTCCAGATTTCTGATTGATTGTTCCAGTGCTGAAGCTTACATTACAAATCTTTTAAATTCCTGGAACAAGCCAACTACGGCTGATTCTTTTACATATACCCAAACTTGTACTTCCAAAAGTTTTCAAATAACATTCAATAATATTCCAGCTGGTTATCGTCCATATATTGCTGCATTGGTTCAAGCTCCATCTTCGGATTATGCTATACAATATACTGCAAAATATCAATGTACTGGATCTACCCAAAAAGATATTACGAAATCGGTCACTTGGTCAGGCTATACAAATAGTGACACAGATTCAAATGGTGCTGTAGTTGTTCTTACAACTATTACAGGTACTCAGTCCAATACTATTGTTACCACATTGCCTTTCAACCCGACTGTTGATCACACAAAAACGATCGAAGTGATTGTTCCAATTCCAACAGTAACAACTACCACTTCCTACATTGGAGTTACTACTTCTTATACCACAATTACTGGTACTATTGGTGGAACGGCTACTGTTATTGTCGATGAACCGTATCATACTACTACCACTGTTACTAGTCCCTGGACTGGAACCTTCACCACTTCAACTACTGTCATTGCTTCTACTGACTCGGT

**>*CtrALS3786*_1020**

ATGATTTTTTCTGAGTTTTTAATATTGTCGCTAACATTCATTGCTACTTCAGTTGCTAAAGAAATTTCCGGTGTGTTCATAGGATTTGAATCCTTAACTTGGGATAAAGCTGGTGATTATGCTTATCAAGGTCCTCAATATCCAACATGGAATGCCGTGCTTGATTGGTCGTTAGATGGCACAACAACTTCCCCAGGTGATACGTTCACTTTGATCATGCCCTGTGTCTTCAAGTTCACTACGTYTGCCACTTCTGTTGATTTGACTGCCAATGGTATTACATATGCCACATGTGATCTTCATGCCGGTGAAGAGTTTACTACTTACTCTAGTTTGACTTGTACTGTTACTGATTCGTTAAGTTCTGTTCATGAAGCTATGGGTACAGTCACAATTCCTTTGGCATTTAACGTTGGTGGTTCTGGTTCTTCTGTTGATATTGCTGATTCTACTTGTTTCACTGCTGGCACAAACACTGTTACTTTCCAAGATGGTGATACATCAATCAGTACCCAAGCCTATTTTGCTGCAGCTACTGGATCTTCTTCTGATCTCCTTTACTTCCAAAGAGTTGTTCCTTCATTGAACAAGCTTAATGCACTTGTAATTCTCCCAGATTGTCCAAATGGTTACACTTCTGGTACTCTTGGTTTCTCATCTTCCAATTCCAGATTTCTGATTGATTGTTCCAGTGCTGAAGCTTACATTACAAATCTTTTAAATTCCTGGAACAAGCCAACTACGGCTGATTCTTTTACATATACCCAAACTTGTACTTCCAAAAGTTTTCAAATAACATTCAATAATATTCCAGCTGGTTATCGTCCATATATTGCTGCATTGGTTCAAGCTCCATCTTCGGATTATGCTATACAATATACTGCAAAATATCAATGTACTGGATCTACCCAAAAAGATATTACGAAATCGGTCACTTGGTCAGGCTATACAAATAGTGACACAGATTCAAATGGTGCTGTAGTTGTTCTTACAACTATTACAGGTACTCAGTCCAATACTATTGTTACCACATTGCCTTTCAACCCGACTGTTGATCACACAAAAACGATCGAAGTGATTGTTCCAATTCCAACAGTAACAACTACCACTTCCTACATTGGAGTTACTACTTCTTATACCACAATTACTGGTACTATTGGTGGAACGGCTACTGTTATTGTCGATGAACCGTATCATACTACTACCACTGTTACTAGTCCCTGGACTGGAACCTTCACCACTTCAACTACTGTCATTGCTTCTACTGACTCGGT

**>*CtrALS3786*_1021**

ATGATTTTTTCTGAGTTTTTAATATTGTCGCTAACATTCATTGCTACTTCAGTTGCTAAAGAAATTTCCGGTGTGTTCATAGGATTTGAATCCTTAACTTGGGATAAAGCTGGTGATTATGCTTATCAAGGTCCTCAATATCCAACATGGAATGCCGTGCTTGATTGGTCGTTAGATGGCACAACAACTTCCCCAGGTGATACGTTCACTTTGATCATGCCCTGTGTCTTCAAGTTCACTACGTTTGCCACTTCTGTTGATTTGACTGCCAATGGTATTACATATGCCACATGTGATCTTCATGCCGGTGAAGAGTTTACTACTTACTCTAGTTTGACTTGTACTGTTACTGATTCGTTAAGTTCTGTTCATGAAGCTATGGGTACAGTCACAATTCCTTTGGCATTTAACGTTGGTGGTTCTGGTTCTTCTGTTGATATTGCTGATTCTACTTGTTTCACTGCTGGCACAAACACTGTTACTTTCCAAGATGGTGATACATCAATCAGTACCCAAGCCTATTTTGCTGCAGCTACTGGATCTTCTTCTGATCTCCTTTACTTCCAAAGAGTTGTTCCTTCATTGAACAAGCTTAATGCACTTGTAATTCTCCCAGATTGTCCAAATGGTTACACTTCTGGTACTCTTGGTTTCTCATCTTCCAATTCCAGATTTCTGATTGATTGTTCCAGTGCTGAAGCTTACATTACAAATCTTTTAAATTCCTGGAACAAGCCAACTACGGCTGATTCTTTTACATATACCCAAACTTGTACTTCCAAAAGTTTTCAAATAACATTCAATAATATTCCAGCTGGTTATCGTCCATATATTGCTGCATTGGTTCAAGCTCCATCTTCGGATTATGCTATACAATATACTGCAAAATATCAATGTACTGGATCTACCCAAAAAGATATTACGAAATCGGTCACTTGGTCAGGCTATACAAATAGTGACACAGATTCAAATGGTGCTGTAGTTGTTCTTACAACTATTACAGGTACTCAGTCCAATACTATTGTTACCACATTGCCTTTCAACCCGACTGTTGATCACACAAAAACGATCGAAGTGATTGTTCCAATTCCAACAGTAACAACTACCACTTCCTACATTGGAGTTACTACTTCTTATACCACAATTACTGGTACTATTGGTGGAACGGCTACTGTTATTGTCGATGAACCGTATCATACTACTACCACTGTTACTAGTCCCTGGACTGGAACCTTCACCACTTCAACTACTGTCATTGCTTCTACTGACTCGGT

**>*CtrALS3786*_3242**

ATGATTTTTTCTGAGTTTTTAATATTGTCGCTAACATTCATTGCTACTTCAGTTGCTAAAGAAATTTCCGGTGTGTTCATAGGATTTGAATCCTTAACTTGGGATAAAGCTGGTGATTATGCTTATCAAGGTCCTCAATATCCAACATGGAATGCCGTGCTTGATTGGTCGTTAGATGGCACAACAACTTCCCCAGGTGATACGTTCACTTTGATCATGCCCTGTGTCTTCAAGTTCACTACGTCTGCCACTTCTGTTGATTTGACTGCCAATGGTATTACATATGCCACATGTGATCTTCATGCCGGTGAAGAGTTTACTACTTACTCTAGTTTGACTTGTACTGTTACTGATTCGTTAAGTTCTGTTCATGAAGCTATGGGTACAGTCACAATTCCTTTGGCATTTAACGTTGGTGGTTCTGGTTCTTCTGTTGATATTGCTGATTCTACTTGTTTCACTGCTGGCACAAACACTGTTACTTTCCAAGATGGTGATACATCAATCAGTACCCAAGCCTATTTTGCTGCAGCTACTGGATCTTCTTCTGATCTCCTTTACTTCCAAAGAGTTGTTCCTTCATTGAACAAGCTTAATGCACTTGTAATTCTCCCAGATTGTCCAAATGGTTACACTTCTGGTACTCTTGGTTTCTCATCTTCCAATTCCAGATTTCTGATTGATTGTTCCAGTGCTGAAGCTTACATTACAAATCTTTTAAATTCCTGGAACAAGCCAACTACGGCTGATTCTTTTACATATACCCAAACTTGTACTTCCAAAAGTTTTCAAATAACATTCAATAATATTCCAGCTGGTTATCGTCCATATATTGCTGCATTGGTTCAAGCTCCATCTTCGGATTATGCTATACAATATACTGCAAAATATCAATGTACTGGATCTACCCAAAAAGATATTACGAAATCGGTCACTTGGTCAGGCTATACAAATAGTGACACAGATTCAAATGGTGCTGTAGTTGTTCTTACAACTATTACAGGTACTCAGTCCAATACTATTGTTACCACATTGCCTTTCAACCCGACTGTTGATCACACAAAAACGATCGAAGTGATTGTTCCAATTCCAACAGTAACAACTACCACTTCCTACATTGGAGTTACTACTTCTTATACCACAATTACTGGTACTATTGGTGGAACGGCTACTGTTATTGTCGATGAACCGTATCATACTACTACCACTGTTACTAGTCCCTGGACTGGAACCTTCACCACTTCAACTACTGTCATTGCTTCTACTGACTCGGT

***CtrALS3791* (Amplified with primers Ctr3791NT-F and Ctr3791NT-R)**

**>*CtrALS3791*_MYA-3404**

ATGTTTCTACTACAAACAACTCTCTTATGCTGTGCATTGATTGCTACTTCAGTTGCTAAAGAAATTTCCGGTGTGTTCATAGGATTTGAATCCTTAACTTGGGATAAAGCTGGTAATTATGCTTATCAAGGTCCTCAATATCCAACATGGAATGCCGTGCTTGATTGGTCGTTAGATGGCACAACAACTTCCCCAGGTGATACGTTCACTTTGATCATGCCCTGTGTCTTCAAGTTCACTACGTCTGCCACTTCTGTTGATTTGACTGCCAATGGTATTACATATGCCACATGTGATCTTCATGCCGGTGAAGAGTTTACTACTTACTCTAGTTTGACTTGTACTGTTACTGATTCGTTAAGTTCTGTTCATGAAGCTATGGGTACAGTCACAATTCCTTTGGCATTTAACGTTGGTGGTTCTGGTTCTTCTGTTGATATTGCTGATTCTACTTGTTTCACTGCTGGCACAAACACTGTTACTTTCCAAGATGGTGATACATCAATCAGTACCCAAGCCTATTTTGCTGCAGCTACTGGATCTTCTTCTGGTCTCCTTTACTTTCAAAGAAGTGTTCCTTCATTGAACAAGCTTAATGCACTTGCAATTCTCCCAGATTGTCCAAATGGTTACACTTCTGGTACTCTTGGTTTCTCATCTTCTAATTCCAGATTTCTGATTGATTGTTCCAGTGCTGAAGCTTACATTACAAATCTTTTAAATTCCTGGAACTACCCAACTTCAGCGGATTCCTTTTCTTATACCCAAACTTGTACTTCCAAAAGTTTTCAAATAACATTCAATAATATTCCAGCCGGCTATCGTCCATATATTGCTGCATTGGTTCAAGCTCCATCTTCGGATTATAAAATTGATTATACTGCAAAATATCAATGTGCTGGATCTTCCCAAAAAGATGCTTCGAAATCGGTCACTTGGTCAGGCTATACAAATAGTGACACAGATTCAAATGGTGCTGTAGTTGTTCTTACAACTATTACAGGTACTCAGTCCAATACTATTGTTACCACATTGCCTTTCAACCCGACTGTTGATCACACAAAAACGATCGAAGTGATTGTTCCAATTCCAACAGTAACAACTACCACTTCCTACATTGGAGTTACTACTTCTTATACCACAATTACTGGTACTATTGGTGGAACGGCTACTGTTATTGTCGATGAACCGTATCATACTACTACYACTGTTACTAGTCCCTGGACTGGAACCTTCACCACTTCAACTACTGTCATTGCTTCTACTGACTCGGTTG

**>*CtrALS3791*_951**

ATGTTTCTACTACAAACAACTCTCTTATGCTGTGCATTGATTGCTACTTCAGTTGCTAAAGAAATTTCCGGTGTGTTCATAGGATTTGAATCCTTAACTTGGGATAAAGCTGGTAATTATGCTTATCAAGGTCCTCAATATCCAACATGGAATGCCGTGCTTGATTGGTCGTTAGATGGCACAACAACTTCCCCAGGTGATACGTTCACTTTGATCATGCCCTGTGTCTTCAAGTTCACTACGTCTGCCACTTCTGTTGATTTGACTGCCAATGGTATTACATATGCCACATGTGATCTTCATGCCGGTGAAGAGTTTACTACTTACTCTAGTTTGACTTGTACTGTTACTGATTCGTTAAGTTCTGTTCATGAAGCTATGGGTACAGTCACAATTCCTTTGGCATTTAACGTTGGTGGTTCTGGTTCTTCTGTTGATATTGCTGATTCTACTTGTTTCACTGCTGGCACAAACACTGTTACTTTCCAAGATGGTGATACATCAATCAGTACCCAAGCCTATTTTGCTGCAGCTACTGGATCTTCTTCTGGTCTCCTTTACTTTCAAAGAAGTGTTCCTTCATTGAACAAGCTTAATGCACTTGCAATTCTCCCAGATTGTCCAAATGGTTACACTTCTGGTACTCTTGGTTTCTCATCTTCTAATTCCAGATTTCTGATTGATTGTTCCAGTGCTGAAGCTTACATTACAAATCTTTTAAATTCCTGGAACTACCCAACTTCAGCGGATTCCTTTTCTTATACCCAAACTTGTACTTCCAAAAGTTTTCAAATAACATTCAATAATATTCCAGCTGGTTATCGTCCATATATTGCTGCATTGGTTCAAGCTCCATCTTCGGATTATAAAATTGATTATACTGCAAAATATCAATGTGCTGGATCTTCCCAAAAAGATGCTTCGAAATCGGTCACTTGGTCAGGCTATACAAATAGTGACACAGATTCAAATGGTGCTGTAGTTGTTCTTACAACTATTACAGGTACTCAGTCCAATACTATTGTTACCACATTGCCTTTCAACCCGACTGTTGATCACACAAAAACGATCGAAGTGATTGTTCCAATTCCAACAGTAACAACTACCACTTCCTACATTGGAGTTACTACTTCTTATACCACAATTACTGGTACTATTGGTGGAACGGCTACTGTTATTGTCGATGAACCGTATCATACTACTACCACTGTTACTAGTCCCTGGACTGGAACCTTCACCACTTCAACTACTGTCATTGCTTCTACTGACTCGGTTG

**>*CtrALS3791*_952**

ATGTTTCTACTACAAACAACTCTCTTATGCTGTGCATTGATTGCTACTTCAGTTGCTAAAGAAATTTCCGGTGTGTTCATAGGATTTGAATCCTTAACTTGGGATAAAGCTGGTAATTATGCTTATCAAGGTCCTCAATATCCAACATGGAATGCCGTGCTTGATTGGTCGTTAGATGGCACAACAACTTCCCCAGGTGATACGTTCACTTTGATCATGCCCTGTGTCTTCAAGTTCACTACGTCTGCCACTTCTGTTGATTTGACTGCCAATGGTATTACATATGCCACATGTGATCTTCATGCCGGTGAAGAGTTTACTACTTACTCTAGTTTGACTTGTACTGTTACTGATTCGTTAAGTTCTGTTCATGAAGCTATGGGTACAGTCACAATTCCTTTGGCATTTAACGTTGGTGGTTCTGGTTCTTCTGTTGATATTGCTGATTCTACTTGTTTCACTGCTGGCACAAACACTGTTACTTTCCAAGATGGTGATACATCAATCAGTACCCAAGCCTATTTTGCTGCAGCTACTGGATCTTCTTCTGGTCTCCTTTACTTTCAAAGAAGTGTTCCTTCATTGAACAAGCTTAATGCACTTGCAATTCTCCCAGATTGTCCAAATGGTTACACTTCTGGTACTCTTGGTTTCTCATCTTCTAATTCCAGATTTCTGATTGATTGTTCCAGTGCTGAAGCTTACATTACAAATCTTTTAAATTCCTGGAACTACCCAACTTCAGCGGATTCCTTTTCTTATACCCAAACTTGTACTTCCAAAAGTTTTCAAATAACATTCAATAATATTCCAGCCGGCTATCGTCCATATATTGCTGCATTGGTTCAAGCTCCATCTTCGGATTATAAAATTGATTATACTGCAAAATATCAATGTGCTGGATCTTCCCAAAAAGATGCTTCAAAATCGGTCACTTGGTCAGGCTATACAAATAGTGACACAGATTCAAATGGTGCTGTAGTTGTTCTTACAACTATTACAGGTACTCAGTCCAATACTATTGTTACCACATTGCCTTTCAACCCGACTGTTGATCACACAAAAACGATCGAAGTGATTGTTCCAATTCCAACAGTAACAACTACCACTTCCTACATTGGAGTTACTACTTCTTATACCACAATTACTGGTACTATTGGTGGAACGGCTACTGTTATTGTCGATGAACCGTATCATACTACTACCACTGTTACTAGTCCCTGGACTGGAACCTTCACCACTTCAACTACTGTCATTGCTTCTACTGACTCGGTTG

**>*CtrALS3791*_1019**

ATGTTTCTACTACAAACAACTCTCTTATGCTGTGCATTGATTGCTACTTCAGTTGCTAAAGAAATTTCCGGTGTGTTCATAGGATTTGAATCCTTAACTTGGGATAAAGCTGGTAATTATGCTTATCAAGGTCCTCAATATCCAACATGGAATGCCGTGCTTGATTGGTCGTTAGATGGCACAACAACTTCCCCAGGTGATACGTTCACTTTGATCATGCCCTGTGTCTTCAAGTTCACTACGTCTGCCACTTCTGTTGATTTGACTGCCAATGGTATTACATATGCCACATGTGATCTTCATGCCGGTGAAGAGTTTACTACTTACTCTAGTTTGACTTGTACTGTTACTGATTCGTTAAGTTCTGTTCATGAAGCTATGGGTACAGTCACAATTCCTTTGGCATTTAACGTTGGTGGTTCTGGTTCTTCTGTTGATATTGCTGATTCTACTTGTTTCACTGCTGGCACAAACACTGTTACTTTCCAAGATGGTGATACATCAATCAGTACCCAAGCCTATTTTGCTGCAGCTACTGGATCTTCTTCTGGTCTCCTTTACTTTCAAAGAAGTGTTCCTTCATTGAACAAGCTTAATGCACTTGCAATTCTCCCAGATTGTCCAAATGGTTACACTTCTGGTACTCTTGGTTTCTCATCTTCTAATTCCAGATTTCTGATTGATTGTTCCAGTGCTGAAGCTTACATTACAAATCTTTTAAATTCCTGGAACTACCCAACTTCAGCGGATTCCTTTTCTTATACCCAAACTTGTACTTCCAAAAGTTTTCAAATAACATTCAATAATATTCCAGCYGGYTATCGTCCATATATTGCTGCATTGGTTCAAGCTCCATCTTCGGATTATAAAATTGATTATACTGCAAAATATCAATGTGCTGGATCTTCCCAAAAAGATGCTTCRAAATCGGTCACTTGGTCAGGCTATACAAATAGTGACACAGATTCAAATGGTGCTGTAGTTGTTCTTACAACTATTACAGGTACTCAGTCCAATACTATTGTTACCACATTGCCTTTCAACCCGACTGTTGATCACACAAAAACGATCGAAGTGATTGTTCCAATTCCAACAGTAACAACTACCACTTCCTACATTGGAGTTACTACTTCTTATACCACAATTACTGGTACTATTGGTGGAACGGCTACTGTTATTGTCGATGAACCGTATCATACTACTACCACTGTTACTAGTCCCTGGACTGGAACCTTCACCACTTCAACTACTGTCATTGCTTCTACTGACTCGGTTG

**>*CtrALS3791*_1020**

ATGTTTCTACTACAAACAACTCTCTTATGCTGTGCATTGATTGCTACTTCAGTTGCTAAAGAAATTTCCGGTGTGTTCATAGGATTTGAATCCTTAACTTGGGATAAAGCTGGTAATTATGCTTATCAAGGTCCTCAATATCCAACATGGAATGCCGTGCTTGATTGGTCGTTAGATGGCACAACAACTTCCCCAGGTGATACGTTCACTTTGATCATGCCCTGTGTCTTCAAGTTCACTACGTCTGCCACTTCTGTTGATTTGACTGCCAATGGTATTACATATGCCACATGTGATCTTCATGCCGGTGAAGAGTTTACTACTTACTCTAGTTTGACTTGTACTGTTACTGATTCGTTAAGTTCTGTTCATGAAGCTATGGGTACAGTCACAATTCCTTTGGCATTTAACGTTGGTGGTTCTGGTTCTTCTGTTGATATTGCTGATTCTACTTGTTTCACTGCTGGCACAAACACTGTTACTTTCCAAGATGGTGATACATCAATCAGTACCCAAGCCTATTTTGCTGCAGCTACTGGATCTTCTTCTGGTCTCCTTTACTTTCAAAGAAGTGTTCCTTCATTGAACAAGCTTAATGCACTTGCAATTCTCCCAGATTGTCCAAATGGTTACACTTCTGGTACTCTTGGTTTCTCATCTTCTAATTCCAGATTTCTGATTGATTGTTCCAGTGCTGAAGCTTACATTACAAATCTTTTAAATTCCTGGAACTACCCAACTTCAGCGGATTCCTTTTCTTATACCCAAACTTGTACTTCCAAAAGTTTTCAAATAACATTCAATAATATTCCAGCCGGCTATCGTCCATATATTGCTGCATTGGTTCAAGCTCCATCTTCGGATTATAAAATTGATTATACTGCAAAATATCAATGTGCTGGATCTTCCCAAAAAGATGCTTCAAAATCGGTCACTTGGTCAGGCTATACAAATAGTGACACAGATTCAAATGGTGCTGTAGTTGTTCTTACAACTATTACAGGTACTCAGTCCAATACTATTGTTACCACATTGCCTTTCAACCCGACTGTTGATCACACAAAAACGATCGAAGTGATTGTTCCAATTCCAACAGTAACAACTACCACTTCCTACATTGGAGTTACTACTTCTTATACCACAATTACTGGTACTATTGGTGGAACGGCTACTGTTATTGTCGATGAACCGTATCATACTACTACCACTGTTACTAGTCCCTGGACTGGAACCTTCACCACTTCAACTACTGTCATTGCTTCTACTGACTCGGTTG

**>*CtrALS3791*_1021**

ATGTTTCTACTACAAACAACTCTCTTATGCTGTGCATTGATTGCTACTTCAGTTGCTAAAGAAATTTCCGGTGTGTTCATAGGATTTGAATCCTTAACTTGGGATAAAGCTGGTAATTATGCTTATCAAGGTCCTCAATATCCAACATGGAATGCCGTGCTTGATTGGTCGTTAGATGGCACAACAACTTCCCCAGGTGATACGTTCACTTTGATCATGCCCTGTGTCTTCAAGTTCACTACGTCTGCCACTTCTGTTGATTTGACTGCCAATGGTATTACATATGCCACATGTGATCTTCATGCCGGTGAAGAGTTTACTACTTACTCTAGTTTGACTTGTACTGTTACTGATTCGTTAAGTTCTGTTCATGAAGCTATGGGTACAGTCACAATTCCTTTGGCATTTAACGTTGGTGGTTCTGGTTCTTCTGTTGATATTGCTGATTCTACTTGTTTCACTGCTGGCACAAACACTGTTACTTTCCAAGATGGTGATACATCAATCAGTACCCAAGCCTATTTTGCTGCAGCTACTGGATCTTCTTCTGGTCTCCTTTACTTTCAAAGAAGTGTTCCTTCATTGAACAAGCTTAATGCACTTGCAATTCTCCCAGATTGTCCAAATGGTTACACTTCTGGTACTCTTGGTTTCTCATCTTCTAATTCCAGATTTCTGATTGATTGTTCCAGTGCTGAAGCTTACATTACAAATCTTTTAAATTCCTGGAACTACCCAACTTCAGCGGATTCCTTTTCTTATACCCAAACTTGTACTTCCAAAAGTTTTCAAATAACATTCAATAATATTCCAGCCGGCTATCGTCCATATATTGCTGCATTGGTTCAAGCTCCATCTTCGGATTATAAAATTGATTATACTGCAAAATATCAATGTGCTGGATCTTCCCAAAAAGATGCTTCGAAATCGGTCACTTGGTCAGGCTATACAAATAGTGACACAGATTCAAATGGTGCTGTAGTTGTTCTTACAACTATTACAGGTACTCAGTCCAATACTATTGTTACCACATTGCCTTTCAACCCGACTGTTGATCACACAAAAACGATCGAAGTGATTGTTCCAATTCCAACAGTAACAACTACCACTTCCTACATTGGAGTTACTACTTCTTATACCACAATTACTGGTACTATTGGTGGAACGGCTACTGTTATTGTCGATGAACCGTATCATACTACTACCACTGTTACTAGTCCCTGGACTGGAACCTTCACCACTTCAACTACTGTCATTGCTTCTACTGACTCGGTTG

**>*CtrALS3791*_3242**

ATGTTTCTACTACAAACAACTCTCTTATGCTGTGCATTGATTGCTACTTCAGTTGCTAAAGAAATTTCCGGTGTGTTCATAGGATTTGAATCCTTAACTTGGGATAAAGCTGGTAATTATGCTTATCAAGGTCCTCAATATCCAACATGGAATGCCGTGCTTGATTGGTCGTTAGATGGCACAACAACTTCCCCAGGTGATACGTTCACTTTGATCATGCCCTGTGTCTTCAAGTTCACTACGTCTGCCACTTCTGTTGATTTGACTGCCAATGGTATTACATATGCCACATGTGATCTTCATGCCGGTGAAGAGTTTACTACTTACTCTAGTTTGACTTGTACTGTTACTGATTCGTTAAGTTCTGTTCATGAAGCTATGGGTACAGTCACAATTCCTTTGGCATTTAACGTTGGTGGTTCTGGTTCTTCTGTTGATATTGCTGATTCTACTTGTTTCACTGCTGGCACAAACACTGTTACTTTCCAAGATGGTGATACATCAATCAGTACCCAAGCCTATTTTGCTGCAGCTACTGGATCTTCTTCTGGTCTCCTTTACTTTCAAAGAAGTGTTCCTTCATTGAACAAGCTTAATGCACTTGCAATTCTCCCAGATTGTCCAAATGGTTACACTTCTGGTACTCTTGGTTTCTCATCTTCTAATTCCAGATTTCTGATTGATTGTTCCAGTGCTGAAGCTTACATTACAAATCTTTTAAATTCCTGGAACTACCCAACTTCAGCGGATTCCTTTTCTTATACCCAAACTTGTACTTCCAAAAGTTTTCAAATAACATTCAATAATATTCCAGCCGGCTATCGTCCATATATTGCTGCATTGGTTCAAGCTCCATCTTCGGATTATAAAATTGATTATACTGCAAAATATCAATGTGCTGGATCTTCCCAAAAAGATGCTTCGAAATCGGTCACTTGGTCAGGCTATACAAATAGTGACACAGATTCAAATGGTGCTGTAGTTGTTCTTACAACTATTACAGGTACTCAGTCCAATACTATTGTTACCACATTGCCTTTCAACCCGACTGTTGATCACACAAAAACGATCGAAGTGATTGTTCCAATTCCAACAGTAACAACTACCACTTCCTACATTGGAGTTACTACTTCTTATACCACAATTACTGGTACTATTGGTGGAACGGCTACTGTTATTGTCGATGAACCGTATCATACTACTACCACTGTTACTAGTCCCTGGACTGGAACCTTCACCACTTCAACTACTGTCATTGCTTCTACTGACTCGGTTG

***CtrALS3797* (Amplified with primers Ctr3797NT-F and Ctr3797NTSq-R2)**

**>*CtrALS3797*_MYA-3404**

ATGAATTTCGTACTGTTATTGTTTACATTGCTCCTTTTAGTCACTCGAGCAACGTCAAAAACACTTACTGGAGTTTTCCAAAGTTTTAATTCGTTGACTTGGGAGAAAGCAGCTCTGTATAAATACAGAGGACCGCAATTTCCGACATGGAACGCTGCTGTTAATTGGGCATTGGATTCCAATGCTAATGCCGGTGATACATTTACCTTAATTATGCCATGTGTTTTTAAATTTACTACTAGTGAAACTTCTATTGATTTAACTGTGGGTAGTAAATCCTATGCTACTTGTAATTTCAATGCTGGGGAACATTTTACCACTTTTTCTAGTTTGAGTTGTACTGTGACACAGAGTGTTCCTGATAATACCAATGCATATGGTACAATCACTGTTCCACTTGCCTTTAATGTTGGGGGTTCTGGTCGTGATGTCGATACTACTGATGCAAAGTGTTTTACTACAGGTGACAATACTGTTACATTTAGTGATGGTGATAAATCATTCTCAACTACAGCAAATTTTGAAGGTGCTGGTACTTTGAATGATGATTATGAATCTTCAAGACTCATTCCTTCACTTGGTAAAACTGATGCTTTGTTGGTTGCACCATTGTGTTCCAATGGGTATAAATCAGGTACTATTGGGTTTTCTTCGAAAGCAAGCGGTTATTCAATTGATTGTAACAATATTCAAGCTGGTATTACTAGTCAATTGAATGCATGGGGTTTTCCAACGGACCTGCAAAGCTTTTCATACACCACTCAATGTACTACCACTAGTTATTCCATAACTTTTAGTACTATTCCAAAAGGTTTACGTCCATTCATTGATGCCTATATTAAAACACCTACTTCCACATATGCGGTGACATACACTTTCAAATATGTTTGTGCCGATGGAAAATCATATAATAGTAATCGAAGTTTGAATTGGTCCGGATATGTTAACGGTGATGCAGATTCTGAAGGTATGGAAATTGTTGTTGCTACTACTACTGGTACTGGTTCTACTACTGGTGTTACAACATTACCATTTGATAAAACCAAAGACAAAACCAAAACAATTCAAGTTATTGAACCAATTCCAACTACAACAGTTACTACTTCATACCTCGGTGTCACAACCTCTTTTTCCACCATTACTGCTACTATCGGTGGAACTGCTACTGTCATTGTTGATGAACCATACCATTCTACCACTACGG

**>*CtrALS3797*_951**

ATGAATTTCGTACTGTTATTGTTTACATTGCTCCTTTTAGTCACTCGAGCAACGTCAAAAACACTTACTGGAGTTTTCCAAAGTTTTGATTCGTTGACTTGGGACAAAGCAAATCTGTATAAATACAGAGGACCACAATATCCGACATGGAACGCTGCTGTTAATTGGGCATTGGATTCCAATGCTGATGCCGGTGACACATTTACCTTAATTATGCCATGTGTTTTTAAATTTACTACTAGTGAAACTTCTATTGATTTAACTGTGGGTAGTAAATCCTATGCTACTTGTAATTTCAATGCTGGGGAACATTTTACCACTTTTTCTAGTTTGAGTTGTACTGTGACACAGAGTGTTCCTGATAATACCAATGCATATGGTACAATCACTGTTCCACTTGCCTTTAATGTTGGGGGCTCTGGTCGTGATGTCGATCTTACTGATGCAAAGTGTTTTACTACAGGTGATAATACTGTTACATTTAGTGATGGTGATAAATCATTCTCCACTACAGCAAATTTCGAAGGTGCCGGTACTTTGAATGACGATTATGAATCTTCAAGACTCATTCCCTCACTTGGTAAAACTGATGCTTTGTTGGTTGCACCATTGTGTTCTAATGGGTATAAATCAGGTACTATTGGGTTTTCCTCAACAACAAAGGGTTTTTCAATTGATTGTAACAATATTCAAGCTGGTATTACTAGTCAATTGAATGCATGGGGTTTTCCAACGGACCTGCAAAGCTTTTCATACACCACTCAATGTACTACCACTAGTTATTCCATAACTTTTAGTACTATTCCAAAAGGTTTACGTCCATTCATTGATGCTTATATTAAAGCACCTACTTCCACATACCCCATGACATACACTTACAAATATGTTTGTTCCGATGGAAAATCATATAATGGCAATACAAAATTGAATTGGTCGGGATATGTTAACAGTGATGCAGATTCTGAAGGTATGGAAATTGTTGTTGCTACTACTACTGGTACTGGTTCTACTACTGGTGTTACAACATTACCATTTGATAAAACCAAAGACAAAACCAAAACAATTCAAGTTATTGAACCAATTCCAACTACAACAGTTACTACTTCATACCTCGGTGTCACAACCTCTTTTTCGACCATTACTGCTACTATCGGTGGAACTGCTACTGTCATTGTTGATGAACCATACCATTCTACCACTACGG

**>*CtrALS3797*_952**

ATGAATTTCGTACTGTTATTGTTTACATTGCTCCTTTTAGTCACTCGAGCAACGTCAAAAACACTTACTGGAGTTTTCCAAAGTTTTAATTCGTTGACTTGGGAGAAAGCAGCTCTGTATAAATACAGAGGACCGCAATTTCCGACATGGAACGCTGCTGTTAATTGGGCATTGGATTCCAATGCTAATGCCGGTGATACATTTACCTTAATTATGCCATGTGTTTTTAAATTTACTACTAGTGAAACTTCTATTGATTTAACTGTGGGTAGTAAATCCTATGCTACTTGTAATTTCAATGCTGGGGAACATTTTACCACTTTTTCTAGTTTGAGTTGTACTGTGACACAGAGTGTTCCTGATAATACCAATGCATATGGTACAATCACTGTTCCACTTGCCTTTAATGTTGGGGGTTCTGGTCGTGATGTCGATACTACTGATGCAAAGTGTTTTACTACAGGTGACAATACTGTTACATTTAGTGATGGTGATAAATCATTCTCAACTACAGCAAATTTTGAAGGTGCTGGTACTTTGAATGATGATTATGAATCTTCAAGACTCATTCCTTCACTTGGTAAAACTGATGCTTTGTTGGTTGCACCATTGTGTTCCAATGGGTATAAATCAGGTACTATTGGGTTTTCTTCGAAAGCAAGCGGTTATTCAATTGATTGTAACAATATTCAAGCTGGTATTACTAGTCAATTGAATGCATGGGGTTTTCCAACGGACCTGCAAAGCTTTTCATACACCACTAAATGTACTACCACTAGTTATTCCATAACTTTTAGTACTATTCCAAAAGGTTTACGTCCATTCATTGATGCCTATATTAAAACACCTACTTCCACATATGCGGTGACATACACTTTCAAATATGTTTGTGCCGATGGAAAATCATATAATAGTAATCGAAGTTTGAATTGGTCCGGATATGTTAACGGTGATGCAGATTCTGAAGGTATGGAAATTGTTGTTGCTACTACTACTGGTACTGGTTCTACTACTGGTGTTACAACATTACCATTTGATAAAACCAAAGACAAAACCAAAACAATTCAAGTTATTGAACCAATTCCAACTACAACAGTTACTACTTCATACCTCGGTGTCACAACCTCTTTTTCCACCATTACTGCTACTATCGGTGGAACTGCTACTGTCATTGTTGATGAACCATACCATTCTACCACTACGG

**>*CtrALS3797*_1019**

ATGAATTTCGTACTGTTATTGTTTACATTGCTCCTTTTAGTCACTCGAGCAACGTCAAAAACACTTACTGGAGTTTTCCAAAGTTTTRATTCGTTGACTTGGGASAAAGCARMTCTGTATAAATACAGAGGACCRCAATWTCCGACATGGAACGCTGCTGTTAATTGGGCATTGGATTCCAATGCTRATGCCGGTGAYACATTTACCTTAATTATGCCATGTGTTTTTAAATTTACTACTAGTGAAACTTCTATTGATTTAACTGTGGGTAGTAAATCCTATGCTACTTGTAATTTCAATGCTGGGGAACATTTTACCACTTTTTCTAGTTTGAGTTGTACTGTGACACAGAGTGTTCCTGATAATACCAATGCATATGGTACAATCACTGTTCCACTTGCCTTTAATGTTGGGGGYTCTGGTCGTGATGTCGATMYTACTGATGCAAAGTGTTTTACTACAGGTGAYAATACTGTTACATTTAGTGATGGTGATAAATCATTCTCMACTACAGCAAATTTYGAAGGTGCYGGTACTTTGAATGAYGATTATGAATCTTCAAGACTCATTCCYTCACTTGGTAAAACTGATGCTTTGTTGGTTGCACCATTGTGTTCCAATGGGTATAAATCAGGTACTATTGGGTTTTCTTCGAMAGCAAGSGGTTATTCAATTGATTGTAACAATATTCAAGCTGGTATTACTAGTCAATTGAATGCATGGGGTTTTCCAACGGACCTGCAAAGCTTTTCATACACCACTMAATGTACTACCACTAGTTATTCCATAACTTTTAGTACTATTCCAAAAGGTTTACGTCCATTCATTGATGCYTATATTAAARCACCTACTTCCACATAYSCSRTGACATACACTTWCAAATATGTTTGTKCCGATGGAAAATCATATAATGGYAATMSAARWTTGAATTGGTCGGGATATGTTAACRGTGATGCAGATTCTGAAGGTATGGAAATTGTTGTTGCTACTACTACTGGTACTGGTTCTACTACTGGTGTTACAACATTACCATTTGATAAAACCAAAGACAAAACCAAAACAATTCAAGTTATTGAACCAATTCCAACTACAACAGTTACTACTTCATACCTCGGTGTCACAACCTCTTTTTCSACCATTACTGCTACTATCGGTGGAACTGCTACTGTCATTGTTGATGAACCATACCATTCTACCACTACGG

**>*CtrALS3797*_1020**

ATGAATTTCGTACTGTTATTGTTTACATTGCTCCTTTTAGTCACTCGAGCAACGTCAAAAACACTTACTGGAGTTTTCCAAAGTTTTGATTCGTTGACTTGGGACAAAGCAAATCTGTATAAATACAGAGGACCACAATATCCGACATGGAACGCTGCTGTTAATTGGGCATTGGATTCCAATGCTGATGCCGGTGACACATTTACCTTAATTATGCCATGTGTTTTTAAATTTACTACTAGTGAAACTTCTATTGATTTAACTGTGGGTAGTAAATCCTATGCTACTTGTAATTTCAATGCTGGGGAACATTTTACCACTTTTTCTAGTTTGAGTTGTACTGTGACACAGAGTGTTCCTGATAATACCAATGCATATGGTACAATCACTGTTCCACTTGCCTTTAATGTTGGGGGCTCTGGTCGTGATGTCGATCTTACTGATGCAAAGTGTTTTACTACAGGTGATAATACTGTTACATTTAGTGATGGTGATAAATCATTCTCCACTACAGCAAATTTCGAAGGTGCCGGTACTTTGAATGACGATTATGAATCTTCAAGACTCATTCCCTCACTTGGTAAAACTGATGCTTTGTTGGTTGCACCATTGTGTTCTAATGGGTATAAATCAGGTACTATTGGGTTTTCCTCAACAACAAAGGGTTTTTCAATTGATTGTAACAATATTCAAGCTGGTATTACTAGTCAATTGAATGCATGGGGTTTTCCAACGGACCTGCAAAGCTTTTCATACACCACTCAATGTACTACCACTAGTTATTCCATAACTTTTAGTACTATTCCAAAAGGTTTACGTCCATTCATTGATGCTTATATTAAAGCACCTACTTCCACATACCCCATGACATACACTTACAAATATGTTTGTTCCGATGGAAAATCATATAATGGCAATACAAAATTGAATTGGTCGGGATATGTTAACAGTGATGCAGATTCTGAAGGTATGGAAATTGTTGTTGCTACTACTACTGGTACTGGTTCTACTACTGGTGTTACAACATTACCATTTGATAAAACCAAAGACAAAACCAAAACAATTCAAGTTATTGAACCAATTCCAACTACAACAGTTACTACTTCATACCTCGGTGTCACAACCTCTTTTTCGACCATTACTGCTACTATCGGTGGAACTGCTACTGTCATTGTTGATGAACCATACCATTCTACCACTACGG

**>*CtrALS3797*_1021**

ATGAATTTCGTACTGTTATTGTTTACATTGCTCCTTTTAGTCACTCGAGCAACGTCAAAAACACTTACTGGAGTTTTCCAAAGTTTTAATTCGTTGACTTGGGAGAAAGCAGCTCTGTATAAATACAGAGGACCGCAATTTCCGACATGGAACGCTGCTGTTAATTGGGCATTGGATTCCAATGCTAATGCCGGTGATACATTTACCTTAATTATGCCATGTGTTTTTAAATTTACTACTAGTGAAACTTCTATTGATTTAACTGTGGGTAGTAAATCCTATGCTACTTGTAATTTCAATGCTGGGGAACATTTTACCACTTTTTCTAGTTTGAGTTGTACTGTGACACAGAGTGTTCCTGATAATACCAATGCATATGGTACAATCACTGTTCCACTTGCCTTTAATGTTGGGGGTTCTGGTCGTGATGTCGATACTACTGATGCAAAGTGTTTTACTACAGGTGACAATACTGTTACATTTAGTGATGGTGATAAATCATTCTCAACTACAGCAAATTTTGAAGGTGCTGGTACTTTGAATGATGATTATGAATCTTCAAGACTCATTCCTTCACTTGGTAAAACTGATGCTTTGTTGGTTGCACCATTGTGTTCCAATGGGTATAAATCAGGTACTATTGGGTTTTCTTCGAAAGCAAGCGGTTATTCAATTGATTGTAACAATATTCAAGCTGGTATTACTAGTCAATTGAATGCATGGGGTTTTCCAACGGACCTGCAAAGCTTTTCATACACCACTAAATGTACTACCACTAGTTATTCCATAACTTTTAGTACTATTCCAAAAGGTTTACGTCCATTCATTGATGCCTATATTAAAACACCTACTTCCACATATGCGGTGACATACACTTTCAAATATGTTTGTGCCGATGGAAAATCATATAATAGTAATCGAAGTTTGAATTGGTCCGGATATGTTAACGGTGATGCAGATTCTGAAGGTATGGAAATTGTTGTTGCTACTACTACTGGTACTGGTTCTACTACTGGTGTTACAACATTACCATTTGATAAAACCAAAGACAAAACCAAAACAATTCAAGTTATTGAACCAATTCCAACTACAACAGTTACTACTTCATACCTCGGTGTCACAACCTCTTTTTCCACCATTACTGCTACTATCGGTGGAACTGCTACTGTCATTGTTGATGAACCATACCATTCTACCACTACGG

**>*CtrALS3797*_3242**

ATGAATTTCGTACTGTTATTGTTTACATTGCTCCTTTTAGTCACTCGAGCAACGTCAAAAACACTTACTGGAGTTTTCCAAAGTTTTRATTCGTTGACTTGGGASAAAGCARMTCTGTATAAATACAGAGGACCRCAATWTCCGACATGGAACGCTGCTGTTAATTGGGCATTGGATTCCAATGCTRATGCCGGTGAYACATTTACCTTAATTATGCCATGTGTTTTTAAATTTACTACTAGTGAAACTTCTATTGATTTAACTGTGGGTAGTAAATCCTATGCTACTTGTAATTTCAATGCTGGGGAACATTTTACCACTTTTTCTAGTTTGAGTTGTACTGTGACACAGAGTGTTCCTGATAATACCAATGCATATGGTACAATCACTGTTCCACTTGCCTTTAATGTTGGGGGYTCTGGTCGTGATGTCGATMYTACTGATGCAAAGTGTTTTACTACAGGTGAYAATACTGTTACATTTAGTGATGGTGATAAATCATTCTCMACTACAGCAAATTTYGAAGGTGCYGGTACTTTGAATGAYGATTATGAATCTTCAAGACTCATTCCYTCACTTGGTAAAACTGATGCTTTGTTGGTTGCACCATTGTGTTCCAATGGGTATAAATCAGGTACTATTGGGTTTTCTTCGAMAGCAAGSGGTTATTCAATTGATTGTAACAATATTCAAGCTGGTATTACTAGTCAATTGAATGCATGGGGTTTTCCAACGGACCTGCAAAGCTTTTCATACACCACTAAATGTACTACCACTAGTTATTCCATAACTTTTAGTACTATTCCAAAAGGTTTACGTCCATTCATTGATGCYTATATTAAARCACCTACTTCCACATAYSCSGTGACATACACTTWCAAATATGTTTGTKCCGATGGAAAATCATATAATGGYAATMSAARWTTGAATTGGTCGGGATATGTTAACRGTGATGCAGATTCTGAAGGTATGGAAATTGTTGTTGCTACTACTACTGGTACTGGTTCTACTACTGGTGTTACAACATTACCATTTGATAAAACCAAAGACAAAACCAAAACAATTCAAGTTATTGAACCAATTCCAACTACAACAGTTACTACTTCATACCTCGGTGTCACAACCTCTTTTTCSACCATTACTGCTACTATCGGTGGAACTGCTACTGTCATTGTTGATGAACCATACCATTCTACCACTACGG

***CtrALS3871* (Amplified with primers Ctr3871NT-F and Ctr3871NT-R)**

**>*CtrALS3871*_MYA-3404**

ATGAACCTAATTACATTTATTTTGTTATTGTCGTCCCTCATCACAATAGTAACACCCAAAGAAATCACTGGAGTATTTACAAGTTTTAATTCCTTAACTTATAATGATGCAGCTAATTATGGTGCTCAATGCCCGGGATATCCAACATGGATTGCAACTTTAGGTTGGTCTTTAGATGGTTCTGTTGCCTCTCCTGGTGACTCATTCACCTTGATTATGCCTTGTGTTTTCAAGTTTACTAGTTCAGAAACATCRGTGGACTTAACTGTTGATGGAGTAAGTTATGCTACTTGTAATTTGAATAACGGGGAAGAATTTACTACATTTTCTAGTATGTCTTGTGTTGTTAGTAGTGCTTTAACTTCTACTACTCAAGCTTTAGGTGCTGTTAGTATTCCATTCTCATTTAATGTTGGTGGTTCTGGTTCGTCCGTTGATTTAGAAGATGCCACTTGTTTTACTGCTGGTACCAATACTGTTACTTTCAAAGATGGTGATAACGAACTCTCGATCAATGCCGTTTTTGACAAAACCACTGCTTCAGTATCAGATGAAATTATTTCAGTTAGATCTGTTCCGTCGATTGGAAAATTGCAACAAATMTCTATTGCAAAAGATTGTCCAAGTGGCTATGGTAGTGGATATATGAGTATAATTATTAAAGAYAATACTGCTGTTATGGATTGCTCTTCAGTTCATATTGGTATTACTAATGAACTCAATGATTGGAATCAACCAATGAATTCTGAATCATTTTCTTACACTAAAAGCTGTTCAGCAACTGAGTTTATTGTTTCATTCACTGATATTGCAGCTGGTTATAGACCATTCATGGACTCATTCCTTACAACAACTGGCAATGCAAAATTAACTGTTGATTATCATTACGAGTATACTTGTAAAAATGGTGATACGGTTGCTGAAACTGACAGAAGAATTTTCAGTCCTTATACGAACAGTAATACTGGCTGTAGCGGTGTTGTTTTGGTTATAACCACCAGAACAGGAACACAAACTACTACTGCTGTTACCACTTTGCCATTCGATCCAAGTCAAGACCATACTAAAACAATTGAAGTTATTGAACCAATTCCAACAACAACAATCACTACTTCATACCTTGGTATCACAACTTCTTATAGTACAATCTCAGGCACTGTTSGTGGAACTGCTACTGTTATTGTTGATGAACCATATCATTCCACAACTACTGTTTATACATCTTGGACTGGAGTGGGAACCACTTCACACACCATCACTGCT

**>*CtrALS3871*_951**

ATGAACCTAATTACATTTATTTTGTTATTGTCGTCCCTCATCACAATAGTAACACCCAAAGAAATCACTGGAGTATTTACAAGTTTTAATTCCTTAACTTATAATGATGCAGCTAATTATGGTGCTCAATGCCCGGGATATCCAACATGGATTGCAACTTTAGGTTGGTCTTTAGATGGTTCTGTTGCCTCTCCTGGTGACTCATTCACCTTGATTATGCCTTGTGTTTTCAAGTTTACTAGTTCAGAAACATCRGTGGACTTAACTGTTGATGGAGTAAGTTATGCTACTTGTAATTTGAATAACGGGGAAGAATTTACTACATTTTCTAGTATGTCTTGTGTTGTTAGTAGTGCTTTAACTTCTACTACTCAAGCTTTAGGTGCTGTTAGTATTCCATTCTCATTTAATGTTGGTGGTTCTGGTTCGTCCGTTGATTTAGAAGATGCCACTTGTTTTACTGCTGGTACCAATACTGTTACTTTCAAAGATGGTGATAACGAACTCTCGATCAATGCCGTTTTTGACAAAACCACTGCTTCAGTATCAGATGAAATTATTTCAGTTAGATCTGTTCCGTCGATTGGAAAATTGCAACAAATCTCTATTGCAAAAGATTGTCCAAGTGGCTATGGTAGTGGATATATGAGTATAATTATTAAAGAYAATACTGCTGTTATGGATTGCTCTTCAGTTCATATTGGTATTACTAATGAACTCAATGATTGGAATCAACCAATGAATTCTGAATCATTTTCTTACACTAAAAGCTGTTCAGCAACTGAGTTTATTGTTTCATTCACTGATATTGCAGCTGGTTATAGACCATTCATGGACTCATTCCTTACAACAACTGGCAATGCAAAATTAACTGTTGATTATCATTACGAGTATACTTGTAAAAATGGTGATACGGTTGCTGAAACTGACAGAAGAATTTTCAGTCCTTATACGAACAGTAATACTGGCTGTAGCGGTGTTGTTTTGGTTATAACCACCAGAACAGGAACACAAACTACTACTGCTGTTACCACTTTGCCATTCGATCCAAGTCAAGACCATACTAAAACAATTGAAGTTATTGAACCAATTCCAACAACAACAATCACTACTTCATACCTTGGTATCACAACTTCTTATAGTACAATCTCAGGCACTGTTGGTGGAACTGCTACTGTTATTGTTGATGAACCATATCATTCCACAACTACTGTTTATACATCTTGGACTGGAGTGGGAACCACTTCACACACCATCACTGCT

**>*CtrALS3871*_952**

ATGAACCTAATTACATTTATTTTGTTATTGTCGTCCCTCATCACAATAGTAACACCCAAAGAAATCACTGGAGTATTTACAAGTTTTAATTCCTTAACTTATAATGATGCAGCTAATTATGGTGCTCAATGCCCGGGATATCCAACATGGATTGCAACTTTAGGTTGGTCTTTAGATGGTTCTGTTGCCTCTCCTGGTGACTCATTCACCTTGATTATGCCTTGTGTTTTCAAGTTTACTAGTTCAGAAACATCRGTGGACTTAACTGTTGATGGAGTAAGTTATGCTACTTGTAATTTGAATAACGGGGAAGAATTTACTACATTTTCTAGTATGTCTTGTGTTGTTAGTAGTGCTTTAACTTCTACTACTCAAGCTTTAGGTGCTGTTAGTATTCCATTCTCATTTAATGTTGGTGGTTCTGGTTCGTCCGTTGATTTAGAAGATGCCACTTGTTTTACTGCTGGTACCAATACTGTTACTTTCAAAGATGGTGATAACGAACTCTCGATCAATGCCGTTTTTGACAAAACCACTGCTTCAGTATCAGATGAAATTATTTCAGTTAGATCTGTTCCGTCGATTGGAAAATTGCAACAAATCTCTATTGCAAAAGATTGTCCAAGTGGCTATGGTAGTGGATATATGAGTATAATTATTAAAGAYAATACTGCTGTTATGGATTGCTCTTCAGTTCATATTGGTATTACTAATGAACTCAATGATTGGAATCAACCAATGAATTCTGAATCATTTTCTTACACTAAAAGCTGTTCAGCAACTGAGTTTATTGTTTCATTCACTGATATTGCAGCTGGTTATAGACCATTCATGGACTCATTCCTTACAACAACTGGCAATGCAAAATTAACTGTTGATTATCATTACGAGTATACTTGTAAAAATGGTGATACGGTTGCTGAAACTGACAGAAGAATTTTCAGTCCTTATACGAACAGTAATACTGGCTGTAGCGGTGTTGTTTTGGTTATAACCACCAGAACAGGAACACAAACTACTACTGCTGTTACCACTTTGCCATTCGATCCAAGTCAAGACCATACTAAAACAATTGAAGTTATTGAACCAATTCCAACAACAACAATCACTACTTCATACCTTGGTATCACAACTTCTTATAGTACAATCTCAGGCACTGTTGGTGGAACTGCTACTGTTATTGTTGATGAACCATATCATTCCACAACTACTGTTTATACATCTTGGACTGGAGTGGGAACCACTTCACACACCATCACTGCT

**>*CtrALS3871*_1019**

ATGAACCTAATTACATTTATTTTGTTATTGTCGTCCCTCATCACAATAGTAACACCCAAAGAAATCACTGGAGTATTTACAAGTTTTAATTCCTTAACTTATAATGATGCAGCTAATTATGGTGCTCAATGCCCGGGATATCCAACATGGATTGCAACTTTAGGTTGGTCTTTAGATGGTTCTGTTGCCTCTCCTGGTGAYWCATTCACCTTGATTATGCCTTGTGTTTTCAARTTTACTAGTTCAGAAACATCRGTGGACTTAACTGTTGATGGAGTAAGTTATGCTACTTGTAATTTGAATAACGGGGAAGAATTTACTACATTTTCTAGTATGTCTTGTGTTGTTAGTAGTGCTTTAACTTCTACTACTCAAGCTTTAGGTGCTGTTAGTATTCCATTCTCATTTAATGTTGGTGGTTCTGGTTCGTCCGTTGATTTAGAAGATGCCACTTGTTTTACTGCTGGTACCAATACTGTTACTTTCAAAGATGGTGATAACGAACTCTCGATCAATGCCGTTTTTGACAAAACCACTGCTTCAGTATCAGATGAAATTATTTCAGTTAGATCTGTTCCGTYGATTGGAAAATTGCAACAAATMTCTATTGCAAAAGATTGTCCAAGTGGCTATGGTAGTGGATATATGAGTATAATTATTAAAGAYAATACTGCTGTTATGGATTGCTCTTCAGTTCATATTGGTATTACTAATGAACTCAATGATTGGAATCAACCAATGAATTCTGAATCATTTTCTTACACTAAAAGCTGTTCAGCAACTGAGTTTATTGTTTCATTCACTGATATTGCAGCTGGTTATAGACCATTCATGGACTCATTCCTTACAACAACTGGCAATGCAAAATTAACTGTTGATTATCATTACGAGTATACTTGTAAAAATGGTGATACGGTTGCTGAAACTGACAGAAGAATTTTCAGTCCTTATACGAACAGTAATACTGGCTGTAGCGGTGTTGTTTTGGTTATAACCACCAGAACAGGAACACAAACTACTACTGCTGTTACCACTTTGCCATTCGATCCAAGTCAAGACCATACTAAAACAATTGAAGTTATTGAACCAATTCCAACAACAACAATCACTACTTCATACCTTGGTATCACAACTTCTTATAGTACAATCTCAGGCACTGTTGGTGGAACTGCTACTGTTATTGTTGATGAACCATATCATTCCACAACTACTGTTTATACATCTTGGACTGGAGTGGGAACCACTTCACACACCATCACTGCT

**>*CtrALS3871*_1020**

ATGAACCTAATTACATTTATTTTGTTATTGTCGTCCCTCATCACAATAGTAACACCCAAAGAAATCACTGGAGTATTTACAAGTTTTAATTTCTTAACTTATAATGATGCAGCTAATTATGGTGCTCAATGCCCGGGATATCCAACATGGATTGCAACTTTAGGTTGGTCTTTAGATGGTTCTGTTGCCTCTCCTGGTGACTCATTCACCTTGATTATGCCTTGTGTTTTCAAGTTTACTAGTTCAGAAACATCGGTGGACTTAACTGTTGATGGAGTAAGTTATGCTACTTGTAATTTGAATAACGGGGAAGAATTTACTACATTTTCTAGTATGTCTTGTGTTGTTAGTAGTGCTTTAACTTCTACTACTCAAGCTTTAGGTGCTGTTAGTATTCCATTCTCATTTAATGTTGGTGGTTCTGGTTCGTCCGTTGATTTAGAAGATGCCACTTGTTTTACTGCTGGTACCAATACTGTTACTTTCAAAGATGGTGATAACGAACTCTCGATCAATGCCGTTTTTGACAAAACCACTGCTTCAGTATCAGATGAAATTATTTCAGTTAGATCTGTTCCGTCGATTGGAAAATTGCAACAAATCTCTATTGCAAAAGATTGTCCAAGTGGCTATGGTAGTGGATATATGAGTATAATTATTAAAGACAATACTGCTGTTATGGATTGCTCTTCAGTTCATATTGGTATTACTAATGAACTCAATGATTGGAATCAACCAATGAATTCTGAATCATTTTCTTACACTAAAAGCTGTTCAGCAACTGAGTTTATTGTTTCATTCACTGATATTGCAGCTGGTTACAGACCATTCATGGACTCATTCCTTACAACAACTGGCAATGCAAAATTAACTGTTGATTATCATTACGAGTATACTTGTAAAAATGGTGATACGGTTGCTGAAACTGACAGAAGAATTTTCAGTCCTTATACGAACAGTAATACTGGCTGTAGCGGTGTTGTTTTGGTTATAACCACCAGAACAGGAACACAAACTACTACTGCTGTTACCACTTTGCCATTCGATCCAAGTCAAGACCATACTAAAACAATTGAAGTTATTGAACCAATTCCAACAACAACAATCACTACTTCATACCTTGGTATCACAACTTCTTATAGTACAATCTCAGGCACTGTTGGTGGAACTGCTACTGTTATTGTTGATGAACCATATCATTCCACAACTACTGTTTATACATCTTGGACTGGAGTGGGAACCACTTCACACACCATCACTGCT

**>*CtrALS3871*_1021**

ATGAACCTAATTACATTTATTTTGTTATTGTCGTCCCTCATCACAATAGTAACACCCAAAGAAATCACTGGAGTATTTACAAGTTTTAATTCCTTAACTTATAATGATGCAGCTAATTATGGTGCTCAATGCCCGGGATATCCAACATGGATTGCAACTTTAGGTTGGTCTTTAGATGGTTCTGTTGCCTCTCCTGGTGACTCATTCACCTTGATTATGCCTTGTGTTTTCAAGTTTACTAGTTCAGAAACATCGGTGGACTTAACTGTTGATGGAGTAAGTTATGCTACTTGTAATTTGAATAACGGGGAAGAATTTACTACATTTTCTAGTATGTCTTGTGTTGTTAGTAGTGCTTTAACTTCTACTACTCAAGCTTTAGGTGCTGTTAGTATTCCATTCTCATTTAATGTTGGTGGTTCTGGTTCGTCCGTTGATTTAGAAGATGCCACTTGTTTTACTGCTGGTACCAATACTGTTACTTTCAAAGATGGTGATAACGAACTCTCGATCAATGCCGTTTTTGACAAAACCACTGCTTCAGTATCAGATGAAATTATTTCAGTTAGATCTGTTCCGTCGATTGGAAAATTGCAACAAATCTCTATTGCAAAAGATTGTCCAAGTGGCTATGGTAGTGGATATATGAGTATAATTATTAAAGACAATACTGCTGTTATGGATTGCTCTTCAGTTCATATTGGTATTACTAATGAACTCAATGATTGGAATCAACCAATGAATTCTGAATCATTTTCTTACACTAAAAGCTGTTCAGCAACTGAGTTTATTGTTTCATTCACTGATATTGCAGCTGGTTATAGACCATTCATGGACTCATTCCTTACAACAACTGGCAATGCAAAATTAACTGTTGATTATCATTACGAGTATACTTGTAAAAATGGTGATACGGTTGCTGAAACTGACAGAAGAATTTTCAGTCCTTATACGAACAGTAATACTGGCTGTAGCGGTGTTGTTTTGGTTATAACCACCAGAACAGGAACACAAACTACTACTGCTGTTACCACTTTGCCATTCGATCCAAGTCAAGACCATACTAAAACAATTGAAGTTATTGAACCAATTCCAACAACAACAATCACTACTTCATACCTTGGTATCACAACTTCTTATAGTACAATCTCAGGCACTGTTGGTGGAACTGCTACTGTTATTGTTGATGAACCATATCATTCCACAACTACTGTTTATACATCTTGGACTGGAGTGGGAACCACTTCACACACCATCACTGCT

**>*CtrALS3871*_3242**

ATGAACCTAATTACATTTATTTTGTTATTGTCGTCCCTCATCACAATAGTAACACCCAAAGAAATCACTGGAGTATTTACAAGTTTTAATTCCTTAACTTATAATGATGCAGCTAATTATGGTGCTCAATGCCCGGGATATCCAACATGGATTGCAACTTTAGGTTGGTCTTTAGATGGTTCTGTTGCCTCTYCTGGTGACTCATTCACCTTGATTATGCCTTGTGTTTTCAAGTTTACTAGTTCAGAAACATCRGTGGACTTAACTGTTGATGGAGTAAGTTATGCTACTTGTAATTTGAATAACGGGGAAGAATTTACTACATTTTCTAGTATGTCTTGTGTTGTTAGTAGTGCTTTAACTTCTACTACTCAAGCTTTAGGTGCTGTTAGTATTCCATTCTCATTTAATGTTGGTGGTTCTGGTTCGTCCGTTGATTTAGAAGATGCCACTTGTTTTACTGCTGGTACCAATACTGTTACTTTCAAAGATGGTGATAACGAACTCTCGATCAATGCCGTTTTTGACAAAACCACTGCTTCAGTATCAGATGAAATTATTTCAGTTAGATCTGTTCCGTCGATTGGAAAATTGCAACAAATCTCTATTGCAAAAGATTGTCCAAGTGGCTATGGTAGTGGATATATGAGTATAATTATTAAAGACAATACTGCTGTTATGGATTGCTCTTCAGTTCATATTGGTATTACTAATGAACTCAATGATTGGAATCAACCAATGAATTCTGAATCATTTTCTTACACTAAAAGCTGTTCAGCAACTGAGTTTATTGTTTCATTCACTGATATTGCAGCTGGTTATAGACCATTCATGGACTCATTCCTTACAACAACTGGCAATGCAAAATTAACTGTTGATTATCATTACGAGTATACTTGTAAAAATGGTGATACGGTTGCTGAAACTGACAGAAGAATTTTCAGTCCTTATACGAACAGTAATACTGGCTGTAGCGGTGTTGTTTTGGTTATAACCACCAGAACAGGAACACAAACTACTACTGCTGTTACCACTTTGCCATTCGATCCAAGTCAAGACCATACTAAAACAATTGAAGTTATTGAACCAATTCCAACAACAACAATCACTACTTCATACCTTGGTATCACAACTTCTTATAGTACAATCTCAGGCACTGTTGGTGGAACTGCTACTGTTATTGTTGATGAACCATATCATTCCACAACTACTGTTTATACATCTTGGACTGGAGTGGGAACCACTTCACACACCATCACTGCT

***CtrALS3882-1* (Amplified with primers Ctr3882NT-F and Ctr3882NT-R2)**

**>*CtrALS3882-1*_MYA-3404**

ATGAAGTTTATTACATTTGGTTTGTTATTGCTGTCTTTGCTTACCTTGGTGACACCGAAAGAAGTTACTGGTATTTTCACTTCTTTTGATTCATTAACATGGAACGAACAAACTACCCCTTTTAGTAGTCCTGCTAGTCCAACCTGGAGAGCAACTTTAGGTTGGTCATTGGATGGTACTAAACTCAACCAAGGTGATACTTTCACTTTAACAATGCCTTGTGTCTTCAAGTTCATCACTGATCAAACTACCATTGATTTAATGGCAAATGGTGTAAGCTATGCTACCTGTACTTTTCATGCTGGTGAAGAATTTACCACTTTCTCAACTGTTGCCTGTGTCGTCAATGACGCTTTGAAATCAAATATGCAAGTTACTGGTTCTGTTACAATTCCATTTACTTTTAATGTTGGTGGTACTGGTACTTCAGTTAGTTTAGAGGATTCCACTTGCTATACCGCTGGTAAGAACACTGTTATTTTCAAGGATGGTGATAACGAGCTTTCAACCATAGCAAACTTTGAACCTACAGATGCATCCAGAACTGAATTAATTACTAATGCTAGATCGATTCCTTCAATTAAAAGAACCAGTCATGTTATTATTGCTCCAGATTGTCCTAGCGGTTACAAGAGTGGTACTATAACTTTTGACACAAATAATGGTGCTGACATTGACTGTGGTCAGACTCACGTTGGTATGACGAATTTTATCAATCCATGGAACTACCCAACTAATTCAGAACAAAATTTTTCAAAGCAACCAACATGTACTAAAGGAAAATATACTCTTTCATTTCAAGATGTTCCTGCTGGATATAGACCATTCTTCGATGTTTTGGTGAAGCCTACTGGTAAAATGACGTTTTATTACAACTCGGATCTTGTCTGTGCCGACGGTACAACCTATAAAAAGGGTCTTGCATGGGATTGGGGTTCGTATCAGAATGATGTTGCTGATAGCAGTGGTGATGTTATAGTAATAACTACAAGAACAGGAACACAAACTACAACTGCTGTTACCACTTTGCCATTCGATCCAAGTAAAGACCATACTAAAACAATTGAAGTTATTGAACCAATTCCAACAACAACAATCACTACTTCATACCTTGGTATCACAACTTCTTATACTACAATCTCAGGCACTGTTGGTGGAACTGCTACTGTTATCGTTGATGAACCATATCATTCCACAACTACTGTTTATACATCTTGGACTGGAGTGGGAACCACTTCATACACCATCACTGCTTCCACTGATTCAATTGACACTGTTT

**>*CtrALS3882-1*_951**

ATGAAGTTTATTACATTTGGTTTGTTATTGCTGTCTTTGCTTACCTTGGTGACACCGAAAGAAGTTACTGGTATTTTCACTTCTTTTGATTCATTAACATGGAACGAACAAACTACCCCTTTTAGTAGTCCTGCTAGTCCAACCTGGAGAGCAACTTTAGGTTGGTCATTGGATGGTACTAAACTCAACCAAGGTGATACTTTCACTTTAACAATGCCTTGTGTCTTCAAGTTCATCACTGATCAAACTACCATTGATTTAATGGCAAATGGTGTAAGCTATGCTACCTGTACTTTTCATGCTGGTGAAGAATTTACCACTTTCTCAACTGTTGCCTGTGTCGTCAATGACGCTTTGAAATCAAATATGCAAGTTACTGGTTCTGTTACAATTCCATTTACTTTTAATGTTGGTGGTACTGGTACTTCAGTTAGTTTAGAGGATTCCACTTGCTATACCGCTGGTAAGAACACTGTTATTTTCAAGGATGGTGATAACGAGCTTTCAACCATAGCAAACTTTGAACCTACAGATGCATCCAGAACTGAATTAATTACTAATGCTAGATCGATTCCTTCAATTAAAAGAACCAGTCATGTTATTATTGCTCCAGATTGTCCTAGCGGTTACAAGAGTGGTACTATAACTTTTGACACAAATAATGGTGCTGACATTGACTGTGGTCAGACTCACGTTGGTATGACGAATTTTATCAATCCATGGAACTACCCAACTAATTCAGAACAAAATTTTTCAAAGCAACCAACATGTACTAAAGGAAAATATACTCTTTCATTTCAAGATGTTCCTGCTGGATATAGACCATTCTTCGATGTTTTGGTGAAGCCTACTGGTAAAATGACGTTTTATTACAACTCGGATCTTGTCTGTGCCGACGGTACAACCTATAAAAAGGGTCTTGCATGGGATTGGGGTTCGTATCAGAATGATGTTGCTGATAGCAGTGGTGATGTTATAGTAATAACTACAAGAACAGGAACACAAACTACAACTGCTGTTACCACTTTGCCATTCGATCCAAGTAAAGACCATACTAAAACAATTGAAGTTATTGAACCAATTCCAACAACAACAATCACTACTTCATACCTTGGTATCACAACTTCTTATACTACAATCTCAGGCACTGTTGGTGGAACTGCTACTGTTATCGTTGATGAACCATATCATTCCACAACTACTGTTTATACATCTTGGACTGGAGTGGGAACCACTTCATACACCATCACTGCTTCCACTGATTCAATTGACACTGTTT

**>*CtrALS3882-1*_952**

ATGAAGTTTATTACATTTGGTTTGTTATTGCTGTCTTTGCTTACCTTGGTGACACCGAAAGAAGTTACTGGTATTTTCACTTCTTTTGATTCATTAACATGGAACGAACAAACTACCCCTTTTAGTAGTCCTGCTAGTCCAACCTGGAGAGCAACTTTAGGTTGGTCATTGGATGGTACTAAACTCAACCAAGGTGATACTTTCACTTTAACAATGCCTTGTGTCTTCAAGTTCATCACTGATCAAACTACCATTGATTTAATGGCAAATGGTGTAAGCTATGCTACCTGTACTTTTCATGCTGGTGAAGAATTTACCACTTTCTCAACTGTTGCCTGTGTCGTCAATGACGCTTTGAAATCAAATATGCAAGTTACTGGTTCTGTTACAATTCCATTTACTTTTAATGTTGGTGGTACTGGTACTTCAGTTAGTTTAGAGGATTCCACTTGCTATACCGCTGGTAAGAACACTGTTATTTTCAAGGATGGTGATAACGAGCTTTCAACCATAGCAAACTTTGAACCTACAGATGCATCCAGAACTGAATTAATTACTAATGCTAGATCGATTCCTTCAATTAAAAGAACCAGTCATGTTATTATTGCTCCAGATTGTCCTAGCGGTTACAAGAGTGGTACTATAACTTTTGACACAAATAATGGTGCTGACATTGACTGTGGTCAGACTCACGTTGGTATGACGAATTTTATCAATCCATGGAACTACCCAACTAATTCAGAACAAAATTTTTCAAAGCAACCAACATGTACTAAAGGAAAATATACTCTTTCATTTCAAGATGTTCCTGCTGGATATAGACCATTCTTCGATGTTTTGGTGAAGCCTACTGGTAAAATGACGTTTTATTACAACTCGGATCTTGTCTGTGCCGACGGTACAACCTATAAAAAGGGTCTTGCATGGGATTGGGGTTCGTATCAGAATGATGTTGCTGATAGCAGTGGTGATGTTATAGTAATAACTACAAGAACAGGAACACAAACTACAACTGCTGTTACCACTTTGCCATTCGATCCAAGTAAAGACCATACTAAAACAATTGAAGTTATTGAACCAATTCCAACAACAACAATCACTACTTCATACCTTGGTATCACAACTTCTTATACTACAATCTCAGGCACTGTTGGTGGAACTGCTACTGTTATCGTTGATGAACCATATCATTCCACAACTACTGTTTATACATCTTGGACTGGAGTGGGAACCACTTCATACACCATCACTGCTTCCACTGATTCAATTGACACTGTTT

**>*CtrALS3882-1*_1019**

ATGAAGTTTATTACATTTGGTTTGTTATTGCTGTCTTTGCTTACCTTGGTGACACCGAAAGAAGTTACTGGTATTTTCACTTCTTTTGATTCATTAACATGGAACGAACAAACTACCCCTTTTAGTAGTCCTGCTAGTCCAACCTGGAGAGCAACTTTAGGTTGGTCATTGGATGGTACTAAACTCAACCAAGGTGATACTTTCACTTTAACAATGCCTTGTGTCTTCAAGTTCATCACTGATCAAACTACCATTGATTTAATGGCAAATGGTGTAAGCTATGCTACCTGTACTTTTCATGCTGGTGAAGAATTTACCACTTTCTCAACTGTTGCCTGTGTCGTCAATGACGCTTTGAAATCAAATATGCAAGTTACTGGTTCTGTTACAATTCCATTTACTTTTAATGTTGGTGGTACTGGTACTTCAGTTAGTTTAGAGGATTCCACTTGCTATACCGCTGGTAAGAACACTGTTATTTTCAAGGATGGTGATAACGAGCTTTCAACCATAGCAAACTTTGAACCTACAGATGCATCCAGAACTGAATTAATTACTAATGCTAGATCGATTCCTTCAATTAAAAGAACCAGTCATGTTATTATTGCTCCAGATTGTCCTAGCGGTTACAAGAGTGGTACTATAACTTTTGACACAAATAATGGTGCTGACATTGACTGTGGTCAGACTCACGTTGGTATGACGAATTTTATCAATCCATGGAACTACCCAACTAATTCAGAACAAAATTTTTCAAAGCAACCAACATGTACTAAAGGAAAATATACTCTTTCATTTCAAGATGTTCCTGCTGGATATAGACCATTCTTCGATGTTTTGGTGAAGCCTACTGGTAAAATGACGTTTTATTACAACTCGGATCTTGTCTGTGCCGACGGTACAACCTATAAAAAGGGTCTTGCATGGGATTGGGGTTCGTATCAGAATGATGTTGCTGATAGCAGTGGTGATGTTATAGTAATAACTACAAGAACAGGAACACAAACTACAACTGCTGTTACCACTTTGCCATTCGATCCAAGTAAAGACCATACTAAAACAATTGAAGTTATTGAACCAATTCCAACAACAACAATCACTACTTCATACCTTGGTATCACAACTTCTTATACTACAATCTCAGGCACTGTTGGTGGAACTGCTACTGTTATCGTTGATGAACCATATCATTCCACAACTACTGTTTATACATCTTGGACTGGAGTGGGAACCACTTCATACACCATCACTGCTTCCACTGATTCAATTGACACTGTTT

**>*CtrALS3882-1*_1020**

ATGAAGTTTATTACATTTGGTTTGTTATTGCTGTCTTTGCTTACCTTGGTGACACCGAAAGAAGTTACTGGTATTTTCACTTCTTTTGATTCATTAACATGGAACGAACAAACTACCCCTTTTAGTAGTCCTGCTAGTCCAACCTGGAGAGCAACTTTAGGTTGGTCATTGGATGGTACTAAACTCAACCAAGGTGATACTTTCACTTTAACAATGCCTTGTGTCTTCAAGTTCATCACTGATCAAACTACCATTGATTTAATGGCAAATGGTGTAAGCTATGCTACCTGTACTTTTCATGCTGGTGAAGAATTTACCACTTTCTCAACTGTTGCCTGTGTCGTCAATGACGCTTTGAAATCAAATATGCAAGTTACTGGTTCTGTTACAATTCCATTTACTTTTAATGTTGGTGGTACTGGTACTTCAGTTAGTTTAGAGGATTCCACTTGCTATACCGCTGGTAAGAACACTGTTATTTTCAAGGATGGTGATAACGAGCTTTCAACCATAGCAAACTTTGAACCTACAGATGCATCCAGAACTGAATTAATTACTAATGCTAGATCGATTCCTTCAATTAAAAGAACCAGTCATGTTATTATTGCTCCAGATTGTCCTAGCGGTTACAAGAGTGGTACTATAACTTTTGACACAAATAATGGTGCTGACATTGACTGTGGTCAGACTCACGTTGGTATGACGAATTTTATCAATCCATGGAACTACCCAACTAATTCAGAACAAAATTTTTCAAAGCAACCAACATGTACTAAAGGAAAATATACTCTTTCATTTCAAGATGTTCCTGCTGGATATAGACCATTCTTCGATGTTTTGGTGAAGCCTACTGGTAAAATGACGTTTTATTACAACTCGGATCTTGTCTGTGCCGACGGTACAACCTATAAAAAGGGTCTTGCATGGGATTGGGGTTCGTATCAGAATGATGTTGCTGATAGCAGTGGTGATGTTATAGTAATAACTACAAGAACAGGAACACAAACTACAACTGCTGTTACCACTTTGCCATTCGATCCAAGTAAAGACCATACTAAAACAATTGAAGTTATTGAACCAATTCCAACAACAACAATCACTACTTCATACCTTGGTATCACAACTTCTTATACTACAATCTCAGGCACTGTTGGTGGAACTGCTACTGTTATCGTTGATGAACCATATCATTCCACAACTACTGTTTATACATCTTGGACTGGAGTGGGAACCACTTCATACACCATCACTGCTTCCACTGATTCAATTGACACTGTTT

**>*CtrALS3882-1*_1021**

ATGAAGTTTATTACATTTGGTTTGTTATTGCTGTCTTTGCTTACCTTGGTGACACCGAAAGAAGTTACTGGTATTTTCACTTCTTTTGATTCATTAACATGGAACGAACAAACTACCCCTTTTAGTAGTCCTGCTAGTCCAACCTGGAGAGCAACTTTAGGTTGGTCATTGGATGGTACTAAACTCAACCAAGGTGATACTTTCACTTTAACAATGCCTTGTGTCTTCAAGTTCATCACTGATCAAACTACCATTGATTTAATGGCAAATGGTGTAAGCTATGCTACCTGTACTTTTCATGCTGGTGAAGAATTTACCACTTTCTCAACTGTTGCCTGTGTCGTCAATGACGCTTTGAAATCAAATATGCAAGTTACTGGTTCTGTTACAATTCCATTTACTTTTAATGTTGGTGGTACTGGTACTTCAGTTAGTTTAGAGGATTCCACTTGCTATACCGCTGGTAAGAACACTGTTATTTTCAAGGATGGTGATAACGAGCTTTCAACCATAGCAAACTTTGAACCTACAGATGCATCCAGAACTGAATTAATTACTAATGCTAGATCGATTCCTTCAATTAAAAGAACCAGTCATGTTATTATTGCTCCAGATTGTCCTAGCGGTTACAAGAGTGGTACTATAACTTTTGACACAAATAATGGTGCTGACATTGACTGTGGTCAGACTCACGTTGGTATGACGAATTTTATCAATCCATGGAACTACCCAACTAATTCAGAACAAAATTTTTCAAAGCAACCAACATGTACTAAAGGAAAATATACTCTTTCATTTCAAGATGTTCCTGCTGGATATAGACCATTCTTCGATGTTTTGGTGAAGCCTACTGGTAAAATGACGTTTTATTACAACTCGGATCTTGTCTGTGCCGACGGTACAACCTATAAAAAGGGTCTTGCATGGGATTGGGGTTCGTATCAGAATGATGTTGCTGATAGCAGTGGTGATGTTATAGTAATAACTACAAGAACAGGAACACAAACTACAACTGCTGTTACCACTTTGCCATTCGATCCAAGTAAAGACCATACTAAAACAATTGAAGTTATTGAACCAATTCCAACAACAACAATCACTACTTCATACCTTGGTATCACAACTTCTTATACTACAATCTCAGGCACTGTTGGTGGAACTGCTACTGTTATCGTTGATGAACCATATCATTCCACAACTACTGTTTATACATCTTGGACTGGAGTGGGAACCACTTCATACACCATCACTGCTTCCACTGATTCAATTGACACTGTTT

**>*CtrALS3882-1*_3242**

ATGAAGTTTATTACATTTGGTTTGTTATTGCTGTCTTTGCTTACCTTGGTGACACCGAAAGAAGTTACTGGTATTTTCACTTCTTTTGATTCATTAACATGGAACGAACAAACTACCCCTTTTAGTAGTCCTGCTAGTCCAACCTGGAGAGCAACTTTAGGTTGGTCATTGGATGGTACTAAACTCAACCAAGGTGATACTTTCACTTTAACAATGCCTTGTGTCTTCAAGTTCATCACTGATCAAACTACCATTGATTTAATGGCAAATGGTGTAAGCTATGCTACCTGTACTTTTCATGCTGGTGAAGAATTTACCACTTTCTCAACTGTTGCCTGTGTCGTCAATGACGCTTTGAAATCAAATATGCAAGTTACTGGTTCTGTTACAATTCCATTTACTTTTAATGTTGGTGGTACTGGTACTTCAGTTAGTTTAGAGGATTCCACTTGCTATACCGCTGGTAAGAACACTGTTATTTTCAAGGATGGTGATAACGAGCTTTCAACCATAGCAAACTTTGAACCTACAGATGCATCCAGAACTGAATTAATTACTAATGCTAGATCGATTCCTTCAATTAAAAGAACCAGTCATGTTATTATTGCTCCAGATTGTCCTAGCGGTTACAAGAGTGGTACTATAACTTTTGACACAAATAATGGTGCTGACATTGACTGTGGTCAGACTCACGTTGGTATGACGAATTTTATCAATCCATGGAACTACCCAACTAATTCAGAACAAAATTTTTCAAAGCAACCAACATGTACTAAAGGAAAATATACTCTTTCATTTCAAGATGTTCCTGCTGGATATAGACCATTCTTCGATGTTTTGGTGAAGCCTACTGGTAAAATGACGTTTTATTACAACTCGGATCTTGTCTGTGCCGACGGTACAACCTATAAAAAGGGTCTTGCATGGGATTGGGGTTCGTATCAGAATGATGTTGCTGATAGCAGTGGTGATGTTATAGTAATAACTACAAGAACAGGAACACAAACTACAACTGCTGTTACCACTTTGCCATTCGATCCAAGTAAAGACCATACTAAAACAATTGAAGTTATTGAACCAATTCCAACAACAACAATCACTACTTCATACCTTGGTATCACAACTTCTTATACTACAATCTCAGGCACTGTTGGTGGAACTGCTACTGTTATCGTTGATGAACCATATCATTCCACAACTACTGTTTATACATCTTGGACTGGAGTGGGAACCACTTCATACACCATCACTGCTTCCACTGATTCAATTGACACTGTTT

***CtrALS3882-2* (Amplified with primers Ctr3882NT-F and Ctr3882NT-R2)**

**>*CtrALS3882-2*_MYA34-4**

ATGAAGTTTATTACAATTGGTTTGTTAGTGTCGGCCCTATTCACAAAAGTAACCCCTAAAGAAATCACAGGGGTATTCACCAGTTTTAATTCCTTAACTTATTTTGATGCAGGTAATTATGGATACCAAGGTCCAGGGAATCCAACATGGACTTCAACTTTAGGTTGGTCATTAGATGGTTCTGTTGCCTCTCCTGGTGATACATTCACCTTGATTATGCCTTGTGTTTTCAAATTTACTAGTTCATCAACATCAGTGGACTTAACTGTTGATGGAGTAAGTTATGCTACTTGTAATTTGAATAACGGTGAAGAATTTACCACATTTTCAAGTATGTCATGTGTTGTTAGTAGTGCTTTAACTTCTACTACTCAAGCTCTTGGTACTGTTACTGTTCCATTCGCATTTAATATTGGTGGTTCTGGTTCGTCCGTTGATTTAGAAGATGCCACTTGTTTCACTTCTGGTACCAATACTGTTACTTTCAAAGATGGTGATAACGAACTCTCGATCAATGCCGTTTTTGACAAAACCACTGCTTCAGTATCAGATGAAATTATTTCAGTTAGATCTGTTCCGTCGATTGGAAAATTGCAACAACTCTCTATTGCAAAAGATTGTCCAAGTGGCTATGGTAGTGGATATATGAGTATAATTATTAAAGATAATACTGCTGTTATGGATTGCTCTTCAGTTCATATTGGTATTACTAATGAATTAAATGATTGGAATCAACCAATGAATTCTGAATCATTTTCTTACACTAAAAGCTGTTCAGCAACTGAGTTTATTGTTTCATTCACTGATATTGCAGCTGGTTATAGACCATTCATGGACTCATTCCTTACTACTACTGCCAATGCTGGATTTACTGTTGATTATCATTACGAGTATACTTGTAAAAATGGTGACACGATTACTAAAACTAACAGTAGAGTTTACAGTCCTTATATTAATGGTAATACTGATAGTAACGGTGCTATTTTGGTTATAACCACCAGAACAGGAACACAAACTACTACTGCTGTTTCTACCTTCCCATTCGATCCAAGTCAAGACCATACTAAAACAATTGAAGTTATTGAACCAATTCCAACAACAACAATCACTACTTCATACCTTGGTATCACAACTTCTTATAGTACAATCTCAGGCACTGTTGGTGGAACTGCTACTGTTATTGTTGATGAACCATATCATTCCACAACTACTGTTTATACATCTTGGACTGGAGTGGGAACCACTTCACACACCATCACTGCTTCCACTGATTCAATTGACACTGTTT

**>*CtrALS3882-2*_951**

ATGAAGTTTATTACAATTGGTTTGTTAGTGTCGGCCCTATTCACAAAAGTAACCCCTAAAGAAATCACAGGGGTATTCACCAGTTTTAATTCCTTAACTTATTTTGATGCAGGTAATTATGGATACCAAGGTCCAGGGAATCCAACATGGAYTKCAACTTTAGGTTGGTCWTTAGATGGTTCTGTTGCCTCTCCTGGTGAYWCATTCACCTTGATTATGCCTTGTGTTTTCAARTTTACTAGTTCAKMAACATCAGTGGACTTAACTGTTGATGGAGTAAGTTATGCTACTTGTAATTTGAATAACGGKGAAGAATTTACYACATTTTCWAGTATGTCATGTGTTGTTAGTAGTGCTTTAACTTCTACTACTCAAGCTYTWGGTRCTGTTASTRTTCCATTCKCATTTAATRTTGGTGGTTCTGGTTCGTCCGTTGATTTAGAAGATGCCACTTGTTTYACTKCTGGTACCAATACTGTTACTTTCAAAGATGGTGATAACGAACTCTCGATCAATGCCGTTTTTGACAAAACCACTGCTTCAGTATCAGATGAAATTATTTCAGTTAGATCTGTTCCGTCGATTGGAAAATTGCAACAAMTCTCTATTGCAAAAGATTGTCCAAGTGGCTATGGTAGTGGATATATGAGTATAATTATTAAAGAYAATACTGCTGTTATGGATTGCTCTTCAGTTCATATTGGTATTACTAATGAAYTMAATGATTGGAATCAACCAATGAATTCTGAATCATTTTCTTACACTAAAAGCTGTTCAGCAACTGAGTTTATTGTTTCATTCACTGATATTGCAGCTGGTTATAGACCATTCATGGACTCATTCCTTACWACWACTGSCAATGCWRRATTWACTGTTGATTATCATTACGAGTATACTTGTAAAAATGGTGAYACGRTTRCTRAAACTRACAGWAGARTTTWCAGTCCTTATAYKAAYRGTAATACTGRYWGTARCGGTGYTRTTTTGGTTATAACCACCAGAACAGGAACACAAACTACTACTGCTGTTWCTACYTTSCCATTCGATCCAAGTCAAGACCATACTAAAACAATTGAAGTTATTGAACCAATTCCAACAACAACAATCACTACTTCATACCTTGGTATCACAACTTCTTATAGTACAATCTCAGGCACTGTTGGTGGAACTGCTACTGTTATTGTTGATGAACCATATCATTCCACAACTACTGTTTATACATCTTGGACTGGAGTGGGAACCACTTCACACACCATCACTGCTTCCACTGATTCAATTGACACTGTTT

**>*CtrALS3882-2*_952**

ATGAAGTTTATTACAATTGGTTTGTTAGTGTCGGCCCTATTCACAAAAGTAACCCCTAAAGAAATCACAGGGGTATTCACCAGTTTTAATTCCTTAACTTATTTTGATGCAGGTAATTATGGATACCAAGGTCCAGGGAATCCAACATGGACTTCAACTTTAGGTTGGTCATTAGATGGTTCTGTTGCCTCTCCTGGTGATACATTCACCTTGATTATGCCTTGTGTTTTCAAATTTACTAGTTCATCAACATCAGTGGACTTAACTGTTGATGGAGTAAGTTATGCTACTTGTAATTTGAATAACGGTGAAGAATTTACCACATTTTCAAGTATGTCATGTGTTGTTAGTAGTGCTTTAACTTCTACTACTCAAGCTCTTGGTACTGTTACTGTTCCATTCGCATTTAATATTGGTGGTTCTGGTTCGTCCGTTGATTTAGAAGATGCCACTTGTTTCACTTCTGGTACCAATACTGTTACTTTCAAAGATGGTGATAACGAACTCTCGATCAATGCCGTTTTTGACAAAACCACTGCTTCAGTATCAGATGAAATTATTTCAGTTAGATCTGTTCCGTCGATTGGAAAATTGCAACAACTCTCTATTGCAAAAGATTGTCCAAGTGGCTATGGTAGTGGATATATGAGTATAATTATTAAAGATAATACTGCTGTTATGGATTGCTCTTCAGTTCATATTGGTATTACTAATGAATTAAATGATTGGAATCAACCAATGAATTCTGAATCATTTTCTTACACTAAAAGCTGTTCAGCAACTGAGTTTATTGTTTCATTCACTGATATTGCAGCTGGTTATAGACCATTCATGGACTCATTCCTTACTACTACTGCCAATGCTGGATTTACTGTTGATTATCATTACGAGTATACTTGTAAAAATGGTGACACGATTACTAAAACTAACAGTAGAGTTTACAGTCCTTATATTAATGGTAATACTGATAGTAACGGTGCTATTTTGGTTATAACCACCAGAACAGGAACACAAACTACTACTGCTGTTTCTACCTTCCCATTCGATCCAAGTCAAGACCATACTAAAACAATTGAAGTTATTGAACCAATTCCAACAACAACAATCACTACTTCATACCTTGGTATCACAACTTCTTATAGTACAATCTCAGGCACTGTTGGTGGAACTGCTACTGTTATTGTTGATGAACCATATCATTCCACAACTACTGTTTATACATCTTGGACTGGAGTGGGAACCACTTCACACACCATCACTGCTTCCACTGATTCAATTGACACTGTTT

**>*CtrALS3882-2*_1019**

ATGAAGTTTATTACAATTGGTTTGTTAGTGTCGGCCCTATTCACAAAAGTAACCCCTAAAGAAATCACAGGGGTATTCACCAGTTTTAATTCCTTAACTTATTTTGATGCAGGTAATTATGGATACCAAGGTCCAGGGAATCCAACATGGACTTCAACTTTAGGTTGGTCATTAGATGGTTCWGTTGCCTCTCCTGGTGATACATTCACCTTGATTATGCCTTGTGTTTTCAAATTTACTAGTTCATCAACATCAGTGGACTTAACTGTTGATGGAGTAAGTTATGCTACTTGTAATTTGAATAACGGTGAAGAATTTACCACATTTTCAAGTATGTCATGTGTTGTTAGTAGTGCTTTAACTTCTACTACTCAAGCTCTTGGTACTGTTACTGTTCCATTCGCATTTAATATTGGTGGTTCTGGTTCGTCCGTTGATTTAGAAGATGCCACTTGTTTCACTTCTGGTACCAATACTGTTACTTTCAAAGATGGTGATAACGAACTCTCGATCAATGCCGTTTTTGACAAAACCACTGCTTCAGTATCAGATGAAATTATTTCAGTTAGATCTGTTCCGTCGATTGGAAAATTGCAACAAMTCTCTATTGCAAAAGATTGTCCAAGTGGCTATGGTAGTGGATATATGAGTATAATTATTAAAGATAATACTGCTGTTATGGATTGCTCTTCAGTTCATATTGGTATTACTAATGAATTAAATGATTGGAATCAACCAATGAATTCTGAATCATTTTCTTACACTAAAAGCTGTTCAGCAACTGAGTTTATTGTTTCATTCACTGATATTGCAGCTGGTTATAGACCATTCATGGACTCATTCCTTACTACTACTGCCAATGCTGGATTTACTGTTGATTATCATTACGAGTATACTTGTAAAAATGGTGACACGATTACTAAAACTAACAGTAGAGTTTACAGTCCTTATATTAATGGTAATACTGATAGTAACGGTGCTATTTTGGTTATAACCACCAGAACAGGAACACAAACTACTACTGCTGTTTCTACCTTCCCATTCGATCCAAGTCAAGACCATACTAAAACAATTGAAGTTATTGAACCAATTCCAACAACAACAATCACTACTTCATACCTTGGTATCACAACTTCTTATAGTACAATCTCAGGCACTGTTGGTGGAACTGCTACTGTTATTGTTGATGAACCATATCATTCCACAACTACTGTTTATACATCTTGGACTGGAGTGGGAACCACTTCACACACCATCACTGCTTCCACTGATTCAATTGACACTGTTT

**>*CtrALS3882-2*_1020**

ATGAAGTTTATTACAATTGGTTTGTTAGTGTCGGCCCTATTCACAAAAGTAACCCCTAAAGAAATCACAGGGGTATTCACCAGTTTTAATTCCTTAACTTATTTTGATGCAGGTAATTATGGATACCAAGGTCCAGGGAATCCAACATGGACTTCAACTTTAGGTTGGTCATTAGATGGTTCAGTTGCCTCTCCTGGTGATACATTCACCTTGATTATGCCTTGTGTTTTCAAATTTACTAGTTCATCAACATCAGTGGACTTAACTGTTGATGGAGTAAGTTATGCTACTTGTAATTTGAATAACGGTGAAGAATTTACCACATTTTCAAGTATGTCATGTGTTGTTAGTAGTGCTTTAACTTCTACTACTCAAGCTCTTGGTACTGTTACTGTTCCATTCGCATTTAATATTGGTGGTTCTGGTTCGTCCGTTGATTTAGAAGATGCCACTTGTTTCACTTCTGGTACCAATACTGTTACTTTCAAAGATGGTGATAACGAACTCTCGATCAATGCCGTTTTTGACAAAACCACTGCTTCAGTATCAGATGAAATTATTTCAGTTAGATCTGTTCCGTCGATTGGAAAATTGCAACAACTCTCTATTGCAAAAGATTGTCCAAGTGGCTATGGTAGTGGATATATGAGTATAATTATTAAAGATAATACTGCTGTTATGGATTGCTCTTCAGTTCATATTGGTATTACTAATGAATTAAATGATTGGAATCAACCAATGAATTCTGAATCATTTTCTTACACTAAAAGCTGTTCAGCAACTGAGTTTATTGTTTCATTCACTGATATTGCAGCTGGTTATAGACCATTCATGGACTCATTCCTTACTACTACTGCCAATGCTGGATTTACTGTTGATTATCATTACGAGTATACTTGTAAAAATGGTGACACGATTACTAAAACTAACAGTAGAGTTTACAGTCCTTATATTAATGGTAATACTGATAGTAACGGTGCTATTTTGGTTATAACCACCAGAACAGGAACACAAACTACTACTGCTGTTTCTACCTTCCCATTCGATCCAAGTCAAGACCATACTAAAACAATTGAAGTTATTGAACCAATTCCAACAACAACAATCACTACTTCATACCTTGGTATCACAACTTCTTATAGTACAATCTCAGGCACTGTTGGTGGAACTGCTACTGTTATTGTTGATGAACCATATCATTCCACAACTACTGTTTATACATCTTGGACTGGAGTGGGAACCACTTCACACACCATCACTGCTTCCACTGATTCAATTGACACTGTTT

**>*CtrALS3882-2*_1021**

ATGAAGTTTATTACAATTGGTTTGTTAGTGTCGGCCCTATTCACAAAAGTAACCCCTAAAGAAATCACAGGGGTATTCACCAGTTTTAATTCCTTAACTTATTTTGATGCAGGTAATTATGGATACCAAGGTCCAGGGAATCCAACATGGACTTCAACTTTAGGTTGGTCATTAGATGGTTCTGTTGCCTCTCCTGGTGATACATTCACCTTGATTATGCCTTGTGTTTTCAAATTTACTAGTTCATCAACATCAGTGGACTTAACTGTTGATGGAGTAAGTTATGCTACTTGTAATTTGAATAACGGTGAAGAATTTACCACATTTTCAAGTATGTCATGTGTTGTTAGTAGTGCTTTAACTTCTACTACTCAAGCTCTTGGTACTGTTACTGTTCCATTCGCATTTAATATTGGTGGTTCTGGTTCGTCCGTTGATTTAGAAGATGCCACTTGTTTCACTTCTGGTACCAATACTGTTACTTTCAAAGATGGTGATAACGAACTCTCGATCAATGCCGTTTTTGACAAAACCACTGCTTCAGTATCAGATGAAATTATTTCAGTTAGATCTGTTCCGTCGATTGGAAAATTGCAACAACTCTCTATTGCAAAAGATTGTCCAAGTGGCTATGGTAGTGGATATATGAGTATAATTATTAAAGATAATACTGCTGTTATGGATTGCTCTTCAGTTCATATTGGTATTACTAATGAATTAAATGATTGGAATCAACCAATGAATTCTGAATCATTTTCTTACACTAAAAGCTGTTCAGCAACTGAGTTTATTGTTTCATTCACTGATATTGCAGCTGGTTATAGACCATTCATGGACTCATTCCTTACTACTACTGCCAATGCTGGATTTACTGTTGATTATCATTACGAGTATACTTGTAAAAATGGTGACACGATTACTAAAACTAACAGTAGAGTTTACAGTCCTTATATTAATGGTAATACTGATAGTAACGGTGCTATTTTGGTTATAACCACCAGAACAGGAACACAAACTACTACTGCTGTTTCTACCTTCCCATTCGATCCAAGTCAAGACCATACTAAAACAATTGAAGTTATTGAACCAATTCCAACAACAACAATCACTACTTCATACCTTGGTATCACAACTTCTTATAGTACAATCTCAGGCACTGTTGGTGGAACTGCTACTGTTATTGTTGATGAACCATATCATTCCACAACTACTGTTTATACATCTTGGACTGGAGTGGGAACCACTTCACACACCATCGCTGCTTCCACTGATTCAATTGACACTGTTT

**>*CtrALS3882-2*_3242**

ATGAAGTTTATTACAATTGGTTTGTTAGTGTCGGCCCTATTCACAAAAGTAACCCCTAAAGAAATCACAGGGGTATTCACCAGTTTTAATTCCTTAACTTATTTTGATGCAGGTAATTATGGATACCAAGGTCCAGGGAATCCAACATGGACTTCAACTTTAGGTTGGTCATTAGATGGTTCWGTTGCCTCTCCTGGTGATACATTCACCTTGATTATGCCTTGTGTTTTCAAATTTACTAGTTCATCAACATCAGTGGACTTAACTGTTGATGGAGTAAGTTATGCTACTTGTAATTTGAATAACGGTGAAGAATTTACCACATTTTCAAGTATGTCATGTGTTGTTAGTAGTGCTTTAACTTCTACTACTCAAGCTCTTGGTACTGTTACTGTTCCATTCGCATTTAATATTGGTGGTTCTGGTTCGTCCGTTGATTTAGAAGATGCCACTTGTTTCACTTCTGGTACCAATACTGTTACTTTCAAAGATGGTGATAACGAACTCTCGATCAATGCCGTTTTTGACAAAACCACTGCTTCAGTATCAGATGAAATTATTTCAGTTAGATCTGTTCCGTCGATTGGAAAATTGCAACAAMTCTCTATTGCAAAAGATTGTCCAAGTGGCTATGGTAGTGGATATATGAGTATAATTATTAAAGATAATACTGCTGTTATGGATTGCTCTTCAGTTCATATTGGTATTACTAATGAATTAAATGATTGGAATCAACCAATGAATTCTGAATCATTTTCTTACACTAAAAGCTGTTCAGCAACTGAGTTTATTGTTTCATTCACTGATATTGCAGCTGGTTATAGACCATTCATGGACTCATTCCTTACTACTACTGCCAATGCTGGATTTACTGTTGATTATCATTACGAGTATACTTGTAAAAATGGTGACACGATTACTAAAACTAACAGTAGAGTTTACAGTCCTTATATTAATGGTAATACTGATAGTAACGGTGCTATTTTGGTTATAACCACCAGAACAGGAACACAAACTACTACTGCTGTTTCTACCTTCCCATTCGATCCAAGTCAAGACCATACTAAAACAATTGAAGTTATTGAACCAATTCCAACAACAACAATCACTACTTCATACCTTGGTATCACAACTTCTTATAGTACAATCTCAGGCACTGTTGGTGGAACTGCTACTGTTATTGTTGATGAACCATATCATTCCACAACTACTGTTTATACATCTTGGACTGGAGTGGGAACCACTTCACACACCATCACTGCTTCCACTGATTCAATTGACACTGTTT
