# Supplementary material for: Pursuing Advances in DNA Sequencing Technology to Solve a Complex Genomic Jigsaw Puzzle: The Agglutinin-Like Sequence (ALS) Genes of Candida tropicalis
Source: Front Microbiol. 2021 Jan 20;11:594531. doi: 10.3389/fmicb.2020.594531 (PMC7856822; doi:10.3389/fmicb.2020.594531)
Supplement: Supplementary file 1 [file Data_Sheet_1.zip › SupplementaryTableS1.docx]

**SUPPLEMENTARY TABLE S1 |** Oligonucleotide primers used for PCR amplification and DNA sequencing in this study.

| **Gene**  **(GenBank Accession)** | **Primer Name** | **Alias** | **Sequence (5’-3’)** | **Location*** |
| --- | --- | --- | --- | --- |
| *CtrALS941*  (MH753531) | Ctr941NT-F | 1-1F | GGCTAATTGACCTTATTGGAG | -122 to -102 |
|  | Ctr941NTSq-F1 | 1-7F | CTACATGTTTTGTTGTTGGAG | 446 to 466 |
|  | Ctr941NTSq-R1 | 1-11R | AGATGGATCTGCAGGATTTG | 527 to 546 |
|  | Ctr941NTSq-F2 | 1-8F | CAATATAGGGTTGCTTATGAGG | 862 to 883 |
|  | Ctr941NTSq-R2 | 1-12R | CAGCTTCACTTCTTTGGTATG | 944 to 964 |
|  | Ctr941NT-R | 1-2R | CTCTTGAGTGTCTGATCCTG | 1442 to 1461 |
|  | Ctr941CT-F | 1-3F | CAGGATCAGACACTCAAGAG | 1442 to 1461 |
|  | Ctr941TR-R | 1-4R | GAGACATAGAACCCTCTCCTG | 7724 to 7744 |
|  | Ctr941CT-F | 1-5F | CAGGAGAGGGTTCTATGTCTC | 7724 to 7744 |
|  | Ctr941CTSq-R3 | 1-13R | GTACAACAC**C^1^**AGGTCAAGTAGAG | 8279 to 8301 |
|  | Ctr941CT-R | 1-6R | CACGAAACAACAAAGATATGG | +68 to +88 |
| *CtrALS1028*  (MH753521) | Ctr1028NT-F | 2-1F | GAGGGCTAAACTAACCTCGAAA | -186 to -165 |
|  | Ctr1028NTSq-F1 | 2-7F | GCTACTTGTACATTTCATTCGG | 286 to 307 |
|  | Ctr1028NTSq-R1 | 2-23R | CAAGTGATGGAATAACTCTGGC | 565 to 586 |
|  | Ctr1028NTSq-F2 | 2-22F | GATTCATTGATTAATGCTGCTACTG | 835 to 859 |
|  | Ctr1028NTSq-R2 | 2-24R | GGTTGGAATAGGAACAAGAACTTC | 1075 to 1098 |
|  | Ctr1028TR-F2 | 2-18F | GAACCATATCATATCACAACCACC | 1189 to 1212 |
|  | Ctr1028NT-R | 2-2R | CACCGTGACTAGCAGTTTCA | 1365 to 1384 |
|  | Ctr1028Gap-F1 | 2-20F | CATACATTACCACCATTACGGAAAC | 1994 to 2018 |
|  | Ctr1028CTSq-F4 | 2-16F | GTCGTCATTATCTGCTGCTACC | 2478 to 2499 |
|  | Ctr1028CT-R | 2-6R | GTGCCACTAGTATGGTGCTTTA | +170 to +191 |
| *CtrALS1030*  (MH753522) | Ctr1030NT-F | 3-1F | GAAATAGAACAGGACGAGCTTAC | -143 to -121 |
|  | Ctr1030NTSq-F1 | 3-7F | GGTACAATTACTTTCCCGTTTGC | 379 to 401 |
|  | Ctr1030NTSq-R1 | 3-13R | CCATGCTTGAAAGTGACTC | 473 to 491 |
|  | Ctr1030NTSq-F2 | 3-8F | CAATGAAGTTCCAGAAGGGTATC | 798 to 820 |
|  | Ctr1030NTSq-R2 | 3-14R | CATCACCCAGTGGAAGACTATC | 952 to 973 |
|  | Ctr1030NT-R | 3-2R | CTACGGAGTGAGATTCAGC | 1258 to 1276 |
|  | Ctr1030TR-F | 3-3F | GCTGAATCTCACTCCGTAG | 1258 to 1276 |
|  | Ctr1030CTSq-F4 | 3-10F | CTGACACAATTGGACAGC | 2554 to 2571 |
|  | Ctr1030CTSq-R3 | 3-15R | CCAGCAAATCAGACAGCAAATC | 2629 to 2650 |
|  | Ctr1030CTSq-F5 | 3-11F | GCTTTATCAATGAGGAGAGC | 3140 to 3159 |
|  | Ctr1030CTSq-F6 | 3-12F | CCGGAAGCCGAAGACAATG | 3577 to 3595 |
|  | Ctr1030CT-R | 3-6R | GTGTCTGATACAAACTGATAGATTC | +158 to +182 |
| *CtrALS1038*  (MK128125) | Ctr1038NT-F | 4-1F | GATTGAATTCCCTCGGATTTATCG | -71 to -48 |
|  | Ctr1038NTSq-F2 | 4-17F | ACCTTGTCATTGCTCCTGATT | 596 to 616 |
|  | Ctr1038NTSq-R1 | 4-18R | GTACCTGGGCACAGTCAATA | 672 to 691 |
|  | Ctr1038NTSq-F1 | 4-11F | CGGTGTCACTACTTCCTATACC | 1113 to 1134 |
|  | Ctr1038NT-R | 4-2R | GCAGTTTCGGT**G^2^**TA**G^2^**GTAGTAG | 1343 to 1364 |
|  | Ctr1038TR-Sq-F1 | 4-13F | GGTTCTGTTTCTGCTGC**T^3^**AC | 1435 to 1454 |
|  | Ctr1038Gap-R | 4-6R | AGATTCAGTTGGAAGTTCGTCG | 1977 to 1998 |
|  | Ctr1038TR-R2 | 4-8R | GTACCAGAAGGAGAATCAGC**A^4^**G | 2699 to 2720 |
|  | Ctr1038CT-F | 4-9F | C**T^5^**GCTGATTCTCCTTCTGGTAC | 2699 to 2720 |
|  | Ctr1038CTSq-R2 | 4-20R | CCTCAGAATGGGATGTGTCAA | 2834 to 2854 |
|  | Ctr1038CT-R | 4-10R | GGCTATTGATAATGAGAAAGTACTCC | +261 to +286 |
| *CtrALS1041*  (MK128127) | Ctr1041NT-F | 5-1F | AGATGTTGATTTTCCCTGTCAG | -100 to -79 |
|  | Ctr1041NTSq-F1 | 5-34F | GTTTTACTGCTGGTACCAATACT | 455 to 477 |
|  | Ctr1041NTSq-R1 | 5-35R | CACTTTCATACCCACTTGGAC | 620 to 640 |
|  | Ctr1041NTSq-F2 | 5-36F | CAACCGCCAATGCAAGATTTA | 851 to 871 |
|  | Ctr1041NTSq-R2 | 5-37R | TTAGTTTGGTCTACGCTTGAATC | 1045 to 1067 |
|  | Ctr1041TR1-F2 | 5-22F | TCACAACTTCCTATACCACCATTAC | 1127 to 1151 |
|  | Ctr1041NT-R | 5-2R | TCAACACTGGAAGATTGGATAAG | 1432 to 1454 |
|  | Ctr1041TRSq-R5 | 5-31R | AGTATCAACTGAGCTTGGGG | 1811 to 1830 |
|  | Ctr1041CT-F3 | 5-26F | GCTTCATCTTCCTTTGCTATAGAATC | 2092 to 2117 |
|  | Ctr1041TR2-R2 | 5-25R | TTTCGATCCGAGAACAGAAGAC | 2160 to 2181 |
|  | Ctr1041CT-F4 | 5-32F | GAGACTGGACTTGAAGAATCCA | 2791 to 2812 |
|  | Ctr1041CT-R3 | 5-30R | G**CA^6^**ACAGTAGTGGTGAATCCT | 2901 to 2921 |
|  | Ctr1041CTSq-R3 | 5-13R | GCAGATTCCTCAGCAGTATTAG | 3914 to 3935 |
|  | Ctr1041CT-R2 | 5-10R | TATAGTACACACTGCAAACCG | +145 to +165 |
| *CtrALS2228*  (MK128126) | Ctr2228NT-F | 6-1F | CCTTGTCTGGGCAATTGAGA | -56 to -37 |
|  | Ctr2228NTSq-F2 | 6-15F | CCATCCCTTAACAAAGTGTCAAATC | 580 to 604 |
|  | Ctr2228NTSq-R1 | 6-16R | ACACCGGAAGAATACCCATTT | 627 to 647 |
|  | Ctr2228NTSq-F1 | 6-9F | GTGGTACTGCTACTGTCATTGT | 1166 to 1187 |
|  | Ctr2228NT-R | 6-2R | GTAGTCAATGTTGGGTTTGGAAG | 1306 to 1328 |
|  | Ctr2228CT-F | 6-5F | AGCCAATCAACCCAACGA | 1736 to 1753 |
|  | Ctr2228CTSq-R2 | 6-7R | GCGTAAAAACCGACAAGGG | 1910 to 1928 |
|  | Ctr2228CTSq-F3 | 6-10F | AGTATGTCTGCTGCCTATAGCTC | 2296 to 2318 |
|  | Ctr2228CTSq-F2 | 6-8F | CACCATCTTCATCGTCATC | 2422 to 2441 |
|  | Ctr2228CT-R | 6-6R | ACAACAACGACGAAATAAGGG | +76 to +96 |
| *CtrALS2229*  (MH753523) | Ctr2229NT-F | 7-1F | TGCATTAAATCCACTTTATGGG | -73 to -52 |
|  | Ctr2229NTSq-F1 | 7-17F | GAGGTACTCATTGTTGCTCCA | 598 to 618 |
|  | Ctr2229NTSq-R1 | 7-18R | ATGATAACGTCACGGTCTGAAG | 662 to 683 |
|  | Ctr2229NT-R | 7-2R | GTGATTGTAGGATTTGGAACTG | 1310 to 1331 |
|  | Ctr2229TR-F | 7-3F | CAGTTCCAAATCCTACAATCAC | 1310 to 1331 |
|  | Ctr2229TR-R | 7-4R | TCGACATTATCGGTTTCCTC | 2254 to 2273 |
|  | Ctr2229CT-F | 7-5F | GAGGAAACCGATAATGTCGA | 2254 to 2273 |
|  | Ctr2229CTSq-R1 | 7-8R | CGGCACCACACTACTGGATG | 3212 to 3231 |
|  | Ctr2229CTSq-F2 | 7-9F | CATCCAGTAGTGTGGTGCCG | 3212 to 3231 |
|  | Ctr2229CTSq-R2 | 7-10R | CATGAAGGTCGGAACCGGTTC | 3820 to 3840 |
|  | Ctr2229CTSq-F5 | 7-16F | GAACCGGTTCCGACCTTCATG | 3820 to 3840 |
|  | Ctr2229CTSq-F4 | 7-15F | CCATCGAGGTAGTCGATTCAA | 4400 to 4420 |
|  | Ctr2229CT-R | 7-6R | GAATGACTAGTTAGGTACTTCCA | +83 to +105 |
| *CtrALS2293*  (MK182724) | Ctr2293NT-F | 13-1F | GCTCAATGCCACTAGAAACTC | -229 to -209 |
|  | Ctr2293NTSq-F2 | 13-12F | GCCACATGTGATCTTCATGCC | 286 to 306 |
|  | Ctr2293NTSq-R1 | 13-24R | GGTACTGATTGATGTATCACCATC | 490 to 513 |
|  | Ctr2293NTSq-F3 | 13-14F | CTACCCAACTTCAGCGG | 732 to 748 |
|  | Ctr2293NTSq-R2 | 13-25R | GGCCCAAGATATCTTTTGTGA | 922 to 942 |
|  | Ctr2293TR-F2 | 13-16F | GGGCCGGTTACACAAATAGTG | 938 to 958 |
|  | Ctr2293NT-R | 13-2R | CAACCGAGTCAGTAGAAGC | 1258 to 1276 |
|  | Ctr2293TR-R2 | 13-17R | CTCGATAACCCAGTAATAGACTCAGTG | 1656 to 1682 |
|  | Ctr2293CTSq-F1 | 13-7F | GGAAACTACCGAGTCAACAG | 1743 to 1762 |
|  | Ctr2293CTSq-R1 | 13-9R | GTGTTGAGCCAATGAGG | 1831 to 1847 |
|  | Ctr2293CTSq-R2 | 13-10R | GATTAGACTCACTTGATTC | 2419 to 2437 |
|  | Ctr2293CT-R | 13-6R | CGAAAGGAAGCAAACGATGATAC | +139 to +161 |
| *CtrALS3786*  (MK332912) | Ctr3786NT-F | 9-1F | GCTCATTTAGTCGGGATG | -80 to -63 |
|  | Ctr3786NTSq-F1 | 9-32F | TACTTCCAAAGAGTTGTTCCTTCA | 559 to 582 |
|  | Ctr3786NTSq-R1 | 9-33R | AATCTGGAATTGGAAGATGAGAAAC | 650 to 674 |
|  | Ctr3786TR1-F2 | 9-16F | ATACTACTACTACTGTTACTAGTCCCT | 1196 to 1222 |
|  | Ctr3786NT-R | 9-2R | TGACAGTTGGGTTTGGAG | 1301 to 1318 |
|  | Ctr3786CT-F | 9-7F | CTACTGAGTTCTCTGAGTCG | 4934 to 4953 |
|  | Ctr3786CT-F2 | 9-18F | GAGTTCTCTGAGTCGACTGTTG | 4939 to 4960 |
|  | Ctr3786TR1-R2 | 9-17R | TCGAGTGTTGAGGAAGAAGATTTA | 5151 to 5174 |
|  | Ctr3786CTSq-R3 | 9-14R | GAGAGTATAGAATCAGAAGAC | 5322 to 5342 |
|  | Ctr3786CT-R2 | 9-20R | ACTACCTAAGCACTTAAAGGAACA | +300 to +323 |
| *CtrALS3791*  (MK170233) | Ctr3791NT-F | 8-1F | CTCTCCTTTGCTTAAATTATCCCATC | -160 to -135 |
|  | Ctr2293NTSq-F2 | 13-12F | GCCACATGTGATCTTCATGCC | 286 to 306 |
|  | Ctr3791NTSq-R1 | 8-25R | ACCATCTTGGAAAGTAACAGTG | 474 to 495 |
|  | Ctr3791TR-F1 | 8-16F | ATGTGCTGGATCTTCCCAAA | 894 to 913 |
|  | Ctr3791NTSq-R2 | 8-26R | GTCGGGTTGAAAGGCAATG | 1031 to 1049 |
|  | Ctr3791NT-R | 8-2R | AGTGGTAGTGACAGTTGGATTT | 1305 to 1326 |
|  | Ctr3791CTSq-R6 | 8-24R | CCAGTTTCACTCGATAACCC | 1681 to 1700 |
|  | Ctr2293CTSq-F1 | 13-7F | GGAAACTACCGAGTCAACAG | 1752 to 1771 |
|  | Ctr3791CTSq-R2 | 8-12R | TCAGAAACAACCGAACTTGAG | 1914 to 1934 |
|  | Ctr2293CTSq-F2 | 13-8F | GACTGTTGTTGCTGTTTC | 2349 to 2366 |
|  | Ctr3791CTSq-R4 | 8-19R | CGAATAGAAATTATAATGAACCCGACG | +41 to +67 |
| *CtrALS3797*  (MN224675) | Ctr3797NT-F | 10-1F | GAACCACGCCTTTACTAGAGATG | -137 to -115 |
|  | Ctr3797NTSq-F1 | 10-81F | GAAGGTGCTGGTACTTTGAATG | 523 to 544 |
|  | Ctr3797NTSq-R1 | 10-82R | AACCCAATAGTACCTGATTTATACCC | 622 to 647 |
|  | Ctr3797TR-F1 | 10-3F | TATGCGGTGACATACACTTTCA | 859 to 880 |
|  | Ctr3797NT-R | 10-2R | CCGTTAACATATCCGGACCAA | 930 to 950 |
|  | Ctr3797NTSq-F2 | 10-73F | CCATACCATTCTACCACTACGG | 1183 to 1204 |
|  | Ctr3797NTSq-R2 | 10-74R | CCGTAGTGGTAGAATGGTATGG | 1183 to 1204 |
|  | Ctr3797rich-F | 10-60F | CTACCTACACTGAAACCGATACTG | 5300 to 5323 |
|  | Ctr3797TR1-R2 | 10-68R | CAGACTTTCCACACTACTGG | 5429 to 5448 |
|  | Ctr3797TR-F2 | 10-5F | CAGATGATGAAGCGCCTTTG | 5474 to 5493 |
|  | Ctr3797TRSq-F1 | 10-76F | ACTGTTATAGAATCCGGCGC | 5617 to 5636 |
|  | Ctr3797TRSq-R1 | 10-77R | GTTTCAACAATTGCGCCGG | 5630 to 5648 |
|  | Ctr3797TRSq-R2 | 10-80R | AATACAAGTAGGACACTCTGAGG | 6767 to 6789 |
|  | Ctr3797Gap-F | 10-7F | ACTACGTTAACTCCACTGTTCC | 6896 to 6917 |
|  | Ctr3797GapSq-R1 | 10-67R | GAAAGTCTGACGAAGCAGC | 6970 to 6988 |
|  | Ctr3797Sq-F21 | 10-52F | GCTTCTACCTCAACGGCTAAA | 7354 to 7374 |
|  | Ctr3797Gap-R | 10-8R | GGTTCACCGCCATTCATTTC | 7567 to 7586 |
|  | Ctr3797GapSq-F1 | 10-66F | GAAATGAATGGCGGTGAACC | 7567 to 7586 |
|  | Ctr3797CT-R | 10-10R | CGATAAACGAAATCCCACGAATAA | +56 to +79 |
| *CtrALS3871*  (MH753524) | Ctr3891Up-F1 | 11-26F | CGGCGTCAACAGATTGC | -998 to -982 |
|  | Ctr3871NT-F | 11-1F | CCGGTTTAGGACGAATTTGC | -59 to -40 |
|  | Ctr3871NTSq-F1 | 11-9F | CCATTCTCATTTAATGTTGGTGG | 397 to 419 |
|  | Ctr3871NTSq-R1 | 11-11R | GTAACAGTATTGGTACCAGC | 463 to 482 |
|  | Ctr3871NTSq-F2 | 11-10F | GACCATTCATGGACTCATTCC | 824 to 844 |
|  | Ctr3871NTSq-R2 | 11-12R | CAGTTTCAGCAACCGTATCACC | 907 to 928 |
|  | Ctr3871NTSq-R3 | 11-25R | CAAAACAACACCGCTACAGC | 968 to 987 |
|  | Ctr3871NT-R | 11-2R | AGCAGTGATGGTGTGTGAAG | 1244 to 1263 |
|  | Ctr3871TR-F | 11-3F | CTTCACACACCATCACTGCT | 1244 to 1263 |
|  | Ctr3871TR-F4 | 11-24F | GGCTACCACCATAACCCAC | 1998 to 2016 |
|  | Ctr3871CTSq-R1 | 11-17R | GTATGTCACTACTAGTTTTGGC | 2998 to 3019 |
|  | Ctr3871CTSq-F2 | 11-14F | CAATCCCAAGGTGTTTCACAAG | 3487 to 3508 |
|  | Ctr3871CTSq-R2 | 11-18R | GAACTACCTTGAGATCTGGATG | 3590 to 3611 |
|  | Ctr3871CTSq-R4 | 11-20R | GTTGACCACCATGACCAG | 4628 to 4645 |
|  | Ctr3871CT-R2 | 11-6R | ACTGGGAAACCGAAACTAATG | +166 to +186 |
| *CtrALS3882-1*  (MH753525) | Ctr3882Up-F1 | 12-33F | GGGAAGATGTTATATTGACACCTC | -3294 to -3271 |
|  | Ctr3882Up-F2 | 12-54F | GGAATGGCAACATTGATCGAC | -2421 to -2401 |
|  | Ctr3882Up-F3 | 12-29F | GAGGCGGATGTGTGTTATGA | -1159 to -1140 |
|  | Ctr3882NT-F | 12-1F | GTGGATGTATTCCCACCAATTTAG | -75 to -52 |
|  | Ctr3882NTSq-R1 | 12-16R | GCATCTGTAGGTTCAAAGTTTGC | 514 to 536 |
|  | Ctr3882TR-F1 | 12-3F | GCTGATAGCAGTGGTGATGT | 955 to 974 |
|  | Ctr3882NT-R2 | 12-19R | TGGGTGGCAGTTTCAGTATAG | 1350 to 1370 |
|  | Ctr3882TR1-F2 | 12-20F | GTGGTACTGATACTGTTCATGTTG | 1481 to 1504 |
|  | Ctr3882TR2-F3 | 12-22F | CGAATCTCAAGGTTCTCCTGAA | 3078 to 3099 |
|  | Ctr3882TR1-R2 | 12-21R | GTAATGGTGACACCTTCTGTATGA | 3135 to 3158 |
|  | Ctr3871CTSq-R2 | 11-18R | GAACTACCTTGAGATCTGGATG | 3473 to 3494 |
|  | Ctr3871CTSq-R4 | 11-20R | GTTGACCACCATGACCAG | 4493 to 4510 |
|  | Ctr3882CT-R | 12-8R | ACTGGAAACCGAAACTAATGAA | +164 to +185 |
| *CtrALS3882-2*  (MN893367) | Ctr3882Up-F1 | 12-33F | GGGAAGATGTTATATTGACACCTC | -3311 to -3288 |
|  | Ctr3882Up-F2 | 12-54F | GGAATGGCAACATTGATCGAC | -2437 to -2417 |
|  | Ctr3882Up-F3 | 12-29F | GAGGCGGATGTGTGTTATGA | -1161 to -1142 |
|  | Ctr3882Up-F4 | 12-62F | GAAAGCAGAGCAATATTATGCTTC | -159 to -136 |
|  | Ctr3882NT-F | 12-1F | GTGGATGTATTCCCACCAATTTAG | -75 to -52 |
|  | Ctr3882NTSq-F1 | 12-24F | GTGCTTTAACTTCTACTACTCAAGC | 353 to 377 |
|  | Ctr3882NTSq-R1 | 12-25R | TCGAGAGTTCGTTATCACCATC | 490 to 511 |
|  | Ctr3882NTSq-F2 | 12-26F | GCTGTTCAGCAACTGAGTT | 770 to 788 |
|  | Ctr3882NTSq-R2 | 12-27R | TCACCATTTTTACAAGTATACTCGTA | 886 to 911 |
|  | Ctr3882NTSq-R3 | 12-36R | GGATCGCACAATGGGAAGGTAGA | 1030 to 1049 |
|  | Ctr3882NTSq-F2 | 12-28F | GGTGGAACTGCTACTGTTATT | 1162 to 1182 |
|  | Ctr3882NT-R2**^7^** | 12-19R | TG**G**GT**G**GC**A**GTTTCAGT**A**TAG | 1359 to 1381 |
|  | Ctr3882TR1-R3 | 12-47R | GGTTGAATAACTAAAACAGTGTCAGTC | 2682 to 2708 |
|  | Ctr3882TR2-F3 | 12-22F | CGAATCTCAAGGTTCTCC**T^8^**GAA | 3195 to 3216 |
|  | Ctr3882TR1-R2 | 12-21R | GTAATGGTGACACCTTCTGTATGA | 3252 to 3275 |
|  | Ctr3882TR2-R4 | 12-49R | TCTGGAGAAACAGTAGAGTCG | 3570 to 3590 |
|  | Ctr3882TR2-R3 | 12-23R | ATGAGGCAGAACCTTCATAAGT | 4360 to 4381 |
|  | Ctr3882CT-Seq-F1 | 12-31F | ACTTATGAAGGTTCTGCCTCAT | 4360 to-4381 |
|  | Ctr3882CT-R | 12-8R | ACTGGAAACCGAAACTAATGAA | +164 to +185 |
|  | Ctr3882Dn-F1**^9^** | 12-57R | ATGAGTTGTGAACCCCGC | +1711 to +1728 |
|  | Ctr3882Dn-F2**^9^** | 12-58R | GTTGTTCTTGTGGTTGAGTCA | +2008 to +2028 |

*Primer location was relative to the GenBank accession number listed in the first column. Negative signs indicated primers that were upstream of the coding region while plus signs denoted primers downstream of the coding region. Sequences upstream and downstream of the coding region were located on *Candida* Genome Database ([www.candidagenome.org](http://www.candidagenome.org)) or from larger contigs in GenBank.

Superscripts were added throughout the table to explain differences between initial primers and validated data resulting from Sanger sequencing.

^1^C was not in the original primer but C was added after Sanger sequence verification of the region.

^2^G was used in each of these positions in the original primer. Sanger sequencing revealed T/C diploid sequence at each of these positions. The final sequence was deposited with T in place of each G.

^3^T was used in the original primer, but Sanger sequencing revealed an A in this position.

^4^A was used in the original reverse primer but T was indicated by Sanger sequencing.

^5^T was used in the original primer but A was revealed by Sanger sequencing.

^6^The CA from the original genome assembly was corrected to TG in the deposited gene sequence.

^7^The Sanger-sequenced region showed TG**A**GT**A**GC**C**GTTTCAGT**G**TAG.

^8^T was used in the original primer but Sanger sequencing indicated it should be A.

^9^The location of this primer might not be completely accurate since the actual PCR size using 12-62F/12-58R was different than expected.
