# Supplementary material for: Pursuing Advances in DNA Sequencing Technology to Solve a Complex Genomic Jigsaw Puzzle: The Agglutinin-Like Sequence (ALS) Genes of Candida tropicalis
Source: Front Microbiol. 2021 Jan 20;11:594531. doi: 10.3389/fmicb.2020.594531 (PMC7856822; doi:10.3389/fmicb.2020.594531)
Supplement: Supplementary file 1 [file Data_Sheet_1.zip › SupplementaryTableS2.docx]

**SUPPLEMENTARY TABLE S2 |** TaqMan *ALS* and control assays.

| **Gene** | **Forward Primer (5’ – 3’)** | **Reverse Primer (5’ – 3’)** | **Probe (5’ – 3’)** | **Product (bp)** | **PCR Eff. (%)** |
| --- | --- | --- | --- | --- | --- |
| *CtrALS941* | CGCTTGCAACAGTTCCAA | CCCATACAGATCAGCTTCACTT | ATCCACCAACACAAGTATATTTAACCTCATAAGCAACCC | 140 | 97 |
| *CtrALS1028* | ATGCTGCTACTGATTATACTTTTACTATTAG | GTTGATTCAAGATAAGTACTAGTTGTAAC | CACATGTGCTACCGGTGATTATCATGATAAATCTATTACTAAAAA | 172 | 95 |
| *CtrALS1030* | GTTTTGTTTTCTCATACTGCCAG | ATCACCCAGTGGAAGACTATC | AAATGAATATGTTGGGGCTGATGGGGTTACA | 138 | 97 |
| *CtrALS1038* | AAATGGGTTTTGACTACAAGTATAGTG | CCCATTACTATCGGCTGTTTG | AGATGGTGAATCTCTTGAGCATCCTACTCACTATGAC | 113 | 99 |
| *CtrALS1041* | GCAACCGCCAATGCAAG | CCCATTACTATCAGTATTACTGTTGA | GCATAAACTCTACTATTAGTTTTATCAACCGTGTCACCATTT | 129 | 99 |
| *CtrALS2228* | AGTGTCCTATCTGCTTTTCTTGG | CGGCCAAACTATTTCTATATGGAG | CACTTGCAGTGGTAGTAGATCAAGTTCTGTGACAAA | 133 | 98 |
| *CtrALS2229* | CCATTTCTACTGGTAACTTTAAGCAA | GTGACGGGAATCATGATACCA | CGATTCAGAATACATGTGAAAAAGACACGTTTAATGATCAATCA | 173 | 97 |
| *CtrALS2293* | CCCAACTTCAGCGGATTCC | TATCTTTTGTGAATCATCTCTTTGGA | ACCTTCACACCTGTATTTTGCAGTATATTGTATAGCATAATCC | 199 | 97 |
| *CtrALS3786* | GCCAACTACGGCTGATTCT | CCAAGTGACCGATTTCGTAAT | TTGGGTAGATCCAGTACATTGATATTTTGCAGTATATTGTATAGC | 205 | 100 |
| *CtrALS3791* | CCCAACTTCAGCGGATTCC | CCAAGTGACCGATTTCGAAG | TGCATTGGTTCAAGCTCCATCTTCGGAT | 205 | 97 |
| *CtrALS3797* | CCTATATTAAAACACCTACTTCCACA | TTCAGAATCTGCATCACCGTTA | TCCATCGGCACAAACATATTTGAAAGTGTATGTCACC | 134 | 97 |
| *CtrALS3871* | AACAACTGGCAATGCAAAATTAAC | GCTACAGCCAGTATTACTGTTCG | TGAAAATTCTTCTGTCAGTTTCAGCAACCGTATCAC | 127 | 98 |
| *CtrALS3882-1* | TTTTGGTGAAGCCTACTGGT | ACCACTGCTATCAGCAACATC | CGGTACAACCTATAAAAAGGGTCTTGCATGGGAT | 134 | 97 |
| *CtrALS3882-2* | TACTACTGCCAATGCTGGATTT | CACCGTTACTATCAGTATTACCATTAA | GGTGACACGATTACTAAAACTAACAGTAGAGTTTACAGTCC | 131 | 99 |
| *CaALS1* | AATGGAATTCAGATTAAATATCAAAATGTA | GGTTTACTTTGCAGACGACTG | TCTGCTACAGATGTTAACCAATATACTTTAGCATATACCAATGAT | 146 | 99 |
| *CaALS2* | TCATAGTTCTCACTGGTGACA | TGTTCTGGCCTGACACC | TTCCGGTATCGTCTGATTCATTAAGTTACAATAAAACTTGTT | 213 | 99 |
| *CaALS3* | CATCTAATGGTATCTTTATCACATATAAAAAC | CCGTTAGATCCAGCATCACTATT | TTCGTACACCTTGTCGTATGCTAATGAATATACTTGTGCTGGT | 202 | 98 |
| *CaALS4* | CGACTGCTGGTACTGGTG | AGCTGACTCGTGTTGCA | ATCCAATTTCACTGGAATCTTTTTCTTACACAAAGACCTGTA | 210 | 102 |
| *CaALS5* | AAGCTGTTCATCTTTTGGTATCTC | TGCATGTTGCCAATAATCATCAA | TCTCCCTCAGATAATAACCAGTATCAATTGTCGTATAAAAATGAC | 157 | 99 |
| *CaALS6* | GTACACCAAACAGTTTTATTATTACTTATG | GATCATTTCCCTTTTTGCCATCC | CTTACGTGAAAAAATCAGCAACAGCAACGAATGG | 159 | 97 |
| *CaALS7* | TTCTGTTCCCTTCGATCATACTA | CACAGGCAAACTCATTTGTATATTT | TGCGATTGTTCAAATACCAACGACAGAACCT | 158 | 97 |
| *CaALS9* | AATTTTCCAGTAAGTTCAGAATCATTT | GGATCAACCACAGTATTGCC | CATCAACAAAAGGRCGATACCCAGCAGGAAC | 197 | 95 LA; 96 SA* |
| *CdALS64210* | GTCTACAGGTATTACAATCACATACAG | TTTATGTTGCACGCTACCAC | CATTGGTATATGACAAGGTATACAGGTTAACATCCGAAGC | 148 | 100 |
| *CdALS64220* | CTGCAGAAAATGTGGGTAGATAC | TGCCTCACTATTCTTATATCCCC | TGACTATACCTGTAACAATGGCTACTCTGTAGTTGATCC | 119 | 102 |
| *CdALS64610* | CTGCGCTCACCTCATATACTA | GCATTATCATAGCCAAGCCAA | AGAAGCATCAACAGGGCGAGCACC | 106 | 100 |
| *CdALS64800* | CACCTACTGGTATTTCTATCACATATG | CTTCGCTACTATTGTATCCGGT | TGCTTCAGGAGTTAAACTGTACGCTTTGTCATATGCT | 189 | 97 |
| *CdALS86150* | GCACATCACGTGCTCTTTATATT | AGGCCATTAACACAAGTAAACTC | TGTGGACGCGATCGTTCAAATACCAACA | 139 | 98 |
| *CdALS86290* | GTTATCGTCCTTTTATTGATACATATGTG | ATTGGTGTACGAAGTCCAAAAG | CAACAACATCGACCGGATTTAATTTGAATTACACGAATT | 146 | 98 |
| Pan*ACT1* | AAGCYGCTGGTATTGACC | HGAVGAWGGAGCCAARGC | TTRTACGGTAACATTGTYATGTCTGGTGGTACYAC | 164 | 98 |
| Pan*TEF1* | AAGAGGTATCACCATYGAYATYGC | GCACARTCAGCTTGRGAAGTAC | ATCATRTTCTTGATGAAATCTCTGTGACCTGGRGCRTC | 132 | 99 |

*LA indicates the large allele of *CaALS9* (*CaALS9-1*; GenBank accession number AY269423.1); SA is the small allele (*CaALS9-2*; GenBank accession number AY269422.1). The alleles are 89% identical in the 5’ end of the gene (Zhao et al., 2007). The TaqMan assay was designed to recognize both alleles equally.
