# Supplementary material for: Pursuing Advances in DNA Sequencing Technology to Solve a Complex Genomic Jigsaw Puzzle: The Agglutinin-Like Sequence (ALS) Genes of Candida tropicalis
Source: Front Microbiol. 2021 Jan 20;11:594531. doi: 10.3389/fmicb.2020.594531 (PMC7856822; doi:10.3389/fmicb.2020.594531)
Supplement: Supplementary file 1 [file Data_Sheet_1.zip › SupplementaryTableS3.docx]

**SUPPLEMENTARY TABLE S3 |** List of cloned 5’ end fragments from *C. tropicalis* and *C. dubliniensis* *ALS* genes used as controls in TaqMan assays*.

Strain Forward Forward Primer Reverse Reverse Primer

Gene ID Primer Name Sequence (5’-3’) Primer Name Sequence (5’-3’)

*CtrALS941* 3593 Ctr941NT-F GGCTAATTGACCTTATTGGAG Ctr941NT-R CTCTTGAGTGTCTGATCCTG

*CtrALS1028* 3594 Ctr1028NT-F GAGGGCTAAACTAACCTCGAAA Ctr1028NT-R CACCGTGACTAGCAGTTTCA

*CtrALS1030* 3595 Ctr1030NT-F GAAATAGAACAGGACGAGCTTAC Ctr1030NT-R CTACGGAGTGAGATTCAGC

*CtrALS1038* 3596 Ctr1038NT-F GATTGAATTCCCTCGGATTTATCG Ctr1038NT-R GCAGTTTCGGTGTAGGTAGTAG

*CtrALS1041* 3597 Ctr1041NT-F AGATGTTGATTTTCCCTGTCAG Ctr1041NT-R TCAACACTGGAAGATTGGATAAG

*CtrASL2228* 3598 Ctr2227NT-F CCTTGTCTGGGCAATTGAGA Ctr2227NT-R GTAGTCAATGTTGGGTTTGGAAG

*CtrALS2229* 3599 Ctr2229NT-F TGCATTAAATCCACTTTATGGG Ctr2229NT-R GTGATTGTAGGATTTGGAACTG

*CtrALS2293* 3605 Ctr2293NT-F GCTCAATGCCACTAGAAACTC Ctr2293NT-R CAACCGAGTCAGTAGAAGC

*CtrALS3786* 3600 Ctr3786NT-F GCTCATTTAGTCGGGATG Ctr3786NT-R TGACAGTTGGGTTTGGAG

*CtrALS3791* 3601 Ctr3791NT-F CTCTCCTTTGCTTAAATTATCCCATC Ctr3791NT-R AGTGGTAGTGACAGTTGGATTT

*CtrALS3797* 3602 Ctr3797NT-F GAACCACGCCTTTACTAGAGATG Ctr3786NT-R TGACAGTTGGGTTTGGAG

*CtrALS3871* 3603 Ctr3871NT-F CCGGTTTAGGACGAATTTGC Ctr3871NT-R AGCAGTGATGGTGTGTGAAG

*CtrALS3882-1* 3604 Ctr3882NT-F GTGGATGTATTCCCACCAATTTAG Ctr3882NT-R2 TGGGTGGCAGTTTCAGTATAG

*CtrALS3882-2* 3641 Ctr3882NT-F GTGGATGTATTCCCACCAATTTAG Ctr3882NT-R2 TGGGTGGCAGTTTCAGTATAG

*CdALS64210* 3612 Cd64210NT-F AAGGGAATGGAAAGAGATGCT Cd64210NT-R ACAGTTGTGGTAGTGTGATATGG

*CdALS64220* 3613 Cd64220NT-F GAAGACTAGAAAGGCTCTCAGC Cd64220NT-R TAGTTGTAGGCAGTGGAACTTG

*CdALS64610* 3615 Cd64610NT-F GGGATACTTTGGGCAAACTTGT Cd64610NT-R CAGTTGTGGTAGTGTGATACGG

*CdALS64800* 3614 Cd64800NT-F ATAAGGAGGTACTTTGGGCAAG Cd64800NT-R GTAGTGGAGACTGTTGGGTTTG

*CdALS86150* 3616 Cd86150NT-F CCGCTTCAAAGATTCTGGATTT Cd86150NT-R GGAGAGGCGCTTGTGAATA

*CdALS86290* 3617 Cd86290NT-F CCCGTAAGTTTGAGTTTATAAGGAA Cd86290NT-R GGATTTGGCAGTGGAACTTG

*Previously constructed clones of *C. albicans ALS* genes were used for assay controls. These are listed below. All control constructs were verified by Sanger sequencing. *CaALS1* = plasmid 857 (full-length *ALS1* amplified from *C. albicans* fosmid 11A4 (Hoyer and Hecht, 2001) and cloned into vector p138NB; Hoyer et al. 1998); *CaALS2* = plasmid 1067 (5’ domain of *ALS2* cloned into p138NB); *CaALS3* = plasmid 848 (5’ domain of *ALS3* cloned into p138NB); *CaALS4* =plasmid 849 (5’ domain of *ALS4* cloned into p138NB); *CaALS5* = plasmid 878 (5’ domain of *ALS5* cloned into p138NB); *CaALS6* = plasmid 773 (5’ domain of *ALS6* cloned into p138NB); *CaALS7* = plasmid 1075 (5’ domain of *ALS7* cloned into pYES2; Thermo Fisher Scientific); *CaALS9-1* = plasmid 2096 (*ALS9* “large allele” cloned into pCRBlunt; Hoyer et al., 2007); *CaALS9-2* = plasmid 2829 (*ALS9* “small allele” cloned into pCRBlunt; Thermo Fisher Scientific).
