# Supplementary material for: Pursuing Advances in DNA Sequencing Technology to Solve a Complex Genomic Jigsaw Puzzle: The Agglutinin-Like Sequence (ALS) Genes of Candida tropicalis
Source: Front Microbiol. 2021 Jan 20;11:594531. doi: 10.3389/fmicb.2020.594531 (PMC7856822; doi:10.3389/fmicb.2020.594531)
Supplement: Supplementary file 1 [file Data_Sheet_1.zip › SupplementaryTableS4.docx]

**SUPPLEMENTARY TABLE S4 |** Evaluation of the specificity of previously published primers for amplification of *C. tropicalis ALS* genes.

Publication Primer Name Target Gene* Sequence (5’ – 3’) Comments

Yu et al., 2016 ALS1 Forward *ALST1* (AF201686) GGGCTCTGGTCGTGATGT Specific for *CtrALS3797*

ALS1 Reverse GTGAGGGAATGAGTCTTG

ALS2 Forward *ALST2* (AF211865) ACTCGTGCCTATACCTAC Specific for *CtrALS1030*

ALS2 Reverse TTGTTGCCGTAATGGTGG

ALS3 Forward *ALST3* (AF211866) AGGTGCTGTAGTTGTTCTT Primers may amplify *CtrALS2293*,

ALS3 Reverse AGCAGTCGGGTTGAAAGG *CtrALS3786*, and *CtrALS3791*

Galán-Ladero et al., 2018 CTRG_1028 F *CTRG_1028*, not “*ALST1*” TGTGCTACCGGTGATTATCATGA Specific for *CtrALS1028*

CTRG_1028 R AATGGTAAAGTTGTGACGCCAGTA

CTRG_02293 F *CTRG_2293*, not “*ALST2*” GGTCCACAATATCCAACATGGA Primers may amplify *CtrALS2293,*

CTRG_02293 R GGCAGATGTAGTGAATTTGAAGACA *CtrALS3786*, and *CtrALS3791*

CTRG_03786 F *CTRG_03786*, not “*ALST3*” AGGTGATACGTTCACTTTGATCATG Primers may amplify *CtrALS2293*,

CTRG_03786 R AGCTGCAGCAAAATAGGCTTGGGT *CtrALS3786*, and *CtrALS3791*

CTRG_03797 F *CTRG_03797*, called “*ALST5*” CGAGCAACGTCAAAAACACTTACT Specific for *CtrALS3797*

CTRG_03797 R GGAATCCAATGCCCAATTAACA

* Partial *ALS* sequences were deduced from PCR amplification of *C. tropicalis* genomic DNA using consensus primers developed from alignment of *C. albicans ALS* sequences (Hoyer et al., 2001). These gene fragments can now be assigned to a complete ORF. In the original manuscript, the gene fragments were named *ALST1* (GenBank accession AF201686.1; now recognized as *CtrALS3797*), *ALST2* (AF211865.1; *CtrALS1030*), and *ALST3* (AF211866.1; *CtrALS2293*). Referring to *CTRG_03797* as “*ALST5*” is most likely a misinterpretation of Supplementary Table 23 from Butler et al. (2009). See **Supplementary Table S5** for more information.
