# Supplementary material for: Pursuing Advances in DNA Sequencing Technology to Solve a Complex Genomic Jigsaw Puzzle: The Agglutinin-Like Sequence (ALS) Genes of Candida tropicalis
Source: Front Microbiol. 2021 Jan 20;11:594531. doi: 10.3389/fmicb.2020.594531 (PMC7856822; doi:10.3389/fmicb.2020.594531)
Supplement: Supplementary file 1 [file Data_Sheet_1.zip › SupplementaryTableS5.docx]

**SUPPLEMENTARY TABLE S5 |** The Als protein family (called Family 17) as presented in Supplementary Table 23 (“Predicted GPI-Protein Cell Wall Families”) from Butler et al. (2009). Purple text was used to indicate predicted proteins that lacked a GPI anchor attachment site. The asterisk was defined just below the species names.

***C. albicans*** ***C. tropicalis*** ***C. parapsilosis*** ***L. elongisporus*** ***M. guilliermondii*** ***C. lusitaniae***

*No orthologs defined in other species can only define as Als-like

Als1 orf19.5741 CTRG_02293* CPAG_00368 LELG_02721 PGUG_03330 CLUG_03274

Als2 orf19.1097 CTRG_03786* CPAG_05056 LELG_05708* PGUG_02302*

Als3 orf19.1816 CTRG_01028* CPAG_05314 LELG_02716* PGUG_03259

Als4 orf19.4555 CTRG_03791 CPAG_00369 LELG_00734* PGUG_00673

Als5 orf19.5736 CTRG_03797* CPAG_05054 LELG_02536*

Als6 orf19.7414 CTRG_02229

Als7 orf19.7400 CTRG_02228*

Als9 orf19.5742 CTRG_01030

CTRG_00941

CTRG_03787

CTRG_03882

CTRG_03871*

CTRG_01041*

CTRG_01038*

In the original table (Butler et al., 2009) and reproduced above, Als protein names were presented to the left of the *C. albicans* ORF numbers. For columns beyond *C. albicans* it was unclear whether reading across rows provided biological insight. In other words, were CTRG_02293, CPAG_00368, LELG_02721, PGUG_03330, and CLUG_03274 all orthologs of *C. albicans* Als1? As another example, should CTRG_03797 be called *C. tropicalis* Als5 with the implication of shared gene expression pattern and/or protein function between the species?

Exploring this question with the *Candida* Gene Order Browser (<http://cgob.ucd.ie/>; Fitzpatrick et al., 2010; Maguire et al., 2013) indicated that some *C. tropicalis* genes shared orthologous physical location with those in *C. albicans*: these instances were marked with a box around the protein name above. These relationships do not extend past *C. tropicalis*, however, so rows in the table should not be interpreted to indicate relationships between the genes/proteins across the fungal species.
